# Half-Sandwich Type Platinum-Group Metal Complexes of *C*-Glucosaminyl Azines: Synthesis and Antineoplastic and Antimicrobial Activities

**DOI:** 10.3390/molecules28073058

**Published:** 2023-03-29

**Authors:** István Kacsir, Adrienn Sipos, Evelin Major, Nikolett Bajusz, Attila Bényei, Péter Buglyó, László Somsák, Gábor Kardos, Péter Bai, Éva Bokor

**Affiliations:** 1 Department of Organic Chemistry, University of Debrecen, P.O. Box 400, H-4002 Debrecen, Hungary; 2 Doctoral School of Chemistry, University of Debrecen, P.O. Box 400, H-4002 Debrecen, Hungary; 3 Department of Medical Chemistry, Faculty of Medicine, University of Debrecen, Egyetem Tér 1., H-4032 Debrecen, Hungary; 4 The Hungarian Academy of Sciences, Center of Excellence, Hungary; 5 MTA-DE Cell Biology and Signaling Research Group ELKH, H-4032 Debrecen, Hungary; 6 Department of Metagenomics, University of Debrecen, H-4032 Debrecen, Hungary; 7 Department of Physical Chemistry, Faculty of Sciences and Technology, University of Debrecen, Egyetem Tér 1., H-4032 Debrecen, Hungary; 8 Department of Inorganic & Analytical Chemistry, Faculty of Sciences and Technology, University of Debrecen, Egyetem Tér 1., H-4032 Debrecen, Hungary; 9 NKFIH-DE Lendület Laboratory of Cellular Metabolism, H-4032 Debrecen, Hungary; 10 Research Center for Molecular Medicine, Faculty of Medicine, University of Debrecen, H-4032 Debrecen, Hungary

**Keywords:** ruthenium, osmium, iridium, rhodium, half-sandwich complex, *C*-glucosaminyl heterocycles, azines, *Staphylococcus aureus*, *Enterococcus*, MRSA, VRE, cytostasis, ovarian cancer

## Abstract

While platinum-based compounds such as cisplatin form the backbone of chemotherapy, the use of these compounds is limited by resistance and toxicity, driving the development of novel complexes with cytostatic properties. In this study, we synthesized a set of half-sandwich complexes of platinum-group metal ions (Ru(II), Os(II), Ir(III) and Rh(III)) with an N,N-bidentate ligand comprising a *C*-glucosaminyl group and a heterocycle, such as pyridine, pyridazine, pyrimidine, pyrazine or quinoline. The sugar-containing ligands themselves are unknown compounds and were obtained by nucleophilic additions of lithiated heterocycles to *O*-perbenzylated 2-nitro-glucal. Reduction of the adducts and, where necessary, subsequent protecting group manipulations furnished the above *C*-glucosaminyl heterocycles in their *O*-perbenzylated, *O*-perbenzoylated and *O*-unprotected forms. The derived complexes were tested on A2780 ovarian cancer cells. Pyridine, pyrazine and pyridazine-containing complexes proved to be cytostatic and cytotoxic on A2780 cells, while pyrimidine and quinoline derivatives were inactive. The best complexes contained pyridine as the heterocycle. The metal ion with polyhapto arene/arenyl moiety also impacted on the biological activity of the complexes. Ruthenium complexes with *p*-cymene and iridium complexes with Cp* had the best performance in ovarian cancer cells, followed by osmium complexes with *p*-cymene and rhodium complexes with Cp*. Finally, the chemical nature of the protective groups on the hydroxyl groups of the carbohydrate moiety were also key determinants of bioactivity; in particular, *O*-benzyl groups were superior to *O*-benzoyl groups. The IC_50_ values of the complexes were in the low micromolar range, and, importantly, the complexes were less active against primary, untransformed human dermal fibroblasts; however, the anticipated therapeutic window is narrow. The bioactive complexes exerted cytostasis on a set of carcinomas such as cell models of glioblastoma, as well as breast and pancreatic cancers. Furthermore, the same complexes exhibited bacteriostatic properties against multiresistant Gram-positive *Staphylococcus aureus* and *Enterococcus* clinical isolates in the low micromolar range.

## 1. Introduction

Registered platinum complexes (cisplatin, oxaliplatin and carboplatin) constitute the backbone of modern oncological chemotherapy in multiple solid tumors with poor prognosis, including a large set of carcinomas, such as ovarian cancer, sarcomas and hematological malignancies [1,2]. The applicability of platinum compounds is limited by platinum resistance and toxicity [3,4,5,6,7,8]. 

Novel organometallic compounds of other platinum-group metals, such as complexes of ruthenium [2,9,10,11,12,13,14,15], osmium [9,11,12,14,16,17,18,19], iridium [11,14,17,20,21] or rhodium [11,14,20,22], are being developed for the replacement of platinum drugs and have been reported to have better toxicity profiles than platinum-based drugs [23,24,25,26]. The anticancer potential of such platinum-group metal complexes is also supported by three ruthenium derivatives, i.e., NAMI-A [27], KP1019/1339 (IT-139, BOLD100) [28] and TLD-1433 [29], which are in different phases of clinical trials against neoplastic diseases such as bladder or lung cancer. 

In the quest for potential substitutes for platins, the half-sandwich type complexes of platinum-group metal ions (e.g., Ru(II), Os(II), Ir(III) and Rh(III)) have emerged as a promising compound class, with a large number of representatives displaying anticancer potencies [14,15,19,21,22]. In addition to the antineoplastic effects, several of these piano-stool complexes have different antimicrobial (e.g., antibacterial [30,31,32,33,34,35,36,37,38,39], antiparasitic [40,41,42], antiviral [30,43] and antifungal [44]) properties.

**Figure 1:**
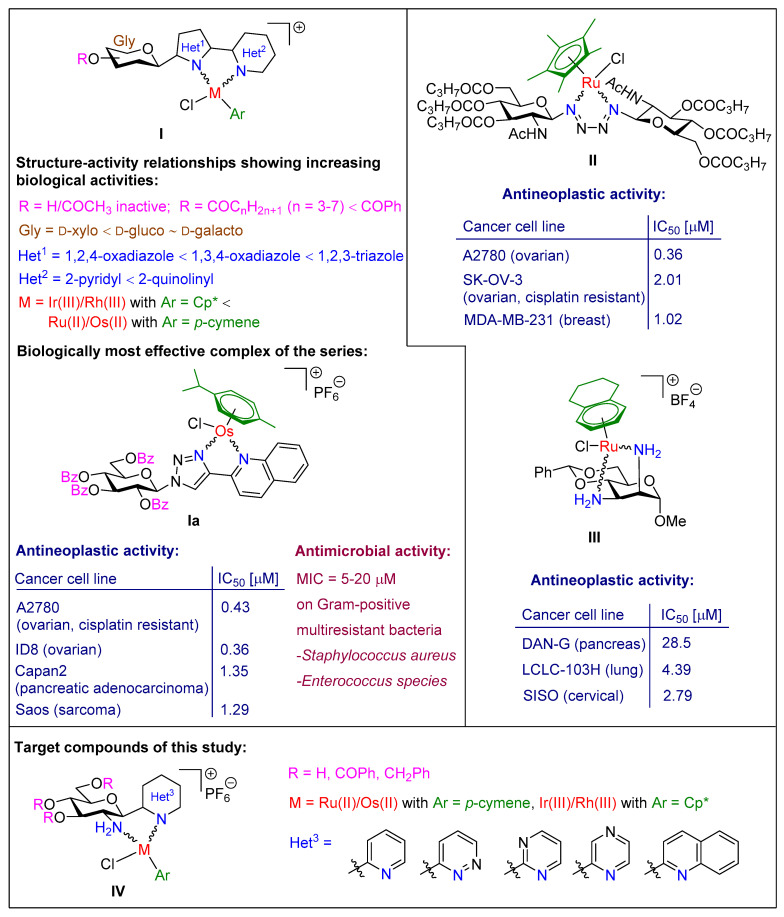
Preliminaries and target compounds of the present work.

We recently reported a series of half-sandwich complexes with five-membered chelate rings constructed with the use of *N*- and *C*-glycopyranosyl heterocyclic N,N-bidentate ligands (Figure 1, **I**) [32,45,46]. Several representatives of **I** displayed low micromolar or, in certain cases, submicromolar (e.g., **Ia**) cytostatic activities against cancer cells, in addition to proving to be selective for such cells. The antiproliferative potency of these complexes is thought to be related to reactive oxygen species production [32,45,46]. It is worth mentioning that the complexes with antineoplastic activities (e.g., **Ia**) were also shown to be effective against Gram-positive multiresistant bacteria [31,32]. A short summary of the structure–activity relationships (SARs) of these complexes is presented in Figure 1, while for a more detailed explanation of the SARs, the reader is referred to our previous publications [31,32,45,46]. One of the most important structural motifs related to biological efficacy is the presence of the sugar moiety *O*-protected with large hydrophobic acyl, preferably with benzoyl groups. This feature contributes, to a large extent, to the favorably increased lipophilic character of the biologically active complexes [32,45,46].

Apart from the above complexes, there are only two more literature examples of half-sandwich complexes with sugar-based N,N-chelators. 1,4-Bis(β-d-glycopyranosyl)tetrazenes [47] (e.g., **II**) and methyl 2,3-diamino-2,3-dideoxy-hexopyranosides [48] (e.g., **III**) were incorporated into the coordination sphere of the reported complexes. The antineoplastic effects of these organometallics were also studied, some of which were found to be cytotoxic at low micromolar concentrations against various cancer cells (**II** and **III** represent the most efficient compounds of the respective series) [47,48]. 

Based on the structures of the sugar-derived ligands of complexes **I** and **III**, we considered that *C*-glucosaminyl N-heterocycles, with an N-donor atom in the glycon and another in the heterocyclic aglycon part, are capable of forming half-sandwich type complexes with a six-membered chelate ring. In the present study, the preparation of a series of *C*-glucosaminyl azines and their incorporation as N,N-bidentate ligands into type **IV** complexes were envisaged. Biological studies were also conducted to reveal the anticancer and antibacterial potencies of the new organometallic compounds. With the exception of the glycosyl heterocyclic ligand, the main structural elements of the new complexes as depicted in formula **IV** were designed to be identical to those of type **I** complexes with biological activities. In addition, the replacement of the *O*-benzoyl groups of the monosaccharide unit by *O*-benzyl groups was envisaged in order to examine the effect of ether-type protection on the biological efficiency of the complexes.

## 2. Results

### 2.1. Chemistry

For the preparation of the planned *C*-glucosaminyl azine-type N,N-chelators, 3,4,6-tri-*O*-benzyl-2-nitro-d-glucal [49] (**1**) was used as the starting material with the expectation that a common general procedure can be elaborated with additions to the double bond of organometallic nucleophiles derived from the respective heterocycles [50]. In line with these plans, nitro-Michael addition [51,52,53] of **1** with lithiated six-membered heterocycles preformed from the corresponding halogenated heterocycles with *n*-butyl lithium or generated in situ (Table 1, *i*) resulted in a set of *C*-(2′-deoxy-2′-nitro-3′,4′,6′-tri-*O*-benzyl-β-d-glucopyranosyl)azines (**2a–e**) in good to acceptable yields. 

In the next step, the reduction of the nitro group of compounds **2** showed much less uniform behavior. To achieve *O*-perbenzylated *C*-glucosaminyl azines, reduction of the nitro group of **2a–e** by Zn-HCl (*ii*) was investigated first. This transformation of **2a** to the expected 2-glucosaminyl pyridine (**3a**) was smoothly accomplished in good yield. However, similar reactions of nitro derivatives **3b–e** led to multicomponent reaction mixtures, from which only the 2-glucosaminyl pyrazine **3d** could be isolated in low yield. Unfortunately, our further attempts to obtain the glucosamine derivatives **3d,e** from **2d,e** under different reductive conditions also failed; either no reaction took place (Fe, *cc*HCl, THF-H_2_O 1:1, 0 °C; SnCl_2_, dry EtOH, reflux; SiCl_3_H, DIPEA, dry CH_3_CN, 0 °C; B_2_(OH)_4_, THF-H_2_O 1:1, 80 °C) or formation of inseparable product mixtures (Sn, *cc*HCl, THF-H_2_O 1:1, 0 °C) was observed.

Next, the synthesis of *O*-unprotected *C*-glucosaminyl azines was examined starting from compounds **2a–e** and **3a** (Table 2). A BCl_3_-mediated *O*-debenzylation of the pyridine derivative **3a** afforded the *O*-unprotected analog **5a** in moderate yield (Table 2, *i*). This reaction was repeated in the presence of the cation scavenger pentamethylbenzene [54] (*ii*) to afford compound **5a** in excellent yield. 

Attempted transformation of compounds **2a–e** into **5a–e** in one step via simultaneous *O*-debenzylation and reduction of the nitro group by catalytic hydrogenation (Pd(C) or Pd(OH)_2_, cat. *cc*HCl, EtOH, reflux,) was unsuccessful; in each case, decomposition of the starting materials (**2a–e**) was observed. Therefore, the synthesis of the glucosamine derivatives **5a–e** from **2a–e** was performed in a two-step procedure. First, the *O*-benzyl-protecting groups of **2a–e** were removed by BCl_3_ (*i*) to afford *C*-(2′-deoxy-2′-nitro-β-d-glucopyranosyl)azines **4a–e** in high yields. Reduction of the nitro group of **4a–e** was then examined under two conditions. Catalytic hydrogenation of **4c** and **4d** (*iii*) afforded the desired pyrimidine- and pyrazine-containing *C*-glucosaminyl derivatives **5c** and **5d**, respectively, in acceptable yields. The same conditions (*iii*) applied to compounds **4a,b,e** resulted in complex product mixtures, in which the desired *C*-glucosaminyl pyridine **5a**, pyridazine **5b** and quinoline **5e** were detected by TLC analysis; however, they could not be separated in a pure state. Treatment of **4e** with Sn powder in the presence of *cc*HCl (*iv*) was also carried out to afford the target **5e** in acceptable yield.

In order to obtain *O*-perbenzoylated *C*-glucosaminyl azines, a direct exchange of the *O*-benzyl protecting groups with benzoyl groups by a Zn(OTf)_2_-mediated reaction [55] of compounds **2a–d** with benzoyl chloride (Table 3, *i*) was performed to afford *C*-(2′-deoxy-2′-nitro-3′,4′,6′-tri-*O*-benzoyl-β-d-glucopyranosyl)azines **6a–d** in good to excellent yields. Subsequent reduction of the nitro group of the pyridine derivative **6a** by Zn-HCl (*ii*) afforded the *O*-perbenzoylated glucosamine derivative **7a** in moderate yield. Analogous reactions (*ii*) carried out with compounds **6b–d** led to complex reaction mixtures, from which the desired *C*-glucosaminyl heterocycles **7b–d** could not be isolated. For the transformation of **6b–d** into **7b–d**, further experiments were conducted under various reductive conditions (e.g., H_2_, Pd(C) or Pd(OH)_2_, dry EtOH, reflux; SnCl_2_, dry EtOH, reflux; Sn, *cc*HCl, THF-H_2_O 1:1, 0 °C; B_2_(OH)_4_, THF-H_2_O 1:1, 80 °C); however, none of these experiments was successful. 

Due to the above difficulties, another three-step procedure starting from **5c-e** was applied to obtain the planned **7c–e** (Table 3). Thus, the NH_2_ group of **5c-e** was protected first as a carbamate using Boc_2_O (*iv*), and the resulting **8c-e** were *O*-perbenzoylated upon treatment with benzoyl chloride (*v*) to afford the *O*- and *N*-protected glucosaminyl derivatives **9c–e**. Finally, acid-mediated liberation of the NH_2_ group in **9c–e** (*vi*) was carried out, providing the final products **7c-e** in high yields.

As mentioned earlier, the 3-(2′-amino-2′-deoxy-β-d-glucopyranosyl)pyridazine **5b** could not be obtained in a pure state from **4b** (Table 2). In order to obtain the *O*-perbenzoylated **9b**, a consecutive three-step procedure starting from **4b** was conducted to avoid the need to use pure intermediate **5b** (Table 3). Thus, the NO_2_→NH_2_ transformation was carried out by catalytic hydrogenation of **4b** (Table 3, *iii*), followed by the Boc protection of the amino group of intermediate **5b** (*iv*); subsequent *O*-perbenzoylation of the resulting **8b** (*v*) furnished the desired *C*-glucosaminyl pyridazine **9b** in acceptable overall yields (27% for three steps). Then, standard *N*-Boc deprotection (*vi*) afforded the desired **7b** in high yield. 

Next, the newly prepared heterocyclic glucosamine derivatives were used as N,N-bidentate ligands in the formation of platinum-group metal half-sandwich complexes. 

Treatment of *O*-perbenzylated 2-glucosaminyl pyridine with dichloro(η^6^-*p-*cymene)ruthenium(II) and -osmium(II) and dichloro(pentamethylcyclopentadienyl)iridium(III) and -rhodium(III) dimers (**Ru-/Os-/Ir-/Rh-dimer**) in the presence of the halide abstractor TlPF_6_ afforded the expected cationic complexes **Ru-3a**, **Os-3a**, **Ir-3a** and **Rh-3a**, respectively, with six-membered chelate rings (Table 4, entries 1–4). Analogous Ru(II) and Os(II) complexes **Ru-3d** and **Os-3d** with the pyrazine derivative **3d** were also obtained under similar conditions (entries 5 and 6, respectively).

Our previous studies [45,46] on other series of half-sandwich complexes constructed with glycopyranosyl azole ligands revealed that the *O*-protection of the hydroxyl groups of the sugar moiety by large, apolar protecting groups played a pivotal role in achieving significant biological effects. Complexes containing *O*-unprotected monosaccharide-based ligands proved to be biologically inactive. Nevertheless, for a comparative study of the new set of platinum-group metal complexes presented here, compound **Ru-5a** incorporating *O*-deprotected 2-glucosaminyl pyridine **5a** was also synthesized (Table 4, entry 7).

Complexations of the *O*-perbenzoylated *C*-glucosaminyl azines **7a–e** with the dimeric chloro-bridged platinum-group metal complexes **Ru-dimer**, **Os-dimer**, **Ir-dimer** and **Rh-dimer** were also performed under the same conditions as described above to afford the expected half-sandwich type complexes **Ru-7a–Ru-7e**, **Os-7a–Os-7e**, **Ir-7a–Ir-7e** and **Rh-7a–Rh-7e**, respectively, in good to excellent yields (Table 5). 

In most of the complexations presented in Table 4 and Table 5, a single diastereoisomer of the complexes was formed. As an exception, the reactions of **7b** yielded complexes **Ru-7b**, **Os-7b**, **Ir-7b** and **Rh-7b** as mixtures of two diastereoisomers (Table 5, entries 5–8).

A single crystal of complex **Ru-3a** was obtained by slow evaporation of a CHCl_3_-MeOH solvent mixture. A search of the Cambridge Structural Database (Ver 5.43, Update November 2021) [56] resulted in 98 hits for similar Ru·Cl·η^6^··NH_2_··N coordination. However, our structure is unique, as the Ru-Cl distance is the shortest in this family of compounds by 2.374(5) Å (average: 2.416(15) Å), while the angle of the arene ring and the N-N-Ru plane is rather high, at 60.7° (average: 57(2)°). Moreover, none of the hits contains a pyranose ring attached to one coordinated amino nitrogen atom in any position. A more detailed search for Ru·Cl·η^6^··NH_2_ coordination revealed more than 200 hits; the Ru-Cl distance was also in the very short region, with an average of 2.42 Å. Ring puckering analysis [57] indicates that the C1-O5 ring has a chair conformation (Θ = 19.6(18)°, Φ = 275(5)°), which is in agreement with the NMR data related to the coupling constants of the proton resonances of the sugar skeleton. 

**Figure 2:**
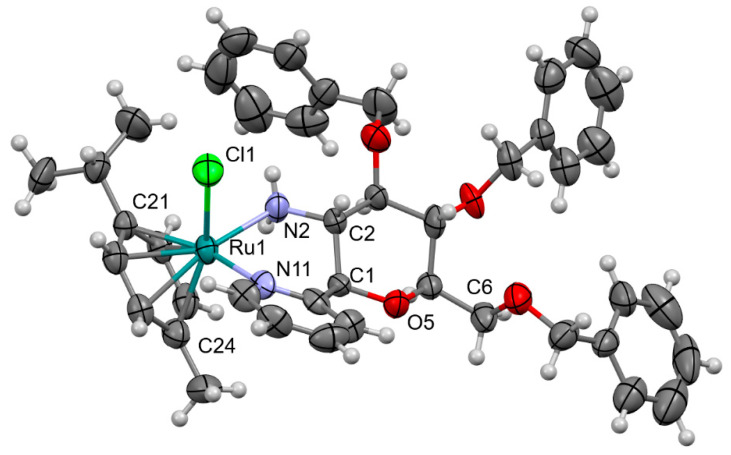
ORTEP view of **Ru-3a** at a 50% probability level with a partial numbering scheme. The PF_6_^−^ counter ion is omitted for clarity. Selected bond distance (Å) and bond length (^o^) data: Ru(1)-N(2) 2.153(13), Ru(1)-N(11) 2.091(14), Ru(1)-Cl(1) 2.374(5), Ru(1)-C_Ar_(avg) 2.180(16), C(1)-C(2) 1.536(19), N(11)-Ru(1)-N(2) 87.3(5), N(11)-Ru(1)-Cl(1) 85.0(4), N(2)-Ru(1)-Cl(1) 82.4(4). For further details, see the Supplementary Materials.

X-ray crystallography analysis of **Ru-3a** provided unequivocal evidence of the existence of a six-membered chelate ring and revealed the spatial arrangement of structural elements in the coordination sphere of the Ru(II) ion (Figure 2). Thus, following the general convention [58], the absolute configuration of the stereogenic Ru(II) was assigned as *R*. The absolute configuration is confirmed by the analysis of the anomalous dispersion data as the Flack parameter [59] (Table S3). 

**Figure 3:**
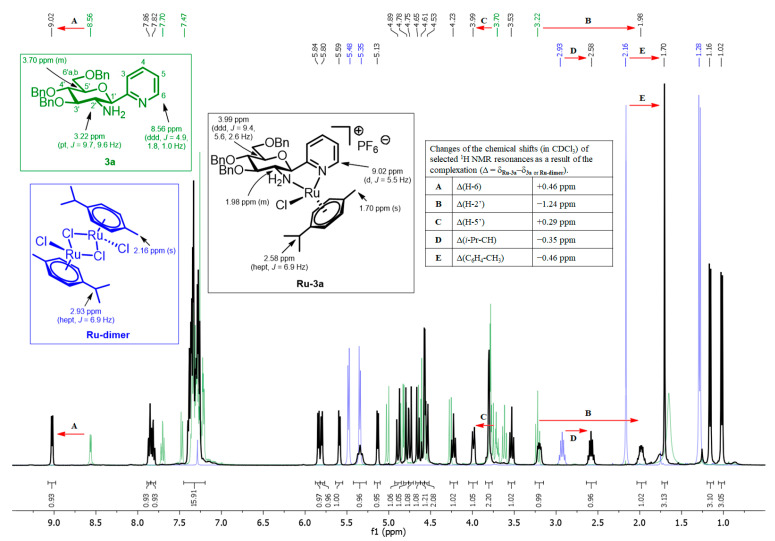
Superposition of the ^1^H-NMR spectra of ligand **3a** (green) and complexes **Ru-dimer** (blue) and **Ru-3a** (black) in CDCl_3_, highlighting the changes in some characteristic resonances as a result of the complexation.

For structural elucidation of the prepared compounds, ^1^H and ^13^C NMR measurements were also performed. Comparison of the ^1^H NMR spectroscopic data of the complexes to those of the starting **Ru/Os/Ir/Rh dimers** and the *C*-glucosaminyl heterocyclic ligands revealed several significant changes in the chemical shifts, some of which are representatively highlighted by the superposition of the ^1^H NMR spectra of **Ru-dimer**, **3a** and **Ru-3a** (Figure 3). 

As a consequence of the complexation, the H-2’ signal of the sugar skeleton of **3a** shifted upfield by 1.2 ppm (**B**), while the H-5′ resonance showed a 0.3 ppm downfield shift (**C**). Such changes in the chemical shifts of H-2′ and H-5′ are characteristic not only of **Ru-3a** but also of all complexes isolated as a single isomer (**Os/Ir/Rh-3a**, **Ru/Os-3d**, **Ru-5a**, **Ru/Os/Ir/Rh-7a** and **Ru/Os/Ir/Rh-7c-d**) and the major component of the diastereomeric mixtures of **Ru/Os/Ir/Rh-7b** (Δ = δ_complex_−δ_ligand_ = (−1.3)–(−0.5) ppm for H-2′ and (+0.3)–(+0.6) ppm for H-5′; Table S1). It should be noted that the formation of the minor stereoisomers of **Ru/Os/Ir/Rh-7b** from ligand **7b** resulted in practically no (for **7b → Ir/Rh-7b**) or less significant changes (+0.15 ppm for **7b → Ru/Os-7b**) in the chemical shift of the H-5′ signal (Table S1). 

The transformation of **Ru-dimer** into **Ru-3a** significantly affected the proton resonances of the *p*-cymene moiety. For example, the signal of C*H_3_* attached to the benzene ring displayed remarkable upfield shifts (0.46 ppm) as a result of the complexation (**E**). A similar trend in the appearance of this C*H_3_* signal was observed for all single isomeric Ru(II) and Os(II) complexes (**Os/Ir/Rh-3a**, **Ru/Os-3d**, **Ru-5a**, **Ru/Os/Ir/Rh-7a** and **Ru/Os/Ir/Rh-7c-d**) and for the main components of complexes **Ru-7b** and **Os-7b** (Δ = δ_complex_ − δ_dimer_ = (−0.3)–(−0.6) ppm for C_6_H_4_-C*H_3_*, Table S1). In the case of the minor isomers of **Ru-7b** and **Os-7b**, the same signal indicated a slight downfield shift (Δ = δ_complex_ − δ_dimer_ = ~+0.1 ppm, Table S1) relative to that of the corresponding **Ru-dimer** and **Os-dimer**, respectively.

These data strongly suggest that in each complex obtained as a single isomer (**Os/Ir/Rh-3a**, **Ru/Os-3d**, **Ru-5a**, **Ru/Os/Ir/Rh-7a** and **Ru/Os/Ir/Rh-7c-d**) and in the major component of **Ru/Os/Ir/Rh-7a**, the absolute configuration of the metal center is identical to that of the reference complex, **Ru-3a**. 

A more detailed collection of the comparative spectroscopic data are presented in Table S1 in the Supplementary Materials.

### 2.2. Cell Biology

#### 2.2.1. *C*-Glucosaminyl Azines Exert Cytostatic Activity

The complexes described above are intended to replace registered platinum complexes. Platinum complexes constitute the core of the chemotherapy regimen used in ovarian cancer [8]; therefore, we used a cellular model of ovarian cancer, A2780, and primary human dermal fibroblasts as models of non-transformed cells (controls). For the characterization of the complexes, we used an MTT assay after 4 h of treatment for the detection of early toxicity and an SRB assay 48 h after treatment for the detection of cytostasis [60,61,62].

**Figure 4:**
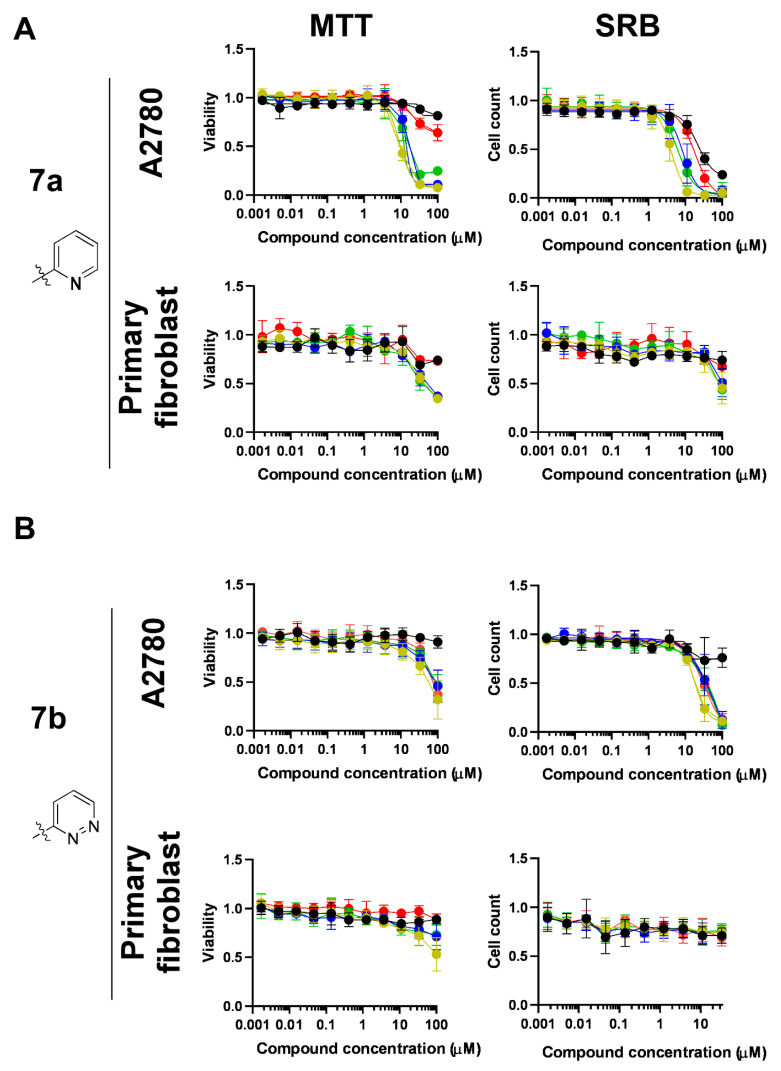
Assessment of free ligands **7a,b** and their complexes (**Ru/Os/Ir/Rh-7a** and **Ru/Os/Ir/Rh-7b**) for cytotoxic and cytostatic activity (panel (**A**) for compound **7a** and its complexes; panel (**B**) for compound **7b** and its complexes). For MTT assays, 3 × 10^3^ A2780 cells or 4 × 10^3^ primary fibroblasts were plated in 96-well plates. For SRB assays, 1.5 × 10^3^ A2780 cells or 2 × 10^3^ primary fibroblasts were plated in 96-well plates. Cells were treated with the compounds in the concentrations indicated for either 4 h for the MTT assay or for 48 h for the SRB assay. Data are represented as average ± SD from three biological replicates; individual assays were performed in duplicate. Values were normalized for vehicle-treated cells; absorbance for vehicle-treated cells equals 1. Statistical significance was assessed using a one-way ANOVA test comparing all values to the lowest concentration of a compound. Before the test, normality was assessed using the Shapiro–Wilk test, and the post hoc test was chosen accordingly. For better visibility, the *p* values and distributions are presented in an Excel sheet available at https://figshare.com/s/9ec2a005e6b9e5874c07. Nonlinear regression was performed on the datasets indicated in Table 6. Color code: black—free ligand (**7a** or **7b**), khaki—ruthenium complex (**Ru-7a** or **Ru-7b**), blue—osmium complex (**Os-7a** or **Os-7b**), green—iridium complex (**Ir-7a** or **Ir-7b**), red—rhodium complex (**Rh-7a** or **Rh-7b**).

**Figure 5:**
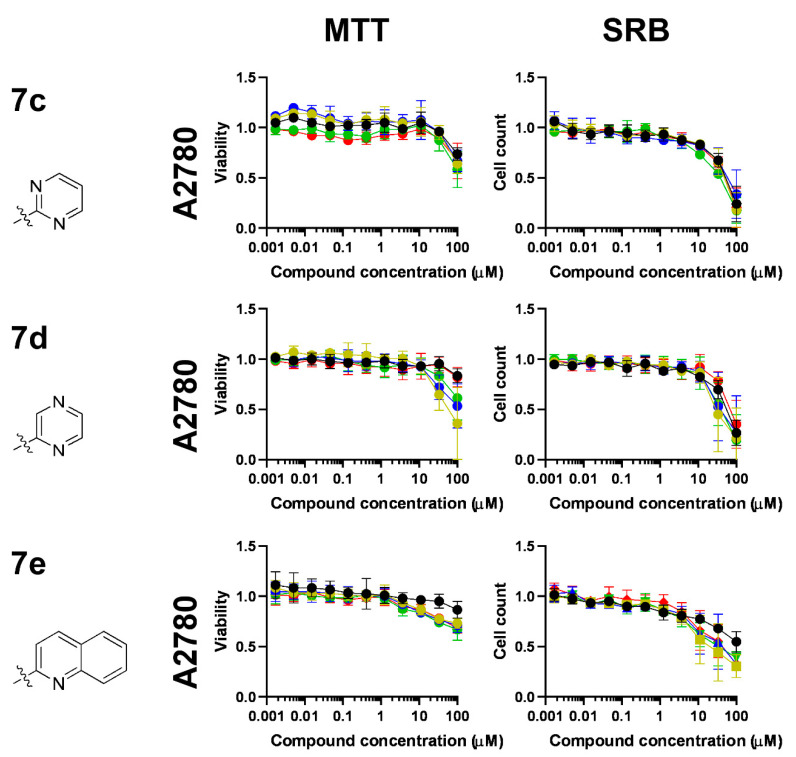
Assessment of free ligands **7c–e** and their complexes (**Ru/Os/Ir/Rh-7c**, **Ru/Os/Ir/Rh-7d** and **Ru/Os/Ir/Rh-7e**) for cytotoxic and cytostatic activity. For the MTT and SRB assays, 3 × 10^3^ A2780 cells and 1.5 × 10^3^ A2780 cells were plated in 96-well plates, respectively. Cells were treated with the compounds in the concentrations indicated for either 4 h for the MTT assay or for 48 h for the SRB assay. Data are represented as average ± SD from three biological replicates; individual assays were performed in duplicate. Values were normalized for vehicle-treated cells; absorbance for vehicle-treated cells equals 1. Statistical significance was assessed using a one-way ANOVA test comparing all values to the lowest concentration of a compound. Before the test, normality was assessed using the Shapiro–Wilk test, and the post hoc test was chosen accordingly. For better visibility, the *p* values and distributions are presented in an Excel sheet available at https://figshare.com/s/9ec2a005e6b9e5874c07. Nonlinear regression was performed on the datasets indicated in Table 6. Color code: black—free ligand (**7c** or **7d** or **7e**), khaki—ruthenium complex (**Ru-7c** or **Ru-7d** or **Ru-7e**), blue—osmium complex (**Os-7c** or **Os-7d** or **Os-7e**), green—iridium complex (**Ir-7c** or **Ir-7d** or **Ir-7e**), red—rhodium complex (**Rh-7c** or **Rh-7d** or **Rh-7e**).

First, we assessed the complexes of ligands **7a–e**. The complexes of the pyridine- and pyridazine-containing ligands **7a** and **7b** had superior bioactivity relative to that of the complexes of ligands **7c**, **7d** and **7e**, with pyrimidine, pyrazine and quinoline aglycon parts, respectively (Figure 4 and Figure 5, Table 6). Complexes of **7a–e** induced early toxicity, as evidenced by the MTT assays. The complexes of the ligand **7a** were the most effective in inducing early toxicity, with IC_50_ values ranging between 9 and 14 µM and achieving more than 90% inhibition (Figure 4, Table 6). Other complexes did not reach over 90% inhibition, although they induced early toxicity (Figure 4 and Figure 5, Table 6). In terms of long-term cytostatic activity, the complexes of **7a** and **7b** exerted complete inhibition of cell growth in SRB assays, with IC_50_ values between 4 and 9 µM (Figure 4, Table 6). Complexes of ligands **7c–e** did not inhibit cell proliferation fully up to 100 μM (Figure 5, Table 6). The best IC_50_ values fell into the low micromolar range; **Ru-3a** and **Ir-3a** had IC_50_ values of 1.86 and 1.69 μM, respectively.

In general, the IC_50_ values of the Ru(II)-containing complexes were lower than those of the Os(II), Ir(III) and Rh(III) analogs constructed with the same ligand (e.g., **Ru-7a** vs. **Ir-7a**, **Os-7a** and **Rh-7a**; Table 6). In terms of effectiveness, the Ru-containing complexes were followed by the corresponding iridium complexes, then by the osmium and, finally, by the rhodium complexes (Figure 4 and Figure 5, Table 6). Of note, the difference between Ru, Os, Ir and Rh complexes was not as pronounced as we observed in our prior studies with glycosyl azole-type ligands [32,45,46]. Furthermore, the free ligands **7a**, **7c**, **7d** and **7e** effectively induced cytostasis (Figure 4 and Figure 5, Table 6).

We further investigated the effects of complexes of **7a** and **7b** on human primary dermal fibroblasts, as these complexes were efficient in inducing cytostasis. Testing the complexes on primary, non-transformed cells informed us of the selectivity of the complexes between transformed cancer cells modelled by A2780 cells and primary, non-transformed cells modelled by primary fibroblasts. The complexes of **7a** and **7b** induced early toxicity (10–70% maximal inhibition) and long-term cytostasis in primary dermal fibroblasts (25–60% maximal inhibition) although with lower efficacy than in A2780 cells (Figure 4, Table 6).

#### 2.2.2. *O*-Benzyl Protective Groups Improve, While the *O*-Deprotection Abrogates the Cytostatic Activity of the Complexes

Next, we assessed the complexes constructed by the *O*-perbenzylated *C*-glucosaminyl heterocycles **3**. Due to the synthetic difficulties of this series of ligands, only the complexes of the pyridine and pyrazine derivatives **3a** and **3d** were available for further analysis.

**Figure 6:**
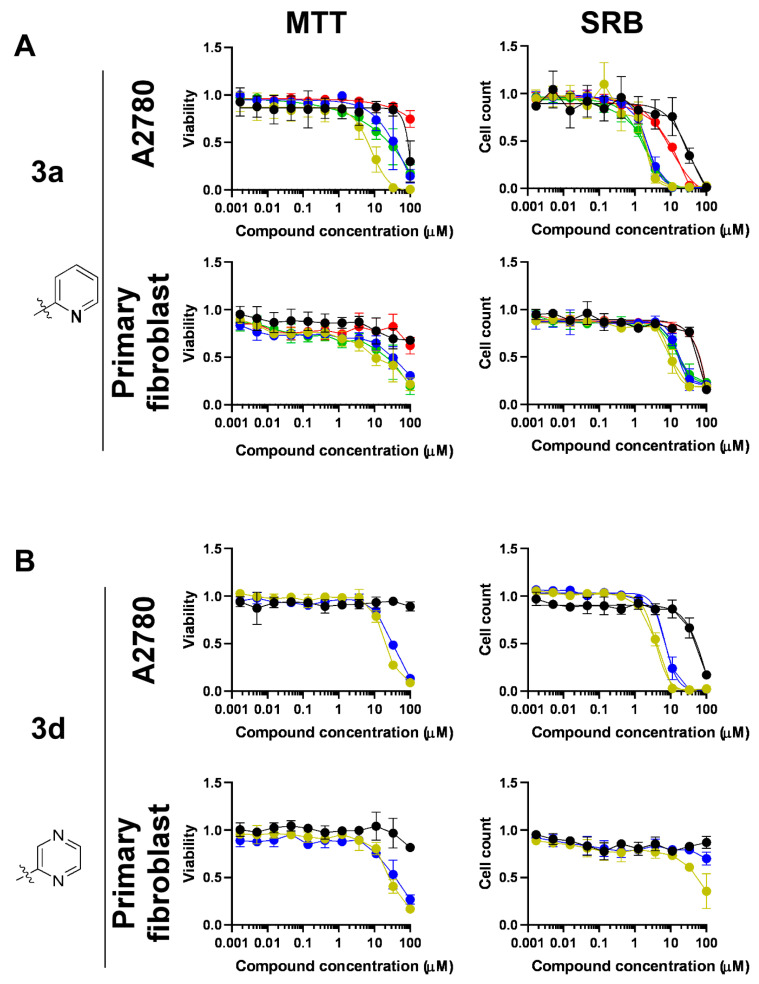
Assessment of the free ligands **3a** and **3d** and their complexes (**Ru/Os/Ir/Rh-3a** and **Ru/Os-3d**) for cytotoxic and cytostatic activity (panel (**A**) for compound **3a** and its complexes; panel (**B**) for compound **3d** and its complexes). For the MTT assays, 3 × 10^3^ A2780 cells or 4 × 10^3^ primary fibroblasts were plated in 96-well plates. For SRB assays, 1.5 × 10^3^ A2780 cells or 2 × 10^3^ primary fibroblasts were plated in 96-well plates. Cells were treated with the compounds in the concentrations indicated for either 4 h for the MTT assay or for 48 h for the SRB assay. Data are represented as average ± SD from three biological replicates; individual assays were performed in duplicate. Values were normalized for vehicle-treated cells; absorbance for vehicle-treated cells equals 1. Statistical significance was assessed using a one-way ANOVA test comparing all values to the lowest concentration of a compound. Before the test, normality was assessed using the Shapiro–Wilk test, and the post hoc test was chosen accordingly. For better visibility, the *p* values and the distributions are presented in an Excel sheet available at https://figshare.com/s/9ec2a005e6b9e5874c07. Nonlinear regression was performed on the datasets indicated in Table 6. Color code: black—free ligand (**3a** or **3d**), khaki—ruthenium complex (**Ru-3a** or **Ru-3d**), blue—osmium complex (**Os-3a** or **Os-3d**), green—iridium complex (**Ir-3a**), red—rhodium complex (**Rh-3a**).

The complexes of **3a**, such as **Ru-3a**, **Os-3a** and **Ir-3a**, exerted considerable early toxicity as judged by MTT assays; **Ru-3a** induced early toxicity on A2780 cells, with an IC_50_ value of 8.02 μM (Figure 6, Table 6). Interestingly, **Rh-3a**-induced early toxicity was negligible on A2780 cells (Figure 6, Table 6). With regard to cytostasis, complexes **Ru-3a**, **Os-3a**, **Ir-3a** and **Rh-3a** were cytostatic on A2780 cells, with micromolar IC_50_ values, whereas ligand **3a** had an IC_50_ value above 30 μM (Figure 6A, Table 6). The complexes were active on primary human dermal fibroblasts. **Ru-3a**, **Os-3a** and **Ir-3a** exerted ≥70% inhibition in MTT assays, while **3a** and **Rh-3a** exerted ~30–40% inhibition (Figure 6A, Table 6). In SRB assays, **3a** and all corresponding complexes (**Ru-3a**, **Os-3a**, **Ir-3a** and **Rh-3a**) exerted full inhibition, and for **Ru-3a**, **Os-3a** and **Ir-3a**, it was possible to determine the IC_50_ values that fell into the range of ~10–15 μM (Figure 6A, Table 6).

Next, we assessed **3d** and its ruthenium and osmium complexes, **Ru-3d** and **Os-3d**. **Ru-3d** and **Os-3d** exerted early toxicity on A2780 and human primary dermal fibroblast cells in low micromolar concentrations (Figure 6B, Table 6). The ligand **3d** had no activity in MTT assays on A2780 and human primary dermal fibroblast cells in low micromolar concentrations (Figure 6, Table 6). Similar to their activity in the MTT assay, **Ru-3d** and **Os-3d** exerted early toxicity in SRB assays on A2780 cells in low micromolar concentrations, while **3d** did not exert considerable activity in SRB assays on A2780 cells (Figure 6B, Table 6). In contrast to their activity in MTT assays, **3d** and **Os-3d** did not inhibit cell proliferation in SRB assays, and **Ru-3d** had only a minor effect on primary human dermal fibroblasts (Figure 6B, Table 6).

**Figure 7:**
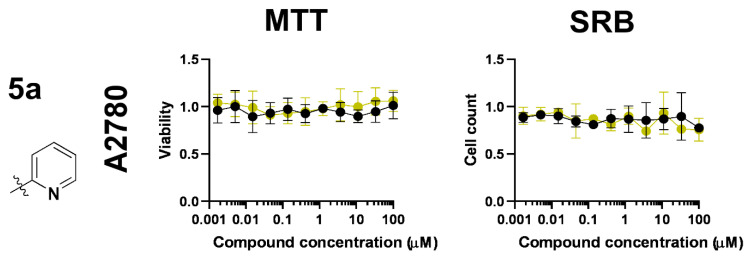
Assessment of the free ligand **5a** and its ruthenium complex, **Ru-5a**, for cytotoxic and cytostatic activity. For MTT and SRB assays, 3 × 10^3^ A2780 cells and 1.5 × 10^3^ A2780 cells were plated in 96-well plates, respectively. Cells were treated with the compounds in the concentrations indicated for either 4 h for the MTT assay or for 48 h for the SRB assay. Data are represented as average ± SD from three biological replicates; individual assays were performed in duplicate. Values were normalized for vehicle-treated cells; absorbance for vehicle-treated cells equals 1. Statistical significance was assessed using a one-way ANOVA test comparing all values to the lowest concentration of a compound. Before the test, normality was assessed using the Shapiro–Wilk test, and the post hoc test was chosen accordingly. For better visibility, the *p* values and the distributions are presented in an Excel sheet available at https://figshare.com/s/9ec2a005e6b9e5874c07. Nonlinear regression was performed on the datasets indicated in Table 6. Color code: black—free ligand (**5a**), khaki—ruthenium complex (**Ru-5a**).

We assessed the effect of the *O*-deprotection of the carbohydrate moiety, which was shown to abrogate the bioactivity, similar to our previous observations [45,46,47]. We used the free ligand, **5a**, which is the deprotected equivalent of **3a** and **7a**, and its ruthenium complex, **Ru-5a**. Ligand **5a** and its ruthenium complex, **Ru-5a**, did not exhibit any biological activity on A2780 cells either in MTT or SRB assays (Figure 7, Table 6).

#### 2.2.3. Compound **Ru-3a** Is Cytostatic in Multiple Carcinoma Cell Lines

Carbohydrate-containing ruthenium, osmium and iridium complexes with similar structures were shown to be effective in a large set of carcinoma, sarcoma and lymphoma cell lines [32,45,46,47,63,64,65,66,67]; therefore, we assessed the bioactivity of these complexes in other carcinoma cell lines. For this assay, we chose **Ru-3a** and the ligand, **3a**, as this complex had one of the best IC_50_ values in A2780 cells (IC_50_ = 1.86 μM) and had the best-performing protective group attached. 

**Figure 8:**
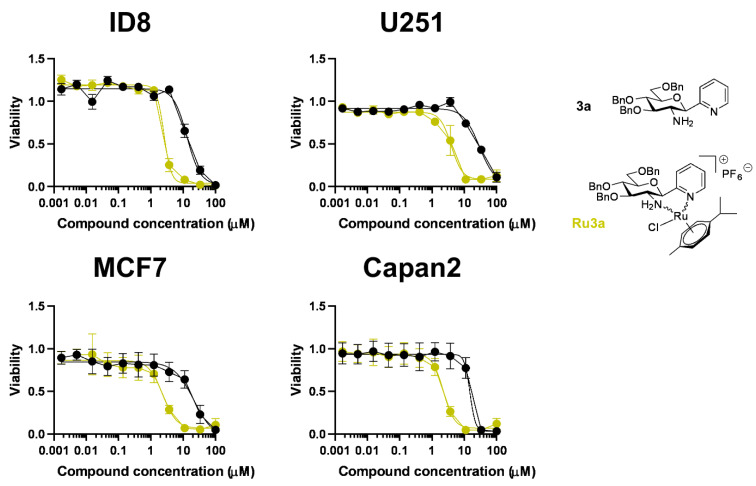
Assessment of free ligand **3a** and complex **Ru-3a** for cytostatic activity in multiple carcinomas. For this assay, 2 × 10^3^ ID8, 3 × 10^3^ U251, 3 × 10^3^ MCF7 or 3 × 10^3^ Capan2 cells were plated in 96 well plates. Cells were treated with the compounds in the concentrations indicated for 48 h; then, an SRB assay was performed. Data are represented as average ± SD from three biological replicates; individual assays were performed in duplicates. Values were normalized for vehicle-treated cells; absorbance for vehicle-treated cells equals 1. Statistical significance was assessed using a one-way ANOVA test comparing all values to the lowest concentration of a compound. Before the test, normality was assessed using the Shapiro–Wilk test, and the post hoc test was chosen accordingly. For better visibility, the *p* values and the distributions are presented in an Excel sheet available at https://figshare.com/s/9ec2a005e6b9e5874c07. Nonlinear regression was performed on the datasets indicated in Table 7.

In agreement with the data presented in Figure 6, **Ru-3a** inhibited the proliferation of another ovarian cancer cell line (ID8), a glioblastoma cell line (U251), a breast carcinoma cell line (MCF7) and a pancreatic adenocarcinoma cell line (Capan2), with IC_50_ values in the low micromolar range falling between 2 and 4 μM (Figure 8, Table 7). Importantly, **3a** was also active in these cell lines, with IC_50_ values falling between 10 and 30 μM (Figure 8, Table 7).

#### 2.2.4. Complexes with Cytostatic Properties Are Bacteriostatic on Gram-Positive Multiresistant *Staphylococcus aureus* and *Enterococcus isolates*

**Figure 9:**
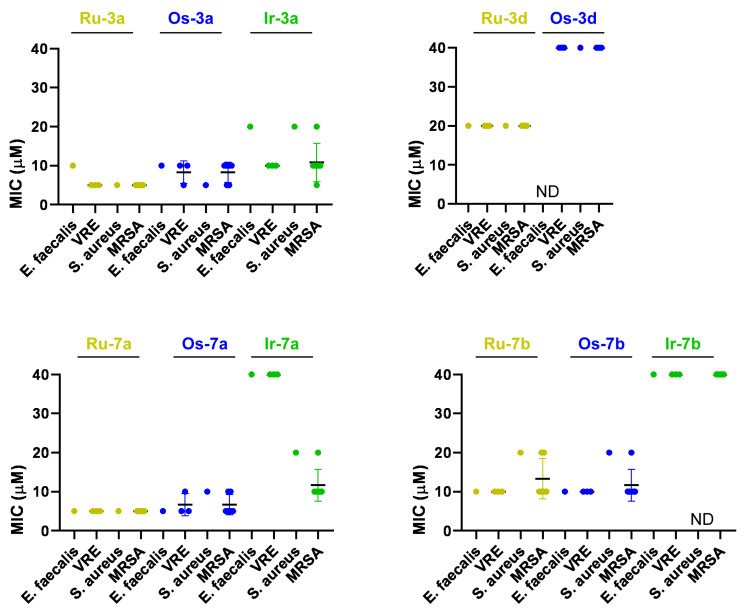
Complexes of the *C*-glucosaminyl heterocycles **3a**, **3d**, **7a** and **7b** exert bacteriostatic activity against reference strains and clinical VRE and MRSA isolates. The MIC values of the complexes were determined against the reference strains of *S. aureus* (ATCC29213) and *E. faecalis* (ATCC29212) and against clinical VRE and MRSA isolates by microdilution assays (repeated at least twice in duplicate), as described in Materials and Methods. Abbreviations: MRSA—methicillin-resistant *Staphylococcus aureus*, VRE—vancomycin-resistant *Enterococcus*, ND—not detected, MIC > 40 μM, Veh—vehicle. Color code: khaki—ruthenium complex, blue—osmium complex, green—iridium complex.

Prior investigations by us [31,32] and others [30,31,32,33,34,35,36,37,38,39,68,69,70,71,72,73,74,75] showed that complexes of the platinum-group metals (platinum, palladium, ruthenium, osmium, iridium and rhodium) can exert bacteriostatic activity. Furthermore, we showed that those compounds were bacteriostatic and cytostatic in neoplasia models [31,32,45,46]. Therefore, we assessed **Ru/Os/Ir/Rh**-**3a**, **Ru/Os**-**3d**, **Ru/Os/Ir/Rh-7a** and **Ru/Os/Ir/Rh-7b** complexes and the corresponding free ligands. Free ligands and rhodium complexes did not exert bacteriostatic activity on any of the investigated strains or isolates. In contrast, the remaining ruthenium, osmium and iridium complexes (**Ru/Os/Ir**-**3a**, **Ru/Os**-**3d**, **Ru/Os/Ir-7a** and **Ru/Os/Ir-7b**) exhibited bacteriostatic activity on the reference strain of *Enterococcus faecalis* and *Staphylococcus aureus*, VRE and MRSA, with the exception of **Os-3d** on the reference strain of *Enterococcus faecalis* and **Ir-7b** on the reference strain of *Staphylococcus aureus* (Figure 9, Table 8). Ruthenium and osmium complexes were characterized by the lowest MIC values, followed by iridium complexes. The ruthenium complexes of **3a** and **7a** had the lowest MIC values on the reference strain and clinical isolates of *Staphylococcus aureus* and *Enterococcus faecalis*, highlighting the superior performance of pyridine-containing complexes in terms of their bacteriostatic activity, similar to their higher performance as cytostatic agents. 

## 3. Discussion

**Figure 10:**
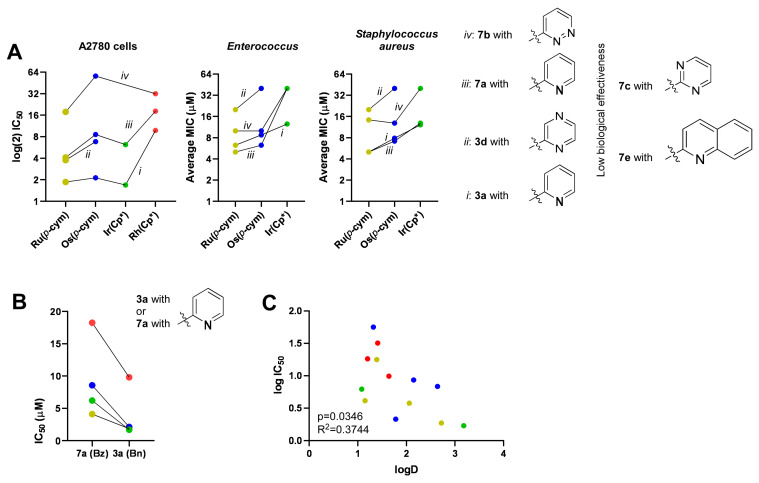
Correlations between the structural features and the bioactivity of the complexes with *O*-protected *C*-glucosaminyl azines in neoplastic cell models. (**A**) The effect of the heterocyclic aglycon and the arene/arenyl metal ion parts on the IC_50_ and average MIC values. The MIC values of the reference strain and the multiresistant clinical isolates were averaged. (**B**) The effect of the *O*-protective groups (benzoyl (Bz) vs. benzyl (Bn) groups) on the IC_50_ values of the **7a** and **3a** complexes. (**C**) Correlation between the apolar character (logD values) and the bioactivity (IC_50_ values) of the complexes. The logD values and the corresponding logIC_50_ values were plotted, and the Pearson correlation was calculated. Color code: khaki—ruthenium complex, blue—osmium complex, green—iridium complex, red—rhodium complex.

In this study, we described a set of half-sandwich type platinum-group metal complexes with *O*-protected *C*-glucosaminyl heterocyclic N,N-bidentate ligands. Among these, the pyridine-containing complexes had the lowest IC_50_ and MIC values, followed by the pyrazine- and pyridazine-containing complexes, while their pyrimidine and quinoline counterparts proved to be inactive (Figure 10A).

Another important structural feature of the complexes is the metal ion with the polyhapto arene/arenyl moiety. In this study, ruthenium complexes with *p*-cymene and iridium complexes with Cp* achieved the best performance in ovarian cancer cells, followed by osmium complexes with *p*-cymene and rhodium complexes with Cp* (Figure 10A). This is at odds with our previous observations, as we identified osmium complexes with the most potent cytostatic properties, followed by ruthenium complexes and, with a large gap, iridium complexes [32,45,46]. Furthermore, rhodium complexes were inactive in cellular models of carcinomas [32,45,46] in contrast to the presently reported results. However, the effect of the metal ions with polyhapto arene/arenyl moiety proved to be similar in terms of the bacteriostatic effects as in our previous experience [31,32], where the ruthenium complexes had the lowest MIC values, followed by osmium and iridium complexes (Figure 10A). Rhodium complexes did not exhibit bacteriostatic properties.

Prior studies have shown that the chemical composition of the protective groups of the sugar part plays a key role in the bioactivity of monosaccharide-containing half-sandwich complexes, showing *O*-benzoyl-protected complexes to be the most effective [32,47]. In this study, besides the *O*-benzoyl-protected *C*-glucosaminyl pyridine **7a** and pyrazine **7d** complexes, their *O*-benzylated counterparts (complexes **3a** and **3d**, respectively) were also tested. Importantly, the benzyl-protected compounds had better IC_50_ values in cancer cell models than the benzoyl-protected compounds (Figure 10B). Interestingly, the bacteriostatic properties of the benzyl/benzoyl-protected compounds did not differ drastically (Figure 9 and Figure 10A). 

Based on the positive logD values (Table 6), all complexes with *O*-benzyl and *O*-benzoyl protective groups are lipophilic. The lipophilic character of the complexes is a prerequisite of their biological activity [31,32,45,46,47,63]. In fact, among the currently identified complexes, the readout of apolarity (logD) and the IC_50_ value correlate (Figure 10C). Apparently, increasing the apolar character of the complexes improves the biological effectiveness, which is further strengthened by the fact that when the protective groups are absent, as in **Ru-5a** or in comparable members of the previously reported series [45,46], the bioactivity of the complexes is lost. 

An unexpected observation was that the *C*-glucosaminyl heterocycles used as ligands, namely pyridines **3a** and **7a**, pyridazine **7b**, pyrimidine **7c**, pyrazines **3d** and **7d** and quinoline **7e**, showed cytostatic effects, which proved comparable to those of the respective complexes in A2780 cells. In addition, **3a** induced cytostasis in primary human fibroblasts. To the best of our knowledge, such effects have not yet been described with *C*-glycosyl heterocycles; therefore, this finding deserves further investigation.

The cytostatic compounds identified in this study were active in other carcinoma cell lines (glioblastoma, breast cancer and pancreatic adenocarcinoma), as well as in another cellular model of ovarian cancer, ID8. Prior studies assessing complexes of similar structure underscore the widespread activity of such complexes, evidencing bioactivity in carcinoma cell lines such as MDA-MD-231 and MCF7 breast cancer cells [45,47], colon cancer [63,64,65,66], lung cancer [63], cervical carcinoma (HeLa) cells [67], U251 glioblastoma cells [45] Capan2 pancreatic adenocarcinoma cells [45,46], L428 Hodgkin lymphoma [32,46] and Saos osteosarcoma [32,46], in addition to ovarian cancer. These cell models include a diverse set of carcinomas, hematological malignancies and a sarcoma.

Importantly, the complexes with a cytostatic property were less active on primary, non-transformed human dermal fibroblasts. While this property of the complexes suggests a selectivity for transformed neoplastic cells over non-transformed cells, the anticipated therapeutic window is relatively narrow as compared to previous observations of complexes with ligands of similar [32,45,46] or different structure [76,77]. 

## 4. Conclusions

In this study, a set of half-sandwich complexes of platinum-group metal ions (Ru(II), Os(II), Ir(III) and Rh(III)) with six-membered *C*-glucosaminyl heterocycles were synthesized. These complexes exerted cytostatic properties against a set of carcinomas with low micromolar IC_50_ values, while they were less active against primary, untransformed human dermal fibroblasts, anticipating a narrow therapeutic window for the compounds. Furthermore, the same complexes had bacteriostatic properties against multiresistant Gram-positive *Staphylococcus aureus* and *Enterococcus* clinical isolates in the low micromolar range. The molecular mechanism of the cytostatic and bacteriostatic properties of the compounds is currently under investigation in our laboratory, and the results will be published in due course.

## 5. Materials and Methods

### 5.1. Synthesis

#### 5.1.1. General Methods

Optical rotations were determined by a P-2000 polarimeter (Jasco, Easton, MD, USA) at room temperature. The ^1^H and ^13^C NMR spectra were recorded with a DRX360 (360/90 MHz for ^1^H/^13^C), DRX400 (400/100 MHz for ^1^H/^13^C) or Bruker Asvance II 500 (500 for ^1^H) spectrometer (Bruker, Karlsruhe, Germany). Chemical shifts are referenced to Me_4_Si (^1^H-NMR) or to the residual solvent signals (^13^C-NMR). The more detailed proton-signal assignments for compounds **3a**, **5a**, **7a**, **Ru-3a**, **Ru-5a**, **Ru-7a**, **Ir-7a**, **Ru-7b** and **Os-7b** are based on COSY correlations. The HRMS data were determined in positive ionization mode using a Bruker maXis II (ESI-HRMS) spectrometer. DC Kieselgel 60 F_254_ plates (Sigma-Aldrich, Saint Louis, MO, USA) were used for TLC analysis, and the spots on the plates were visualized under UV light and developed by gentle heating. For column chromatographic purification, Kieselgel 60 silica gel (Molar Chemicals, Halásztelek, Hungary, particle size 0.063–0.2 mm) was applied. Among anhydrous solvents, EtOH (VWR Chemicals), pyridine (VWR Chemicals) and 1,2-dichloroethane (Sigma-Aldrich) were purchased from the indicated suppliers, while the others were obtained by applying standard distillation methods. Anhydrous CH_2_Cl_2_ was prepared by distillation from P_4_O_10_ and stored over 4 Å molecular sieves, while THF was distilled first from sodium benzophenone ketyl and redistilled from LiAlH_4_ directly before use. 2-Bromopyridine (TCI), 3-bromopyridazine (Fluorochem), 2-iodopyrimidine (Fluorochem), 2-iodopyrazine (Fluorochem), 2-bromoquinoline (TCI), dichloro(η^6^-*p*-cymene)ruthenium(II) dimer (Ru-dimer, Strem Chemicals, Newburyport, MA, USA), dichloro(pentamethylcyclopentadienyl)iridium(III) dimer (**Ir-dimer**, Acros Organics), dichloro(pentamethylcyclopentadienyl)rhodium(III) dimer (**Rh-dimer**, Alfa Aesar) and TlPF_6_ (Strem Chemicals) are commercially available chemicals. Dichloro(η^6^-*p*-cymene)osmium(II) dimer [78] (**Os-dimer**) and 3,4,6-tri-*O*-benzyl-2-nitro-d-glucal [49,79] (**1**) were synthesized by the adaptation of literature procedures.

#### 5.1.2. General Procedure I for the Preparation of *C-*(2′-Deoxy-2′-nitro-3′,4′,6′-tri-*O*-benzyl-β-d-glucopyranosyl)heterocycles **2a–e**

Method A: In a dry, round-bottom flask, the corresponding halogenated heterocycle (4.33 mmol, 2 eq.) was dissolved in freshly distilled dry THF (10 mL). The stirred solution was cooled down to −78 °C, and a 2.5 M solution of *n*-butyllithium in *n*-hexane (1.74 mL, 4.33 mmol, 2 eq.) was added dropwise over 10 min, with stirring continued for 5 min to form the corresponding lithiated heterocycle. In another dry, round-bottom flask containing activated 4 Å molecular sieves (powder, 0.2 g), 2-nitroglucal **1** (1.0 g, 2.17 mmol) was dissolved in freshly distilled dry THF (10 mL). After cooling this solution to −78 °C, the solution of freshly prepared lithiated heterocycle was added. The reaction mixture was then stirred at −78 °C, and the transformation was monitored by TLC (1:4 EtOAc-hexane). When the TLC indicated complete disappearance of **1**, the reaction was quenched by the addition of sat. aq. NH_4_Cl solution (100 mL) and allowed to warm to rt. The molecular sieves were then filtered off. The filtrate was diluted with EtOAc (200 mL) and extracted with water (100 mL) and brine (100 mL). The separated organic phase was dried over MgSO_4_ and filtered, and the solvents were removed under reduced pressure. The residue was purified by column chromatography.

Method B: In a dry, round-bottom flask, 2-nitroglucal **1** (1.0 g, 2.17 mmol) and the corresponding halogenated heterocycle (2.60 mmol, 1.2 eq.) were dissolved in freshly distilled dry THF (20 mL). The stirred solution was cooled down to −78 °C, and a 2.5 M solution of *n*-butyllithium in *n*-hexane (1.04 mL, 2.60 mmol, 1.2 eq.) was added over 15 min by means of a syringe pump. Stirring was continued for an additional 15 min at the same temperature. After completion of the reaction (~0.5 h), as judged by TLC (1:2 EtOAc-hexane), the reaction was quenched by the addition of sat. aq. NH_4_Cl solution (100 mL) and allowed to warm to rt. The mixture was diluted with EtOAc (200 mL) and extracted with water (100 mL) and brine (100 mL). The separated organic phase was dried over MgSO_4_ and filtered, and the solvents were removed under reduced pressure. The residue was purified by column chromatography.

#### 5.1.3. General Procedure II for Cleavage of the *O*-Benzyl Protecting Groups of the *O*-Perbenzylated *C*-Glycopyranosyl Heterocycle **2a–e**, **3a** to obtain compounds **4a–e** and **5a**

A solution of the corresponding *O*-perbenzylated *C*-glycopyranosyl heterocycle **2a–e** or **3a** in dry CH_2_Cl_2_ (10 mL/100 mg substrate) was cooled down to −78 °C, and a 1 M solution of BCl_3_ in CH_2_Cl_2_ (5 eq.) was added dropwise over 5 min. The reaction mixture was stirred at this temperature until the TLC (9:1 CHCl_3_-MeOH) showed the completion of the reaction. Then, the reaction was quenched by the addition of MeOH (10 mL) and allowed to warm to rt. The solvents were then removed under reduced pressure, and the residue was purified by column chromatography.

#### 5.1.4. General Procedure III for the Preparation of *C*-(2′-Deoxy-2′-nitro-3′,4′,6′-tri-*O*-benzoyl-β-d-glucopyranosyl)heterocycles **6a–d**

To a solution of the corresponding *C*-(2′-deoxy-2′-nitro-3′,4′,6′-tri-*O*-benzyl-β-d-glucopyranosyl)heterocycle **2a–d** in dry dichloroethane (10 mL/100 mg substrate), Zn(OTf)_2_ (2 eq.) and benzoyl chloride (6 eq.) were added, and the reaction mixture was stirred at rt. After completion of the reaction, as judged by TLC (1:2 EtOAc-hexane), the reaction mixture was diluted with CH_2_Cl_2_ (40 mL) and extracted with sat. aq. NaHCO_3_ solution (50 mL), then with water (50 mL). The separated organic phase was dried over MgSO_4_ and filtered, and the solvents were removed under diminished pressure. The crude product was purified by column chromatography. 

#### 5.1.5. General Procedure IV for the Preparation of *C*-(2′-(*tert*-Butoxycarbonyl)amino-2′-deoxy-β-d-glucopyranosyl)heterocycles **8c–e**

To a solution of the corresponding *C*-(2′-amino-2′-deoxy-β-d-glucopyranosyl)azine **5c-e** in a 1:1 mixture of water and 1,4-dioxane (5 mL/50 mg substrate), Boc_2_O (2 eq.) was added, and the reaction mixture was stirred at rt. When the TLC (9:1 CHCl_3_-MeOH) showed complete transformation of the staring material (~1 day), the solvents were removed under reduced pressure. The residue was purified by column chromatography.

#### 5.1.6. General Procedure V for the Preparation of *C*-(2′-(*tert*-Butoxycarbonyl)amino-2′-deoxy-3′,4′,6′-tri-*O*-benzoyl-β-d-glucopyranosyl)heterocycles **9c–e**

To a solution of the corresponding *C*-(2′-(*tert*-butoxycarbonyl)amino-2′-deoxy-β-d-glucopyranosyl)azine **8c–e** in dry pyridine (5 mL/100 mg substrate), benzoyl chloride (1.2 eq./OH group) was added at rt. The reaction mixture was stirred at 60 °C for 1 h. Since the TLC (1:1 EtOAc-hexane) showed the incompleteness of the reaction, an additional portion of benzoyl chloride (1.2 eq./OH group) was added to the reaction mixture, and heating was continued for 1 h. The reaction mixture was allowed to cool to rt and further stirred overnight. The reaction mixture was then diluted with CH_2_Cl_2_ (50 mL) and extracted with sat. aq. solution of NaHCO_3_ (25 mL), then with water (25 mL). The separated organic phase was dried over MgSO_4_ and filtered, and the solvents were removed under diminished pressure. The residue was purified by column chromatography.

#### 5.1.7. General Procedure VI for the Preparation of *C*-(2′-Amino-2′-deoxy-3′,4′,6′-tri-*O*-benzoyl-β-d-glucopyranosyl)heterocycles **7b–e** from Compounds **9b–e**

The corresponding *C*-(2′-(*tert*-butoxycarbonyl)amino-2′-deoxy-3′,4′,6′-tri-*O*-benzoyl-β-d-glucopyranosyl)azine **9b–e** was dissolved in dry CH_2_Cl_2_ (5 mL/100 mg substrate), and trifluoroacetic acid (2 eq.) was added. The reaction mixture was stirred at rt until the TLC (95:5 CHCl_3_-MeOH or 1:1 EtOAc-hexane) indicated complete disappearance of the starting material (~1 h). The solvent and the excess CF_3_COOH were then removed under reduced pressure. The residue was dissolved in CH_2_Cl_2_ (50 mL) and extracted with sat. aq. solution of NaHCO_3_ (25 mL) and with water (25 mL). The separated organic phase was dried over MgSO_4_ and filtered, and the solvent was removed under diminished pressure. The residue was purified by column chromatography. 

#### 5.1.8. General Procedure VII for the Synthesis of Half-Sandwich Platinum-Group Metal Complexes

To a solution of the corresponding complex dimer (**Ru-dimer**, **Os-dimer** ([(η^6^-*p*-cym)M^II^Cl_2_]_2_ (M = Ru, Os) or **Ir-dimer**, **Rh-dimer** [(η^5^-Cp*)M^III^Cl_2_]_2_ (M = Ir, Rh)) in CH_2_Cl_2_ (1 mL/10 mg dimer), the appropriate *C*-glucosaminyl azine (1.9–2.3 eq.) and TlPF_6_ (2 eq.) were added. To this stirred reaction mixture, MeOH (1 mL/10 mg dimer) was added at rt in order to accelerate the precipitation of the TlCl. The heterogeneous mixture was then continued further stirred at rt, and the completion of the reaction was monitored by TLC (95:5 CHCl_3_-MeOH). When TLC showed total disappearance of the starting dimer (~1 h), the precipitated TlCl was filtered off. The resulting solution was evaporated under diminished pressure. The remaining crude complex was purified by column chromatography and/or crystallization.

#### 5.1.9. Synthesis and Characterization of the New Compounds

2-(2′-Deoxy-2′-nitro-3′,4′,6′-tri-*O*-benzyl-β-d-glucopyranosyl)pyridine (**2a**) 

Prepared from 2-nitroglucal **1** (0.50 g, 1.08 mmol) and 2-bromopyridine (0.21 mL, 0.34 g, 2.16 mmol, 2 eq.) according to general procedure I, method A. Reaction time: 1 h. Purified by column chromatography (1:4 EtOAc-hexane) to afford 0.30 g (52%) of a colorless syrup. R_f_ = 0.18 (1:4 EtOAc-hexane). ^1^H NMR (400 MHz, CDCl_3_) δ (ppm): 8.57 (1H, ddd, *J* = 4.9, 1.8, 0.9 Hz, H-6), 7.71 (1H, td, *J* = 7.8, 1.8 Hz, H-4), 7.42 (1H, ddd, *J* = 7.8, 1.8, 0.9 Hz, H-3), 7.32–7.18 (16H, m, Ar, H-5), 4.95 (1H, pt, *J* = 9.8, 9.2 Hz, H-2′ or H-3′ or H-4′), 4.91 (1H, d, *J* = 9.7 Hz, H-1′), 4.83, 4.61 (2 × 1H, 2 d, *J* = 10.6 Hz in both, Ph*CH*_2_), 4.81, 4.63 (2 × 1H, 2 d, *J* = 10.9 Hz in both, Ph*CH*_2_), 4.59, 4.53 (2 × 1H, 2 d, *J* = 12.1 Hz in both, Ph*CH*_2_), 4.46 (1H, pt, *J* = 8.6, 8.5 Hz, H-2′ or H-3′ or H-4′), 3.82 (1H, pt, *J* = 9.5, 8.5 Hz, H-2′ or H-3′ or H-4′), 3.81–3.77 (3H, m, H-5′, H-6′a,b); ^13^C NMR (90 MHz, CDCl_3_) δ (ppm): 154.9 (C-2), 149.6 (C-6), 137.1 (C-4), 138.0, 137.7, 137.4, 128.6–127.9 (Ar), 124.1, 122.4 (C-3, C-5), 90.0, 83.2, 80.0, 77.9 (2) (C-1′–C-5′), 75.7, 75.4, 73.7 (3 × Ph*CH*_2_), 68.7 (C-6′). ^1^H and ^13^C NMR data correspond to those reported in [50].

3-(2′-Deoxy-2′-nitro-3′,4′,6′-tri-*O*-benzyl-β-d-glucopyranosyl)pyridazine (**2b**)

Prepared from 2-nitroglucal **1** (2.00 g, 4.33 mmol) and 3-bromopyridazine (0.83 g, 5.20 mmol, 1.2 eq.) according to general procedure I, method B. Purification of the crude product by column chromatography (1:1 EtOAc-hexane) afforded a syrup, which was triturated in a solvent mixture of EtOAc (0.5 mL) and diisopropyl ether (15 mL). The precipitated product was filtered off and washed with diisopropyl ether to afford 0.30 g (13%) of a white, amorphous solid. R_f_ = 0.21 (1:1 EtOAc-hexane). ^1^H NMR (400 MHz, CDCl_3_) δ (ppm): 9.19 (1H, dd, *J* = 5.0, 1.7 Hz, H-6), 7.65 (1H, dd, *J* = 8.5, 1.7 Hz, H-4), 7.53 (1H, dd, *J* = 8.5, 5.0 Hz, H-5), 7.35–7.19 (15H, m, Ar), 5.19 (1H, d, *J* = 10.0 Hz, H-1′), 4.95 (1H, pt, *J* = 10.0, 9.9 Hz, H-2′ or H-3′ or H-4′), 4.85, 4.63 (2 × 1H, 2 d, *J* = 10.8 Hz in both, Ph*CH*_2_), 4.83, 4.65 (2 × 1H, 2 d, *J* = 10.4 Hz in both, Ph*CH*_2_), 4.58, 4.52 (2 × 1H, 2 d, *J* = 12.2 Hz in both, Ph*CH*_2_), 4.51 (1H, pt, *J* = 9.8, 8.3 Hz, H-2′ or H-3′ or H-4′), 3.86 (1H, pt, *J* = 9.4, 8.3 Hz, H-2′ or H-3′ or H-4′), 3.83–3.74 (3H, m, H-5′, H-6′a,b); ^13^C NMR (100 MHz, CDCl_3_) δ (ppm): 158.0 (C-3), 151.9 (C-6), 137.9, 137.6, 137.2, 128.7–127.9 (Ar), 127.3, 125.5 (C-4, C-5), 89.5, 82.8, 80.0, 78.5, 77.6 (C-1′–C-5′), 75.9, 75.4, 73.7 (3 × Ph*CH*_2_), 68.5 (C-6′). ESI-HRMS positive mode (*m*/*z*): calcd for C_31_H_32_N_3_O_6_^+^ [M+H]^+^ 542.2286; C_31_H_31_N_3_O_6_Na^+^ [M+Na]^+^ 564.2105; C_62_H_62_N_6_O_12_Na^+^ [2M+Na]^+^ 1105.4318. Found: [M+H]^+^ 542.2291; [M+Na]^+^ 564.2106; [2M+Na]^+^ 1105.4318.

2-(2′-Deoxy-2′-nitro-3′,4′,6′-tri-*O*-benzyl-β-d-glucopyranosyl)pyrimidine (**2c**)

Prepared from 2-nitroglucal **1** (1.00 g, 2.17 mmol) and 2-iodopyrimidine (0.54 g, 2.60 mmol, 1.2 eq.) according to general procedure I, method B. Purified by column chromatography (1:2 EtOAc-hexane) to afford 0.83 g (71%) of a white, amorphous solid. R_f_ = 0.29 (1:2 EtOAc-hexane). ^1^H NMR (360 MHz, CDCl_3_) δ (ppm): 8.77 (2H, d, *J* = 4.9 Hz, H-4, H-6), 7.35–7.15 (16H, m, Ar, H-5), 5.24 (1H, pt, *J* = 10.1, 10.0 Hz, H-2′ or H-3′ or H-4′), 5.07 (1H, d, *J* = 10.0 Hz, H-1′), 4.82, 4.63 (2 × 1H, 2 d, *J* = 10.6 Hz in both, Ph*CH*_2_), 4.82, 4.58 (2 × 1H, 2 d, *J* = 10.6 Hz in both, Ph*CH*_2_), 4.58,4.49 (2 × 1H, 2 d, *J* = 12.2 Hz in both, Ph*CH*_2_), 4.46 (1H, pt, *J* = 9.9, 9.5 Hz, H-2′ or H-3′ or H-4′), 3.86 (1H, pt, *J* = 9.9, 8.5 Hz, H-2′ or H-3′ or H-4′), 3.84–3.74 (3H, m, H-5′, H-6′a,b); ^13^C NMR (100 MHz, CDCl_3_) δ (ppm): 163.6 (C-2), 157.8 (C-4, C-6), 137.8, 137.6, 137.3, 128.7–127.9 (Ar), 121.3 (C-5), 88.4, 83.0, 80.6, 80.2, 77.7 (C-1′–C-5′), 75.7, 75.3, 73.6 (3 × Ph*CH*_2_), 68.4 (C-6′). ESI-HRMS positive mode (*m*/*z*): calcd for C_31_H_32_N_3_O_6_^+^ [M+H]^+^ 542.2286; C_31_H_31_N_3_O_6_Na^+^ [M+Na]^+^ 562.2105. Found: [M+H]^+^ 542.2288; [M+Na]^+^ 562.2105.

2-(2′-Deoxy-2′-nitro-3′,4′,6′-tri-*O*-benzyl-β-d-glucopyranosyl)pyrazine (**2d**)

Prepared from 2-nitroglucal **1** (1.00 g, 2.17 mmol) and 2-iodopyrazine (0.54 g, 2.60 mmol, 1.2 eq.) according to general procedure I, method B. Purified by column chromatography (1:3 EtOAc-hexane) to afford 0.74 g (63%) of a white, amorphous solid. R_f_ = 0.54 (1:2 EtOAc-hexane). ^1^H NMR (400 MHz, CDCl_3_) δ (ppm): 8.75 (1H, d, *J* = 1.4 Hz, H-3), 8.58 (1H, d, *J* = 2.5 Hz, H-6), 8.52 (1H, dd, *J* = 2.5, 1.4 Hz, H-5), 7.35–7.19 (15H, m, Ar) 5.00 (1H, d, *J* = 9.9 Hz, H-1′), 4.94 (1H, pt, *J* = 9.9, 9.7 Hz, H-2′ or H-3′ or H-4′), 4.84, 4.62 (2 × 1H, 2 d, *J* = 10.8 Hz in both, Ph*CH*_2_), 4.82, 4.63 (2 × 1H, 2 d, *J* = 10.6 Hz in both, Ph*CH*_2_), 4.59, 4.53 (2 × 1H, 2 d, *J* = 12.1 Hz in both, Ph*CH*_2_), 4.45 (1H, pt, *J* = 8.7, 8.7 Hz, H-2′ or H-3′ or H-4′), 3.83 (1H, pt, *J* = 9.4, 8.7 Hz, H-2′ or H-3′ or H-4′), 3.82–3.76 (3H, m, H-5′, H-6′a,b); ^13^C NMR (100 MHz, CDCl_3_) δ (ppm): 150.7 (C-2), 145.3, 144.2, 143.9 (C-3, C-5, C-6), 137.8, 137.6, 137.2, 128.7–127.9 (Ar), 89.2, 83.0, 80.1, 77.8, 77.6 (C-1′–C-5′), 75.9, 75.4, 73.7 (3 × Ph*CH*_2_), 68.5 (C-6′). ESI-HRMS positive mode (*m*/*z*): calcd for C_31_H_31_N_3_O_6_Na^+^ [M+Na]^+^ 564.2105. Found: 564.2107.

2-(2′-Deoxy-2′-nitro-3′,4′,6′-tri-*O*-benzyl-β-d-glucopyranosyl)quinoline (**2e**)

Prepared from 2-nitroglucal **1** (2.00 g, 4.33 mmol) and 2-bromoquinoline (1.83 g, 8.80 mmol, 2 eq.) according to general procedure I, method A. Reaction time: 2 h. Purified by column chromatography (1:9 EtOAc-hexane) to afford 1.53 g (60%) of a colorless syrup. R_f_ = 0.35 (1:4 EtOAc-hexane). ^1^H NMR (400 MHz, CDCl_3_) δ (ppm): 8.18 (1H, d, *J* = 8.5 Hz, H-3 or H-4), 8.04 (1H, dd, *J* = 8.5, 1.0 Hz, H-5 or H-8), 7.79 (1H, dd, *J* = 8.2, 1.4 Hz, H-5 or H-8), 7.69 (1H, ddd, *J* = 8.5, 7.0, 1.4 Hz, H-6 or H-7), 7.57 (1H, d, *J* = 8.5 Hz, H-3 or H-4), 7.53 (1H, ddd, *J* = 8.2, 7.0, 1.0 Hz, H-6 or H-7), 7.35–7.20 (15H, m, Ar), 5.13 (1H, d, *J* = 9.7 Hz, H-1′), 5.10 (1H, pt, *J* = 9.8, 8.0 Hz, H-2′ or H-3′ or H-4′), 4.86, 4.65 (2 × 1H, 2 d, *J* = 10.9 Hz in both, Ph*CH*_2_), 4.84, 4.64 (2 × 1H, 2 d, *J* = 10.6 Hz in both, Ph*CH*_2_), 4.61, 4.53 (2 × 1H, 2 d, *J* = 12.2 Hz in both, Ph*CH*_2_), 4.50 (1H, pt, *J* = 8.5, 8.4 Hz, H-2′ or H-3′ or H-4′), 4.88 (1H, pt, *J* = 9.5, 8.4 Hz, H-2′ or H-3′ or H-4′), 3.86–3.80 (3H, m, H-5′, H-6′a,b); ^13^C NMR (100 MHz, CDCl_3_) δ (ppm): 155.0, 147.3 (C-2, C-8a), 138.0, 137.7, 137.3, 137.4, 130.0, 129.9, 128.7–127.8, 127.6, 127.2, 119.4 (Ar, C-3–C-8, C-4a), 89.3, 83.3, 80.1, 80.0, 77.8 (C-1′–C-5′), 75.8, 75.4, 73.7 (3 × Ph*CH*_2_), 68.6 (C-6′). ESI-HRMS positive mode (*m*/*z*): calcd for C_36_H_34_N_2_O_6_Na^+^ [M+Na]^+^ 613.2309. Found: 613.2309.

2-(2′-Amino-2′-deoxy-3′,4′,6′-tri-*O*-benzyl-β-d-glucopyranosyl)pyridine (**3a**)

Compound **2a** (0.19 g, 0.35 mmol) and Zn powder (0.69 g, 10.55 mmol, 30 eq.) were suspended in a solvent mixture of THF (10 mL) and water (5 mL). This suspension was cooled down in an ice bath, and *cc*HCl solution was added (0.7 mL, 8.14 mmol, 23 eq.). The reaction mixture was stirred at rt until the TLC (1:1 EtOAc-hexane) showed total consumption of the starting material (1 h). The reaction was quenched by the addition of sat. aq. NaHCO_3_ solution (50 mL). The insoluble inorganic salts and the rest of the Zn were filtered off, and the remaining solution was extracted with CH_2_Cl_2_ (2 × 50 mL). The combined organic phase was extracted with water (50 mL), then with brine (50 mL), dried over MgSO_4_ and filtered. The solvent was removed under reduced pressure. The residue was purified by column chromatography (95:5 CHCl_3_-MeOH) to afford114 mg (64%) of a pale yellow amorphous solid. R_f_ = 0.34 (95:5 CHCl_3_-MeOH); [α]_D_ = +30 (c 0.5, CHCl_3_). ^1^H NMR (400 MHz, CDCl_3_) δ (ppm): 8.56 (1H, ddd, *J* = 4.9, 1.8, 1.0 Hz, H-6), 7.70 (1H, td, *J* = 7.7, 1.8 Hz, H-4), 7.47 (1H, ddd, *J* = 7.7, 1.8, 1.0 Hz, H-3), 7.37–7.20 (16H, m, Ar, H-5), 5.01, 4.80 (2 × 1H, 2 d, *J* = 11.4 Hz in both, Ph*CH*_2_), 4.84, 4.63 (2 × 1H, 2 d, *J* = 10.7 Hz in both, Ph*CH*_2_), 4.62, 4.56 (2 × 1H, 2 d, *J* = 12.2 Hz in both, Ph*CH*_2_), 4.27 (1H, d, *J* = 9.6 Hz, H-1′), 3.80–3.75 (3H, m, H-4′, H-6′a,b), 3.70 (1H, m, H-5′), 3.62 (1H, pt, *J* = 9.2, 9.2 Hz, H-3′), 3.22 (1H, pt, *J* = 9.7, 9.6 Hz, H-2′), 1.65 (2H, s, NH_2_); ^13^C NMR (90 MHz, CDCl_3_) δ (ppm): 158.7 (C-2), 148.9 (C-6), 138.7, 138.3, 138.2 (Ar), 137.0 (C-4), 128.6–127.7 (Ar), 123.2, 122.7 (C-3, C-5), 87.0, 83.0, 79.8, 78.9 (C-1′, C-3′–C-5′), 75.5, 75.0, 73.6 (3 × Ph*CH*_2_), 69.4 (C-6′), 57.2 (C-2′). ESI-HRMS positive mode (*m*/*z*): calcd for C_32_H_34_N_2_O_4_Na^+^ [M+Na]^+^ 533.2410. Found: 533.2411.

2-(2′-Amino-2′-deoxy-3′,4′,6′-tri-*O*-benzyl-β-d-glucopyranosyl)pyrazine (**3d**)

Compound **2d** (0.10 g, 0.19 mmol) and Zn powder (0.12 g, 1.84 mmol, 10 eq.) were suspended in a solvent mixture of THF (5 mL) and water (2.5 mL). To this stirred mixture, a 1 M aq. solution of HCl (1.5 mL, 1.50 mmol, 8 eq.) was added dropwise over 1 h using a syringe pump. The reaction mixture was further stirred at rt until TLC (95:5 CHCl_3_-MeOH) indicated the completion of the reaction (2 h). The reaction was quenched by the addition of sat. aq. NaHCO_3_ solution (25 mL). The insoluble inorganic salts and the rest of the Zn were filtered off, and the remaining solution was extracted with CH_2_Cl_2_ (2 × 25 mL). The combined organic phase was extracted with water (25 mL), dried over MgSO_4_ and filtered. The solvent was removed under reduced pressure. Column chromatographic purification of the residue (95:5 CHCl_3_-MeOH) afforded 6.8 mg (7%) of a pale yellow syrup. R_f_ = 0.23 (95:5 CHCl_3_-MeOH); [α]_D_ = +19 (c 0.1, CHCl_3_). ^1^H NMR (400 MHz, CDCl_3_) δ (ppm): 8.77 (1H, d, *J* = 1.0 Hz, H-3), 8.53–8.52 (2H, m, H-5, H-6), 7.35–7.21 (15H, m, Ar), 5.03, 4.78 (2 × 1H, 2 d, *J* = 11.4 Hz in both, Ph*CH*_2_), 4.85, 4.64 (2 × 1H, 2 d, *J* = 10.8 Hz in both, Ph*CH*_2_), 4.61, 4.56 (2 × 1H, 2 d, *J* = 12.3 Hz in both, Ph*CH*_2_), 4.31 (1H, d, *J* = 9.7 Hz, H-1′), 3.81–3.73 (2H, m, H-6′a,b), 3.77 (1H, pt, *J* = 9.8, 8.5 Hz, H-3′ or H-4′), 3.71 (1H, m, H-5′), 3.60 (1H, pt, *J* = 9.2, 9.0 Hz, H-3′ or H-4′), 3.25 (1H, pt, *J* = 9.7, 9.6 Hz, H-2′), 1.68 (2H, br s, NH_2_); ^13^C NMR (100 MHz, CDCl_3_) δ (ppm): 154.2 (C-2), 145.0, 144.3, 143.5 (C-3, C-5, C-6), 138.6, 138.2, 138.1, 129.9–127.8 (Ar), 86.9, 81.4, 80.0, 78.8 (C-1′, C-3′–C-5′), 75.6, 75.0, 73.7 (3 × Ph*CH*_2_), 69.2 (C-6′), 56.8 (C-2′). ESI-HRMS positive mode (*m*/*z*): calcd for C_31_H_34_N_3_O_4_^+^ [M+H]^+^ 512.2544; C_31_H_33_N_3_O_4_Na^+^ [M+Na]^+^ 534.2363. Found: [M+H]^+^ 512.2541, [M+Na]^+^ 534.2359. 

2-(2′-Deoxy-2′-nitro-β-d-glucopyranosyl)pyridine (**4a**)

Prepared from compound **2a** (0.30 g, 0.55 mmol) according to general procedure II. Reaction time: 0.5 h. Purification by column chromatography (9:1 CHCl_3_-MeOH) yielded 128 mg (85%) of a white, amorphous solid. R_f_ = 0.53 (4:1 CHCl_3_-MeOH). ^1^H NMR (400 MHz, CD_3_OD) δ (ppm): 8.53 (1H, d, *J* = 4.4 Hz, H-6), 7.85 (1H, t, *J* = 7.7 Hz, H-4), 7.54 (1H, d, *J* = 7.8 Hz, H-3), 7.40 (1H, m, H-5), 4.95 (1H, d, *J* = 9.7 Hz, H-1′), 4.74 (1H, pt, *J* = 10.1, 9.9 Hz, H-2′ or H-3′ or H-4′), 4.21 (1H, pt, *J* = 9.1, 8.7 Hz, H-2′ or H-3′ or H-4′), 3.91 (1H, m, H-6′a), 3.76 (1H, dd, *J* = 12.0, 4.2 Hz, H-6′b), 3.62–3.55 (2H, m, H-2′ or H-3′ or H-4′, H-5′); ^13^C NMR (90 MHz, CD_3_OD) δ (ppm): 156.4 (C-2), 150.2 (C-6), 138.9 (C-4), 125.6, 124.4 (C-3, C-5), 92.7, 82.7, 80.7, 76.4, 71.1 (C-1′–C-5′), 62.4 (C-6′). ESI-HRMS positive mode (*m*/*z*): calcd for C_11_H_14_N_2_O_6_Na^+^ [M+Na]^+^ 293.0744. Found: 293.0744. 

3-(2′-Deoxy-2′-nitro-β-d-glucopyranosyl)pyridazine (**4b**)

Prepared from compound **2b** (0.30 g, 0.55 mmol) according to general procedure II. Reaction time: 0.5 h. Purification by column chromatography (9:1 CHCl_3_-MeOH) yielded 133 mg (89%) of a white, amorphous solid. R_f_ = 0.39 (4:1 CHCl_3_-MeOH). ^1^H NMR (360 MHz, CD_3_OD) δ (ppm): 9.17 (1H, d, *J* = 4.9 Hz, H-6), 8.00 (1H, dd, *J* = 8.6, 1.4 Hz, H-4), 7.79 (1H, dd, *J* = 8.6, 4.9 Hz, H-5), 5.22 (1H, d, *J* = 10.0 Hz, H-1′), 4.82 (1H, pt, *J* = 10.1, 10.0 Hz, H-2′ or H-3′ or H-4′), 4.25 (1H, pt, *J* = 10.0, 9.9 Hz, H-2′ or H-3′ or H-4′), 3.94 (1H, dd, *J* = 12.2, 2.1 Hz, H-6′a), 3.77 (1H, dd, *J* = 12.2, 5.3 Hz, H-6′b), 3.67 (1H, ddd, *J* = 9.8, 5.3, 2.1 Hz, H-5′), 3.57 (1H, pt, *J* = 9.8, 9.5 Hz, H-2′ or H-3′ or H-4′); ^13^C NMR (90 MHz, CD_3_OD) δ (ppm): 160.3 (C-3), 153.0 (C-6), 129.7, 128.2 (C-4, C-5), 91.9, 82.9, 79.2, 76.3, 71.0 (C-1′–C-5′), 62.4 (C-6′). ESI-HRMS positive mode (*m*/*z*): calcd for C_10_H_13_N_3_O_6_Na^+^ [M+Na]^+^ 294.0697. Found: 294.0698.

2-(2′-Deoxy-2′-nitro-β-d-glucopyranosyl)pyrimidine (**4c**)

Prepared from compound **2c** (0.10 g, 0.18 mmol) according to general procedure II. Reaction time: 0.5 h. Purification by column chromatography (9:1 CHCl_3_-MeOH) yielded 49 mg (98%) of a white, amorphous solid. R_f_ = 0.52 (2:1 CHCl_3_-MeOH). ^1^H NMR (360 MHz, CD_3_OD) δ (ppm): 8.99 (2H, d, *J* = 5.0 Hz, H-4, H-6), 7.70 (1H, t, *J* = 5.0 Hz, H-5), 5.24 (1H, d, *J* = 10.1 Hz, H-1′), 4.85 (1H, pt, *J* = 10.1, 10.0 Hz, H-2′ or H-3′ or H-4′), 4.20 (1H, pt, *J* = 10.0, 9.9 Hz, H-2′ or H-3′ or H-4′), 3.96 (1H, dd, *J* = 12.2, 2.1 Hz, H-6′a), 3.76 (1H, dd, *J* = 12.2, 5.5 Hz, H-6′b), 3.68 (1H, ddd, *J* = 9.5, 5.5, 2.1 Hz, H-5′), 3.52 (1H, pt, *J* = 9.9, 9.5 Hz, H-2′ or H-3′ or H-4′); ^13^C NMR (90 MHz, CD_3_OD) δ (ppm): 165.2 (C-2), 159.0 (C-4, C-6), 122.8 (C-5), 91.2, 82.9, 80.8, 76.3, 71.0 (C-1′–C-5′), 62.4 (C-6′). ESI-HRMS positive mode (*m*/*z*): calcd for C_10_H_13_N_3_O_6_Na^+^ [M+Na]^+^ 294.0697. Found: 294.0698.

2-(2′-Deoxy-2′-nitro-β-d-glucopyranosyl)pyrazine(**4d**)

Prepared from compound **2d** (0.30 g, 0.55 mmol) according to general procedure II. Reaction time: 0.5 h. Purification by column chromatography (9:1 CHCl_3_-MeOH) yielded 117 mg (78%) of a white, amorphous solid. R_f_ = 0.48 (4:1 CHCl_3_-MeOH). ^1^H NMR (400 MHz, CD_3_OD) δ (ppm): 8.83 (1H, d, *J* = 1.5 Hz, H-3), 8.60 (1H, d, *J* = 2.6 Hz, H-6), 8.57 (1H, dd, *J* = 2.6, 1.5 Hz, H-5), 5.10 (1H, d, *J* = 9.9 Hz, H-1′), 4.81 (1H, pt, *J* = 10.0, 10.0 Hz, H-2′ or H-3′ or H-4′), 4.21 (1H, pt, *J* = 10.0, 9.7 Hz, H-2′ or H-3′ or H-4′), 3.93 (1H, dd, *J* = 12.2, 2.1 Hz, H-6′a), 3.76 (1H, dd, *J* = 12.2, 5.4 Hz, H-6′b), 3.64 (1H, ddd, *J* = 10.0, 5.4, 2.1 Hz, H-5′), 3.54 (1H, pt, *J* = 9.4, 9.3 Hz, H-2′ or H-3′ or H-4′); ^13^C NMR (90 MHz, CD_3_OD) δ (ppm): 152.9 (C-2), 146.2, 145.5, 145.2 (C-3, C-5, C-6), 91.6, 82.9, 78.6, 76.5, 71.1 (C-1′–C-5′), 62.4 (C-6′). ESI-HRMS positive mode (*m*/*z*): calcd for C_10_H_13_N_3_O_6_Na^+^ [M+Na]^+^ 294.0697. Found: 294.0698.

2-(2′-Deoxy-2′-nitro-β-d-glucopyranosyl)quinoline (**4e**)

Prepared from compound **2e** (83 mg, 0.14 mmol) according to general procedure II. Reaction time: 1 h. Purification by column chromatography (9:1 CHCl_3_-MeOH) yielded 31 mg (76%) of a white, amorphous solid. R_f_ = 0.19 (9:1 CHCl_3_-MeOH). ^1^H NMR (400 MHz, CD_3_OD) δ (ppm): 8.36, 7.70 (2 × 1H, 2 d, *J* = 8.5 Hz in both, H-3, H-4), 8.00, 7.93 (2 ×1H, 2 d, *J* = 7.9 Hz in both, H-5, H-8), 7.77, 7.61 (2 × 1H, 2 t, *J* = 7.9 Hz in both, H-6, H-7), 5.14 (1H, d, *J* = 9.9 Hz, H-1′), 4.90 (1H, pt, *J* = 10.1, 10.0 Hz, H-2′ or H-3′ or H-4′), 4.27 (1H, pt, *J* = 9.7, 9.0 Hz, H-2′ or H-3′ or H-4′), 3.96 (1H, dd, *J* = 12.2, < 1Hz, H-6′a), 3.80 (1H, dd, *J* = 12.2, 4.7 Hz, H-6′b), 3.67 (1H, m, H-5′), 3.61 (1H, pt, *J* = 9.2, 9.1 Hz, H-2′ or H-3′ or H-4′); ^13^C NMR (90 MHz, CD_3_OD) δ (ppm): 157.2, 148.3 (C-2, C-8a), 138.9, 131.2, 129.7, 129.0, 128.4, 121.2 (C-3–C-8), 129.5 (C-4a), 92.3, 82.9, 81.1, 76.6, 71.2 (C-1′–C-5′), 62.5 (C-6′). ESI-HRMS positive mode (*m*/*z*): calcd for C_15_H_16_N_2_O_6_Na^+^ [M+Na]^+^ 343.0901. Found: 343.0900.

2-(2′-Amino-2′-deoxy-β-d-glucopyranosyl)pyridine (**5a**)

Method A: Prepared from compound **3a** (65 mg, 0.13 mmol) according to general procedure II. Reaction time: 0.5 h. Purification by column chromatography (7:3 CHCl_3_-MeOH) yielded 15 mg (42%) of a white, amorphous solid.

Method B: Compound **3a** (0.22 g, 0.43 mmol) and pentamethylbenzene (0.58 g, 3.88 mmol, 9 eq.) were dissolved in dry CH_2_Cl_2_ (22 mL), and the solution was cooled down to −78 °C. To this solution, a 1 M solution of BCl_3_ in CH_2_Cl_2_ (1.72 mL, 1.72 mmol, 4 eq.) was added dropwise over 5 min. The reaction mixture was stirred at this temperature until the TLC (9:1 CHCl_3_-MeOH) showed the completion of the reaction (0.5 h). Then, the reaction was quenched by the addition of MeOH (10 mL) and allowed to warm to rt. The solvents were then removed under reduced pressure. Purification of the residue by column chromatography (7:3 CHCl_3_-MeOH) yielded 113 mg (95%) of a white, amorphous solid. R_f_ = 0.23 (7:3 CHCl_3_-MeOH); [α]_D_ = +84 (c 0.5, MeOH). ^1^H NMR (400 MHz, CD_3_OD) δ (ppm): 8.57 (1H, d, *J* = 4.5 Hz, H-6), 7.90 (1H, td, *J* = 7.8, 1.7 Hz, H-4), 7.72 (1H, d, *J* = 7.8 Hz, H-3), 7.41 (1H, dd, *J* = 7.5, 5.0 Hz, H-5), 4.57 (1H, d, *J* = 10.0 Hz, H-1′), 3.94 (1H, dd, *J* = 12.1, 1.8 Hz, H-6′a), 3.76 (1H, dd, *J* = 12.1, 4.9 Hz, H-6′b), 3.66 (1H, pt, *J* = 9.0, 8.9 Hz, H-3′), 3.54–3.45 (2H, m, H-4′, H-5′), 3.21 (1H, pt, *J* = 10.0, 9.9 Hz, H-2′); ^13^C NMR (90 MHz, CD_3_OD) δ (ppm): 158.7 (C-2), 149.6 (C-6), 139.1 (C-4), 125.1, 123.9 (C-3, C-5), 82.5, 78.7, 76.4, 71.6 (C-1′, C-3′–C-5′), 62.7 (C-6′), 57.6 (C-2′). ESI-HRMS positive mode (*m*/*z*): calcd for C_11_H_17_N_2_O_4_^+^ [M+H]^+^ 241.1183; C_11_H_16_N_2_O_4_Na^+^ [M+Na]^+^ 263.1001. Found: [M +H]^+^ 241.1183; [M+Na]^+^ 263.1002.

2-(2′-Amino-2′-deoxy-β-d-glucopyranosyl)pyrimidine (**5c**)

A degassed, vigorously stirred suspension of 10% Pd(C) (56 mg) in dry EtOH (11 mL) was saturated with H_2_, and compound **4c** (0.11 g, 0.41 mmol) was added. The reaction mixture was heated at reflux temperature until the TLC (3:2 CHCl_3_-MeOH) indicated complete conversion of the starting material. After completion of the reaction (2 h), the catalyst was filtered off through a pad of celite and washed with MeOH. The resulting solution was then evaporated under reduced pressure. Purification of the remaining crude product by column chromatography (3:2 CHCl_3_-MeOH) yielded 37 mg (38%) of a white, amorphous solid. R_f_ = 0.10 (3:2 CHCl_3_-MeOH); [α]_D_ = +22 (c 0.1, MeOH). ^1^H NMR (400 MHz, CD_3_OD) δ (ppm): 8.85 (2H, d, *J* = 4.9 Hz, H-4, H-6), 7.48 (1H, t, *J* = 4.9 Hz, H-5), 4.42 (1H, d, *J* = 9.7 Hz, H-1′), 3.87 (1H, dd, *J* = 12.1, 1.5 Hz, H-6′a), 3.73 (1H, dd, *J* = 12.1, 4.5 Hz, H-6′b), 3.50 (1H, pt, *J* = 9.5, 9.0 Hz, H-3′ or H-4′), 3.50–3.45 (1H, m, H-5′), 3.44 (1H, pt, *J* = 9.2, 9.0 Hz, H-3′ or H-4′), 3.08 (1H, pt, *J* = 9.5, 9.4 Hz, H-2′); ^13^C NMR (90 MHz, CD_3_OD) δ (ppm): 167.7 (C-2), 158.8 (C-4, C-6), 122.2 (C-5), 83.8, 82.6, 79.3, 71.4 (C-1′, C-3′–C-5′), 62.8 (C-6′), 57.6 (C-2′). ESI-HRMS positive mode (*m*/*z*): calcd for C_10_H_15_N_3_O_4_Na^+^ [M+Na]^+^ 264.0955. Found: 264.0957.

2-(2′-Amino-2′-deoxy-β-d-glucopyranosyl)pyrazine (**5d**)

A degassed, vigorously stirred suspension of 10% Pd(C) (60 mg) in dry EtOH (12 mL) was saturated with H_2_, and compound **4d** (0.12 g, 0.43 mmol) was added. The reaction mixture was heated at reflux temperature until the TLC (3:2 CHCl_3_-MeOH) indicated complete conversion of the starting material. After completion of the reaction (6 h), the catalyst was filtered off through a pad of celite and washed with MeOH. The resulting solution was then evaporated under reduced pressure. Purification of the remaining crude product by column chromatography (7:3 CHCl_3_-MeOH) yielded 68 mg (66%) of a white, amorphous solid. R_f_ = 0.15 (3:2 CHCl_3_-MeOH); [α]_D_ = +41 (c 0.5, MeOH). ^1^H NMR (400 MHz, CD_3_OD) δ (ppm): 8.79 (1H, d, *J* = 1.3 Hz, H-3), 8.62 (1H, dd, *J* = 2.4, 1.3 Hz, H-5), 8.57 (1H, d, *J* = 2.4 Hz, H-6), 4.38 (1H, d, *J* = 9.7 Hz, H-1′), 3.90 (1H, dd, *J* = 12.1, 1.7 Hz, H-6′a), 3.73 (1H, dd, *J* = 12.1, 5.0 Hz, H-6′b), 3.51–3.40 (3H, m, H-3′, H-4′, H-5′), 3.00 (1H, pt, *J* = 9.5, 9.4 Hz, H-2′); ^13^C NMR (90 MHz, CD_3_OD) δ (ppm): 155.4 (C-2), 146.0, 145.4, 145.1 (C-3, C-5, C-6), 82.6, 81.4, 79.2, 71.6 (C-1′, C-3′–C-5′), 62.8 (C-6′), 58.0 (C-2′). ESI-HRMS positive mode (*m*/*z*): calcd for C_10_H_16_N_3_O_4_Na^+^ [M+H]^+^ 242.1135. Found: 242.1133.

2-(2′-Amino-2′-deoxy-β-d-glucopyranosyl)quinoline (**5e**)

Compound **4e** (0.10 g, 0.33 mmol) and tin powder (1.17 g, 9.82 mmol, 30 eq.) were suspended in a solvent mixture of THF (5 mL) and water (2.5 mL). This heterogenous mixture was cooled down in an ice bath, and *cc*HCl solution (0.85 mL, 9.88 mmol 30 eq.) was added. The reaction mixture was then stirred at rt. When the TLC (4:1 CHCl_3_-MeOH) showed complete conversion of **4e** (1 d), a 2 M aq. solution of NaOH was added to the reaction mixture to obtain a slightly basic solution, which was then neutralized by the addition of sat. aq. NH_4_Cl solution. The solvents were removed under diminished pressure. The residue was treated with MeOH (20 mL), and the inseparable inorganic salts and the excess of the unreacted Sn were filtered off. The resulting solution was evaporated in vacuo. Column chromatographic purification of the residue (9:1 CHCl_3_-MeOH) yielded 28 mg (29%) of a pale yellow, amorphous solid. R_f_ = 0.11 (4:1 CHCl_3_-MeOH); [α]_D_ = −11 (c 0.1, MeOH). ^1^H NMR (400 MHz, CD_3_OD) δ (ppm): 8.34 (1H, d, *J* = 8.5 Hz, H-4), 8.06 (1H, dd, *J* = 8.5, 1.2 Hz, H-8), 7.92 (1H, dd, *J* = 8.2, 1.4 Hz, H-5), 7.76 (1H, ddd, *J* = 8.5, 6.8, 1.4 Hz, H-7), 7.71 (1H, d, *J* = 8.5 Hz, H-3), 7.59 (1H, ddd, *J* = 8.2, 6.8, 1.2 Hz, H-6), 4.50 (1H, d, *J* = 9.7 Hz, H-1′), 3.94 (1H, dd, *J* = 12.2, 1.3 Hz, H-6′a), 3.79 (1H, ddd, *J* = 12.2, 3.2, 1.3 Hz, H-5′), 3.56–3.50 (3H, m, H-3′, H-4′, H-6′b), 3.12 (1H, pt, *J* = 9.5, 9.4 Hz, H-2′); ^13^C NMR (90 MHz, CD_3_OD) δ (ppm): 160.1, 148.3 (C-2, C-8a), 138.7, 131.0, 129.6, 129.0, 128.0, 121.7 (C-3–C-8), 129.4 (C-4a), 83.3, 82.6, 79.1, 71.7 (C-1′, C-3′–C-5′), 62.9 (C-6′) 58.3(C-2′). ESI-HRMS positive mode (*m*/*z*): calcd for C_15_H_18_N_2_O_4_Na^+^ [M+Na]^+^ 313.1159; C_30_H_36_N_4_O_8_Na^+^ [2M+Na]^+^ 603.2425. Found: [M+Na]^+^ 313.1158; [2M+Na]^+^ 603.2425.

2-(2′-Deoxy-2′-nitro-3′,4′,6′-tri-*O*-benzoyl-β-d-glucopyranosyl)pyridine (**6a**)

Prepared from compound **2a** (95 mg, 0.18 mmol) and benzoyl chloride (0.13 mL, 1.12 mmol, 6 eq.) according to general procedure III. Reaction time: 5 d. Purified by column chromatography (1:4 EtOAc-hexane) yielded 90 mg (88%) of a white, amorphous solid. R_f_ = 0.38 (1:2 EtOAc-hexane). ^1^H NMR (400 MHz, CDCl_3_) δ (ppm): 8.60 (1H, ddd, *J* = 4.9, 1.5, 0.9 Hz, H-6), 8.14–7.29 (18H, m, Ar, H-3, H-4, H-5), 6.39 (1H, pt, *J* = 10.0, 9.7 Hz, H-2′ or H-3′ or H-4′), 5.76 (1H, pt, *J* = 9.8, 9.8 Hz, H-2′ or H-3′ or H-4′), 5.46 (1H, pt, *J* = 10.1, 10.1 Hz, H-2′ or H-3′ or H-4′), 5.28 (1H, d, *J* = 9.9 Hz, H-1′), 4.66 (1H, dd, *J* = 12.4, 3.1 Hz, H-6′a), 4.53 (1H, dd, *J* = 12.4, 5.3 Hz, H-6′b), 4.41 (1H, ddd, *J* = 10.1, 5.3, 3.1 Hz, H-5′); ^13^C NMR (100 MHz, CDCl_3_) δ (ppm): 166.2, 165.4, 165.2 (3 × C=O), 153.8 (C-2), 149.7 (C-6), 137.4 (C-4), 133.8 (2), 133.3, 130.1–128.4 (Ar), 124.6, 123.1 (C-3, C-5), 87.2, 79.5, 76.9, 73.3, 69.4 (C-1′–C-5′), 63.3 (C-6′). ESI-HRMS positive mode (*m*/*z*): calcd for C_32_H_27_N_2_O_9_^+^ [M+H]^+^ 583.1711; C_32_H_26_N_2_O_9_Na^+^ [M+Na]^+^ 605.1531. Found: [M+H]^+^ 583.1713; [M+Na]^+^ 605.1532.

3-(2′-Deoxy-2′-nitro-3′,4′,6′-tri-*O*-benzoyl-β-d-glucopyranosyl)pyridazine (**6b**)

Prepared from compound **2b** (50 mg, 0.092 mmol) and benzoyl chloride (64 μL, 0.55 mmol, 6 eq.) according to general procedure III. Reaction time: 10 d. Purification by column chromatography (1:1 EtOAc-hexane) yielded 52 mg (97%) of a white, amorphous solid. R_f_ = 0.32 (1:1 EtOAc-hexane). ^1^H NMR (400 MHz, CDCl_3_) δ (ppm): 9.21 (1H, d, *J* = 5.0 Hz, H-6), 8.01–7.33 (17H, m, Ar, H-4, H-5), 6.45 (1H, pt, *J* = 9.8, 9.2 Hz, H-2′ or H-3′ or H-4′), 5.79 (1H, pt, *J* = 9.5, 9.5 Hz, H-2′ or H-3′ or H-4′), 5.60–5.49 (2H, m, H-1′, H-2′ or H-3′ or H-4′), 4.73–4.43 (3H, m, H-5′, H-6′a,b); ^13^C NMR (100 MHz, CDCl_3_) δ (ppm): 166.2, 165.3, 165.2 (3 × C=O), 157.0 (C-3), 152.1 (C-6), 133.9, 133.4, 130.1–129.8, 129.4, 128.6–128.5, 128.4, 128.2 (Ar), 127.5, 126.2 (C-4, C-5), 86.4, 78.0, 77.0, 73.0, 69.0 (C-1′–C-5′), 62.9 (C-6′). ESI-HRMS positive mode (*m*/*z*): calcd for C_31_H_25_N_3_O_9_Na^+^ [M+Na]^+^ 606.1483. Found: 606.1479.

2-(2′-Deoxy-2′-nitro-3′,4′,6′-tri-*O*-benzoyl-β-d-glucopyranosyl)pyrimidine (**6c**)

Prepared from compound **2c** (0.50 g, 0.92 mmol) and benzoyl chloride (0.65 mL, 5.60 mmol, 6 eq.) according to general procedure III. Reaction time: 30 d. Purification by column chromatography (1:2 EtOAc-hexane) yielded 0.24 g (45%) of a white, amorphous solid. R_f_ = 0.34 (1:2 EtOAc-hexane). ^1^H NMR (360 MHz, CDCl_3_) δ (ppm): 8.81 (2H, d, *J* = 4.8 Hz, H-4, H-6), 7.98–7.33 (16H, m, Ar, H-5), 6.39 (1H, pt, *J* = 10.0, 9.7 Hz, H-2′ or H-3′ or H-4′), 5.80 (1H, pt, *J* = 9.7, 9.6 Hz, H-2′ or H-3′ or H-4′), 5.68 (1H, pt, *J* = 10.2, 10.2 Hz, H-2′ or H-3′ or H-4′), 5.42 (1H, d, *J* = 10.0 Hz, H-1′), 4.65 (1H, dd, *J* = 12.3, 3.0 Hz, H-6′a), 4.52 (1H, dd, *J* = 12.3, 5.0 Hz, H-6′b), 4.44 (1H, ddd, *J* = 10.0, 5.0, 3.0 Hz, H-5′); ^13^C NMR (100 MHz, CDCl_3_) δ (ppm): 166.2, 165.3 (2), 162.8 (3 × C=O, C-2), 157.9 (C-4, C-6), 133.8, 133.2, 130.1–128.4 (Ar), 121.5 (C-5), 85.8, 80.4, 77.2, 73.0, 69.2 (C-1′–C-5′), 63.2 (C-6′). ESI-HRMS positive mode (*m*/*z*): calcd for C_31_H_25_N_3_O_9_Na^+^ [M+Na]^+^ 606.1483. Found: 606.1483.

2-(2′-Deoxy-2′-nitro-3′,4′,6′-tri-*O*-benzoyl-β-d-glucopyranosyl)pyrazine (**6d**)

Prepared from compound **2d** (50 mg, 0.092 mmol) and benzoyl chloride (64 μL, 0.55 mmol, 6 eq.) according to general procedure III. Reaction time: 5 d. Purification by column chromatography (1:2 EtOAc-hexane) yielded 44 mg (82%) of a white, amorphous solid. R_f_ = 0.14 (1:2 EtOAc-hexane). ^1^H NMR (400 MHz, CDCl_3_) δ (ppm): 8.83 (1H, d, *J* = 1.5 Hz, H-3), 8.62 (1H, d, *J* = 2.5 Hz, H-6), 8.55 (1H, dd, *J* = 2.5, 1.5 Hz, H-5), 8.01–7.35 (15H, m, Ar), 6.38 (1H, pt, *J* = 9.4, 9.3 Hz, H-2′ or H-3′ or H-4′), 5.74 (1H, pt, *J* = 9.7, 9.7 Hz, H-2′ or H-3′ or H-4′), 5.43 (1H, pt, *J* = 9.9, 9.5 Hz, H-2′ or H-3′ or H-4′), 5.39 (1H, d, *J* = 9.8 Hz, H-1′), 4.68 (1H, dd, *J* = 12.3, 2.9 Hz, H-6′a), 4.52 (1H, dd, *J* = 12.3, 5.4 Hz, H-6′b), 4.28 (1H, ddd, *J* = 10.0, 5.4, 2.9 Hz, H-5′); ^13^C NMR (100 MHz, CDCl_3_) δ (ppm): 166.2, 165.3, 165.2 (3 × C=O), 149.7 (C-2), 145.7, 144.7, 144.0 (C-3, C-5, C-6), 133.9, 133.4, 130.1–129.9, 129.5, 128.7–128.6, 128.5, 128.3 (Ar), 86.3, 77.4, 77.1, 73.1, 69.1 (C-1′–C-5′), 63.0 (C-6′). ESI-HRMS positive mode (*m*/*z*): calcd for C_31_H_26_N_3_O_9_^+^ [M+H]^+^ 584.1664; C_31_H_25_N_3_O_9_Na^+^ [M+Na]^+^ 606.1483. Found: [M+H]^+^ 584.1659; [M+Na]^+^ 606.1477.

2-(2′-Amino-2′-deoxy-3′,4′,6′-tri-*O*-benzoyl-β-d-glucopyranosyl)pyridine (**7a**)

Compound **6a** (0.10 g, 0.17 mmol) and Zn powder (0.11 g, 1.71 mmol, 10 eq.) were suspended in a solvent mixture of THF (10 mL) and water (5 mL). To this stirred mixture, a 2 M aq. solution of HCl was added (2.6 mL, 5.14 mmol, 30 eq.). The reaction mixture was further stirred at rt until the TLC (95:5 CHCl_3_-MeOH) showed total consumption of the starting material (5 h). The reaction was quenched by the addition of sat. aq. NaHCO_3_ solution (50 mL). The insoluble inorganic salts and the rest of the Zn were filtered off, and the remaining solution was extracted with CH_2_Cl_2_ (2 × 50 mL). The combined organic phase was extracted with water (50 mL), then with brine (50 mL), dried over MgSO_4_ and filtered. The solvent was removed under reduced pressure. The residue was purified by column chromatography (100:1 CHCl_3_-MeOH) to yield 36 mg (38%) of a white, amorphous solid. R_f_ = 0.47 (50:1 CHCl_3_-MeOH); [α]_D_ = −16 (c 0.5, CHCl_3_). ^1^H NMR (400 MHz, CDCl_3_) δ (ppm): 8.60 (1H, ddd, *J* = 4.9, 1.9, 0.9 Hz, H-6), 8.01–7.25 (18H, m, Ar, H-3, H-4, H-5), 5.71 (1H, pt, *J* = 9.5, 9.5 Hz, H-4′), 5.66 (1H, pt, *J* = 9.5, 9.5 Hz, H-3′), 4.63 (1H, dd, *J* = 12.2, 3.1 Hz, H-6′a), 4.53 (1H, d, *J* = 9.5 Hz, H-1′), 4.52 (1H, dd, *J* = 12.2, 5.3 Hz, H-6′b), 4.25 (1H, ddd, *J* = 9.4, 5.3, 3.1 Hz, H-5′), 3.58 (1H, pt, *J* = 9.7, 9.6 Hz, H-2′), 1.71 (2H, s, NH_2_); ^13^C NMR (100 MHz, CDCl_3_) δ (ppm): 166.6, 166.3, 165.7 (3 × C=O), 157.7 (C-2), 149.1 (C-6), 137.2 (C-4), 133.4, 133.3, 133.1, 129.9–128.4 (Ar), 123.6, 122.9 (C-3, C-5), 83.9, 77.7, 76.6, 70.3 (C-1′, C-3′–C-5′), 64.0 (C-6′), 56.7 (C-2′). ESI-HRMS positive mode (*m*/*z*): calcd for C_32_H_29_N_2_O_7_^+^ [M+H]^+^ 553.1969; C_32_H_28_N_2_O_7_Na^+^ [M+Na]^+^ 575.1789. Found: [M+H]^+^ 553.1970; [M+Na]^+^ 575.1789.

3-(2′-Amino-2′-deoxy-3′,4′,6′-tri-*O*-benzoyl-β-d-glucopyranosyl)pyridazine (**7b**)

Prepared from compound **9b** (105 mg, 0.16 mmol) according to general procedure VI. Purification by column chromatography (95:5 CHCl_3_-MeOH) yielded 78 mg (88%) of a white, amorphous solid. R_f_ = 0.31 (95:5 CHCl_3_-MeOH); [α]_D_ = +7 (c 0.5, CHCl_3_). ^1^H NMR (400 MHz, CDCl_3_) δ (ppm): 9.17 (1H, d, *J* = 5.0 Hz, H-6), 8.02–7.32 (17H, m, Ar, H-4, H-5), 5.70 (2H, m, H-3′, H-4′), 4.82 (1H, d, *J* = 9.7 Hz, H-1′), 4.65 (1H, dd, *J* = 12.2, 2.9 Hz, H-6′a), 4.52 (1H, dd, *J* = 12.2, 5.2 Hz, H-6′b), 4.32–4.27 (1H, m, H-5′), 3.55 (1H, pt, *J* = 9.7, 9.6 Hz, H-2′), 1.78 (2H, s, NH_2_); ^13^C NMR (100 MHz, CDCl_3_) δ (ppm): 166.6, 166.3, 165.6 (3 × C=O), 160.6 (C-3), 151.5 (C-6), 133.5, 133.4, 133.2, 129.9–129.8, 129.7, 129.3, 129.0, 128.5–128.4 (Ar), 127.5, 125.8 (C-4, C-5), 82.3, 77.4, 76.7, 69.9 (C-1′, C-3′–C-5′), 63.6 (C-6′), 56.7 (C-2′). ESI-HRMS positive mode (*m*/*z*): calcd for C_31_H_28_N_3_O_7_^+^ [M+H]^+^ 554.1922; C_31_H_27_N_3_O_7_Na^+^ [M+Na]^+^ 576.1741. Found: [M+H]^+^ 554.1922; [M+Na]^+^ 576.1740.

2-(2′-Amino-2′-deoxy-3′,4′,6′-tri-*O*-benzoyl-β-d-glucopyranosyl)pyrimidine (**7c**)

Prepared from compound **9c** (90 mg, 0.14 mmol) according to general procedure VI. Purification by column chromatography (95:5 CHCl_3_-MeOH) yielded 73 mg (96%) of a white, amorphous solid. R_f_ = 0.33 (95:5 CHCl_3_-MeOH); [α]_D_ = −15 (c 0.5, CHCl_3_). ^1^H NMR (400 MHz, CDCl_3_) δ (ppm): 8.84 (2H, d, *J* = 4.9 Hz, H-4, H-6), 7.99–7.30 (16H, m, Ar, H-5), 5.74 (1H, pt, *J* = 9.7, 9.6 Hz, H-3′ or H-4′), 5.65 (1H, pt, *J* = 9.8, 9.6 Hz, H-3′ or H-4′), 4.67 (1H, d, *J* = 9.9 Hz, H-1′), 4.60 (1H, dd, *J* = 12.2, 3.2 Hz, H-6′a), 4.51 (1H, dd, *J* = 12.2, 5.4 Hz, H-6′b), 4.29 (1H, ddd, *J* = 9.2, 5.4, 3.2 Hz, H-5′), 3.88 (1H, pt, *J* = 10.0, 9.8 Hz, H-2′), 1.46 (2H, s, NH_2_); ^13^C NMR (90 MHz, CDCl_3_) δ (ppm): 166.8, 166.3, 165.9, 165.6 (3 × C=O, C-2), 157.7 (C-4, C-6), 133.4, 133.3, 133.0, 130.0–129.8, 129.4, 129.2, 128.5–128.3 (Ar), 120.9 (C-5), 85.2, 77.8, 77.0, 70.4 (C-1′, C-3′–C-5′), 64.2 (C-6′), 55.3 (C-2′). ESI-HRMS positive mode (*m*/*z*): calcd for C_31_H_28_N_3_O_7_^+^ [M+H]^+^ 554.1922; C_31_H_27_N_3_O_7_Na^+^ [M+Na]^+^ 576.1741. Found: [M+H]^+^ 554.1916; [M+Na]^+^ 576.1734.

2-(2′-Amino-2′-deoxy-3′,4′,6′-tri-*O*-benzoyl-β-d-glucopyranosyl)pyrazine (**7d**)

Prepared from compound **9d** (0.12 g, 0.18 mmol) according to general procedure VI. Purification by column chromatography (95:5 CHCl_3_-MeOH) yielded 98 mg (96%) of a white, amorphous solid. R_f_ = 0.32 (95:5 CHCl_3_-MeOH); [α]_D_ = −7 (c 0.5, CHCl_3_). ^1^H NMR (400 MHz, CDCl_3_) δ (ppm): 8.82 (1H, d, *J* = 1.2 Hz, H-3), 8.58–8.56 (2H, m, H-5, H-6), 8.01–7.31 (15H, m, Ar), 5.71 (1H, pt, *J* = 9.7, 9.4 Hz, H-3′ or H-4′), 5.68 (1H, pt, *J* = 9.7, 9.4 Hz, H-3′ or H-4′), 4.65 (1H, dd, *J* = 12.3, 3.0 Hz, H-6′a), 4.62 (1H, d, *J* = 9.8 Hz, H-1′), 4.51 (1H, dd, *J* = 12.3, 5.3 Hz, H-6′b), 4.28 (1H, ddd, *J* = 8.9, 5.3, 3.0 Hz, H-5′), 3.64 (1H, pt, *J* = 9.7, 9.7 Hz, H-2′), 1.82 (2H, s, NH_2_); ^13^C NMR (100 MHz, CDCl_3_) δ (ppm): 166.6, 166.2, 165.6 (3 × C=O), 153.1 (C-2), 145.1, 144.6, 143.6 (C-3, C-5, C-6), 133.5, 133.4, 133.1, 129.9–129.8, 129.7, 129.2, 129.0, 128.5–128.4 (Ar), 81.9, 77.4, 76.7, 70.0 (C-1′, C-3′–C-5′), 63.7 (C-6′), 56.1 (C-2′). ESI-HRMS positive mode (*m*/*z*): calcd for C_31_H_28_N_3_O_7_^+^ [M+H]^+^ 554.1922; C_31_H_27_N_3_O_7_Na^+^ [M+Na]^+^ 576.1741. Found: [M+H]^+^ 554.1916; [M+Na]^+^ 576.1735.

2-(2′-Amino-2′-deoxy-3′,4′,6′-tri-*O*-benzoyl-β-d-glucopyranosyl)quinoline (**7e**)

Prepared from compound **9e** (0.12 g, 0.16 mmol) according to general procedure VI. Purification by column chromatography (1:1 EtOAc-hexane) yielded 83 mg (84%) of a white, amorphous solid. R_f_ = 0.18 (1:1 EtOAc-hexane); [α]_D_ = +30 (c 0.5, CHCl_3_). ^1^H NMR (500 MHz, CDCl_3_) δ (ppm): 8.22–7.31 (21H, m, Ar, H-3–H-8), 5.77–5.71 (2H, m, H-3′, H-4′), 4.73 (1H, d, *J* = 9.5 Hz, H-1′), 4.66 (1H, dd, *J* = 12.2, 2.8 Hz, H-6′a), 4.54 (1H, dd, *J* = 12.2, 5.4 Hz, H-6′b), 4.31 (1H, ddd, *J* = 9.1, 5.4, 2.8 Hz, H-5′), 3.69 (1H, pt, *J* = 9.5, 9.1 Hz, H-2′), 1.83 (2H, br s, NH_2_); ^13^C NMR (90 MHz, CDCl_3_) δ (ppm): 166.6, 166.3, 165.7 (3 × C=O), 157.9, 147.2 (C-2, C-8a), 137.4, 133.4, 133.3, 133.1, 130.0–129.6, 129.5, 129.2, 128.5–128.4, 128.0, 127.7, 127.0, 119.9 (Ar, C-3–C-8, C-4a), 84.2, 77.6, 76.7, 70.3 (C-1′, C-3′–C-5′), 63.9 (C-6′), 56.5 (C-2′). ESI-HRMS positive mode (*m*/*z*): calcd for C_36_H_31_N_2_O_7_^+^ [M+H]^+^ 603.2126. Found: 603.2123.

2-(2′-(*tert*-Butoxycarbonyl)amino-2′-deoxy-β-d-glucopyranosyl)pyrimidine (**8c**)

Prepared from compound **5c** (60 mg, 0.25 mmol) and Boc_2_O (0.11 g, 0.50 mmol) according to general procedure IV. Purification by column chromatography (9:1 CHCl_3_-MeOH) yielded 57 mg (67%) of a white, amorphous solid. R_f_ = 0.37 (4:1 CHCl_3_-MeOH). ^1^H NMR (400 MHz, D_2_O) δ (ppm): 8.81 (2H, d, *J* = 5.0 Hz, H-4, H-6), 7.55 (1H, t, *J* = 5.0 Hz, H-5), 4.51 (1H, d, *J* = 9.7 Hz, H-1′), 3.94 (1H, dd, *J* = 12.4, 1.9 Hz, H-6′a), 3.86 (1H, dd, *J* = 12.4, 4.6 Hz, H-6′b), 3.74–3.61 (4H, m, H-2′–H-5′), 1.21 (6H, s, 2 × C*H_3_*), 1.10 (3H, s, C*H_3_*); ^13^C NMR (100 MHz, D_2_O + 2 drops of CD_3_OD) δ (ppm): 165.0 (C-2), 157.7 (C-4, C-6), 156.8 (C=O), 121.6 (C-5), 81.2, 79.7, 74.5, 69.8 (C-1′, C-3′–C-5′), 80.9 (*C*(CH_3_)_3_), 60.9 (C-6′), 56.8 (C-2′), 27.6 (3 × *C*H_3_). ESI-HRMS positive mode (*m*/*z*): calcd for C_15_H_23_N_3_O_6_Na^+^ [M+Na]^+^ 364.1479; C_30_H_46_N_6_O_12_Na^+^ [2M+Na]^+^ 705.3066. Found: [M+Na]^+^ 364.1474; [2M+Na]^+^ 705.3057.

2-(2′-(*tert*-Butoxycarbonyl)amino-2′-deoxy-β-d-glucopyranosyl)pyrazine (**8d**)

Prepared from compound **5d** (0.10 g, 0.42 mmol) and Boc_2_O (0.18 g, 0.83 mmol) according to general procedure IV. Purification by column chromatography (9:1 CHCl_3_-MeOH) yielded 94 mg (67%) of a white, amorphous solid. R_f_ = 0.37 (4:1 CHCl_3_-MeOH). ^1^H NMR (400 MHz, CD_3_OD) δ (ppm): 8.72 (1H, d, *J* = 1.5 Hz, H-3), 8.56 (1H, dd, *J* = 2.7, 1.5 Hz, H-5), 8.53 (1H, d, *J* = 2.7 Hz, H-6), 4.41 (1H, d, *J* = 9.1 Hz, H-1′), 3.90 (1H, dd, *J* = 12.1, 2.2 Hz, H-6′a), 3.77 (1H, dd, *J* = 12.1, 5.1 Hz, H-6′b), 3.62–3.52 (3H, m, H-2′, H-3′, H-4′), 3.44 (1H, ddd, *J* = 9.0, 5.1, 2.2 Hz, H-5′), 1.22 (9H, s, 3 × C*H_3_*); ^13^C NMR (100 MHz, CD_3_OD) δ (ppm): 157.5, 155.3 (C=O, C-2), 145.0, 144.9, 144.8 (C-3, C-5, C-6), 82.3, 81.3, 76.6, 71.8 (C-1′, C-3′–C-5′), 79.9 (*C*(CH_3_)_3_), 62.7 (C-6′), 58.6 (C-2′), 28.6 (3 × *C*H_3_). ESI-HRMS positive mode (*m*/*z*): calcd for C_15_H_23_N_3_O_6_Na^+^ [M+Na]^+^ 364.1479. Found: 364.1471.

2-(2′-(*tert*-Butoxycarbonyl)amino-2′-deoxy-β-d-glucopyranosyl)quinoline (**8e**)

Prepared from compound **5e** (25 mg, 0.086 mmol) and Boc_2_O (37.6 mg, 0.172 mmol) according to general procedure IV. Purification by column chromatography (9:1 CHCl_3_-MeOH) yielded 27 mg (80%) of a white, amorphous solid. R_f_ = 0.35 (4:1 CHCl_3_-MeOH). ^1^H NMR (360 MHz, CD_3_OD) δ (ppm): 8.29 (1H, d, *J* = 8.5 Hz, H-4), 8.05 (1H, dd, *J* = 8.5, 1.2 Hz, H-8), 7.90 (1H, dd, *J* = 8.1, 1.4 Hz, H-5), 7.75 (1H, ddd, *J* = 8.5, 6.9, 1.5 Hz, H-7), 7.69 (1H, d, *J* = 8.5 Hz, H-3), 7.58 (1H, ddd, *J* = 8.1, 6.9, 1.2 Hz, H-6), 4.49 (1H, d, *J* = 9.4 Hz, H-1′), 3.94 (1H, dd, *J* = 12.1, 2.3 Hz, H-6′a), 3.82 (1H, dd, *J* = 12.1, 5.2 Hz, H-6′b), 3.70–3.56 (3H, m, H-2′, H-3′, H-4′), 3.49 (1H, ddd, *J* = 9.5, 5.2, 2.3 Hz, H-5′), 1.00 (6H, s, 2 × C*H_3_*), 0.79 (3H, s, C*H_3_*); ^13^C NMR (90 MHz, CD_3_OD) δ (ppm): 160.2, 157.4, 148.0 (C=O, C-2, C-8a,), 138.2, 130.9, 129.2, 128.9, 127.8, 121.7 (C-3–C-8), 129.4 (C-4a), 83.6, 82.3, 77.0, 72.0 (C-1′, C-3′–C-5′), 79.8 (*C*(CH_3_)_3_), 62.9 (C-6′) 58.8 (C-2′), 28.4 (3 × *C*H_3_). ESI-HRMS positive mode (*m*/*z*): calcd for C_20_H_26_N_2_O_6_Na^+^ [M+Na]^+^ 413.1683. Found: 413.1683.

3-(2′-(*tert*-Butoxycarbonyl)amino-2′-deoxy-3′,4′,6′-tri-*O*-benzoyl-β-d-glucopyranosyl)pyridazine (**9b**)

A degassed, vigorously stirred suspension of 10% Pd(C) (65 mg) in dry EtOH (13 mL) was saturated with H_2_, and compound **4b** (0.13 g, 0.48 mmol) was added. The reaction mixture was heated at reflux temperature until the TLC (3:2 CHCl_3_-MeOH) indicated complete conversion of the starting material. After completion of the reaction (3 h), the catalyst was filtered off through a pad of celite and washed with EtOH. The resulting solution was then evaporated under reduced pressure. Purification of the residue by column chromatography (3:2 CHCl_3_-MeOH) yielded 100 mg of a white, amorphous solid containing the desired 3-(2′-amino-2′-deoxy-β-d-glucopyranosyl)pyridazine **5b**, along with unidentified impurities. This mixture was dissolved in a solvent mixture of water (5 mL) and 1,4-dioxane (5 mL), and Boc_2_O (0.21 g, 0.96 mmol) was added. The reaction mixture was stirred at rt until the TLC (9:1 CHCl_3_-MeOH) showed complete transformation of **5b** (1 day). Then, the solvents were removed under reduced pressure. Column chromatographic purification of the residue (9:1 CHCl_3_-MeOH) resulted in 70 mg of 3-(2′-(*tert*-butoxycarbonyl)amino-2′-deoxy-β-d-glucopyranosyl)pyridazine (**8b**) contaminated with inseparable impurities. To a solution of the resulting **8b** in dry pyridine (5 mL), benzoyl chloride (0.2 mL, 1.72 mmol) was added at rt. The reaction mixture was stirred at 60 °C for 1 h. Since the TLC (1:1 EtOAc-hexane) showed incompleteness of the reaction, an additional portion of benzoyl chloride (0.2 mL, 1.72 mmol) was added to the reaction mixture, and heating was continued for 1 h. The reaction mixture was allowed to cool to rt and further stirred overnight. The reaction mixture was then diluted with CH_2_Cl_2_ (50 mL) and extracted with sat. aq. solution of NaHCO_3_ (25 mL), then with water (25 mL). The separated organic phase was dried over MgSO_4_ and filtered, and the solvents were removed under diminished pressure. The residue was purified by column chromatography (1:1 EtOAc-hexane) to afford the title compound **9b** (83 mg, 27% for 3 steps) as a white, amorphous solid. R_f_ = 0.28 (1:1 EtOAc-hexane. ^1^H NMR (400 MHz, CDCl_3_) δ (ppm): 9.12 (1H, dd, *J* = 4.9, 1.7 Hz, H-6), 8.04–7.32 (17H, m, Ar, H-4, H-5), 5.82 (1H, pt, *J* = 9.3, 9.3 Hz, H-3′ or H-4′), 5.79 (1H, pt, *J* = 9.7, 9.5 Hz, H-3′ or H-4′), 5.12 (1H, d, *J* = 9.9 Hz, NH), 5.02 (1H, d, *J* = 10.3 Hz, H-1′), 4.67 (1H, dd, *J* = 12.3, 2.8 Hz, H-6′a), 4.50 (1H, dd, *J* = 12.3, 4.8 Hz, H-6′b), 4.30 (1H, ddd, *J* = 9.5, 4.8, 2.8 Hz, H-5′), 4.28 (1H, pt, *J* = 10.1, 10.0 Hz, H-2′), 1.05 (9H, s, 3 × C*H_3_*); ^13^C NMR (100 MHz, CDCl_3_) δ (ppm): 166.7, 166.2, 165.4 (3 × C=O), 159.6, 155.0 (C=O, C-3), 151.4 (C-6), 133.6, 133.4, 133.2, 130.0–129.8, 129.7, 129.1, 128.9, 128.6–128.4 (Ar), 127.3, 125.7 (C-4, C-5), 81.3, 76.7, 74.3, 69.8 (C-1′, C-3′–C-5′), 80.0 (*C*(CH_3_)_3_), 63.3 (C-6′), 55.9 (C-2′), 27.9 (3 × *C*H_3_). ESI-HRMS positive mode (*m*/*z*): calcd for C_36_H_35_N_3_O_9_Na^+^ [M+Na]^+^ 676.2266; C_72_H_70_N_6_O_18_Na^+^ [2M+Na]^+^ 1329.4639. Found: [M+Na]^+^ 676.2256; [2M+Na]^+^ 1329.4641.

2-(2′-(*tert*-Butoxycarbonyl)amino-2′-deoxy-3′,4′,6′-tri-*O*-benzoyl-β-d-glucopyranosyl)pyrimidine (**9c**)

Prepared from compound **8c** (60 mg, 0.18 mmol) and benzoyl chloride (0.15 mL, 1.29 mmol, 7.2 eq.) according to general procedure V. Purification by column chromatography (1:1 EtOAc-hexane) yielded 92 mg (80%) of a white, amorphous solid. R_f_ = 0.22 (1:1 EtOAc-hexane). ^1^H NMR (400 MHz, CDCl_3_) δ (ppm): 8.82 (2H, d, *J* = 4.9 Hz, H-4, H-6), 7.98–7.27 (16H, m, Ar, H-5), 5.86 (1H, pt, *J* = 9.9, 9.6 Hz, H-3′ or H-4′), 5.80 (1H, pt, *J* = 9.6, 9.4 Hz, H-3′ or H-4′), 4.98 (1H, d, *J* = 9.5 Hz, NH), 4.90 (1H, d, *J* = 10.2 Hz, H-1′), 4.63 (1H, dd, *J* = 12.3, 3.5 Hz, H-6′a), 4.58 (1H, dd, *J* = 12.3, 5.4 Hz, H-6′b), 4.55 (1H, pt, *J* = 10.2, 9.7 Hz, H-2′), 4.30 (1H, ddd, *J* = 9.2, 5.4, 3.5 Hz, H-5′), 1.09 (9H, s, 3 × C*H_3_*); ^13^C NMR (100 MHz, CDCl_3_) δ (ppm): 166.7, 166.3, 165.4, 165.2 (3 × C=O, C-2), 154.6 (C=O), 157.4 (C-4, C-6), 133.4, 133.3, 133.0, 130.0–129.8, 129.7, 129.2, 129.0, 128.4–128.2 (Ar), 120.7 (C-5), 82.8, 76.7, 74.6, 70.2 (C-1′, C-3′–C-5′), 79.6 (*C*(CH_3_)_3_), 64.1 (C-6′), 55.2 (C-2′), 28.0 (3 × *C*H_3_). ESI-HRMS positive mode (*m*/*z*): calcd for C_36_H_35_N_3_O_9_Na^+^ [M+Na]^+^ 676.2266. Found: 676.2256.

2-(2′-(*tert*-Butoxycarbonyl)amino-2′-deoxy-3′,4′,6′-tri-*O*-benzoyl-β-d-glucopyranosyl)pyrazine (**9d**)

Prepared from compound **8d** (90 mg, 0.26 mmol) and benzoyl chloride (0.22 mL, 1.89 mmol, 7.2 eq.) according to general procedure V. Purification by column chromatography (1:1 EtOAc-hexane) yielded 122 mg (71%) of a white, amorphous solid. R_f_ = 0.36 (1:1 EtOAc-hexane). ^1^H NMR (400 MHz, CDCl_3_) δ (ppm): 8.83 (1H, s, H-3), 8.55–8.53 (2H, m, H-5, H-6), 8.02–7.30 (15H, m, Ar), 5.88 (1H, d, *J* = 9.8, 9.6 Hz, H-3′ or H-4′), 5.83 (1H, d, *J* = 9.5, 9.3 Hz, H-3′ or H-4′), 5.15 (1H, d, *J* = 9.3 Hz, NH), 4.83 (1H, d, *J* = 10.2 Hz, H-1′), 4.68 (1H, dd, *J* = 12.3, 2.9 Hz, H-6′a), 4.53 (1H, dd, *J* = 12.3, 4.9 Hz, H-6′b), 4.35 (1H, pt, *J* = 9.8, 9.7 Hz, H-2′), 4.34–4.29 (H, m, H-5′), 1.09 (9H, s, 3 × C*H_3_*); ^13^C NMR (90 MHz, CDCl_3_) δ (ppm): 166.8, 166.3, 165.4 (3 × C=O), 154.8, 152.4 (C=O, C-2), 144.6 (2), 143.4 (C-3, C-5, C-6), 133.5, 133.4, 133.2, 130.0–129.8, 129.7, 129.1, 129.0, 128.5–128.4 (Ar), 80.7, 76.8, 74.3, 70.0 (C-1′, C-3′–C-5′), 79.9 (*C*(CH_3_)_3_), 63.5 (C-6′), 55.9 (C-2′), 28.0 (3 × *C*H_3_). ESI-HRMS positive mode (*m*/*z*): calcd for C_36_H_35_N_3_O_9_Na^+^ [M+Na]^+^ 676.2266. Found: 676.2260.

2-(2′-(*tert*-Butoxycarbonyl)amino-2′-deoxy-3′,4′,6′-tri-*O*-benzoyl-β-d-glucopyranosyl)quinoline (**9e**)

Prepared from compound **8e** (50 mg, 0.13 mmol) and benzoyl chloride (0.11 mL, 0.95 mmol, 7.2 eq.) according to general procedure V. Purification by column chromatography (1:2 EtOAc-hexane) yielded 73 mg (81%) of a white, amorphous solid. R_f_ = 0.21 (1:2 EtOAc-hexane). ^1^H NMR (500 MHz, CDCl_3_) δ (ppm): 8.23–7.32 (21H, m, Ar, H-3–H-8), 5.84 (1H, pt, *J* = 9.6, 9.5 Hz, H-3′ or H-4′), 5.77 (1H, dd, *J* = 10.0, 9.8 Hz, H-3′ or H-4′), 4.98 (1H, d, *J* = 9.8 Hz, NH), 4.89 (1H, d, *J* = 10.2 Hz, H-1′), 4.67 (1H, dd, *J* = 12.2, 2.9 Hz, H-6′a), 4.52 (1H, dd, *J* = 12.2, 4.8 Hz, H-6′b), 4.36 (1H, q, *J* = 10.2 Hz, H-2′), 4.28 (1H, ddd, *J* = 9.7, 4.8, 2.9 Hz, H-5′), 0.86 (9H, s, 3 × C*H_3_*); ^13^C NMR (90 MHz, CDCl_3_) δ (ppm): 170.8, 166.4, 165.5 (3 × C=O), 157.4, 155.1, 146.5 (C=O, C-2, C-8a), 137.9, 133.5, 133.3, 133.2, 130.3–129.8, 129.4, 129.1, 128.5–128.3, 128.2, 127.9, 127.0, 119.5 (Ar, C-3–C-8, C-4a), 82.5, 76.6, 74.7, 70.1 (C-1′, C-3′–C-5′), 79.5 (*C*(CH_3_)_3_), 63.5 (C-6′), 56.1 (C-2′), 27.7 (3 × *C*H_3_). ESI-HRMS positive mode (*m*/*z*): calcd for C_41_H_38_N_2_O_9_Na^+^ [M+Na]^+^ 725.2470. Found: 725.2473.

Complex **Ru-3a**

Prepared from compound **3a** (47 mg, 0.092 mmol, 1.9 eq.), **Ru-dimer** (30 mg, 0.049 mmol) and TlPF_6_ (34 mg, 0.097 mmol) according to general procedure VII. Purification by column chromatography (95:5 CHCl_3_-MeOH) yielded 63 mg (74%) of a yellow solid. R_f_ = 0.50 (95:5 CHCl_3_-MeOH). ^1^H NMR (400 MHz, CDCl_3_) δ (ppm): 9.02 (1H, d, *J* = 5.5 Hz, H-6), 7.86 (1H, dd, *J* = 8.1, 1.8 Hz, H-3), 7.82 (1H, td, *J* = 8.1, 1.5 Hz, H-4), 7.40–7.26 (16H, m, Ar, H-5), 5.84, 5.80, 5.59, 5.13 (4 × 1H, 4 d, *J* = 5.9 Hz in each, 4 × *p*-cym-CH_Ar_), 5.34 (1H, pt, *J* = 10.8 Hz, NH_2_), 4.89, 4.59 (2 × 1H, 2 d, *J* = 12.1 Hz in both, Ph*CH*_2_), 4.78, 4.56 (2 × 1H, 2 d, *J* = 12.3 Hz in both, Ph*CH*_2_), 4.75, 4.65 (2 × 1H, 2 d, *J* = 11.3 Hz in both, Ph*CH*_2_), 4.54 (1H, d, *J* = 10.2 Hz, H-1′), 4.23 (1H, pt, *J* = 8.9, 8.8 Hz, H-3′), 3.99 (1H, ddd, *J* = 9.4, 5.6, 2.6, H-5′), 3.84–3.77 (2H, m, H-6′a,b), 3.53 (1H, pt, *J* = 9.1, 9.0 Hz, H-4′), 3.20 (1H, dd, *J* = 10.8, 5.3 Hz, NH_2_), 2.58 (1H, hept, *J* = 6.9 Hz, *i*-Pr-C*H*), 2.03–1.94 (1H, m, H-2′), 1.70 (3H, s, C_6_H_4_-C*H_3_*), 1.16, 1.02 (2 × 3H, 2 d, *J* = 6.9 Hz in both, 2 × *i*-Pr-C*H*_3_); ^13^C NMR (100 MHz, CDCl_3_) δ (ppm): 162.0 (C-2), 156.8 (C-6), 139.4 (C-4), 138.4, 138.1 (2), 129.1–127.8 (Ar), 124.5, 123.2 (C-3, C-5), 104.9, 98.7 (2 × *p*-cym-C_qAr_), 86.6, 84.8, 83.8, 83.4, 82.9, 77.7, 76.8, 76.0 (4 × *p*-cym-CH_Ar_, C-1′, C-3′–C-5′), 75.1, 74.2, 73.5 (3 × Ph*CH*_2_), 68.7 (C-6′), 53.6 (C-2′), 31.0 (*i*-Pr-*C*H), 23.2, 21.5 (2 × *i*-Pr-*C*H_3_), 17.7 (C_6_H_4_-*C*H_3_). ESI-HRMS positive mode (*m*/*z*): calcd for C_42_H_48_ClN_2_O_4_Ru^+^ [M-PF_6_]^+^ 781.2349. Found: 781.2346.

Complex **Os-3a**

Prepared from compound **3a** (12.3 mg, 0.024 mmol, 1.9 eq.), **Os-dimer** (10.0 mg, 0.013 mmol) and TlPF_6_ (8.7 mg, 0.025 mmol) according to general procedure VII. After purification by column chromatography (95:5 CHCl_3_-MeOH), the complex was dissolved in CHCl_3_ (1 mL), and diisopropyl ether (8 mL) was added. The precipitated product was filtered off, then washed with CHCl_3_-diisopropyl ether (1:8, 1 mL) to yield 15.6 mg (64%) of a dark purple solid. R_f_ = 0.58 (95:5 CHCl_3_-MeOH). ^1^H NMR (400 MHz, CDCl_3_) δ (ppm): 8.87 (1H, dd, *J* = 6.0, 1.5 Hz, H-6), 7.90 (1H, d, *J* = 7.8 Hz, H-3), 7.81 (1H, td, *J* = 7.8, 1.5 Hz, H-4), 7.41–7.26 (16H, m, Ar, H-5), 6.09, 6.08, 5.82, 5.27 (4 × 1H, 4 d, *J* = 5.7 Hz in each, 4 × *p*-cym-CH_Ar_), 5.90–5.76 (1H, broad signal, NH_2_), 4.91, 4.77 (2 × 1H, 2 d, *J* = 12.3 Hz in both, Ph*CH*_2_), 4.76, 4.67 (2 × 1H, 2 d, *J* = 11.5 Hz in both, Ph*CH*_2_), 4.59, 4.55 (2 × 1H, 2 d, *J* = 12.2 Hz in both, Ph*CH*_2_), 4.50 (1H, d, *J* = 10.2 Hz, H-1′), 4.22 (1H, pt, *J* = 9.0, 8.8 Hz, H-3′ or H-4′), 4.00–3.98 (1H, m, H-5′), 3.84–3.77 (2H, m, H-6′a,b), 3.67 (1H, dd, *J* = 11.4, 4.6 Hz, NH_2_), 3.57 (1H, pt, *J* = 9.2, 9.1 Hz, H-3′ or H-4′), 2.47 (1H, hept, *J* = 7.0 Hz, *i*-Pr-C*H*), 2.32–2.23 (1H, m, H-2′), 1.75 (3H, s, C_6_H_4_-C*H_3_*), 1.19, 0.98 (2 × 3H, 2 d, *J* = 6.9 Hz in both, 2 × *i*-Pr-C*H*_3_); ^13^C NMR (100 MHz, CDCl_3_) δ (ppm): 161.3 (C-2), 157.7 (C-6), 139.5 (C-4), 138.4, 138.1 (2), 129.9–127.8 (Ar), 124.9, 122.5 (C-4, C-5), 95.0, 89.9 (2 × *p*-cym-C_qAr_), 82.7, 78.3, 77.7, 76.8, 76.3, 75.6, 75.4, 73.6 (4 × *p*-cym-CH_Ar_, C-1′, C-3′–C-5′), 75.2, 74.2, 73.5 (3 × Ph*CH*_2_), 68.7 (C-6′), 53.6 (C-2′), 31.1 (*i*-Pr-*C*H), 23.6, 21.6 (2 × *i*-Pr-*C*H_3_), 17.6 (C_6_H_4_-*C*H_3_). ESI-HRMS positive mode (*m*/*z*): calcd for C_42_H_47_N_2_O_4_Os^+^ [M-HCl-PF_6_]^+^ 835.3148. Found: 835.3143.

Complex **Ir-3a**

Prepared from compound **3a** (12.2 mg, 0.024 mmol, 1.9 eq.), **Ir-dimer** (10.0 mg, 0.013 mmol) and TlPF_6_ (8.8 mg, 0.025 mmol) according to general procedure VII. Purification by column chromatography (95:5 CHCl_3_-MeOH) yielded 22.7 mg (93%) of a yellow solid. R_f_ = 0.51 (95:5 CHCl_3_-MeOH). ^1^H NMR (400 MHz, CDCl_3_) δ (ppm): 8.58 (1H, d, *J* = 5.8 Hz, H-6), 7.92–7.87 (2H, m, H-3, H-4), 7.42–7.27 (16H, m, Ar, H-5), 4.83, 4.70 (2 × 1H, 2 d, *J* = 12.6 Hz in both, Ph*CH*_2_), 4.78, 4.69 (2 × 1H, 2 d, *J* = 11.4 Hz in both, PhC*H*_2_), 4.60, 4.56 (2 × 1H, 2 d, *J* = 12.1 Hz in both, Ph*CH*_2_), 4.25 (1H, d, *J* = 10.0 Hz, H-1′), 4.12–4.05 (2H, m, H-5′, NH_2_), 4.08 (1H, pt, *J* = 8.1, 8,0 Hz, H-3′ or H-4′), 3.95 (1H, dd, *J* = 11.5, 5.5 Hz, NH_2_), 3.85 (1H, dd, *J* = 10.8, 4.1 Hz, H-6′a), 3.79 (1H, dd, *J* = 10.8, 3.0 Hz, H-6′b), 3.69 (1H, pt, *J* = 8.0, 7.9 Hz, H-3′ or H-4′), 2.55–2.47 (1H, m, H-2′), 1.41 (15H, s, Cp*-C*H*_3_); ^13^C NMR (100 MHz, CDCl_3_) δ (ppm): 160.8 (C-2), 155.3 (C-6), 139.9 (C-4), 138.3, 138.1, 137.8, 129.9, 129.3–127.9 (Ar), 126.0, 123.0 (C-4, C-5), 88.1 (Cp*), 81.5, 77.5, 77.4, 77.1 (C-1′, C-3′–C-5′), 74.3, 74.1, 73.5 (3 × Ph*CH*_2_), 69.0 (C-6′), 54.1 (C-2′), 8.5 (Cp*-*C*H_3_). ESI-HRMS positive mode (*m*/*z*): calcd for C_42_H_49_ClN_2_O_4_Ir^+^ [M-PF_6_]^+^ 873.2999. Found: 873.2997.

Complex **Rh-3a**

Prepared from compound **3a** (15.7 mg, 0.031 mmol, 1.9 eq.), **Rh-dimer** (10.0 mg, 0.016 mmol) and TlPF_6_ (11.3 mg, 0.032 mmol) according to general procedure VII. After purification by column chromatography (95:5 CHCl_3_-MeOH), the complex was dissolved in CHCl_3_ (1 mL), and diisopropyl ether (8 mL) was added. The precipitated product was filtered off, then washed with CHCl_3_-diisopropyl ether (1:8, 1 mL) to yield 23.6 mg (83%) of an orange solid. R_f_ = 0.30 (95:5 CHCl_3_-MeOH).^1^H NMR (400 MHz, CDCl_3_) δ (ppm): 8.61 (1H, dd, *J* = 5.7, 1.7 Hz, H-6), 7.94–7.85 (2H, m, H-3, H-4), 7.46–7.28 (16H, m, Ar, H-5), 4.81, 4.68 (2 × 1H, 2 d, *J* = 12.5 Hz in both, Ph*CH*_2_), 4.77, 4.70 (2 × 1H, 2 d, *J* = 11.2 Hz in both, Ph*CH*_2_), 4.61, 4.58 (2 × 1H, 2 d, *J* = 12.8 Hz in both, Ph*CH*_2_), 4.26 (1H, d, *J* = 9.9 Hz, H-1′), 4.03 (1H, ddd, *J* = 7.6, 4.1, 3.2 Hz, H-5′), 3.96 (1H, pt, *J* = 7.7, 7.6 Hz, H-3′ or H-4′), 3.85 (1H, dd, *J* = 10.8, 4.1 Hz, H-6′a), 3.79 (1H, dd, *J* = 10.8, 3.2 Hz, H-6′b), 3.72 (1H, pt, *J* = 7.7, 7.7 Hz, H-3′ or H-4′), 3.47 (1H, dd, *J* = 11.1, 5.9 Hz, NH_2_), 3.24 (1H, pt, *J* = 10.2 Hz, NH_2_), 2.40–2.32 (1H, m, H-2′), 1.43 (15H, s, Cp*-C*H*_3_); ^13^C NMR (100 MHz, CDCl_3_) δ (ppm): 160.8 (C-2), 154.2 (C-6), 139.8 (C-4), 138.2, 138.1, 137.7, 129.9, 129.2–127.9 (Ar), 125.6, 123.2 (C-3, C-5), 96.5, 96.4 (Cp*), 82.3, 77.7, 77.6, 76.9 (C-1′, C-3′–C-5′), 74.3, 74.0, 73.6 (3 × Ph*CH*_2_), 69.0 (C-6′), 54.4 (C-2′), 8.9 (Cp*-*C*H_3_). ESI-HRMS positive mode (*m*/*z*): calcd for C_42_H_49_ClN_2_O_4_Rh^+^ [M-PF_6_]^+^ 783.2430. Found: 783.2430.

Complex **Ru-3d**

Prepared from compound **3d** (16.7 mg, 0.033 mmol, 2 eq.), **Ru-dimer** (10.0 mg, 0.016 mmol) and TlPF_6_ (11.4 mg, 0.033 mmol) according to general procedure VII. After purification by column chromatography (100:1 CHCl_3_-MeOH), the complex was dissolved in CHCl_3_ (1.5 mL), and diisopropyl ether (12 mL) was added. The precipitated product was filtered off, then washed with diisopropyl ether (1 mL) to yield 10.0 mg (33%) of a brown solid. R_f_ = 0.31 (95:5 CHCl_3_-MeOH). ^1^H NMR (400 MHz, CDCl_3_) δ (ppm): 9.09 (1H, s, H-3), 8.94 (1H, dd, *J* = 3.2, 1.1 Hz, H-5), 8.65 (1H, d, *J* = 3.2 Hz, H-6), 7.43–7.26 (15H, m, Ar), 5.89, 5.85, 5.54, 5.15 (4 × 1H, 4 d, *J* = 6.0 Hz in each, 4 × *p*-cym-CH_Ar_), 5.22–5.16 (1H, broad signal, NH_2_), 4.92, 4.77 (2 × 1H, 2 d, *J* = 12.3 Hz in both, Ph*CH*_2_), 4.76, 4.66 (2 × 1H, 2 d, *J* = 11.3 Hz in both, Ph*CH*_2_), 4.67 (1H, d, *J* = 10.3 Hz, H-1′), 4.56 (2H, s, Ph*CH*_2_), 4.20 (1H, pt, *J* = 9.1, 8.9 Hz, H-3′ or H-4′), 3.99–3.94 (1H, m, H-5′), 3.81–3.77 (2H, m, H-6′a,b), 3.56 (1H, pt, *J* = 9.2, 9.1 Hz, H-3′ or H-4′), 3.16 (1H, dd, *J* = 11.1, 5.1 Hz, NH_2_), 2.56 (1H, hept, *J* = 6.9 Hz, *i*-Pr-*CH*), 2.04–1.95 (1H, m, H-2′), 1.76 (3H, s, C_6_H_4_-C*H_3_*), 1.16, 1.03 (2 × 3H, 2 d, *J* = 6.9 Hz in both, 2 × *i*-Pr-C*H*_3_); ^13^C NMR (100 MHz, CDCl_3_) δ (ppm): 156.5 (C-2), 149.9, 145.3, 145.2 (C-3, C-5, C-6), 138.4, 138.0, 137.9, 129.1–127.9 (Ar), 105.7, 98.9 (2 × *p*-cym-C_qAr_), 86.8, 85.2, 84.1, 84.0, 83.3, 77.5, 77.2, 75.1 (4 × *p*-cym-CH_Ar_, C-1′, C-3′–C-5′), 75.3, 74.4, 73.5 (3 × Ph*CH*_2_), 68.5 (C-6′), 53.2 (C-2′), 31.1 (*i*-Pr-*C*H), 23.0, 21.6 (2 × *i*-Pr-*C*H_3_), 17.8 (C_6_H_4_-*C*H_3_). ESI-HRMS positive mode (*m*/*z*): calcd for C_41_H_47_ClN_3_O_4_Ru^+^ [M-PF_6_]^+^ 782.2307. Found: 782.2315.

Complex **Os-3d**

Prepared from compound **3d** (13.0 mg, 0.025 mmol, 2 eq.), **Os-dimer** (10.0 mg, 0.013 mmol) and TlPF_6_ (8.7 mg, 0.025 mmol) according to general procedure VII. After purification by column chromatography (100:1 CHCl_3_-MeOH), the complex was dissolved in CHCl_3_ (1.5 mL), and diisopropyl ether (12 mL) was added. The precipitated product was filtered off, then washed with diisopropyl ether (1 mL) to yield 12.0 mg (47%) of a greenish–brown solid. R_f_ = 0.27 (95:5 CHCl_3_-MeOH). ^1^H NMR (400 MHz, CDCl_3_) δ (ppm): 9.10 (1H, s, H-3), 8.76 (1H, dd, *J* = 3.3, 1.1 Hz, H-5), 8.56 (1H, d, *J* = 3.3 Hz, H-6), 7.42–7.27 (15H, m, Ar), 6.12, 6.11, 5.80, 5.29 (4 × 1H, 4 d, *J* = 5.8 Hz in each, 4 × *p*-cym-CH_Ar_), 5.90–5.77 (1H, broad signal, NH_2_), 4.92, 4.75 (2 × 1H, 2 d, *J* = 12.3 Hz in both, Ph*CH*_2_), 4.77, 4.68 (2 × 1H, 2 d, *J* = 11.4 Hz in both, Ph*CH*_2_), 4.70–4.65 (1H, broad signal, NH_2_), 4.62 (1H, d, *J* = 10.4 Hz, H-1′), 4.56 (2H, s, Ph*CH*_2_), 4.23 (1H, pt, *J* = 9.1, 9.0 Hz, H-3′ or H-4′), 3.99–3.95 (1H, m, H-5′), 3.83–3.76 (2H, m, H-6′a,b), 3.59 (1H, pt, *J* = 9.3, 9.2 Hz, H-3′ or H-4′), 2.46 (1H, hept, *J* = 6.9 Hz, *i*-Pr-C*H*), 2.33–2.24 (1H, m, H-2′), 1.80 (3H, s, C_6_H_4_-C*H_3_*), 1.18, 0.99 (2 × 3H, 2 d, *J* = 6.9 Hz in both, 2 × *i*-Pr-C*H*_3_); ^13^C NMR (100 MHz, CDCl_3_) δ (ppm): 155.4 (C-2), 150.4, 146.0, 144.8 (C-3, C-5, C-6), 138.4, 138.0, 137.9, 129.1–127.9 (Ar), 96.2, 90.3 (2 × *p*-cym-C_qAr_), 82.5, 78.5, 77.5, 77.1, 76.2, 76.0, 75.3, 74.7 (4 × *p*-cym-CH_Ar_, C-1′, C-3′–C-5′), 75.3, 74.4, 73.5 (3 × Ph*CH*_2_), 68.4 (C-6′), 53.0 (C-2′), 31.1 (*i*-Pr-*C*H), 23.4, 21.7 (2 × *i*-Pr-*C*H_3_), 17.6 (C_6_H_4_-*C*H_3_). ESI-HRMS positive mode (*m*/*z*): calcd for C_41_H_47_ClN_3_O_4_Os^+^ [M-PF_6_]^+^ 872.2861. Found: 872.2867.

Complex **Ru-5a**

Prepared from compound **5a** (26 mg, 0.094 mmol, 2.3 eq.), **Ru-dimer** (25 mg, 0.041 mmol) and TlPF_6_ (62 mg, 0.176 mmol, 4.3 eq.) according to general procedure VII. Column chromatographic purification (95:5 CHCl_3_-MeOH) yielded 23 mg (43%) of a yellow solid. R_f_ = 0.50 (7:3 CHCl_3_-MeOH). ^1^H NMR (400 MHz, CD_3_OD) δ (ppm); 9.09 (1H, ddd, *J* = 5.8, 1.4, 0.8 Hz, H-6), 8.07–8.00 (2H, m, H-3, H-4), 7.53–4.49 (1H, ddd, *J* = 7.8, 5.8, 2.0 Hz, H-5), 5.96, 5.94, 5.81, 5.64 (4 × 1H, 4 d, *J* = 6.0 Hz in each, 4 × *p*-cym-CH_Ar_), 5.70 (1H, dd, *J* = 11.8, 5.6 Hz, NH_2_), 4.41 (1H, d, *J* = 10.1 Hz, H-1′), 4.01 (1H, dd, *J* = 12.2, 2.1 Hz, H-6′a), 3.87–3.79 (1H, broad signal, NH_2_), 3.84 (1H, pt, *J* = 9.6, 8.9 Hz, H-3′), 3.82 (1H, dd, *J* = 12.2, 5.6 Hz, H-6′b), 3.67 (1H, ddd, *J* = 9.5, 5.6, 2.1 Hz, H-5′), 3.39 (1H, pt, *J* = 9.3, 9.2 Hz, H-4′), 2.83 (1H, hept, *J* = 6.9 Hz, *i*-Pr-C*H*), 2.22–2.18 (1H, m, H-2), 1.91 (3H, s, C*H*_3_), 1.28, 1.23 (2 × 3H, 2 d, *J* = 6.9 Hz in both, 2 × *i*-Pr-C*H*_3_); ^13^C NMR (100 MHz, CD_3_OD) δ (ppm): 161.8 (C-2), 158.1 (C-6), 140.9 (C-4), 125.8, 123.6 (C-3, C-5), 106.0, 100.8 (2 × *p*-cym-C_qAr_), 87.8, 85.2, 84.1, 83.4, 81.8, 80.2, 77.5, 71.3 (4 × *p*-cym-CH_Ar_, C-1′, C-3′–C-5′), 62.6 (C-6′), 56.1 (C-2′), 32.3 (*i*-Pr-*C*H), 23.3, 21.9 (2 × *i*-Pr-*C*H_3_), 18.0 (C_6_H_4_-*C*H_3_). ESI-HRMS positive mode (*m*/*z*): calcd for C_21_H_30_ClN_2_O_4_Ru^+^ [M-PF_6_]^+^ 511.0935. Found: 511.0934.

Complex **Ru-7a**

Prepared from compound **7a** (18.0 mg, 0.033 mmol, 2 eq.), **Ru-dimer** (10.0 mg, 0.016 mmol) and TlPF_6_ (11.4 mg, 0.033 mmol) according to general procedure VII. The crude product was dissolved in CHCl_3_ (3 mL), and diisopropyl ether (12 mL) was added. The precipitation was filtered off, then washed with CHCl_3_-diisopropyl ether (1:2, 1 mL) to yield 25.5 mg (81%) of a yellow solid. R_f_ = 0.49 (95:5 CHCl_3_-MeOH). ^1^H NMR (400 MHz, CDCl_3_) δ (ppm): 9.02 (1H, d, *J* = 6.1 Hz, H-6), 8.12–7.31 (18H, m, Ar, H-3, H-4, H-5), 6.68–6.59 (1H, broad signal, NH_2_), 6.15 (1H, d, *J* = 5.9 Hz, *p*-cym-CH_Ar_), 6.10 (1H, pt, *J* = 9.4, 9.3 Hz, H-3′), 6.03, 6.00 (2 × 1H, 2 d, *J* = 6.0 Hz in both, 2 × *p*-cym-CH_Ar_), 5.75 (1H, pt, *J* = 9.8, 9.6 Hz, H-4′), 5.47 (1H, d, *J* = 6.0 Hz, *p*-cym-CH_Ar_), 5.01–4.97 (2H, m, H-1′, H-6′a), 4.86 (1H, ddd, *J* = 10.0, 3.4, 2.8 Hz, H-5′), 4.49 (1H, dd, *J* = 12.7, 3.4 Hz, H-6′b), 3.39 (1H, dd, *J* = 11.2, 7.3 Hz, NH_2_), 2.89 (1H, hept, *J* = 6.9 Hz, *i*-Pr-C*H*), 2.65–2.57 (1H, m, H-2′), 1.81 (3H, s, C_6_H_4_-C*H_3_*), 1.25, 1.23 (2 × 3H, 2 d, *J* = 6.9 Hz in both, 2 × *i*-Pr-C*H*_3_); ^13^C NMR (100 MHz, CDCl_3_) δ (ppm): 168.7, 166.3, 165.6 (3 × C=O), 160.2 (C-2), 156.7 (C-6), 140.0 (C-4), 134.4, 133.8, 133.3, 130.4–130.0, 129.9, 128.7–128.6, 127.8 (Ar), 125.3, 122.9 (C-3, C-5), 104.3, 100.7 (2 × *p*-cym-C_qAr_), 88.0, 84.5, 82.6, 82.3, 77.9, 77.2, 74.4, 67.6 (4 × *p*-cym-CH_Ar_, C-1′, C-3′–C-5′), 62.1 (C-6′), 54.3 (C-2′), 31.0 (*i*-Pr-*C*H), 22.8, 22.2 (2 × *i*-Pr-*C*H_3_), 17.9 (C_6_H_4_-*C*H_3_). ESI-HRMS positive mode (*m*/*z*): calcd for C_42_H_42_ClN_2_O_7_Ru^+^ [M-PF_6_]^+^ 823.1727. Found: 823.1727.

Complex **Os-7a**

Prepared from compound **7a** (14.0 mg, 0.025 mmol, 2 eq.), **Os-dimer** (10.0 mg, 0.013 mmol) and TlPF_6_ (8.7 mg, 0.025 mmol) according to general procedure VII. After purification by column chromatography (95:5 CHCl_3_-MeOH), the complex was dissolved in CHCl_3_ (2 mL), and diisopropyl ether (16 mL) was added. The precipitated product was filtered off, then washed with CHCl_3_-diisopropyl ether (1:4, 1 mL) to yield 11.5 mg (43%) of a dark purple solid. R_f_ = 0.45 (95:5 CHCl_3_-MeOH). ^1^H NMR (500 MHz, CDCl_3_) δ (ppm): 8.86 (1H, dd, *J* = 5.9, 1.6 Hz, H-6), 8.15–7.34 (18H, m, Ar, H-3, H-4, H-5), 7.24–7.16 (1H, broad signal, NH_2_), 6.52, 6.38, 6.20, 5.66 (4 × 1H, 4 d, *J* = 5.6 Hz in each, 4 × *p*-cym-CH_Ar_), 5.96 (1H, pt, *J* = 9.3, 9.1 Hz, H-3′ or H-4′), 5.78 (1H, pt, *J* = 9.7, 9.6 Hz, H-3′ or H-4′), 4.99 (1H, dd, *J* = 12.7, 2.1 Hz, H-6′a), 4.88 (1H, d, *J* = 10.1 Hz, H-1′), 4.84 (1H, ddd, *J* = 10.2, 3.3, 2.1 Hz, H-5′), 4.49 (1H, dd, *J* = 12.7, 3.3 Hz, H-6′b), 4.24 (1H, dd, *J* = 11.6, 7.2 Hz, NH_2_), 2.87–2.78 (2H, m, H-2′, *i*-Pr-C*H*), 1.85 (3H, s, C_6_H_4_-C*H_3_*), 1.27, 1.26 (2 × 3H, 2 d, *J* = 6.9 Hz in both, 2 × *i*-Pr-C*H*_3_); ^13^C NMR (100 MHz, CDCl_3_) δ (ppm): 168.8, 166.3, 165.6 (3 × C=O), 159.6 (C-2), 156.8 (C-6), 140.2 (C-4), 134.5, 133.8, 133.3, 130.4–130.0, 129.9, 128.8–128.6, 127.7 (Ar), 125.5, 122.4 (C-3, C-5), 94.3, 92.2 (2 × *p*-cym-C_qAr_), 80.6, 79.0, 77.1, 76.2, 74.3, 73.2, 73.1, 67.3 (4 × *p*-cym-CH_Ar_, C-1′, C-3′–C-5′), 62.0 (C-6′), 54.2 (C-2′), 31.2 (*i*-Pr-*C*H), 23.1, 22.6 (2 × *i*-Pr-*C*H_3_), 17.8 (C_6_H_4_-*C*H_3_). ESI-HRMS positive mode (*m*/*z*): calcd for C_42_H_42_ClN_2_O_7_Os^+^ [M-PF_6_]^+^ 913.2292. Found: 913.2288.

Complex **Ir-7a**

Prepared from compound **7a** (13.9 mg, 0.025 mmol, 2 eq.), **Ir-dimer** (10.0 mg, 0.013 mmol) and TlPF_6_ (8.8 mg, 0.025 mmol) according to general procedure VII. The crude product was dissolved in CHCl_3_ (3 mL), and diisopropyl ether (12 mL) was added. The precipitation was filtered off, then washed with CHCl_3_-diisopropyl ether (1:1, 1 mL) to yield 20.1 mg (76%) of a yellow solid. R_f_ = 0.36 (95:5 CHCl_3_-MeOH). ^1^H NMR (400 MHz, CDCl_3_) δ (ppm): 8.67 (1H, ddd, *J* = 5.8, 1.6, 0.7 Hz, H-6), 8.14–7.36 (18H, m, Ar, H-3, H-4, H-5), 6.08 (1H, dd, *J* = 11.9, 6.0 Hz, NH_2_), 5.82 (1H, pt, *J* = 9.9, 9.7 Hz, H-4′), 5.69 (1H, pt, *J* = 9.6, 9.5 Hz, H-3′), 5.08 (1H, dd, *J* = 12.8, 2.2 Hz, H-6′a), 4.78 (1H, ddd, *J* = 10.0, 3.2, 2.2 Hz, H-5′), 4.53 (1H, dd, *J* = 12.8, 3.2 Hz, H-6′b), 4.49 (1H, d, *J* = 10.5 Hz, H-1′), 4.22 (1H, dd, *J* = 11.9, 8.1 Hz, NH_2_), 3.09–3.00 (1H, m, H-2′), 1.76 (15H, s, Cp*-C*H*_3_); ^13^C NMR (100 MHz, acetone-d_6_) δ (ppm): 167.9, 166.5, 166.0 (3 × C=O), 158.9 (C-2), 156.3 (C-6), 141.4 (C-4), 134.7, 134.6, 134.2, 130.8, 130.7–130.4, 130.0, 129.9, 129.5–129.4 (Ar), 127.2, 123.4 (C-3, C-5), 89.0 (Cp*), 81.8, 76.4, 76.3, 70.1 (C-1′, C-3′–C-5′), 63.3 (C-6′), 54.8 (C-2′), 9.2 (Cp*-*C*H_3_). ESI-HRMS positive mode (*m*/*z*): calcd for C_42_H_43_ClN_2_O_7_Ir^+^ [M-PF_6_]^+^ 915.2377. Found: 915.2381.

Complex **Rh-7a**

Prepared from compound **7a** (17.9 mg, 0.032 mmol, 2 eq.), **Rh-dimer** (10.0 mg, 0.016 mmol) and TlPF_6_ (11.3 mg, 0.032 mmol) according to general procedure VII. The crude product was dissolved in CHCl_3_ (3 mL), and diisopropyl ether (12 mL) was added. The precipitation was filtered off, then washed with CHCl_3_-diisopropyl ether (1:1, 0.5 mL) to yield 27.1 mg (86%) of an orange solid. R_f_ = 0.32 (95:5 CHCl_3_-MeOH). ^1^H NMR (400 MHz, CDCl_3_) δ (ppm): 8.68 (1H, d, *J* = 5.3 Hz, H-6), 8.13–7.43 (18H, m, Ar, H-3, H-4, H-5), 5.81 (1H, pt, *J* = 9.8, 9.7 Hz, H-3′ or H-4′), 5.66 (1H, pt, *J* = 9.8, 9.6 Hz, H-3′ or H-4′), 5.15 (1H, dd, *J* = 11.0, 5.0 Hz, NH_2_), 5.06 (1H, dd, *J* = 12.9, 2.2 Hz, H-6′a), 4.72 (1H, ddd, *J* = 10.2, 3.1, 2.2 Hz, H-5′), 4.53 (1H, d, *J* = 10.0 Hz, H-1′), 4.51 (1H, dd, *J* = 12.9, 3.1 Hz, H-6′b), 3.51 (1H, pt, *J* = 10.2, 9.8 Hz, NH_2_), 2.88–2.80 (1H, m, H-2′), 1.76 (15H, s, Cp*-C*H*_3_); ^13^C NMR (100 MHz, CDCl_3_) δ (ppm): 169.3, 166.2, 165.6 (3 × C=O), 158.0 (C-2), 153.8 (C-6), 140.4 (C-4), 134.6, 133.8, 133.3, 130.5–130.0, 129.9, 128.8–128.7, 128.6, 127.6 (Ar), 126.0, 123.4 (C-3, C-5), 97.4, 97.3 (Cp*), 79.1, 78.3, 75.3, 67.2 (C-1′, C-3′–C-5′), 62.0 (C-6′), 55.0 (C-2′), 9.3 (Cp*-*C*H_3_). ESI-HRMS positive mode (*m*/*z*): calcd for C_42_H_43_ClN_2_O_7_Rh^+^ [M-PF_6_]^+^ 825.1808. Found: 825.1807.

Complex **Ru-7b**

Prepared from compound **7b** (18.5 mg, 0.033 mmol, 2.05 eq.), **Ru-dimer** (10.0 mg, 0.016 mmol) and TlPF_6_ (11.4 mg, 0.033 mmol) according to general procedure VII. The crude product was dissolved in CHCl_3_ (3 mL), and diisopropyl ether (12 mL) was added. The precipitation was filtered off, then washed with CHCl_3_-diisopropyl ether (1:2, 2 mL) to yield 28.0 mg (88%) of a brownish–orange solid. R_f_ = 0.51 (95:5 CHCl_3_-MeOH). Diastereomeric ratio = 2:1. ^1^H NMR (400 MHz, CDCl_3_) δ (ppm): 9.18 (dd, *J* = 4.9, 2.0 Hz, major H-6), 9.08 (dd, *J* = 4.8, 2.0 Hz, minor H-6), 8.21 (dd, *J* = 8.5, 2.0 Hz, major H-4), 8.19 (dd, *J* = 8.4, 2.0 Hz, minor H-4), 8.08–7.25 (m, minor and major Ar), 7.79 (dd, *J* = 8.5, 4.9 Hz, minor H-5), 7.77 (dd, *J* = 8.5, 4.9 Hz, major H-5), 6.33 (pt, *J* = 10.6 Hz, major NH_2_), 6.09 (pt, *J* = 9.2, 9.0 Hz, major H-3′), 6.06 (pt, *J* = 9.5, 9.1 Hz, minor H-3′), 6.03–5.96 (broad signal, minor NH_2_), 6.00, 5.94, 5.76, 5.42 (4 d, *J* = 6.0 Hz in each, 4 × major *p*-cym-CH_Ar_), 5.82, 5.80, 5.77, 5.69 (4 d, *J* = 6.0 Hz in each, 4 × minor *p*-cym-CH_Ar_), 5.65 (pt, *J* = 9.9, 9.6 Hz, major H-4′), 5.57 (pt, *J* = 9.2, 9.1 Hz, minor H-4′), 5.23 (d, *J* = 10.3 Hz, major H-1′), 5.18 (d, *J* = 10.5 Hz, minor H-1′), 4.97 (dd, *J* = 12.7, 2.2 Hz, major H-6′a), 4.72–4.68 (m, minor H-6′a or H-6′b), 4.68 (ddd, *J* = 10.1, 3.4, 2.2 Hz, major H-5′), 4.49–4.44 (m, minor H-6′a or H-6′b), 4.46 (dd, *J* = 12.7, 3.4 Hz, major H-6′b), 4.44–4.39 (m, minor H-5′), 4.13 (dd, *J* = 11.3, 4.9 Hz, major NH_2_), 3.79 (pt, *J* = 11.9 Hz, minor NH_2_), 3.22–3.15 (m, minor H-2′), 3.01 (hept, *J* = 6.9 Hz, minor *i*-Pr-C*H*), 2.93–2.85 (m, major H-2′), 2.80 (hept, *J* = 6.9 Hz, major *i*-Pr-C*H*), 2.28 (s, minor C_6_H_4_-C*H_3_*), 1.85 (s, major C_6_H_4_-C*H_3_*), 1.28, 1.27 (2 d, *J* = 6.9 Hz in both, 2 × minor *i*-Pr-C*H*_3_), 1.22, 1.17 (2 d, *J* = 6.9 Hz in both, 2 × major *i*-Pr-C*H*_3_); ^13^C NMR (100 MHz, CDCl_3_) δ (ppm): 167.7, 166.4, 165.4, 165.3 (3 × major C=O, major C-3), 166.9, 166.3, 165.3, 164.1 (3 × minor C=O, minor C-3), 152.3 (major C-6), 151.1 (minor C-6), 134.1, 133.9, 133.8, 133.7, 133.5 (2), 130.3–127.8 (minor and major Ar, C-4, C-5), 105.7, 100.5 (2 × minor *p*-cym-C_qAr_), 104.9, 100.5 (2 × major *p*-cym-C_qAr_), 88.5, 85.7, 85.5, 83.2 (4 × major *p*-cym-CH_Ar_), 86.6, 86.5, 86.0, 83.3 (4 × minor *p*-cym-CH_Ar_), 77.4, 75.0, 74.4, 67.7 (major C-1′, C-3′–C-5′), 76.3, 75.7, 73.3, 69.2 (minor C-1′, C-3′–C-5′), 62.7 (minor C-6′), 61.9 (major C-6′), 53.6 (minor C-2′), 53.6 (major C-2′), 30.9 (minor *i*-Pr-*C*H), 30.8 (major *i*-Pr-*C*H), 22.9, 21.8 (2 × major *i*-Pr-*C*H_3_), 22.5, 22.3 (2 × minor *i*-Pr-*C*H_3_), 18.2 (minor C_6_H_4_-*C*H_3_), 17.9 (major C_6_H_4_-*C*H_3_); ESI-HRMS positive mode (*m*/*z*): calcd for C_41_H_41_ClN_3_O_7_Ru^+^ [M-PF_6_]^+^ 824.1679. Found: 824.1674.

Complex **Os-7b**

Prepared from compound **7b** (14.2 mg, 0.026 mmol, 2.05 eq.), **Os-dimer** (10.0 mg, 0.0126 mmol) and TlPF_6_ (8.7 mg, 0.025 mmol) according to general procedure VII. The crude product was dissolved in CHCl_3_ (3 mL), and diisopropyl ether (6 mL) was added. The precipitation was filtered off, then washed with CHCl_3_-diisopropyl ether (1:2, 3 mL) to yield 19.6 mg (73%) of a dark green solid. R_f_ = 0.38 (95:5 CHCl_3_-MeOH). Diastereomeric ratio = 5:4. ^1^H NMR (400 MHz, CDCl_3_) δ (ppm): 9.08 (dd, *J* = 4.9, 2.0 Hz, major H-6), 8.93 (dd, *J* = 4.8, 2.0 Hz, minor H-6), 8.30 (dd, *J* = 8.7, 2.0 Hz, minor H-4), 8.24 (dd, *J* = 8.6, 2.0 Hz, major H-4), 8.12–7.31 (m, minor and major Ar), 7.85 (dd, *J* = 8.7, 4.8 Hz, minor H-5), 7.69 (dd, *J* = 8.6, 4.9 Hz, major H-5), 6.89 (pt, *J* = 11.0 Hz, major NH_2_), 6.72–6.65 (broad signal, minor NH_2_), 6.27, 6.22, 6.19, 5.70 (4 d, *J* = 5.8 Hz in each, 4 × major *p*-cym-CH_Ar_), 6.08, 6.04, 6.03, 5.92 (4 d, *J* = 5.7 Hz in each, 4 × minor *p*-cym-CH_Ar_), 5.94 (pt, *J* = 9.0, 9.0 Hz, major H-3′), 5.88 (pt, *J* = 9.2, 8.9 Hz, minor H-3′), 5.73 (pt, *J* = 9.9, 9.8 Hz, major H-4′), 5.63 (pt, *J* = 9.4, 9.3 Hz, minor H-4′), 5.49 (d, *J* = 10.5 Hz, minor H-1′), 5.12 (d, *J* = 10.3 Hz, major H-1′), 4.98 (dd, *J* = 12.7, 2.2 Hz, major H-6′a), 4.73 (dd, *J* = 12.5, 2.5 Hz, minor H-6′a), 4.70 (ddd, *J* = 10.1, 3.4, 2.2 Hz, major H-5′), 4.64 (dd, *J* = 11.7, 4.9 Hz, major NH_2_), 4.52–4.44 (m, minor and major H-6′b), 4.44–4.34 (m, minor H-5′, NH_2_), 3.38–3.27 (m, minor H-2′), 3.13–3.05 (m, major H-2′), 2.88 (hept, *J* = 6.9 Hz, minor *i*-Pr-C*H*), 2.77 (hept, *J* = 6.9 Hz, major *i*-Pr-C*H*), 2.26 (s, minor C_6_H_4_-C*H_3_*), 1.93 (s, major C_6_H_4_-C*H_3_*), 1.26–1.20 (m, 2 × minor and major *i*-Pr-C*H*_3_); ^13^C NMR (100 MHz, CDCl_3_) δ (ppm): 167.9, 167.0, 166.4, 166.3, 165.4, 165.3, 164.4, 163.6 (3 × minor and major C=O, minor and major C-3), 152.7 (major C-6), 152.0 (minor C-6), 134.2, 133.9, 133.8, 133.7, 133.5, 133.4, 130.9–127.9 (minor and major Ar, C-4, C-5), 96.5, 94.2 (2 × minor *p*-cym-C_qAr_), 94.9, 92.5 (2 × major *p*-cym-C_qAr_), 80.3, 79.8 (2), 78.3 (2), 77.0, 76.5, 75.2 (2), 75.9, 75.8, 74.3, 73.6, 73.3, 69.0, 67.4 (4 × minor and major *p*-cym-CH_Ar_, minor and major C-1′, C-3′–C-5′), 62.6 (minor C-6′), 61.8 (major C-6′), 54.5 (minor C-2′), 53.5 (major C-2′), 31.0 (minor *i*-Pr-*C*H), 30.9 (major *i*-Pr-*C*H), 22.4, 21.9 (2 × major *i*-Pr-*C*H_3_), 22.7, 22.6 (2 × minor *i*-Pr-*C*H_3_), 18.2 (minor C_6_H_4_-*C*H_3_), 17.9 (major C_6_H_4_-*C*H_3_). ESI-HRMS positive mode (*m*/*z*): calcd for C_41_H_41_ClN_3_O_7_Os^+^ [M-PF_6_]^+^ 914.2234. Found: 914.2239.

Complex **Ir-7b**

Prepared from compound **7b** (14.2 mg, 0.026 mmol, 2.05 eq.), **Ir-dimer** (10.0 mg, 0.013 mmol) and TlPF_6_ (8.8 mg, 0.025 mmol) according to general procedure VII. The crude product was dissolved in CHCl_3_ (3 mL), and diisopropyl ether (6 mL) was added. The precipitation was filtered off, then washed with CHCl_3_-diisopropyl ether (1:1, 4 mL) to yield 18.5 mg (69%) of a yellow solid. R_f_ = 0.33 (95:5 CHCl_3_-MeOH). Diastereomeric ratio = 9:1. ^1^H NMR (400 MHz, CDCl_3_) δ (ppm): 9.09 (dd, *J* = 4.9, 2.0 Hz, major H-6), 8.96 (dd, *J* = 4.8, 2.0 Hz, minor H-6), 8.34 (dd, *J* = 8.7, 2.0 Hz, major H-4), 8.29 (dd, *J* = 8.7, 2.0 Hz, minor H-4), 8.12–7.34 (m, minor and major Ar), 7.86 (dd, *J* = 8.7, 4.8 Hz, minor H-5), 7.78 (dd, *J* = 8.7, 4.9 Hz, major H-5), 5.96 (d, *J* = 10.2 Hz, minor H-1′), 5.87 (pt, *J* = 9.1, 9.0 Hz, major H-3′ or H-4′), 5.81 (pt, *J* = 9.8, 9.3 Hz, minor H-3′ or H-4′), 5.78 (pt, *J* = 9.9, 9.5 Hz, major H-3′ or H-4′), 5.66 (pt, *J* = 9.2, 9.1 Hz, minor H-3′ or H-4′), 5.59 (pt, *J* = 10.6 Hz, major NH_2_), 5.36–5.29 (m, minor NH_2_), 5.12–5.05 (m, minor NH_2_), 4.99 (dd, *J* = 12.7, 2.2 Hz, major H-6′a), 4.89 (d, *J* = 10.3 Hz, major H-1′), 4.75 (ddd, *J* = 10.3, 3.6, 2.2 Hz, major H-5′), 4.77–4.66 (m, major NH_2_, minor H-6′a), 4.54 (dd, *J* = 12.5, 5.2 Hz, minor H-6′b), 4.48 (dd, *J* = 12.7, 3.6 Hz, major H-6′b), 4.29 (ddd, *J* = 10.0, 5.2, 2.7 Hz, minor H-5′), 3.75–3.67 (m, minor H-2′), 3.44–3.35 (m, major H-2′), 1.69 (s, major Cp*-C*H*_3_), 1.57 (s, minor Cp*-C*H*_3_); ^13^C NMR (100 MHz, CDCl_3_) δ (ppm); only the major isomer can be clearly assigned: 168.4, 166.4, 165.5, 163.7 (3 × C=O, C-3), 153.5 (C-6), 134.3, 133.8, 133.4, 130.5–130.0, 129.8, 129.6–127.6, 127.9 (Ar, C-4, C-5), 89.3 (Cp*), 78.0, 76.4, 74.6, 67.3 (C-1′, C-3′–C-5′), 61.9 (C-6′), 54.3 (C-2′), 8.8 (Cp*-*C*H_3_). ESI-HRMS positive mode (*m*/*z*): calcd for C_41_H_42_ClN_3_O_7_Ir^+^ [M-PF_6_]^+^ 916.2329. Found: 916.2322.

Complex **Rh-7b**

Prepared from compound **7b** (9.2 mg, 0.017 mmol, 2.05 eq.), **Rh-dimer** (5.0 mg, 0.008 mmol) and TlPF_6_ (5.6 mg, 0.016 mmol) according to general procedure VII. The crude product was dissolved in CHCl_3_ (1.5 mL), and diisopropyl ether (3 mL) was added. The precipitation was filtered off, then washed with CHCl_3_-diisopropyl ether (1:1, 2 mL) to yield 13.5 mg (86%) of an orange solid. R_f_ = 0.38 (95:5 CHCl_3_-MeOH). Diastereomeric ratio = 5:1. ^1^H NMR (400 MHz, CDCl_3_) δ (ppm): 9.17 (dd, *J* = 4.9, 1.9 Hz, major H-6), 9.10 (dd, *J* = 4.8, 1.9 Hz, minor H-6), 8.28 (dd, *J* = 8.7, 1.9 Hz, major H-4), 8.17 (dd, *J* = 8.7, 1.9 Hz, minor H-4), 8.12–7.32 (m, minor and major Ar), 7.80 (dd, *J* = 8.7, 4.8 Hz, minor H-5), 7.77 (dd, *J* = 8.7, 4.9 Hz, major H-5), 6.01 (d, *J* = 10.0 Hz, minor H-1′), 5.89 (pt, *J* = 9.2, 9.1 Hz, major H-3′ or H-4′), 5.79 (pt, *J* = 9.7, 9.6 Hz, minor H-3′ or H-4′), 5.74 (pt, *J* = 9.8, 9.6 Hz, major H-3′ or H-4′), 5.61 (pt, *J* = 9.6, 9.4 Hz, minor H-3′ or H-4′), 5.07 (d, *J* = 10.3 Hz, major H-1′), 4.95 (dd, *J* = 12.7, 2.2 Hz, major H-6′a), 4.92–4.87 (broad signal, minor NH_2_), 4.81 (pt, *J* = 10.6 Hz, major NH_2_), 4.73 (ddd, *J* = 10.2, 3.7, 2.2 Hz, major H-5′), 4.63 (dd, *J* = 12.4, 2.6 Hz, minor H-6′a), 4.50 (dd, *J* = 12.4, 5.4 Hz, minor H-6′b), 4.48 (dd, *J* = 12.7, 3.7 Hz, major H-6′b), 4.37 (pt, *J* = 9.1 Hz, minor NH_2_), 4.25 (dd, *J* = 10.6, 4.4 Hz, major NH_2_), 4.21 (ddd, *J* = 10.1, 5.4, 2.6 Hz, minor H-5′), 3.57–3.47 (m, minor H-2′), 3.30–3.21 (m, major H-2′), 1.73 (s, major Cp*-C*H*_3_); 1.65 (s, minor Cp*-C*H*_3_); ^13^C NMR (90 MHz, CDCl_3_) δ (ppm): 170.0, 165.1, 164.5, 162.7 (3 × minor C=O, minor C-3), 168.3, 166.4, 165.5, 164.5 (3 × major C=O, major C-3), 152.8 (major C-6), 151.4 (minor C-6), 134.5, 134.2, 133.7 (2), 133.4, 133.3, 130.5–128.1 (minor and major C-4, C-5, Ar), 98.0, 97.9 (minor Cp*), 97.5, 97.4 (major Cp*), 79.1, 76.4, 74.3, 68.9 (minor C-1′, C-3′–C-5′), 78.9, 75.5, 74.6, 67.5 (major C-1′, C-3′–C-5′), 63.0 (minor C-6′), 62.0 (major C-6′), 54.8 (minor C-2′), 52.6 (major C-2′), 9.0 (major Cp*-*C*H_3_), 8.7 (minor Cp*-*C*H_3_). ESI-HRMS positive mode (*m*/*z*): calcd for C_41_H_42_ClN_3_O_7_Rh^+^ [M-PF_6_]^+^ 826.1761. Found: 826.1757.

Complex **Ru-7c**

Prepared from compound **7c** (18.5 mg, 0.033 mmol, 2.05 eq.), **Ru-dimer** (10.0 mg, 0.016 mmol) and TlPF_6_ (11.4 mg, 0.033 mmol) according to general procedure VII. The crude product was dissolved in CHCl_3_ (3 mL), and diisopropyl ether (12 mL) was added. The precipitation was filtered off, then washed with CHCl_3_-diisopropyl ether (1:2, 2 mL) to yield 31.2 mg (99%) of a brownish–orange solid. R_f_ = 0.44 (95:5 CHCl_3_-MeOH). ^1^H NMR (400 MHz, CDCl_3_) δ (ppm): 9.33, 8.93 (2 ×1H, 2 dd, *J* = 5.8, 2.2 Hz and 4.7, 2.2 Hz, respectively, H-4, H-6), 8.02–7.21 (16H, m, Ar, H-5), 6.19 (1H, dd, *J* = 11.6, 8.3 Hz, NH_2_), 6.11 (1H, pt, *J* = 9.2, 9.2 Hz, H-3′ or H-4′), 6.01, 5.91, 5.89, 5.40 (4 × 1H, 4 d, *J* = 6.0 Hz in each, 4 × *p*-cym-CH_Ar_), 5.71 (1H, pt, *J* = 9.7, 9.6 Hz, H-3′ or H-4′), 5.11 (1H, d, *J* = 10.3 Hz, H-1′), 4.82 (1H, dd, *J* = 12.7, 2.4 Hz, H-6′a), 4.73 (1H, m, H-5′), 4.60 (1H, dd, *J* = 12.7, 3.6 Hz, H-6′b), 3.67 (1H, dd, *J* = 11.6, 6.2 Hz, NH_2_), 2.94–2.86 (1H, m, H-2′), 2.75 (1H, hept, *J* = 6.9 Hz, *i*-Pr-C*H*), 1.72 (3H, s, C_6_H_4_-C*H_3_*), 1.23, 1.09 (2 × 3H, 2 d, *J* = 6.9 Hz in both, 2 × *i*-Pr-C*H*_3_); ^13^C NMR (90 MHz, CDCl_3_) δ (ppm): 168.5, 166.4, 166.2, 165.2 (3 × C=O, C-2), 165.1 (C-6), 159.4 (C-4), 134.4, 133.8, 133.2, 130.3–128.5, 127.4 (Ar), 122.1 (C-5), 105.4, 100.8 (2 × *p*-cym-C_qAr_), 86.2, 84.2, 83.5, 82.6 (4 × *p*-cym-CH_Ar_), 78.0, 77.4, 74.3, 68.2 (C-1′, C-3′–C-5′), 62.7 (C-6′), 54.2 (C-2′), 31.0 (*i*-Pr-*C*H), 23.0, 21.8 (2 × *i*-Pr-*C*H_3_), 18.0 (C_6_H_4_-*C*H_3_). ESI-HRMS positive mode (*m*/*z*): calcd for C_41_H_41_ClN_3_O_7_Ru^+^ [M-PF_6_]^+^ 824.1679. Found: 824.1681.

Complex **Os-7c**

Prepared from compound **7c** (14.2 mg, 0.026 mmol, 2.05 eq.), **Os-dimer** (10.0 mg, 0.013 mmol) and TlPF_6_ (8.7 mg, 0.025 mmol) according to general procedure VII. The crude product was dissolved in CHCl_3_ (3 mL), and diisopropyl ether (6 mL) was added. The precipitation was filtered off, then washed with CHCl_3_-diisopropyl ether (1:1, 2 mL) to yield 26.3 mg (98%) of a dark green solid. R_f_ = 0.27 (95:5 CHCl_3_-MeOH). ^1^H NMR (400 MHz, CDCl_3_) δ (ppm): 9.21, 8.86 (2 ×1H, 2 dd, *J* = 5.9, 2.2 Hz and 4.7, 2.2 Hz, respectively, H-4, H-6), 8.03–7.21 (16H, m, Ar, H-5), 6.86 (1H, dd, *J* = 12.0, 8.5 Hz, NH_2_), 6.23, 6.18, 6.14, 5.57 (4 × 1H, 4 d, *J* = 5.7 Hz in each, 4 × *p*-cym-CH_Ar_), 6.07 (1H, pt, *J* = 9.2, 9.2 Hz, H-3′ or H-4′), 5.74 (1H, pt, *J* = 9.8, 9.6 Hz, H-3′ or H-4′), 5.03 (1H, d, *J* = 10.3 Hz, H-1′), 4.82 (1H, dd, *J* = 12.7, 2.4 Hz, H-6′a), 4.71 (1H, ddd, *J* = 10.2, 3.5, 2.4 Hz, H-5′), 4.60 (1H, dd, *J* = 12.7, 3.5 Hz, H-6′b), 4.43 (1H, dd, *J* = 12.0, 6.0 Hz, NH_2_), 3.19–3.10 (1H, m, H-2′), 2.68 (1H, hept, *J* = 6.9 Hz, *i*-Pr-C*H*), 1.75 (3H, s, C_6_H_4_-C*H_3_*), 1.23, 1.06 (2 × 3H, 2 d, *J* = 6.9 Hz in both, 2 × *i*-Pr-C*H*_3_); ^13^C NMR (100 MHz, CDCl_3_) δ (ppm): 168.4, 166.2, 165.6, 165.2 (3 × C=O, C-2), 165.5 (C-6), 159.2 (C-4), 134.5, 133.9, 133.2, 130.3–130.0, 129.8, 128.8–128.5, 127.3 (Ar), 122.4 (C-5), 96.0, 92.5 (2 × *p*-cym-C_qAr_), 78.3, 77.9, 77.0, 75.8, 74.5, 74.2, 73.5, 68.0 (4 × *p*-cym-CH_Ar_, C-1′, C-3′–C-5′), 62.6 (C-6′), 53.9 (C-2′), 31.1 (*i*-Pr-*C*H), 23.4, 21.9 (2 × *i*-Pr-*C*H_3_), 17.9 (C_6_H_4_-*C*H_3_). ESI-HRMS positive mode (*m*/*z*): calcd for C_41_H_41_ClN_3_O_7_Os^+^ [M-PF_6_]^+^ 914.2234. Found: 914.2234.

Complex **Ir-7c**

Prepared from compound **7c** (14.2 mg, 0.026 mmol, 2.05 eq.), **Ir-dimer** (10.0 mg, 0.013 mmol) and TlPF_6_ (8.8 mg, 0.025 mmol) according to general procedure VII. The crude product was dissolved in CHCl_3_ (3 mL), and diisopropyl ether (6 mL) was added. The precipitation was filtered off, then washed with CHCl_3_-diisopropyl ether (1:1, 4 mL) to yield 26.4 mg (99%) of a yellow solid. R_f_ = 0.30 (95:5 CHCl_3_-MeOH). ^1^H NMR (400 MHz, CDCl_3_) δ (ppm): 8.98, 8.84 (2 ×1H, 2 dd, *J* = 4.8, 2.2 Hz and 5.9, 2.2 Hz, respectively, H-4, H-6), 8.01–7.29 (16H, m, Ar, H-5), 5.94 (1H, dd, *J* = 11.8, 5.2 Hz, NH_2_), 5.83 (1H, pt, *J* = 9.6, 9.6 Hz, H-3′ or H-4′), 5.66 (1H, pt, *J* = 9.5, 9.6 Hz, H-3′ or H-4′), 4.81–4.56 (4H, m, H-1′, H-5′, H-6′a,b), 4.29 (1H, dd, *J* = 11.8, 8.2 Hz, NH_2_), 3.37–3.29 (1H, m, H-2′), 1.74 (15H, s, Cp*-C*H*_3_); ^13^C NMR (90 MHz, CDCl_3_) δ (ppm): 169.3, 166.3, 165.5, 164.1 (3 × C=O, C-2), 162.3, 160.5 (C-4, C-6), 134.8, 133.8, 133.2, 130.6–130.0, 129.8, 128.9–128.5, 127.5 (Ar), 123.2 (C-5), 89.4 (Cp*), 80.5, 77.9, 75.2, 67.7 (C-1′, C-3′–C-5′), 62.8 (C-6′), 54.9 (C-2′), 9.1 (Cp*-*C*H_3_). ESI-HRMS positive mode (*m*/*z*): calcd for C_41_H_42_ClN_3_O_7_Ir^+^ [M-PF_6_]^+^ 916.2329. Found: 916.2324.

Complex **Rh-7c**

Prepared from compound **7c** (9.2 mg, 0.017 mmol, 2.05 eq.), **Rh-dimer** (5.0 mg, 0.008 mmol) and TlPF_6_ (5.6 mg, 0.016 mmol) according to general procedure VII. The crude product was dissolved in CHCl_3_ (1.5 mL), and diisopropyl ether (3 mL) was added. The precipitation was filtered off, then washed with CHCl_3_-diisopropyl ether (1:1, 4 mL) to yield 15.5 mg (99%) of an orange solid. R_f_ = 0.24 (95:5 CHCl_3_-MeOH). ^1^H NMR (400 MHz, CDCl_3_) δ (ppm): 9.02, 8.92 (2 ×1H, 2 dd, *J* = 4.9, 2.3 Hz and 5.7, 2.3 Hz, respectively, H-4, H-6), 8.03–7.29 (16H, m, Ar, H-5), 5.84 (1H, pt, *J* = 9.6, 9.6 Hz, H-3′ or H-4′), 5.67 (1H, pt, *J* = 9.7, 9.6 Hz, H-3′ or H-4′), 5.15 (1H, dd, *J* = 12.1, 3.7 Hz, NH_2_), 4.85–4.58 (3H, m, H-5′, H-6′a,b), 4.72 (1H, d, *J* = 10.2 Hz, H-1′), 3.46 (1H, pt, *J* = 10.0 Hz, NH_2_), 3.21–3.13 (1H, m, H-2′), 1.81 (15H, s, Cp*-C*H*_3_); ^13^C NMR (90 MHz, CDCl_3_) δ (ppm): 169.4, 166.3, 165.5, 164.7 (3 × C=O, C-2), 161.7, 160.4 (C-4, C-6), 134.8, 133.8, 133.2, 130.6–130.0, 129.9, 128.9–128.5, 127.5 (Ar), 122.8 (C-5), 97.9, 97.8 (Cp*), 80.0, 78.4, 75.4, 67.8 (C-1′, C-3′–C-5′), 62.8 (C-6′), 55.3 (C-2′), 9.5 (Cp*-*C*H_3_). ESI-HRMS positive mode (*m*/*z*): calcd for C_41_H_42_ClN_3_O_7_Rh^+^ [M-PF_6_]^+^ 826.1761. Found: 826.1757.

Complex **Ru-7d**

Prepared from compound **7d** (18.5 mg, 0.033 mmol, 2.05 eq.), **Ru-dimer** (10.0 mg, 0.016 mmol) and TlPF_6_ (11.4 mg, 0.033 mmol) according to general procedure VII. The crude product was dissolved in CHCl_3_ (3 mL), and diisopropyl ether (6 mL) was added. The precipitation was filtered off, then washed with CHCl_3_-diisopropyl ether (1:2, 6 mL) to yield 28.8 mg (91%) of a brown solid. R_f_ = 0.35 (95:5 CHCl_3_-MeOH). ^1^H NMR (400 MHz, CDCl_3_) δ (ppm): 9.09 (1H, s, H-3), 9.02 (1H, dd, *J* = 3.2, 1.1 Hz, H-5), 8.76 (1H, d, *J* = 3.2 Hz, H-6), 8.08–7.26 (15H, m, Ar), 6.26 (1H, dd, *J* = 11.5, 8.3 Hz, NH_2_), 6.07 (1H, pt, *J* = 9.3, 9.2 Hz, H-3′ or H-4′), 6.01–5.96 (3H, m, 3 × *p*-cym-CH_Ar_), 5.72 (1H, pt, *J* = 9.7, 9.7 Hz, H-3′ or H-4′), 5.44 (1H, d, *J* = 6.0 Hz, *p*-cym-CH_Ar_), 5.03 (1H, d, *J* = 10.2 Hz, H-1′), 4.98 (1H, dd, *J* = 12.8, 2.2 Hz, H-6′a), 4.76 (1H, ddd, *J* = 10.2, 3.4, 2.2 Hz, H-5′), 4.48 (1H, dd, *J* = 12.8, 3.4 Hz, H-6′b), 3.54 (1H, dd, *J* = 11.5, 6.7 Hz, NH_2_), 2.81 (1H, hept, *J* = 6.9 Hz, *i*-Pr-C*H*), 2.73–2.64 (1H, m, H-2′), 1.77 (3H, s, C_6_H_4_-C*H_3_*), 1.23, 1.16 (2 × 3H, 2 d, *J* = 6.9 Hz in both, 2 × *i*-Pr-C*H*_3_); ^13^C NMR (90 MHz, CDCl_3_) δ (ppm): 168.4, 166.3, 165.4 (3 × C=O), 154.7 (C-2), 150.1, 146.3, 144.6 (C-3, C-5, C-6), 134.4, 133.9, 133.4, 130.3–130.0, 129.7, 128.8–128.6, 127.6 (Ar), 105.4, 101.0 (2 × *p*-cym-C_qAr_), 87.4, 84.9, 83.4, 83.3 (4 × *p*-cym-CH_Ar_), 77.0, 76.9, 74.6, 67.7 (C-1′, C-3′–C-5′), 61.9 (C-6′), 53.8 (C-2′), 31.0 (*i*-Pr-*C*H), 22.9, 21.9 (2 × *i*-Pr-*C*H_3_), 17.9 (C_6_H_4_-*C*H_3_). ESI-HRMS positive mode (*m*/*z*): calcd for C_41_H_41_ClN_3_O_7_Ru^+^ [M-PF_6_]^+^ 824.1679. Found: 824.1673.

Complex **Os-7d**

Prepared from compound **7d** (14.2 mg, 0.026 mmol, 2.05 eq.), **Os-dimer** (10.0 mg, 0.013 mmol) and TlPF_6_ (8.7 mg, 0.025 mmol) according to general procedure VII. The crude product was dissolved in CHCl_3_ (3 mL), and diisopropyl ether (6 mL) was added. The precipitation was filtered off, then washed with CHCl_3_-diisopropyl ether (1:2, 3 mL) to yield 24.4 mg (91%) of a brown solid. R_f_ = 0.29 (95:5 CHCl_3_-MeOH). ^1^H NMR (400 MHz, CDCl_3_) δ (ppm): 9.09 (1H, s, H-3), 8.87 (1H, dd, *J* = 3.2, 1.1 Hz, H-5), 8.69 (1H, d, *J* = 3.2 Hz, H-6), 8.09–7.28 (15H, m, Ar), 6.96 (1H, dd, *J* = 11.9, 8.4 Hz, NH_2_), 6.27–6.24 (2H, m, 2 × *p*-cym-CH_Ar_), 6.20 (1H, d, *J* = 5.7 Hz, *p*-cym-CH_Ar_), 6.03 (1H, pt, *J* = 9.2, 9.2 Hz, H-3′ or H-4′), 5.75 (1H, pt, *J* = 9.7, 9.6 Hz, H-3′ or H-4′), 5.62 (1H, d, *J* = 5.7 Hz, *p*-cym-CH_Ar_), 4.99 (1H, dd, *J* = 12.8, 2.2 Hz, H-6′a), 4.94 (1H, d, *J* = 10.2 Hz, H-1′), 4.75 (1H, ddd, *J* = 10.2, 3.4, 2.2 Hz, H-5′), 4.49 (1H, dd, *J* = 12.8, 3.4 Hz, H-6′b), 4.28 (1H, dd, *J* = 11.9, 6.4 Hz, NH_2_), 2.97–2.88 (1H, m, H-2′), 2.74 (1H, hept, *J* = 6.9 Hz, *i*-Pr-C*H*), 1.80 (3H, s, C_6_H_4_-C*H_3_*), 1.23, 1.14 (2 × 3H, 2 d, *J* = 6.9 Hz in both, 2 × *i*-Pr-C*H*_3_); ^13^C NMR (90 MHz, CDCl_3_) δ (ppm): 168.5, 166.3, 165.3 (3 × C=O), 153.6 (C-2), 150.4, 147.1, 144.3 (C-3, C-5, C-6), 134.5, 133.9, 133.4, 130.3–130.0, 129.7, 128.8–128.6, 127.5 (Ar), 95.9, 92.7 (2 × *p*-cym-C_qAr_), 79.5, 77.6, 76.7, 76.6, 74.6, 74.3, 74.1, 67.5 (4 × *p*-cym-CH_Ar_, C-1′, C-3′–C-5′), 61.8 (C-6′), 53.6 (C-2′), 31.1 (*i*-Pr-*C*H), 23.3, 22.1 (2 × *i*-Pr-*C*H_3_), 17.7 (C_6_H_4_-*C*H_3_). ESI-HRMS positive mode (*m*/*z*): calcd for C_41_H_41_ClN_3_O_7_Os^+^ [M-PF_6_]^+^ 914.2234. Found: 914.2233.

Complex **Ir-7d**

Prepared from compound **7d** (14.2 mg, 0.026 mmol, 2.05 eq.), **Ir-dimer** (10.0 mg, 0.013 mmol) and TlPF_6_ (8.8 mg, 0.025 mmol) according to general procedure VII. The crude product was dissolved in CHCl_3_ (3 mL), and diisopropyl ether (6 mL) was added. The precipitation was filtered off, then washed with CHCl_3_-diisopropyl ether (1:1, 4 mL) to yield 26.5 mg (99%) of a yellow solid. R_f_ = 0.24 (95:5 CHCl_3_-MeOH). ^1^H NMR (400 MHz, CDCl_3_) δ (ppm): 9.18 (1H, s, H-3), 8.76 (1H, d, *J* = 3.2 Hz, H-6), 8.54 (1H, dd, *J* = 3.2, 1.2 Hz, H-5), 8.10–7.32 (15H, m, Ar), 5.94 (1H, dd, *J* = 11.8, 6.4 Hz, NH_2_), 5.84 (1H, pt, *J* = 9.8, 9.7 Hz, H-3′ or H-4′), 5.68 (1H, pt, *J* = 9.6, 9.5 Hz, H-3′ or H-4′), 5.03 (1H, dd, *J* = 12.9, 2.2 Hz, H-6′a), 4.74 (1H, ddd, *J* = 10.1, 3.0, 2.2 Hz, H-5′), 4.56 (1H, d, *J* = 10.1 Hz, H-1′), 4.50 (1H, dd, *J* = 12.9, 3.0 Hz, H-6′b), 4.27 (1H, dd, *J* = 11.8, 7.9 Hz, NH_2_), 3.17–3.09 (1H, m, H-2′), 1.73 (15H, s, Cp*-C*H*_3_); ^13^C NMR (90 MHz, CDCl_3_) δ (ppm): 169.3, 166.2, 165.5 (3 × C=O), 152.1 (C-2), 147.8, 147.5, 145.5 (C-3, C-5, C-6), 134.8, 133.8, 133.3, 130.6–130.0, 129.8, 128.8–128.6, 128.5, 127.5 (Ar), 89.7 (Cp*), 78.9, 77.7, 75.4, 67.1 (C-1′, C-3′–C-5′), 61.8 (C-6′), 54.5 (C-2′), 8.9 (Cp*-*C*H_3_). ESI-HRMS positive mode (*m*/*z*): calcd for C_41_H_42_ClN_3_O_7_Ir^+^ [M-PF_6_]^+^ 916.2329. Found: 916.2331.

Complex **Rh-7d**

Prepared from compound **7d** (9.2 mg, 0.017 mmol, 2.05 eq.), **Rh-dimer** (5.0 mg, 0.008 mmol) and TlPF_6_ (5.6 mg, 0.016 mmol) according to general procedure VII. The crude product was dissolved in CHCl_3_ (1.5 mL), and diisopropyl ether (3 mL) was added. The precipitation was filtered off, then washed with CHCl_3_-diisopropyl ether (1:1, 4 mL) to yield 15.1 mg (96%) of an orange solid. R_f_ = 0.21 (95:5 CHCl_3_-MeOH). ^1^H NMR (400 MHz, CDCl_3_) δ (ppm): 9.19 (1H, s, H-3), 8.83 (1H, d, *J* = 3.1 Hz, H-6), 8.61 (1H, dd, *J* = 3.1, 1.2 Hz, H-5), 8.11–7.32 (15H, m, Ar), 5.83 (1H, pt, *J* = 9.8, 9.7 Hz, H-3′ or H-4′), 5.69 (1H, pt, *J* = 9.7, 9.6 Hz, H-3′ or H-4′), 5.13 (1H, dd, *J* = 11.4, 6.1 Hz, NH_2_), 5.05 (1H, dd, *J* = 12.9, 2.2 Hz, H-6′a), 4.75 (1H, ddd, *J* = 10.2, 2.9, 2.2 Hz, H-5′), 4.69 (1H, d, *J* = 10.1 Hz, H-1′), 4.51 (1H, dd, *J* = 12.9, 2.9 Hz, H-6′b), 3.54 (1H, pt, *J* = 9.9 Hz, NH_2_), 3.02–2.93 (1H, m, H-2′), 1.78 (15H, s, Cp*-C*H*_3_); ^13^C NMR (100 MHz, CDCl_3_) δ (ppm): 169.4, 166.2, 165.5 (3 × C=O), 152.6 (C-2), 147.1, 146.8, 145.6 (C-3, C-5, C-6), 134.8, 133.8, 133.3, 130.6–130.0, 129.8–128.7, 128.6, 127.5 (Ar), 98.1, 98.0 (Cp*), 78.3, 78.2, 75.5, 67.1 (C-1′, C-3′–C-5′), 61.7 (C-6′), 54.9 (C-2′), 9.3 (Cp*-*C*H_3_). ESI-HRMS positive mode (*m*/*z*): calcd for C_41_H_42_ClN_3_O_7_Rh^+^ [M-PF_6_]^+^ 826.1761. Found: 826.1754.

Complex **Ru-7e**

Prepared from compound **7e** (21.2 mg, 0.035 mmol, 2.15 eq.), **Ru-dimer** (10.0 mg, 0.016 mmol) and TlPF_6_ (11.4 mg, 0.033 mmol) according to general procedure VII. The crude product was dissolved in CHCl_3_ (3 mL), and diisopropyl ether (12 mL) was added. The precipitation was filtered off, then washed with CHCl_3_-diisopropyl ether (1:2, 2 mL) to yield 27.2 mg (82%) of an orange solid. R_f_ = 0.66 (95:5 CHCl_3_-MeOH). ^1^H NMR (400 MHz, CDCl_3_) δ (ppm): 9.38 (1H, d, *J* = 9.3 Hz, H-3 or H-4 or H-5 or H-8), 8.37 (1H, d, *J* = 8.7 Hz, H-3 or H-4 or H-5 or H-8), 8.16–7.34 (19H, m, Ar, H-3 and/or H-4 and/or H-5 and/or H-8, H-6, H-7), 6.58 (1H, dd, *J* = 11.4, 6.3 Hz, NH_2_), 6.22–6.18 (3H, m, 3 × *p*-cym-CH_Ar_), 6.04 (1H, pt, *J* = 9.5, 9.2 Hz, H-3′ or H-4′), 5.83 (1H, pt, *J* = 9.7, 9.7 Hz, H-3′ or H-4′), 5.51 (1H, d, *J* = 6.1 Hz, *p*-cym-CH_Ar_), 5.14 (1H, d, *J* = 10.1 Hz, H-1′), 5.05 (1H, dd, *J* = 12.7, 2.1 Hz, H-6′a), 4.93 (1H, ddd, *J* = 9.8, 3.3, 2.1 Hz, H-5′), 4.53 (1H, dd, *J* = 12.7, 3.3 Hz, H-6′b), 3.61 (1H, dd, *J* = 11.4, 7.6 Hz, NH_2_), 2.89 (1H, hept, *J* = 6.9 Hz, *i*-Pr-C*H*), 2.82–2.74 (1H, m, H-2), 1.59 (3H, s, C_6_H_4_-C*H_3_*), 1.26, 1.19 (2 × 3H, 2 d, *J* = 6.9 Hz in both, 2 × *i*-Pr-C*H*_3_); ^13^C NMR (100 MHz, CDCl_3_) δ (ppm): 168.9, 166.3, 165.7, 163.5, 149.3 (3 × C=O, C-2, C-8a), 141.3, 134.4, 133.8, 133.3, 131.4, 131.3, 130.4–130.0, 129.9, 129.3, 129.0–128.6, 127.8, 120.0 (Ar, C-3–C-8, C-4a), 104.4, 100.6 (2 × *p*-cym-C_qAr_), 87.3, 85.2, 83.3, 82.3, 79.9, 77.4, 74.6, 67.3 (4 × *p*-cym-CH_Ar_, C-1′, C-3′–C-5′), 62.1 (C-6′), 54.6 (C-2′), 31.0 (*i*-Pr-*C*H), 22.8, 21.7 (2 × *i*-Pr-*C*H_3_), 17.7 (C_6_H_4_-*C*H_3_). ESI-HRMS positive mode (*m*/*z*): calcd for C_46_H_44_ClN_2_O_7_Ru^+^ [M-PF_6_]^+^ 873.1885. Found: [M-PF_6_]^+^ 873.1876.

Complex **Os-7e**

Prepared from compound **7e** (15.6 mg, 0.026 mmol, 2.05 eq.), **Os-dimer** (10.0 mg, 0.013 mmol) and TlPF_6_ (8.7 mg, 0.025 mmol) according to general procedure VII. The crude product was dissolved in CHCl_3_ (1 mL), and diisopropyl ether (8 mL) was added. The precipitation was filtered off, then washed with CHCl_3_-diisopropyl ether (1:4, 2 mL) to yield 26.9 mg (96%) of a dark green solid. R_f_ = 0.58 (95:5 CHCl_3_-MeOH). ^1^H NMR (400 MHz, CDCl_3_) δ (ppm): 9.18 (1H, d, *J* = 9.1 Hz, H-3 or H-4 or H-5 or H-8), 8.29 (1H, d, *J* = 8.7 Hz, H-3 or H-4 or H-5 or H-8), 8.16–7.33 (19H, m, Ar, H-3 and/or H-4 and/or H-5 and/or H-8, H-6, H-7), 7.25 (1H, dd, *J* = 11.7, 6.9 Hz, NH_2_), 6.54–6.51 (2H, m, 2 × *p*-cym-CH_Ar_), 6.48 (1H, d, *J* = 5.7 Hz, *p*-cym-CH_Ar_), 5.96 (1H, pt, *J* = 9.2, 9.1 Hz, H-3′ or H-4′), 5.83 (1H, pt, *J* = 9.8, 9.6 Hz, H-3′ or H-4′), 5.71 (1H, d, *J* = 5.7 Hz, *p*-cym-CH_Ar_), 5.04 (1H, dd, *J* = 12.7, 2.3 Hz, H-6′a), 4.99 (1H, d, *J* = 10.2 Hz, H-1′), 4.90 (1H, ddd, *J* = 10.0, 3.4, 2.3 Hz, H-5′), 4.58 (1H, dd, *J* = 11.9, 7.3 Hz, NH_2_), 4.52 (1H, dd, *J* = 12.7, 3.4 Hz, H-6′b), 3.03–2.95 (1H, m, H-2′), 2.82 (1H, hept, *J* = 6.9 Hz, *i*-Pr-C*H*), 1.63 (3H, s, C_6_H_4_-C*H_3_*), 1.28, 1.18 (2 × 3H, 2 d, *J* = 6.9 Hz in both, 2 × *i*-Pr-C*H*_3_); ^13^C NMR (100 MHz, CDCl_3_) δ (ppm): 168.9, 166.3, 165.7, 163.2, 149.4 (3 × C=O, C-2, C-8a), 141.6, 134.4, 133.8, 133.3, 132.5, 131.4, 130.5–130.0, 129.9, 129.4, 129.0–128.6, 127.8, 119.5 (C-3–C-8, C-4a, Ar), 95.0, 92.4 (2 × *p*-cym-C_qAr_), 81.0, 79.3, 77.4, 76.8, 75.2, 74.5, 73.5, 67.2 (4 × *p*-cym-CH_Ar_, C-1′, C-3′–C-5′), 62.1 (C-6′), 54.8 (C-2′), 31.1 (*i*-Pr-*C*H), 23.1, 21.9 (2 × *i*-Pr-*C*H_3_), 17.6 (C_6_H_4_-*C*H_3_). ESI-HRMS positive mode (*m*/*z*): calcd for C_46_H_44_ClN_2_O_7_Os^+^ [M-PF_6_]^+^ 963.2439. Found: 963.2432.

Complex **Ir-7e**

Prepared from compound **7e** (15.5 mg, 0.026 mmol, 2.05 eq.), **Ir-dimer** (10.0 mg, 0.013 mmol) and TlPF_6_ (8.8 mg, 0.025 mmol) according to general procedure VII, with a slight modification. During the reaction, the desired product **Ir-7e** also precipitated from the reaction mixture. Therefore, after completion of the reaction, the solvents were removed under reduced pressure. Then, the residue was treated with CH_3_CN (10 mL), and the insoluble TlCl was filtered off. The resulting solution was evaporated in vacuo. The residue was dissolved in a mixture of CH_3_CN (0.1 mL) and CHCl_3_ (2 mL), and diisopropyl ether (8 mL) was added. The precipitated product was filtered off, then washed with CHCl_3_-diisopropyl ether (1:2, 2 mL) to yield 24.6 mg (88%) of a dark yellow solid. R_f_ = 0.41 (95:5 CHCl_3_-MeOH). ^1^H NMR (400 MHz, CD_3_CN) δ (ppm): 8.72 (1H, d, *J* = 9.0 Hz, H-3 or H-4 or H-5 or H-8), 8.60 (1H, d, *J* = 8.7 Hz, H-3 or H-4 or H-5 or H-8), 8.16–7.43 (19H, Ar, H-3 and/or H-4 and/or H-5 and/or H-8, H-6, H-7), 5.80 (1H, pt, *J* = 9.8, 9.4 Hz, H-3′ or H-4′), 5.73 (1H, pt, *J* = 9.6, 9.3 Hz, H-3′ or H-4′), 5.27 (1H, d, *J* = 12.9, NH_2_), 4.80 (1H, dd, *J* = 12.4, 2.2 Hz, H-6′a), 4.82–4.73 (1H, broad signal, NH_2_), 4.63 (1H, dd, *J* = 12.4, 3.9 Hz, H-6′b), 4.62–4.56 (1H, m, H-5′), 4.55 (1H, d, *J* = 10.5 Hz, H-1′), 3.50–3.52 (1H, m, H-2′), 1.70 (15H, s, Cp*-C*H*_3_); ^13^C NMR (90 MHz, CD_3_CN) δ (ppm): 168.1, 166.8, 166.2, 162.6, 147.1 (3 × C=O, C-2, C-8a), 142.9, 134.9, 134.8, 134.4, 132.6, 131.9, 130.6–130.0, 129.9, 129.6–129.4, 120.4 (Ar, C-3–C-8, C-4a), 89.7 (Cp*), 84.3, 76.4 (2), 70.0 (C-1′, C-3′–C-5′), 63.3 (C-6′), 54.4 (C-2′), 9.9 (Cp*-*C*H_3_). ESI-HRMS positive mode (*m*/*z*): calcd for C_46_H_46_N_2_O_7_Ir^+^ [M+H-Cl-PF_6_]^+^ 931.2929. Found: 9631.2920.

Complex **Rh-7e**

Prepared from compound **7e** (20.0 mg, 0.033 mmol, 2.05 eq.), **Rh-dimer** (10.0 mg, 0.016 mmol) and TlPF_6_ (11.3 mg, 0.032 mmol) according to general procedure VII, with a slight modification. During the reaction, the desired product, **Rh-7e**, also precipitated from the reaction mixture. Therefore, after completion of the reaction, the solvents were removed under reduced pressure. Then, the residue was treated with CH_3_CN (10 mL), and the insoluble TlCl was filtered off. The resulting solution was evaporated in vacuo. The residue was dissolved in a mixture of CH_3_CN (0.1 mL) and CHCl_3_ (2 mL), and diisopropyl ether (8 mL) was added. The precipitated product was filtered off, then washed with CHCl_3_-diisopropyl ether (1:4, 2 mL) to yield 29.9 mg (90%) of a red solid. R_f_ = 0.58 (95:5 CHCl_3_-MeOH). ^1^H NMR (400 MHz, CD_3_CN) δ (ppm): 8.86 (1H, d, *J* = 8.9 Hz, H-3 or H-4 or H-5 or H-8), 8.61 (1H, d, *J* = 8.7 Hz, H-3 or H-4 or H-5 or H-8), 8.12–7.43 (19H, m, Ar, H-3 and/or H-4 and/or H-5 and/or H-8, H-6, H-7), 5.83 (1H, dd, *J* = 10.2, 9.3 Hz, H-3′ or H-4′), 5.68 (1H, pt, *J* = 9.8, 9.4 Hz, H-3′ or H-4′), 4.80 (1H, dd, *J* = 12.6, 2.6 Hz, H-6′a), 4.71 (1H, d, *J* = 10.3 Hz, H-1′), 4.64 (1H, dd, *J* = 12.6, 4.0 Hz, H-6′b), 4.63–4.58 (1H, m, H-5′), 4.49 (1H, d, *J* = 12.6 Hz, NH_2_), 3.95 (1H, pt, *J* = 10.4 Hz, NH_2_), 3.24–3.16 (1H, m, H-2′), 1.68 (15H, s, Cp*-C*H*_3_); ^13^C NMR (90 MHz, CD_3_CN) δ (ppm): 168.2, 166.9, 166.3, 162.7, 147.2 (3 × C=O, C-2, C-8a), 142.3, 134.9, 134.8, 134.4, 132.0, 131.6, 130.8, 130.7–130.5, 130.0, 129.9, 129.8–129.3, 120.8 (Ar, C-3–C-8, C-4a), 98.1, 98.0 (Cp*), 82.1, 77.3, 76.3, 70.0 (C-1′, C-3′–C-5′), 63.4 (C-6′), 54.4 (C-2′), 10.0 (Cp*-*C*H_3_). ESI-HRMS positive mode (*m*/*z*): calcd for C_46_H_44_ClN_2_O_7_Rh^+^ [M-PF_6_]^+^ 875.1965. Found: 875.1953.

### 5.2. X-ray Crystallography

X-ray-quality crystals of **Ru-3a** were grown by slow evaporation of a CHCl_3_-MeOH solvent mixture. A crystal well-looking in a polarized light microscope was fixed under a microscope onto a Mitegen loop using high-density oil. Diffraction intensity data were collected at room temperature using a Bruker-D8 Venture diffractometer (Bruker AXS GmbH, Karlsruhe, Germany) equipped with INCOATEC IμS 3.0 (Incoatec GmbH, Geesthacht, Germany) dual (Cu and Mo) sealed-tube micro sources and a Photon ii charge-integrating pixel array detector (Bruker AXS GmbH, Karlsruhe, Germany) using Mo Kα (λ = 0.71073 Å) radiation. High-multiplicity data collection and integration were performed using APEX4 software (version 2021–4.0, Bruker AXS Inc., 2021, Madison, WI, USA). Data reduction and multiscan absorption correction were performed using SAINT (version 8.40B, Bruker AXS Inc., 2019, Madison, USA). The structure was solved using direct methods and refined on F^2^ using the SHELXL program [80] incorporated into the APEX4 suite. Refinement was performed anisotropically for all non-hydrogen atoms. Hydrogen atoms were placed into geometric positions, except the amino protons, which could be found on the difference electron density map, with the respective N-H distances restrained. Multiscan absorption correction had to be applied because of the presence of heavy atoms and due to the shape of the crystal. Further experimental details are shown in Table S3. The CIF file was manually edited using Publcif software [81], while graphics were prepared using the Mercury program [82]. The results for the X-ray diffraction structure determinations were acceptable according to the Checkcif functionality of PLATON software (Utrecht University, Utrecht, The Netherlands) [83].

Structural parameters such as bond length and angle data were in the expected range (Table S5), except the short Ru-Cl distance. The solid-state structure was stabilized by strong N-H..Cl and N-H..F, as well as weak C-H..Cl hydrogen bonds (Figure S2 and Table S4).

### 5.3. Determination of the Distribution Coefficients (logD)

The logD values of the newly synthesized complexes were determined according to a procedure described in our previous publications [45].

### 5.4. Chemicals for Biology Experiments

All chemicals used in the cell biology and biochemistry assays were obtained from Sigma-Aldrich unless otherwise stated. The free ligands and complexes investigated in this study were dissolved in dimethyl-sulfoxide for biology experiments, and 0.1% dimethyl-sulfoxide was used as a vehicle control.

### 5.5. Cell Lines

Cells were cultured under standard cell culture conditions: 37 °C, 5% CO_2_, humidified atmosphere.

*A2780* cells were cultured in RMPI 1640 medium supplemented with 10% fetal calf serum, 2 mM glutamine and 1% penicillin-streptomycin.

*ID8* cells were cultured in high-glucose DMEM (4.5 g/L glucose) supplemented with 4% fetal calf serum, 2 mM glutamine, 1% penicillin-streptomycin and 1% ITS supplement (I3146, Sigma-Aldrich).

*Capan2* cells were maintained in MEM, 10% fetal bovine serum, 1% penicillin-streptomycin and 2 mM glutamine.

*Human primary dermal fibroblasts* were cultured in low-glucose DMEM (1 g/L glucose) supplemented with 20% fetal calf serum, 2 mM glutamine and 1% penicillin-streptomycin.

*U251* cells were maintained in MEM, 10% fetal bovine serum, 1% penicillin-streptomycin and 2 mM glutamine. 

*MCF7* cells were maintained in MEM, 10% fetal bovine serum and 1% penicillin-streptomycin, 2 mM Glutamine. 

### 5.6. Bacterial Reference Strains

The reference strains of *Staphylococcus aureus* (ATCC29213) and *Enterococcus faecalis* (ATCC29212) were purchased from the ATCC (Manassas, VA, USA).

### 5.7. Clinical Isolates of S. aureus and E. faecium

We used a set of clinical isolates of *S. aureus* and *E. faecium* that were collected at the Medical Center of the University of Debrecen (Hungary) between 1 January 2018. and 31 December 2020. The isolates were reported in [31] and are presented in Table 9. The clinical isolates were identified using a Microflex MALDI-TOF mass spectrometer (Bruker, Billerica, MA, USA). The antibiotic susceptibility of the isolates was tested following the European Committee on Antimicrobial Susceptibility Testing (EUCAST) guidelines, which were valid at the time of collection.

### 5.8. Methylthiazolyldiphenyl-Tetrazolium Bromide (MTT) Reduction Assay

An MTT reduction assay was measures the activity of mitochondrial complex I and can be used to detect toxicity [60,61]. The assay was performed in a manner similar to that described in [84]. Briefly, cells were plated in 96-well plates the day before the assay. Cells were treated with the compounds for 4 h; then, MTT was added to a 0.5 mg/mL final concentration, and cells were incubated at 37 °C in a cell incubator for 40–60 min as a function of the cell line being assessed. Culture medium was removed, the reduced MTT dye was dissolved in dimethyl-sulfoxide, and plates were measured in a plate photometer (Thermo Scientific Multiscan GO spectrophotometer, Waltham, MA, USA) at 540 nm. On each plate, wells were designed to contain vehicle-treated cells. In calculations, the readings for these wells were considered to 1, and all readings were expressed relative to these values.

### 5.9. Sulforhodamine B (SRB) Binding Assay

An SRB assay measures protein content of cells in correlation with the cell number in an assay well and can therefore be used to assess cell proliferation or long-term cytostasis [62]. Cells were seeded in 96-well plates the day before the assay. Cells were treated with the compounds for 48 h. Then medium was removed, and cells were fixed with 10% trichloroacetic acid. Fixed cells were washed in distilled water 3 times, followed by staining with SRB (0.4 m/V% dissolved in 1% acetic acid) for 10 min. Stained cells were washed in 1% acetic acid 5 times; acetic acid was removed, and cells were left to dry. Protein-bound SRB was released by adding 100 μL 10 mM Tris base. Plates were measured in a plate photometer (Thermo Scientific Multiscan GO spectrophotometer, Waltham, MA, USA) at 540 nm. On each plate, wells were designed to contain vehicle-treated cells. In calculations, the readings for these wells were considered to be 1, and all readings were expressed relative to these values.

### 5.10. Broth Microdilution

Microdilution experiments were performed according to the standards of EUCAST [85]. The bacterial isolates to be tested were grown on Mueller–Hinton agar plates. The inoculum density of bacteria was set at 5.0 × 10^5^ CFU/mL in microtiter plates in a final volume of 200 μL Mueller–Hinton broth. The tested concentration range was 0.08–40 μM (10 concentrations, twofold serial dilutions), and a drug-free growth control and an inoculum-free negative control were included. The inoculated plates were incubated for 24 h at 37 °C, then visually assessed. Minimum inhibitory concentration (MIC) was defined as the lowest concentration with a 50%≤ inhibitory effect compared to the growth control. All experiments were performed at least twice in duplicate.

### 5.11. Statistical Evaluation

Statistical analysis was performed using version 8.0.1 of GraphPad Prism. Values were tested for normal distribution using the Shapiro–Wilk normality test. When necessary, values were log-normalized or normalized using the Box–Cox normalization method [86] as indicated in the figure captions. The following statistical test, post hoc test and the level of significance are indicated in the figure captions. Nonlinear regression was performed using the built-in “[Inhibitor] vs. response—Variable slope (four parameters), least square fit” utility of GraphPad, which yielded IC_50_ and Hill slope values if the sigmoid curves reached a plateau of inhibition and there was no decrease between two subsequent data points or when inhibition was over 90%. In other cases, the percentage of inhibition was taken for the maximum concentration (100 μM).

## Figures and Tables

**Table 1:** Synthesis of *C*-(2′-deoxy-2′-nitro-3′,4′,6′-tri-*O*-benzyl-β-d-glucopyranosyl)azines and their transformation into *C*-(2′-amino-2′-deoxy-3′,4′,6′-tri-*O*-benzyl-β-d-glucopyranosyl)azines.

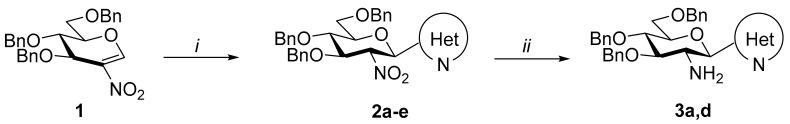 Conditions: (*i*) 1.2–2.0 equiv. of Het-Hlg (Hlg = Br, I), 2.5 M solution of *n*-Bu-Li in hexane (1.2–2.0 equiv.), dry THF, −78 °C; (*ii*) Zn powder, *cc*HCl or 2M aq. HCl, THF-H_2_O (2:1), r.t.					
**Het**		**Conditions and Yields (%)**			
			**2**		**3**
**a**	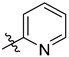	*i*	52	*ii*	64
**b**	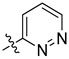	*i*	13	*ii*	NI *
**c**	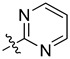	*i*	71	*ii*	NI *
**d**	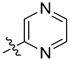	*i*	63	*ii*	7
**e**	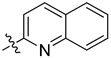	*i*	60	*ii*	NI *

* Could not be isolated.

**Table 2:** Synthesis of *C*-(2′-amino-2′-deoxy-β-d-glucopyranosyl)azines.

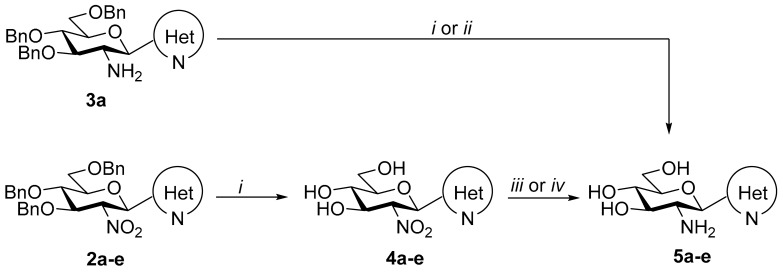 Conditions: (*i*) 1M solution of BCl_3_ in CH_2_Cl_2_ (5 equiv.), dry CH_2_Cl_2_, −78 °C; (*ii*) 1M solution of BCl_3_ in CH_2_Cl_2_ (4 equiv.), pentamethylbenzene (9 equiv.), dry CH_2_Cl_2_, −78 °C; (*iii*) H_2_, Pd(C), dry EtOH, reflux; (*iv*) Sn powder, *cc*HCl, THF-H_2_O (2:1), r.t.					
**Het**		**Conditions and Yields (%)**			
			**4**		**5**
**a**	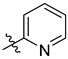	*i*	85	*i*	42 (from **3a**)
				*ii*	95 (from **3a**)
				*iii*	NI * (from **4a**)
**b**	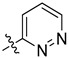	*i*	89	*iii*	NI * (from **4b**)
				*iv*	NI * (from **4b**)
**c**	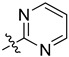	*i*	98	*iii*	38 (from **4c**)
**d**	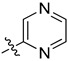	*i*	78	*iii*	66 (from **4d**)
**e**	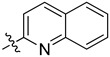	*i*	76	*iii*	NI * (from **4e**)
				*iv*	29 (from **4e**)

* Formation of the expected product was detected, but the compound could not be isolated in a pure state.

**Table 3:** Synthesis of *C*-(2′-amino-2′-deoxy-3′,4′,6′-tri-*O*-benzoyl-β-d-glucopyranosyl)azines.

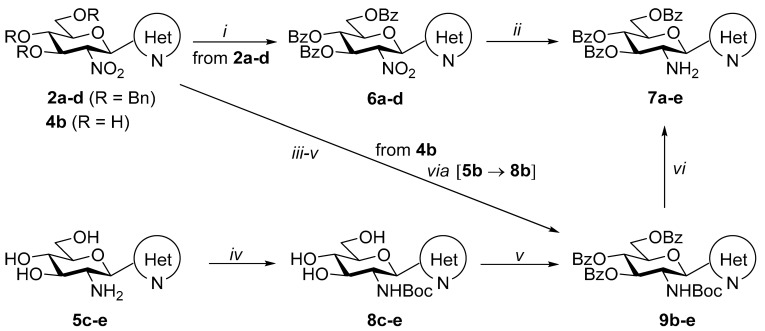 Conditions: (*i*) 6 equiv. of benzoyl chloride, 2 equiv. of Zn(OTf)_2_, dry ClCH_2_CH_2_Cl, r.t.; (*ii*) Zn powder, *cc*HCl or 2M aq. HCl, THF-H_2_O (2:1), r.t.; (*iii*) H_2_, Pd(C), dry EtOH, reflux; (*iv*) 2 equiv. of Boc_2_O, 1,4-dioxane-H_2_O (1:1), r.t.; (*v*) 7.2 equiv. of benzoyl chloride, dry pyridine, 60 °C; (*vi*) 2 equiv. of CF_3_COOH, dry CH_2_Cl_2_, r.t.									
**Het**		**Conditions and Yields (%)**							
			**6**		**7**		**8**		**9**
**a**	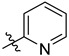	*i*	88	*ii*	38 (from **6a**)	-	-	-	-
**b**	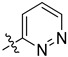	*i*	97	*ii*	NI * (from **6b**)	*iv*	NI *	*iii-v*	27 ** (from **4b**)
				*vi*	88 (from **9b**)				
**c**	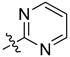	*i*	45	*ii*	NI * (from **6c**)	*iv*	67	*v*	80 (from **8c**)
				*vi*	96 (from **9c**)				
**d**	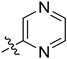	*i*	82	*ii*	NI * (from **6d**)	*iv*	67	*v*	71 (from **8d**)
				*vi*	96 (from **9d**)				
**e**	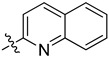	*i*	-	*vi*	84 (from **9e**)	*iv*	80	*v*	81 (from **8e**)

* Could not be isolated. ** Overall yield for three steps.

**Table 4:** Synthesis of half-sandwich platinum-group metal complexes with the *O*-perbenzylated and *O*-unprotected *C*-glucosaminyl azines.

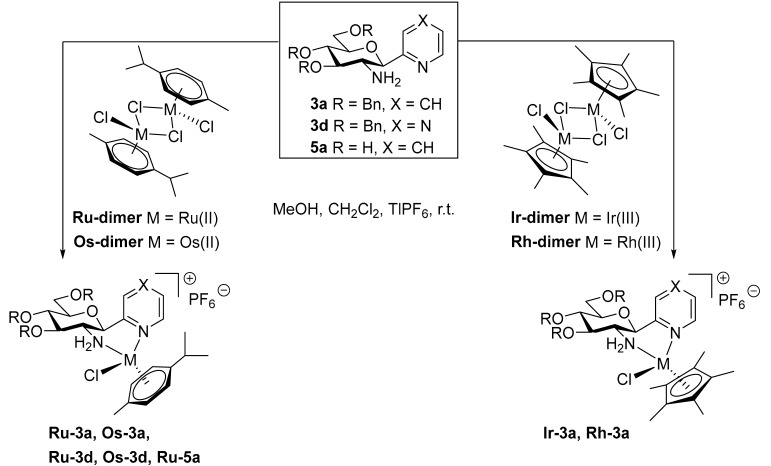						
**Entry**	**Ligand**	**R**	**X**	**M**	**Product**	Yield (%) *
1	**3a**	Bn	CH	Ru(II)	**Ru-3a**	74
2				Os(II)	**Os-3a**	64
3				Ir(III)	**Ir-3a**	93
4				Rh(III)	**Rh-3a**	83
5	**3d**	Bn	N	Ru(II)	**Ru-3d**	33
6		Bn	N	Os(II)	**Os-3d**	47
7	**5a**	H	CH	Ru(II)	**Ru-5a**	43

* Each complex was isolated as a single diastereoisomer.

**Table 5:** Synthesis of half-sandwich platinum-group metal complexes with the *O*-perbenzoylated *C*-glucosaminyl azines.

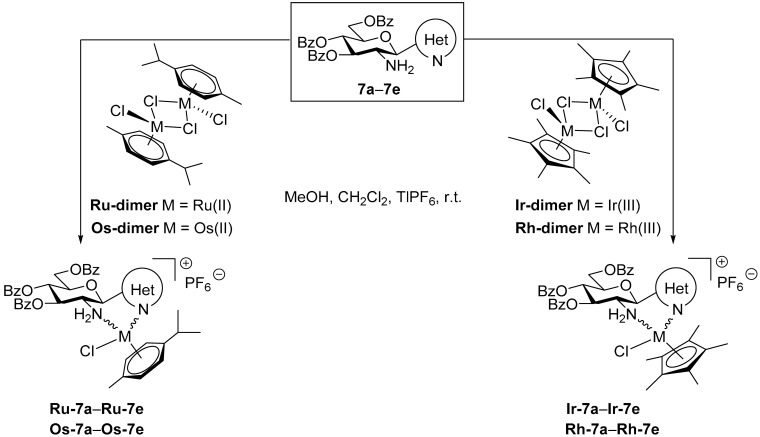						
**Entry**	**Ligand**	**Het**	**M**	**Product**	**Yield (%)**	**Number of Isomers**
1	**7a**	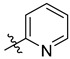	Ru(II)	**Ru-7a**	81	1
2			Os(II)	**Os-7a**	43	1
3			Ir(III)	**Ir-7a**	76	1
4			Rh(III)	**Rh-7a**	86	1
5	**7b**	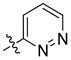	Ru(II)	**Ru-7b**	88	2 (d.r. = 2:1) *
6			Os(II)	**Os-7b**	73	2 (d.r. = 5:4) *
7			Ir(III)	**Ir-7b**	69	2 (d.r. = 9:1 ) *
8			Rh(III)	**Rh-7b**	86	2 (d.r. = 5:1) *
9	**7c**	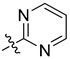	Ru(II)	**Ru-7c**	99	1
10			Os(II)	**Os-7c**	98	1
11			Ir(III)	**Ir-7c**	99	1
12			Rh(III)	**Rh-7c**	99	1
13	**7d**	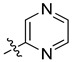	Ru(II)	**Ru-7d**	91	1
14			Os(II)	**Os-7d**	91	1
15			Ir(III)	**Ir-7d**	99	1
16			Rh(III)	**Rh-7d**	96	1
17	**7e**	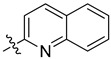	Ru(II)	**Ru-7e**	82	1
18			Os(II)	**Os-7e**	96	1
19			Ir(III)	**Ir-7e**	88	1
20			Rh(III)	**Rh-7e**	90	1

* Diastereomeric ratio (d.r.).

**Table 6:** Kinetic and logD values of the complexes assessed in the present manuscript.

	A2780						Fibroblast						logD
	MTT			SRB			MTT			SRB			
	Max Inhibition	IC_50_	Hill	Max Inhibition	IC_50_	Hill	Max Inhibition	IC_50_	Hill	Max Inhibition	IC_50_	Hill	
**3a**	70.22	ND	ND	>90	36.23	1.33	32.22	ND	ND	75	ND	ND	
**Ru-3a**	>90	8.02	1.68	>90	1.86	2.98	80.22	ND	ND	75	9.62	2.87	2.72
**Os-3a**	85.65	ND	ND	>90	2.13	2.12	69.36	ND	ND	75	15.08	3.09	1.78
**Ir-3a**	82.71	ND	ND	>90	1.69	1.41	80.22	ND	ND	75	14.14	2.12	3.18
**Rh-3a**	25.36	ND	ND	>90	9.81	1.11	37.82	ND	ND	75	ND	ND	1.64
													
**3d**	ND	ND	ND	13.07	ND	ND	18.39	ND	ND	13.15	ND	ND	
**Ru-3d**	>90	19.34	2.33	>90	3.77	2.31	80.31	ND	ND	64.44	ND	ND	2.06
**Os-3d**	86.83	32.34	1.98	>90	6.83	2.52	73.24	ND	ND	30.17	ND	ND	2.64
													
**5a**	ND	ND	ND	ND	ND	ND							
**Ru-5a**	ND	ND	ND	ND	ND	ND							−1.91
													
**7a**	18.61	ND	ND	76.24	ND	ND	27.02	ND	ND	26.00	ND	ND	
**Ru-7a**	>90	9.15	2.54	>90	4.11	2.46	65.18	ND	ND	56.31	ND	ND	1.15
**Os-7a**	>90	13.77	ND	>90	8.58	2.26	65.18	ND	ND	49.00	ND	ND	2.15
**Ir-7a**	>90	11.64	ND	>90	6.20	1.96	65.18	ND	ND	56.31	ND	ND	1.08
**Rh-7a**	35.97	ND	ND	>90	18.25	1.88	27.02	ND	ND	32.56	ND	ND	1.20
													
**7b**	9.12	ND	ND	18.38	ND	ND	11.39	ND	ND	ND	ND	ND	
**Ru-7b**	68.22	ND	ND	>90	17.78	2.57	47.04	ND	ND	ND	ND	ND	1.39
**Os-7b**	53.39	ND	ND	>90	56.37	1.26	27.22	ND	ND	ND	ND	ND	1.32
**Ir-7b**	53.39	ND	ND	77.58	ND	ND	27.22	ND	ND	ND	ND	ND	1.17
**Rh-7b**	62.85	ND	ND	>90	31.98	1.97	11.39	ND	ND	ND	ND	ND	1.41
													
**7c**	26.30	ND	ND	79.53	ND	ND							
**Ru-7c**	33.13	ND	ND	66.28	ND	ND							1.31
**Os-7c**	33.13	ND	ND	66.28	ND	ND							1.59
**Ir-7c**	41.53	ND	ND	82.52	ND	ND							1.40
**Rh-7c**	33.13	ND	ND	79.53	ND	ND							1.13
													
**7d**	18.08	ND	ND	73.37	ND	ND							
**Ru-7d**	64.04	ND	ND	80.44	ND	ND							1.26
**Os-7d**	46.72	ND	ND	80.44	ND	ND							1.42
**Ir-7d**	38.71	ND	ND	80.44	ND	ND							1.30
**Rh-7d**	18.08	ND	ND	64.76	ND	ND							1.04
													
**7e**	13.56	ND	ND	45.20	ND	ND							
**Ru-7e**	27.04	ND	ND	67.50	ND	ND							2.22
**Os-7e**	27.04	ND	ND	67.50	ND	ND							2.21
**Ir-7e**	27.04	ND	ND	61.28	ND	ND							1.39
**Rh-7e**	31.89	ND	ND	67.32	ND	ND							1.66

ND—not detectable.

**Table 7:** The kinetic values of compounds **3a** and **Ru-3a** in cancer cell lines other than A2780.

	ID8			U251			MCF7			Capan2		
	SRB			SRB			SRB			SRB		
	Max Inhibition	IC_50_	Hill	Max Inhibition	IC_50_	Hill	Max Inhibition	IC_50_	Hill	Max Inhibition	IC_50_	Hill
**3a**	>90	13.05	2.10	>90	29.36	1.77	>90	21.33	1.43	>90	14.99	ND
**Ru-3a**	>90	2.54	3.87	>90	3.97	2.55	>90	2.30	2.02	>90	2.25	2.55

ND—not detectable.

**Table 8:** The MIC values (μM) of a subset of ligands and complexes.

	**3a**	**Ru-3a**	**Os-3a**	**Ir-3a**	**Rh-3a**	**3d**		**Ru-3d**	**Os-3d**		
*E. faecalis* ATCC 29,212	>40	10	10	20	>40	>40		20	>40		
27,085 VRE	>40	5	10	10	>40	>40		20	40		
25,051 VRE	>40	5	5	10	>40	>40		20	40		
25,498 VRE	>40	5	10	10	>40	>40		20	40		
											
*S. aureus* ATCC 29213	>40	5	5	20	>40	>40		20	40		
24,408 MRSA	>40	5	10	10	>40	>40		20	40		
24,328 MRSA	>40	5	5	10	>40	>40		20	40		
20,426 MRSA	>40	5	5	10	>40	>40		20	40		
											
24,268 MRSA	>40	5	10	5	>40	>40		20	40		
24,035 MRSA	>40	5	10	20	>40	>40		20	40		
24,272 MRSA	>40	5	10	10	>40	>40		20	40		
											
	**7a**	**Ru-7a**	**Os-7a**	**Ir-7a**	**Rh-7a**	**7b**		**Ru-7b**	**Os-7b**	**Ir-7b**	**Rh-7b**
*E. faecalis* ATCC 29,212	>40	5	5	40	>40	>40		10	10	40	>40
27,085 VRE	>40	5	10	40	>40	>40		10	10	40	>40
25,051 VRE	>40	5	5	40	>40	>40		10	10	40	>40
25,498 VRE	>40	5	5	40	>40	>40		10	10	40	>40
											
*S. aureus* ATCC 29,213	>40	5	10	20	>40	>40		20	20	>40	>40
24,408 MRSA	>40	5	5	10	>40	>40		10	10	40	>40
24,328 MRSA	>40	5	5	20	>40	>40		20	20	40	>40
20,426 MRSA	>40	5	10	10	>40	>40		10	10	40	>40
											
24,268 MRSA	>40	5	5	10	>40	>40		10	10	40	>40
24,035 MRSA	>40	5	10	10	>40	>40		10	10	40	>40
24,272 MRSA	>40	5	5	10	>40	>40		20	10	40	>40
											
	**7e**	**Ru-7e**	**Os-7e**	**Ir-7e**	**Rh-7e**		**Vehicle Control**				
*E. faecalis* ATCC 29.212	>40	>40	>40	>40	>40		>40				
27,085 VRE	>40	>40	>40	>40	>40		>40				
25,051 VRE	>40	>40	>40	>40	>40		>40				
25,498 VRE	>40	>40	>40	>40	>40		>40				
										
*S. aureus* ATCC 29.213	>40	>40	>40	>40	>40		>40				
24,408 MRSA	>40	>40	>40	>40	>40		>40				
24,328 MRSA	>40	>40	>40	>40	>40		>40				
20,426 MRSA	>40	>40	>40	>40	>40		>40				
										
24,268 MRSA	>40	>40	>40	>40	>40		>40				
24,035 MRSA	>40	>40	>40	>40	>40		>40				
24,272 MRSA	>40	>40	>40	>40	>40		>40				

**Table 9:** The clinical isolates used in the study. VRE—vancomycin-resistant *Enterococcus*, MRSA—methicillin-resistant *Staphylococcus aureus*.

Species and Strain Identity		Sample	Year
Reference	*E. faecalis*		
VRE	25,051	Nephrostoma	2018
VRE	27,085	Wound	2018
VRE	25,498	Rectal swab to screen for multiresistant pathogens	2018
			
Reference	*S. aureus*		
MRSA	24,272	Throat	2018
MRSA	24,408	Bronchial	2018
MRSA	20,426	Blood	2020
MRSA	24,035	Wound	2018
MRSA	24,328	Throat	2018
MRSA	24,268	Throat	2018

## Data Availability

The datasets generated and analyzed for this study can be found at Figshare.com (https://figshare.com/s/9ec2a005e6b9e5874c07; DOI: 10.6084/m9.figshare.21786020). The supplementary crystallographic data for the **Ru-3a** structure described in this paper can be obtained free of charge from the Cambridge Crystallographic Data Centre via http://www.ccdc.cam.ac.uk/data_request/cif (accessed on 29 March 2023) using reference deposition number 2241543.

## Supplementary Materials

The following supporting information can be downloaded at https://www.mdpi.com/article/10.3390/molecules28073058/s1, Copies of ^1^H and ^13^C NMR spectra; Table S1. Changes in the chemical shifts of selected ^1^H NMR resonances as a result of complex formation; Table S2. Distribution coefficient of the synthesized complexes (logD); X-Ray diffraction study of **Ru-3a**: Figure S1. Ball-and-stick model of **Ru-3a** with a partial numbering scheme, Table S3. Experimental details, Figure S2. Packing diagram of **Ru-3a**, Table S4. Hydrogen-bond geometry (Å, º) for **Ru-3a**, Table S5. Geometric parameters (Å, º) for **Ru-3a**.
