# Supplementary material for: Half-Sandwich Type Platinum-Group Metal Complexes of C-Glucosaminyl Azines: Synthesis and Antineoplastic and Antimicrobial Activities
Source: Molecules. 2023 Mar 29;28(7):3058. doi: 10.3390/molecules28073058 (PMC10096180; doi:10.3390/molecules28073058)
Supplement: Supplementary file 1 [file molecules-28-03058-s001.zip › SUPPORTING INFORMATION-final-230327.pdf]

SUPPORTING INFORMATION

Half-sandwich type platinum-group metal complexes of *C*-glucosaminyl azines: synthesis and antineoplastic and antimicrobial activities

István Kacsir[1,2], Adrienn Sipos[3,4,5], Evelin Major[6], Nikolett Bajusz[6], Attila Bényei[7], Péter Buglyó[8], László Somsák[1], Gábor Kardos[6], Péter Bai[3,4,5,9,10,*], Éva Bokor[1,*]

[1]Department of Organic Chemistry, University of Debrecen, P.O. Box 400, H-4002 Debrecen, Hungary;

[2]Doctoral School of Chemistry, University of Debrecen, P.O. Box 400, H-4002 Debrecen, Hungary;

[3]Department of Medical Chemistry, Faculty of Medicine, University of Debrecen, Egyetem Tér 1., H-4032 Debrecen, Hungary;

[4]The Hungarian Academy of Sciences, Center of Excellence, Hungary

[5]MTA-DE Cell Biology and Signaling Research Group ELKH, Debrecen, Hungary

[6]Department of Metagenomics, University of Debrecen, H-4032 Debrecen, Hungary;

[7]Department of Physical Chemistry, Faculty of Sciences and Technology, University of Debrecen, Egyetem Tér 1., H-4032 Debrecen, Hungary;

[8]Department of Inorganic & Analytical Chemistry, Faculty of Sciences and Technology, University of Debrecen, Egyetem Tér 1., H-4032 Debrecen, Hungary;

[9]NKFIH-DE Lendület Laboratory of Cellular Metabolism, H-4032 Debrecen, Hungary

[10]Research Center for Molecular Medicine, Faculty of Medicine, University of Debrecen, H-4032 Debrecen, Hungary

Table of contents:

1. **Copies of ^{1}H and ^{13}C NMR spectra** **3**
2. **Table S1.** Changes of the chemical shifts of selected ^{1}H NMR resonances as a result of the complex formation **62**
3. **Table S2.** Distribution coefficient of the synthesized complexes (logD)* **63**
4. **X-Ray diffraction study of Ru-3a** **65**
   - General information 65
   - **Figure S1.** Ball and stick model of **Ru-3a** with partial numbering scheme 65
   - **Table S3.** Experimental details 66
   - **Figure S2.** Packing diagram of **Ru-3a** 67
   - **Table S4.** Hydrogen-bond geometry (Å, º) for **Ru-3a** 67
   - **Table S5.** Geometric parameters (Å, º) for **Ru-3a** 68
5. **References** **74**

1. Copies of ^{1}H and ^{13}C NMR spectra

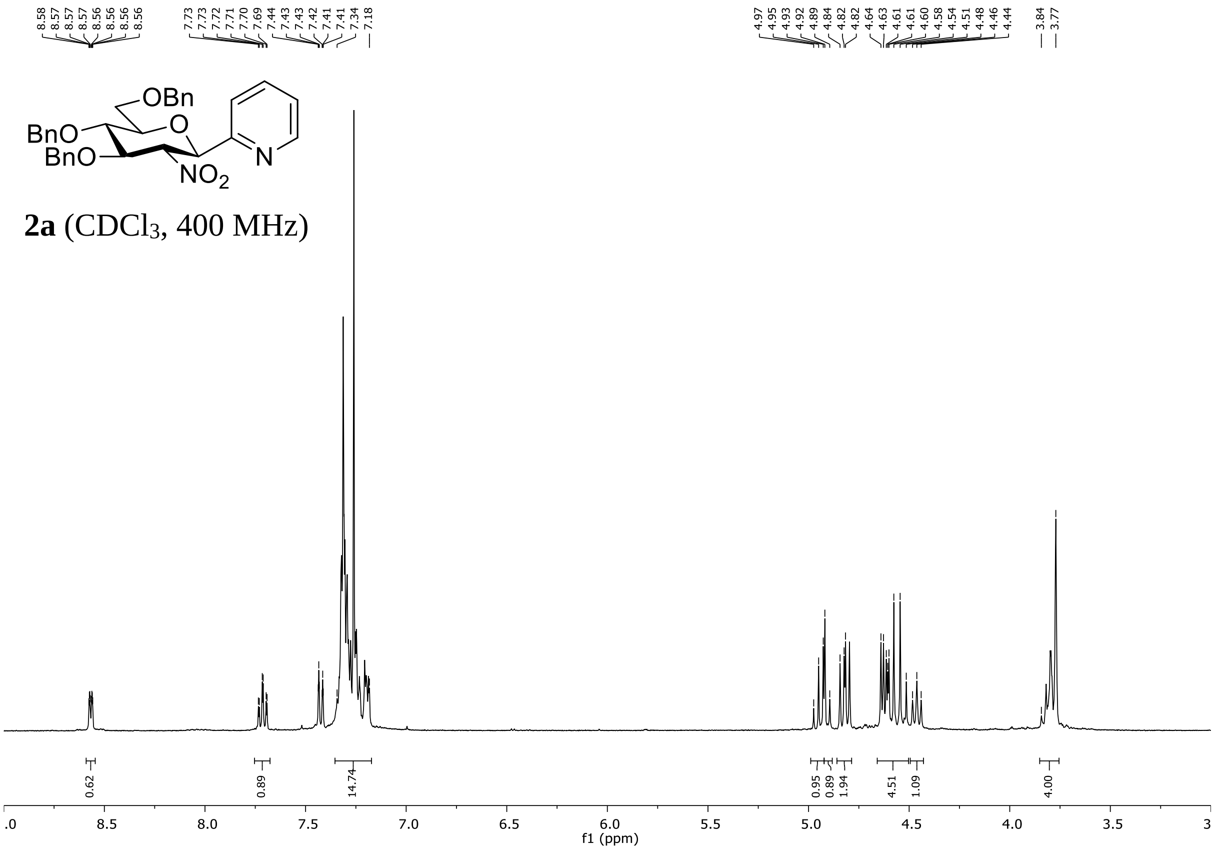

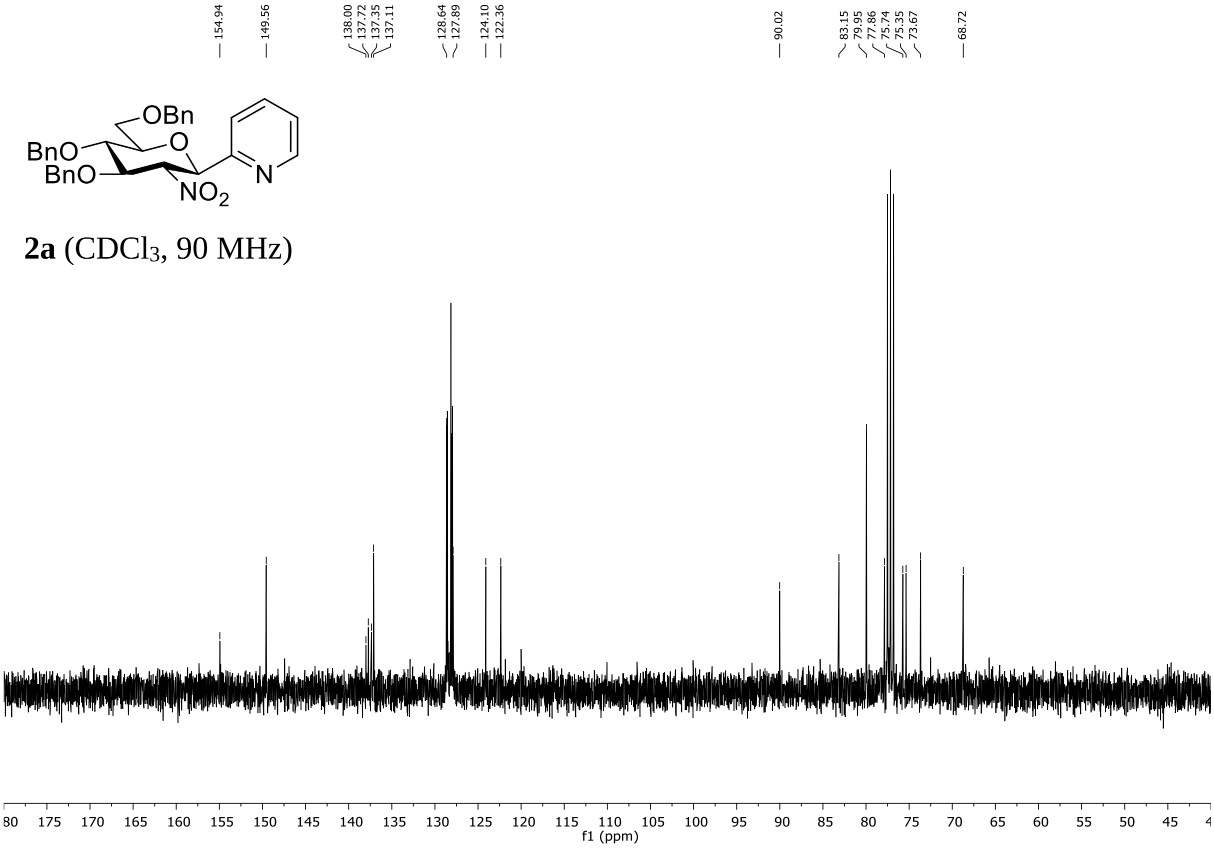

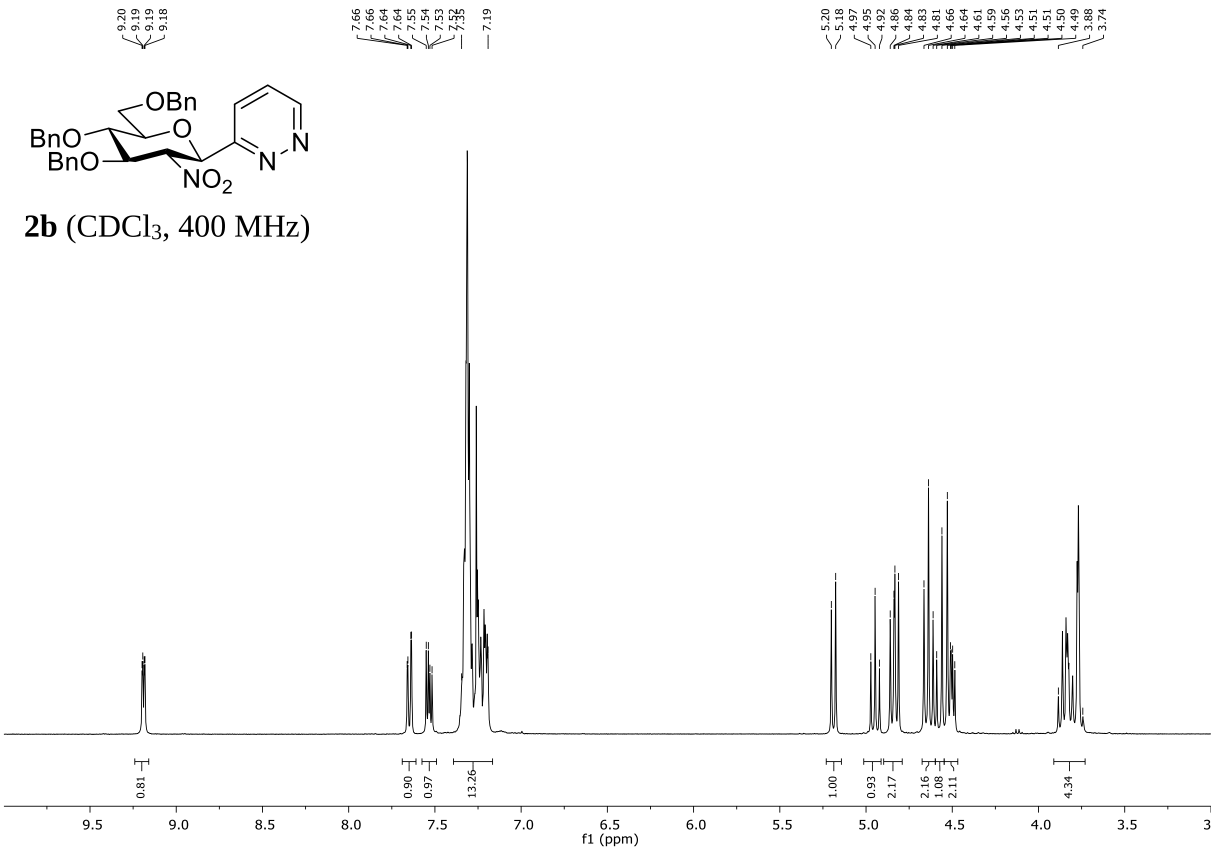

2b ($CDCl_3$, 400 MHz)

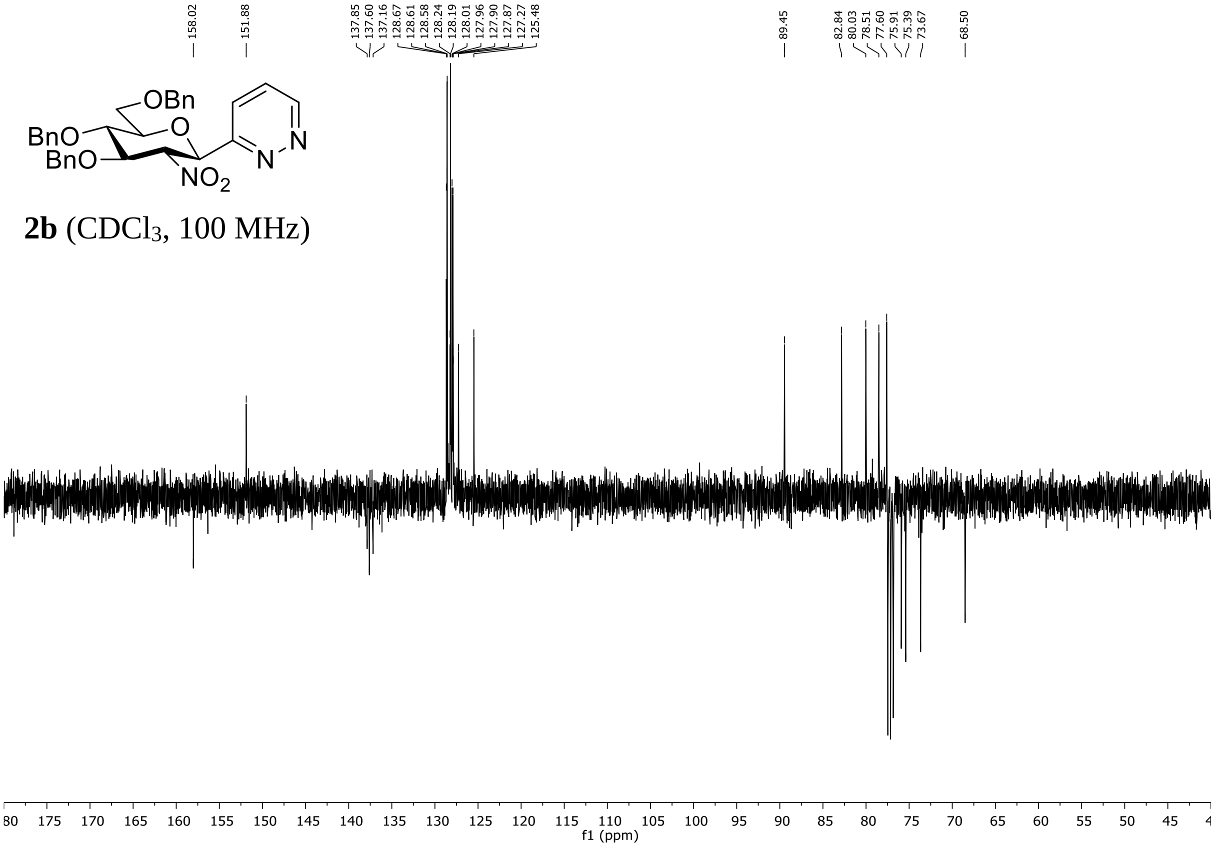

2b ($CDCl_3$, 100 MHz)

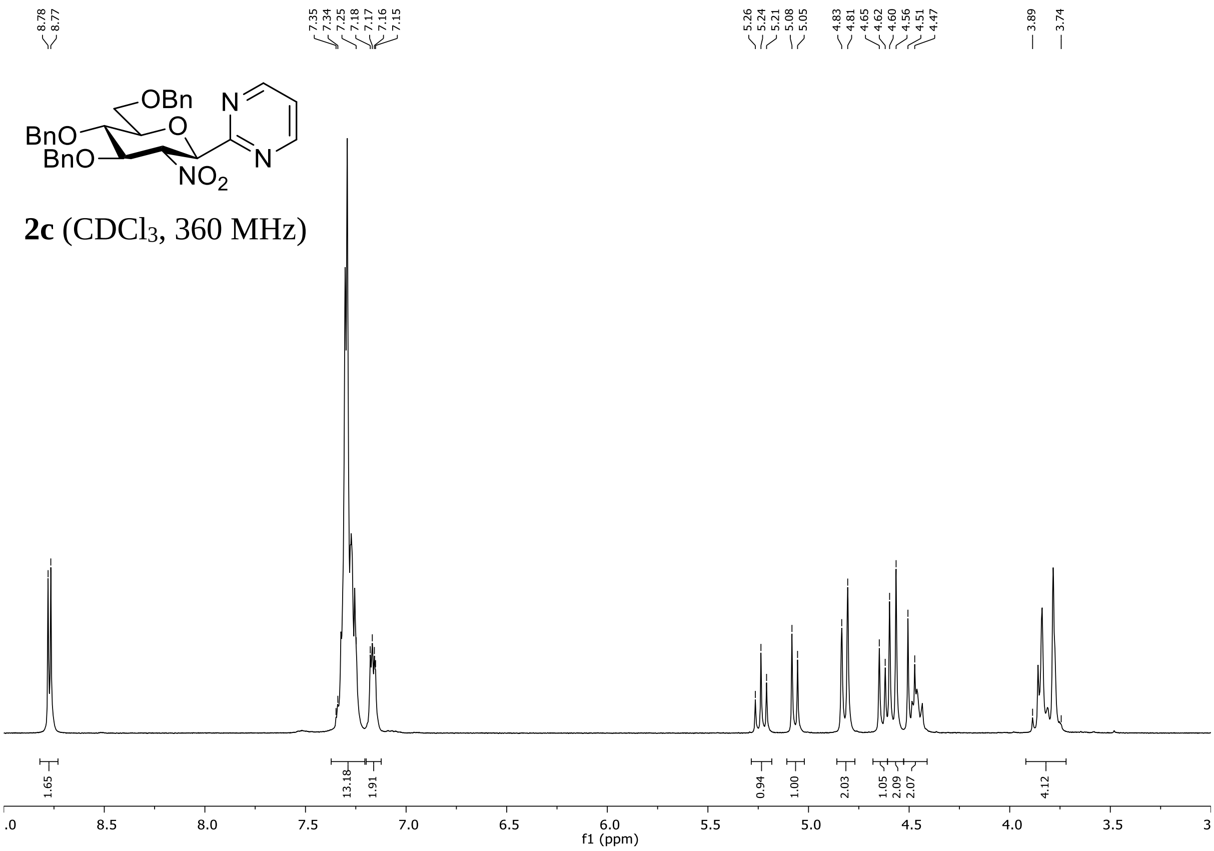
8.78
8.77
7.35
7.34
7.25
7.18
7.17
7.16
7.15
5.26
5.24
5.21
5.08
5.05
4.83
4.81
4.65
4.62
4.60
4.56
4.51
4.47
3.89
3.74
OBn
BnO
BnO
O
N
N
NO2
2c (CDCl3, 360 MHz)
1.65
13.18
1.91
0.94
1.00
2.03
1.05
2.09
2.07
4.12
f1 (ppm)

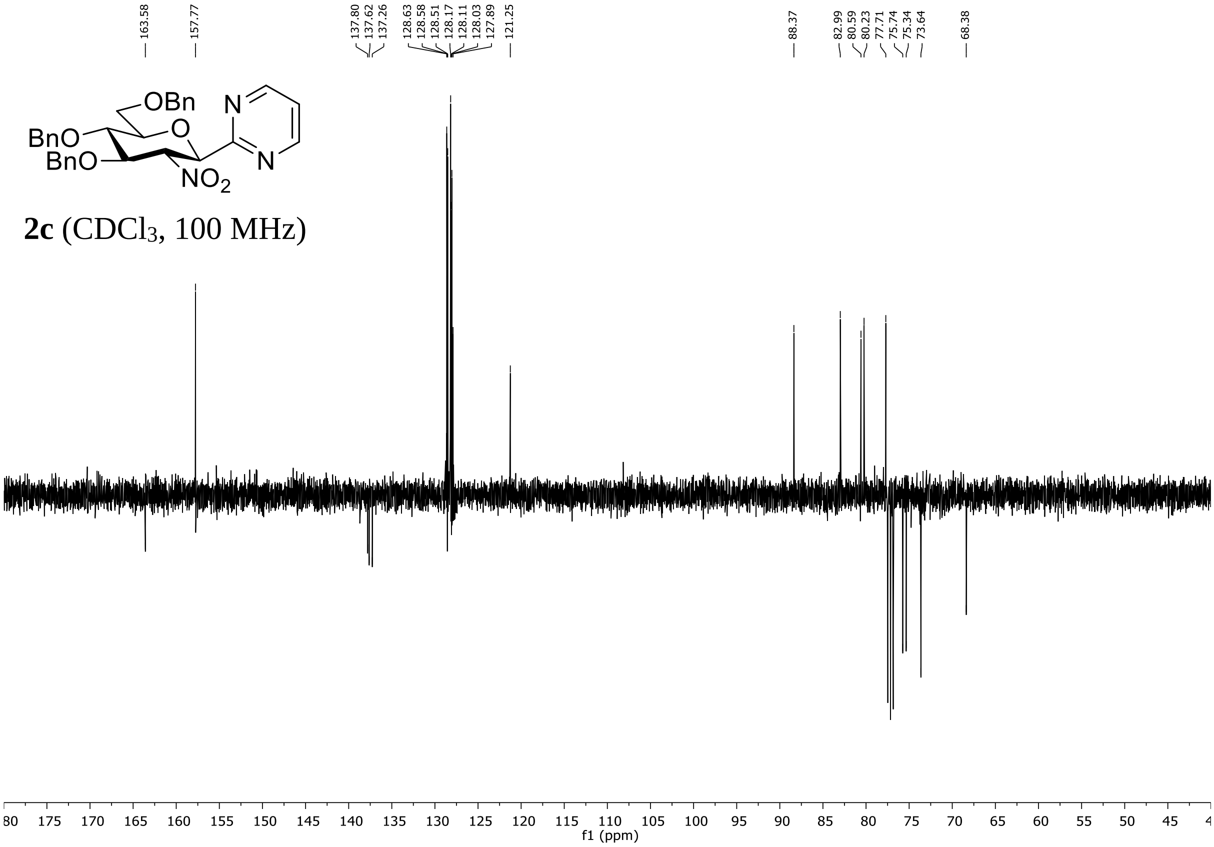
163.58
157.77
137.80
137.62
137.26
128.63
128.58
128.51
128.17
128.11
128.03
127.89
121.25
88.37
82.99
80.59
80.23
77.71
75.74
75.34
73.64
68.38
OBn
BnO
BnO
O
N
N
NO2
2c (CDCl3, 100 MHz)
f1 (ppm)

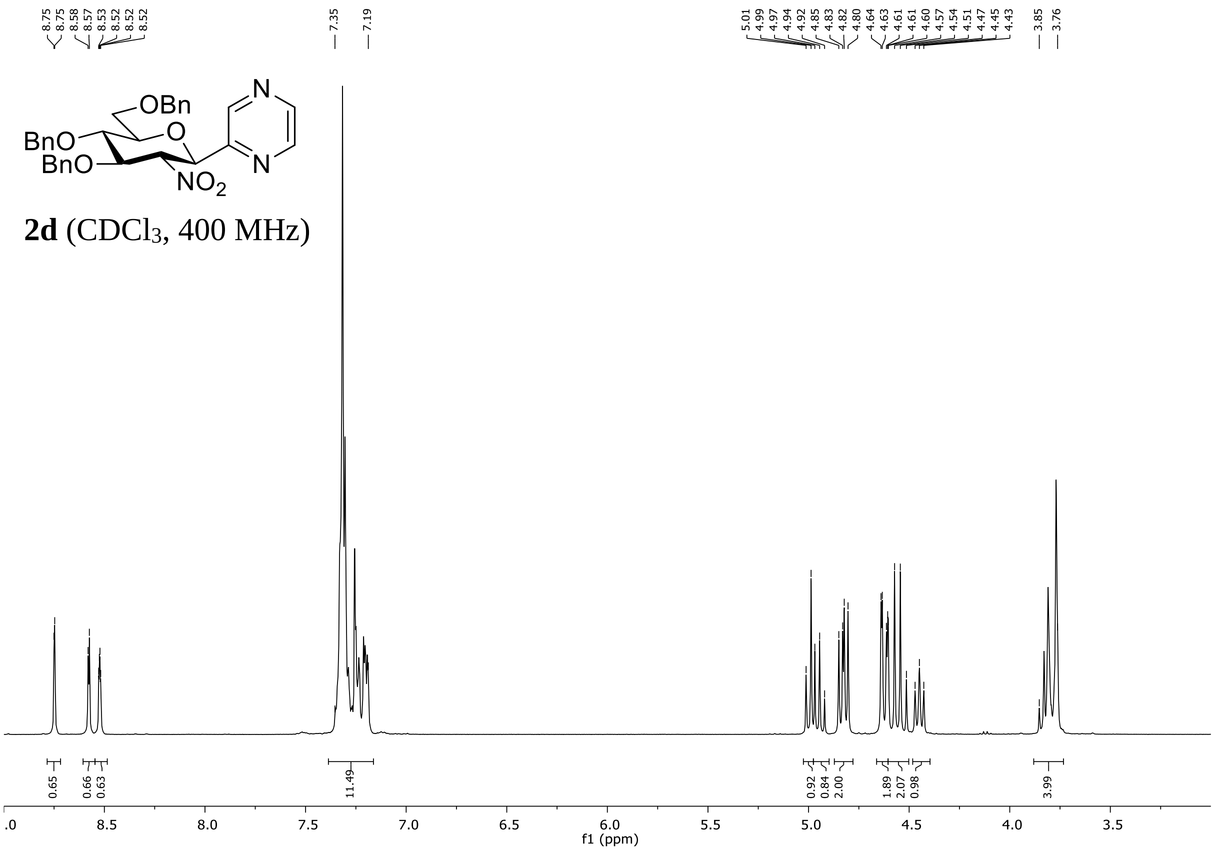
8.75
8.75
8.58
8.57
8.53
8.52
8.52
8.52
7.35
7.19
5.01
4.99
4.97
4.94
4.92
4.85
4.83
4.82
4.80
4.64
4.63
4.61
4.61
4.60
4.57
4.54
4.51
4.47
4.45
4.43
3.85
3.76
OBn
BnO
BnO
O
N
N
NO_2
2d ($CDCl_3$, 400 MHz)
0.65
0.66
0.63
11.49
0.92
0.84
2.00
1.89
2.07
0.98
3.99
f1 (ppm)

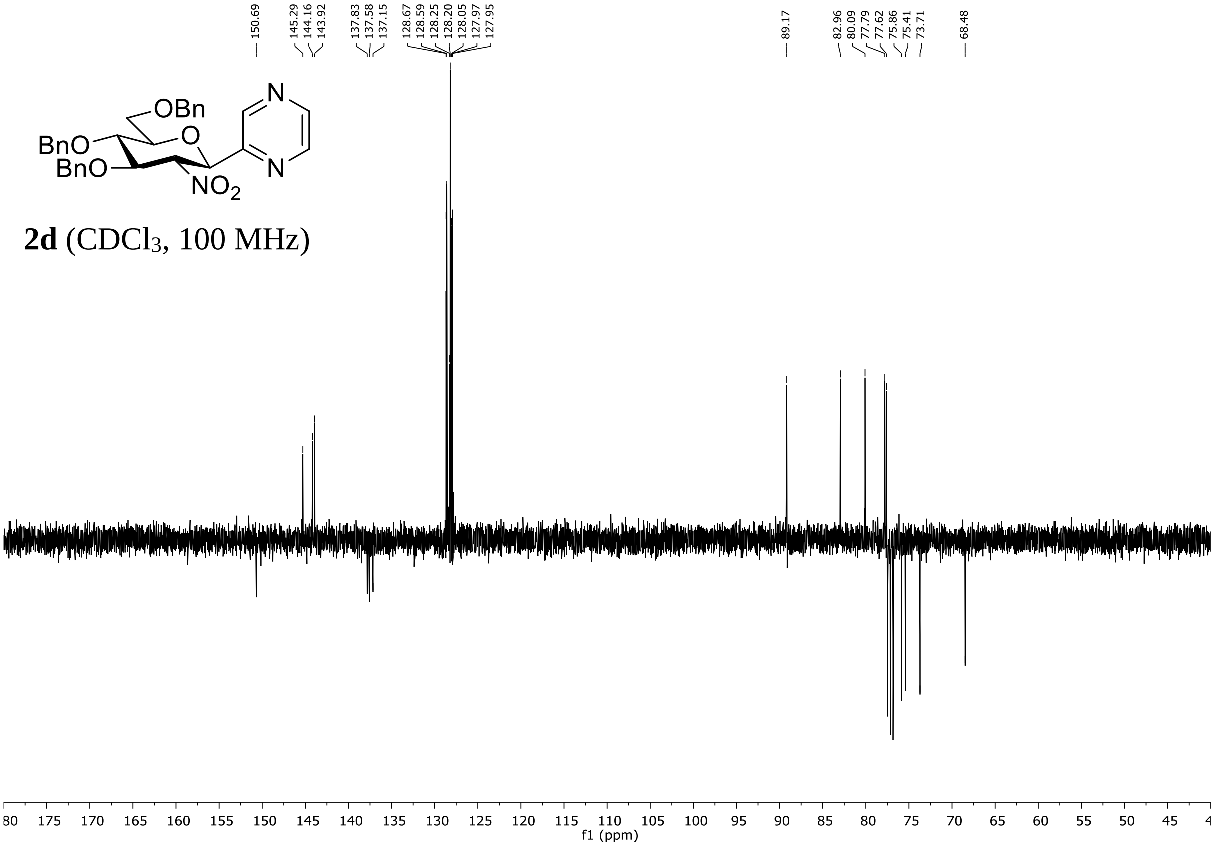
150.69
145.29
144.16
143.92
137.83
137.58
137.15
128.67
128.59
128.25
128.20
128.05
127.97
127.95
89.17
82.96
80.09
77.79
77.62
75.86
75.41
73.71
68.48
OBn
BnO
BnO
O
N
N
NO_2
2d ($CDCl_3$, 100 MHz)
f1 (ppm)

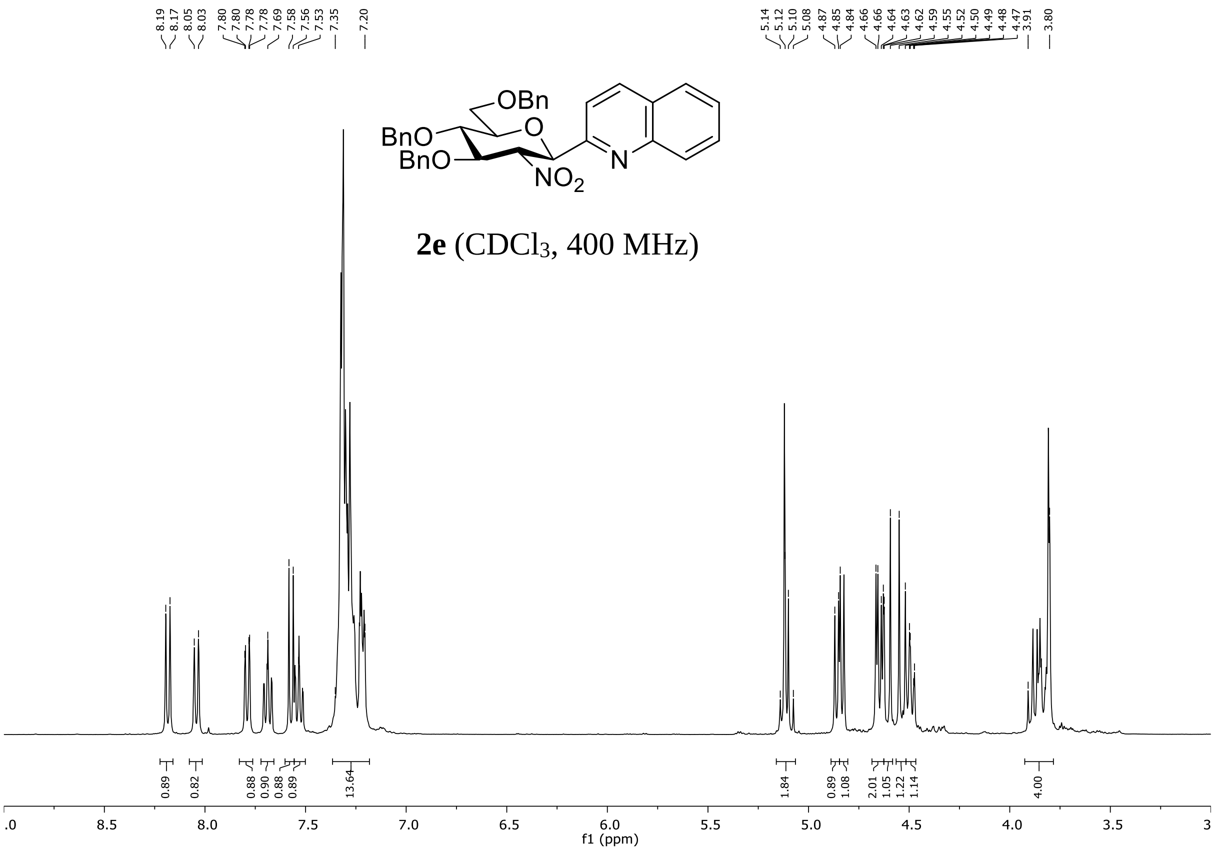

2e ($CDCl_3$, 400 MHz)

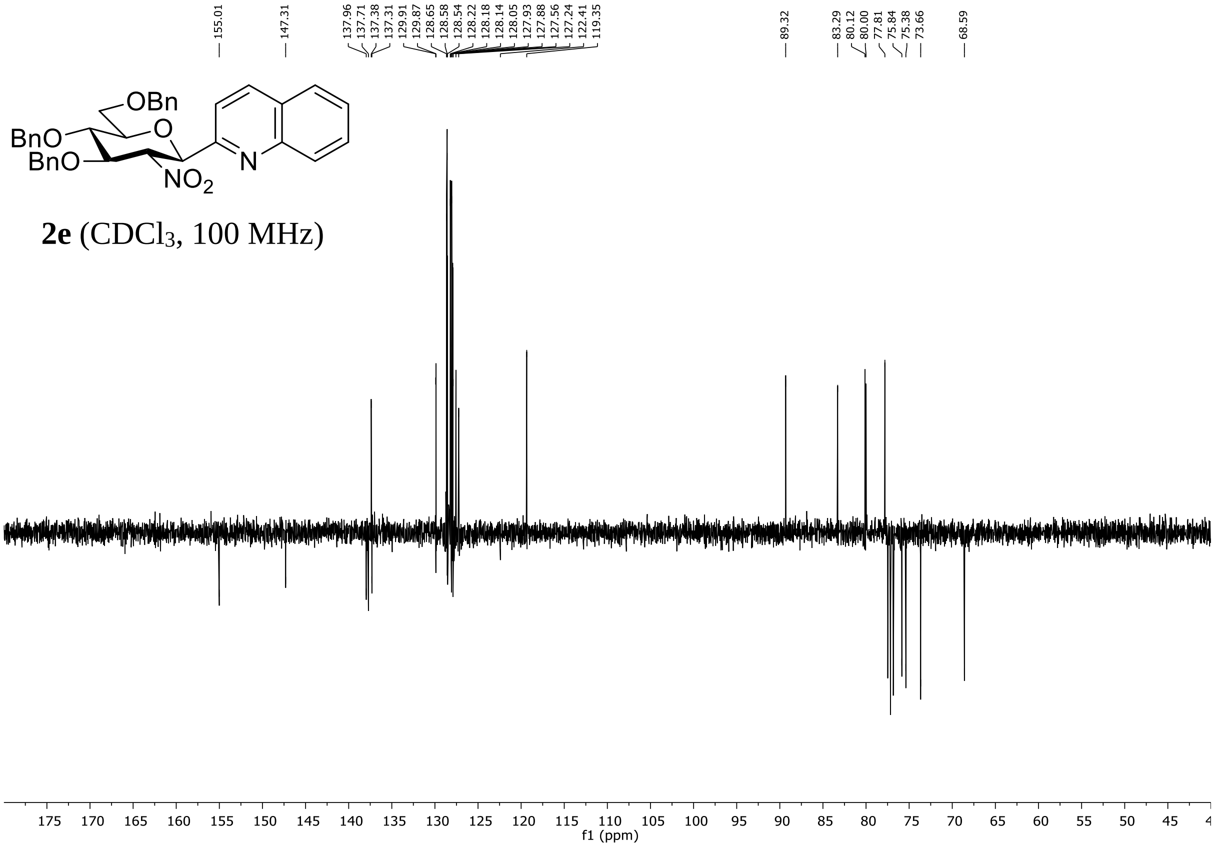

2e ($CDCl_3$, 100 MHz)

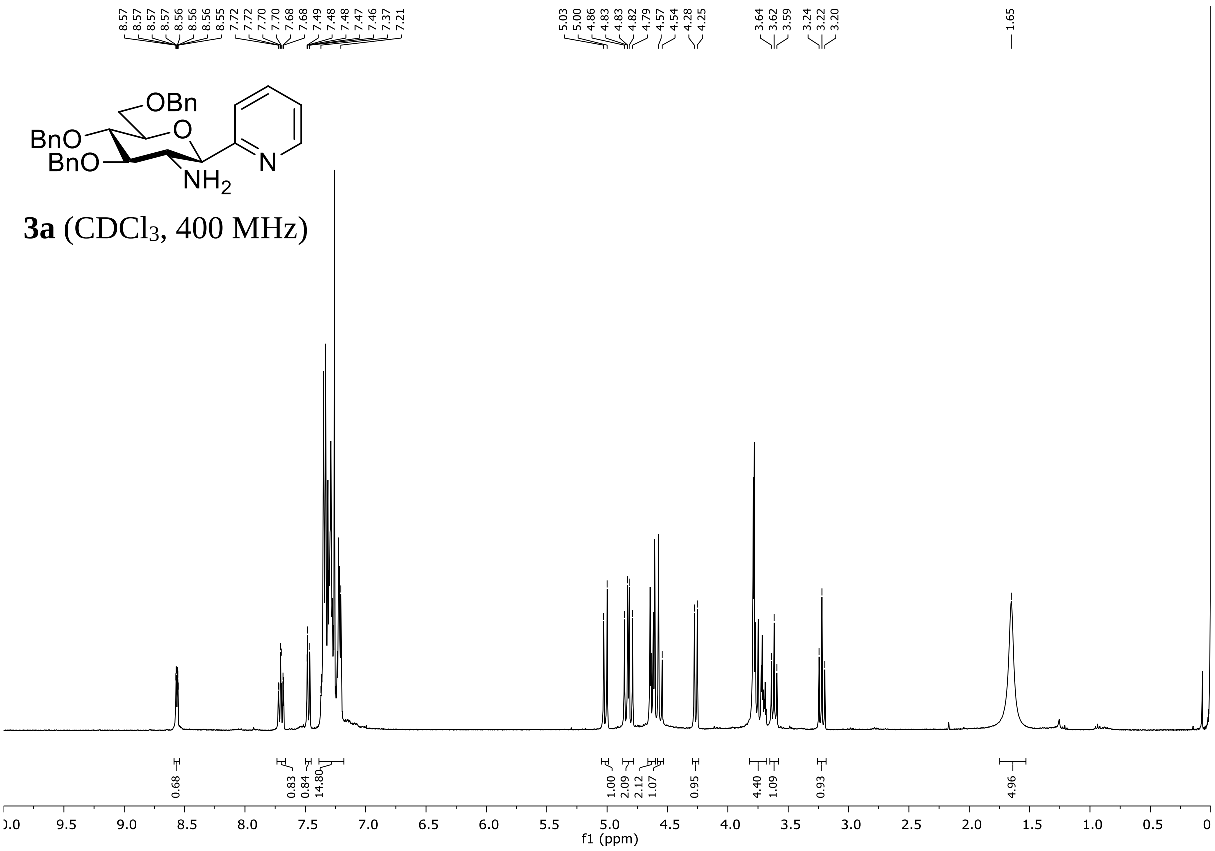
8.57 8.57 8.57 8.56 8.56 8.56 8.55 7.72 7.72 7.70 7.70 7.68 7.68 7.49 7.48 7.48 7.47 7.46 7.37 7.21
5.03 5.00 4.86 4.83 4.83 4.82 4.79 4.57 4.54 4.28 4.25
3.64 3.62 3.59 3.24 3.22 3.20
1.65
OBn
BnO
BnO
O
N
NH2
3a (CDCl3, 400 MHz)
0.68 0.83 0.84 14.80 1.00 2.09 2.12 1.07 0.95 4.40 1.09 0.93 4.96
f1 (ppm)

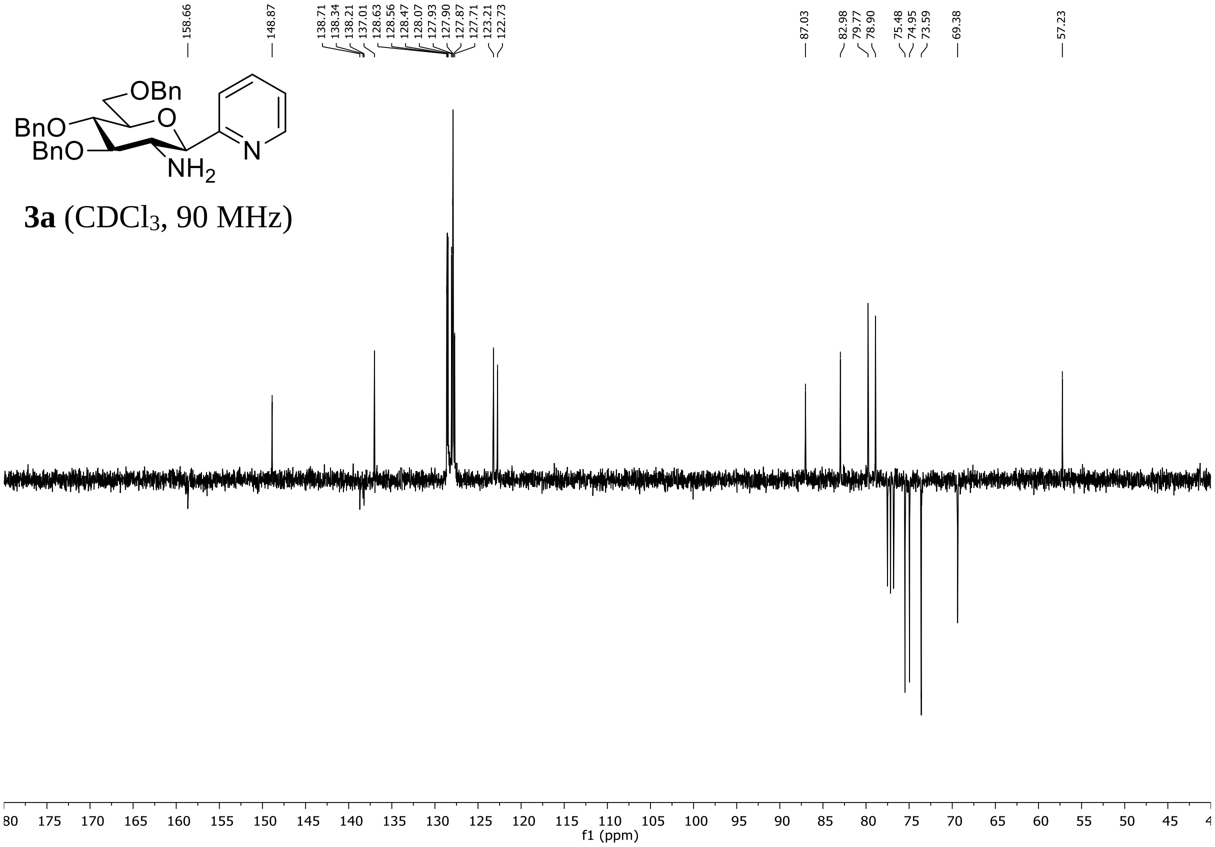
158.66
148.87
138.71 138.34 138.21 137.01 128.63 128.56 128.47 128.07 127.93 127.90 127.87 127.71 123.21 122.73
87.03
82.98 79.77 78.90
75.48 74.95 73.59
69.38
57.23
OBn
BnO
BnO
O
N
NH2
3a (CDCl3, 90 MHz)
f1 (ppm)

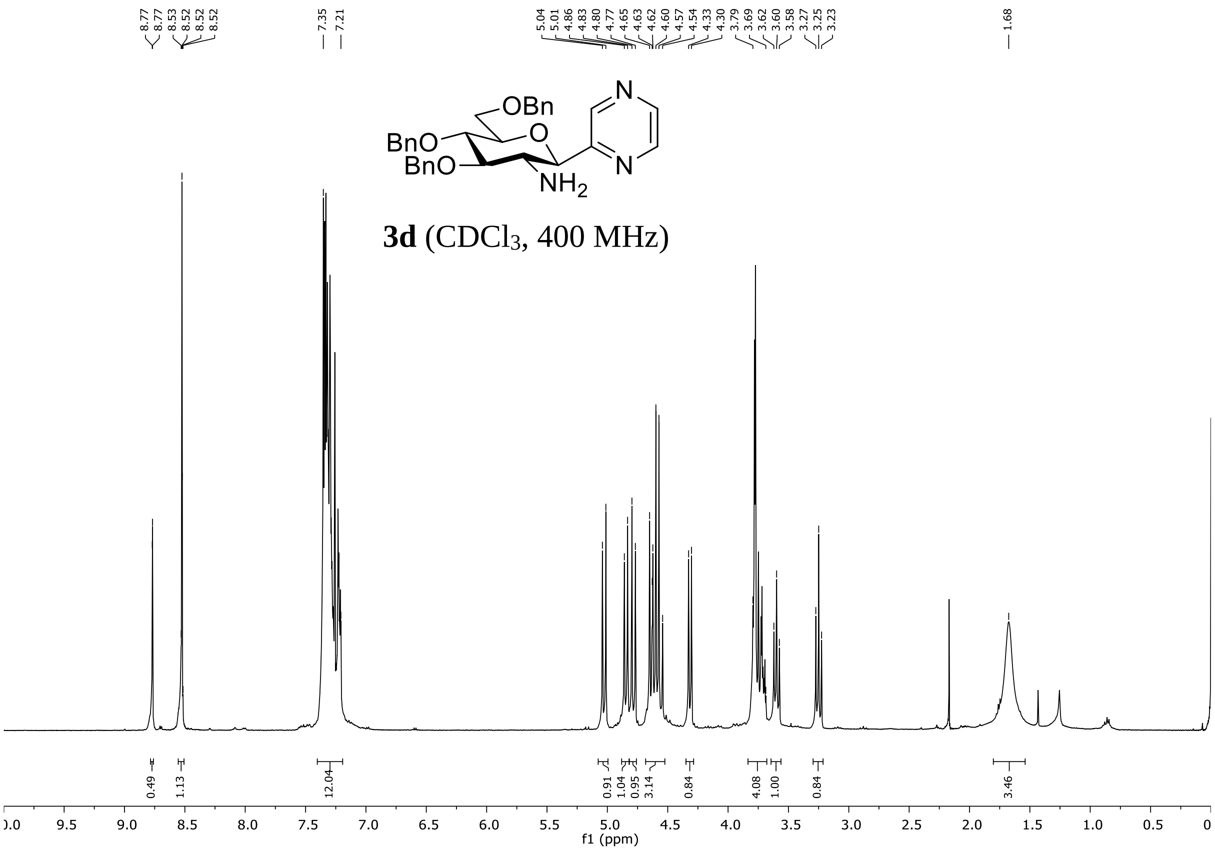

3d ($CDCl_3$, 400 MHz)

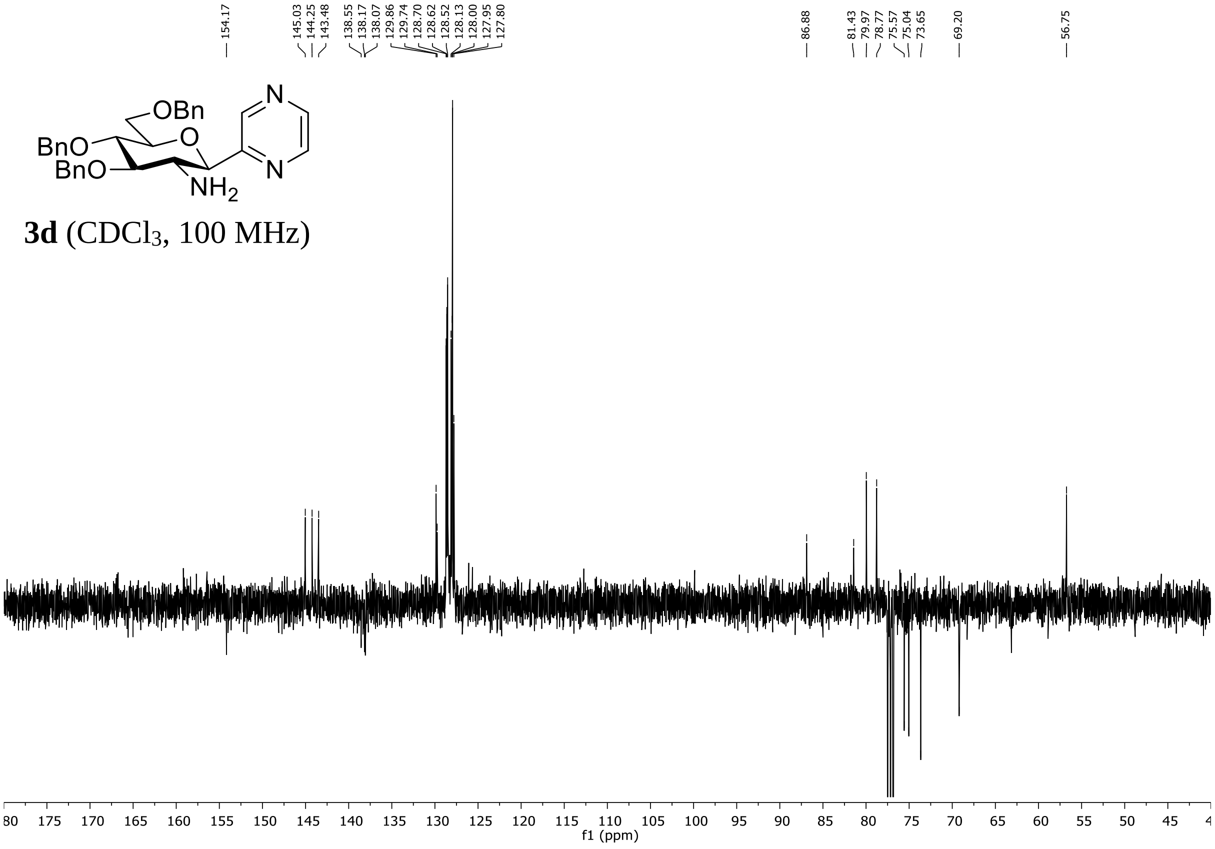

3d ($CDCl_3$, 100 MHz)

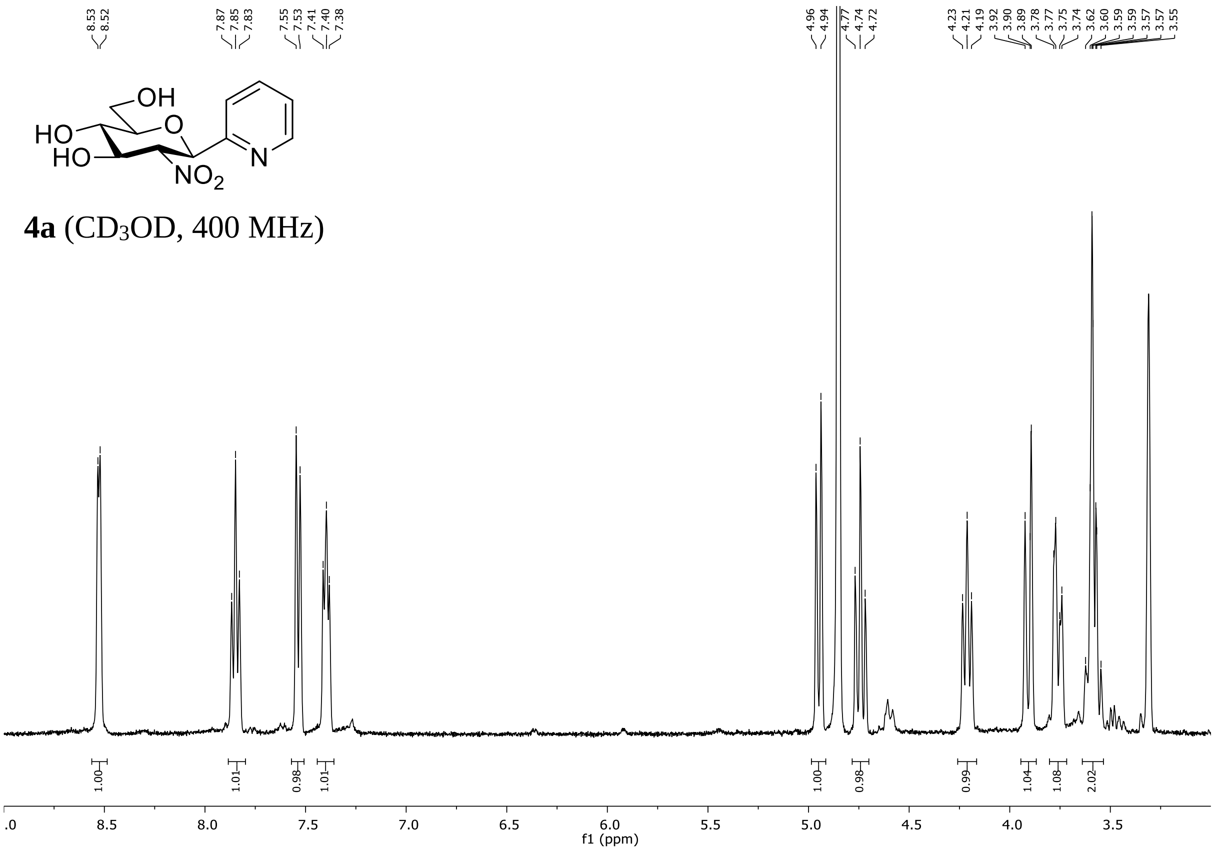

4a (CD_3OD, 400 MHz)

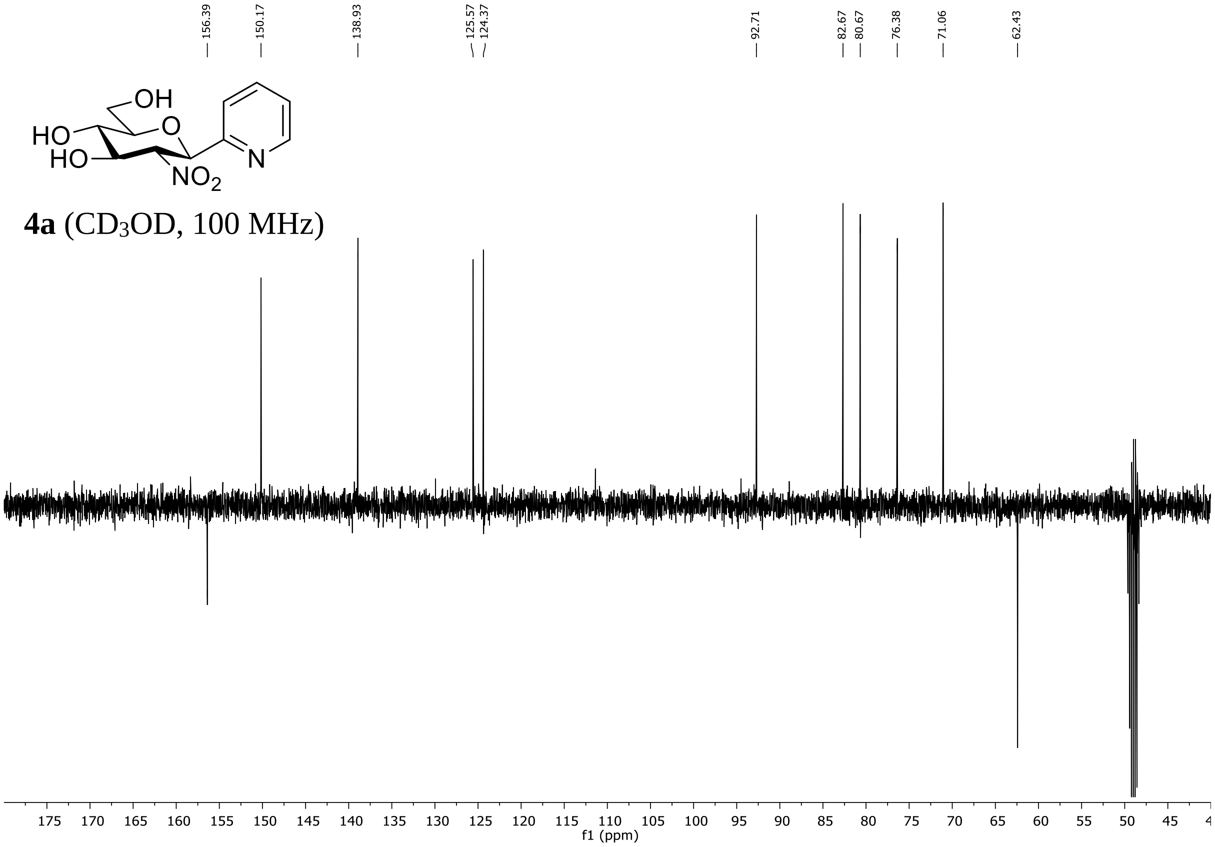

4a (CD_3OD, 100 MHz)

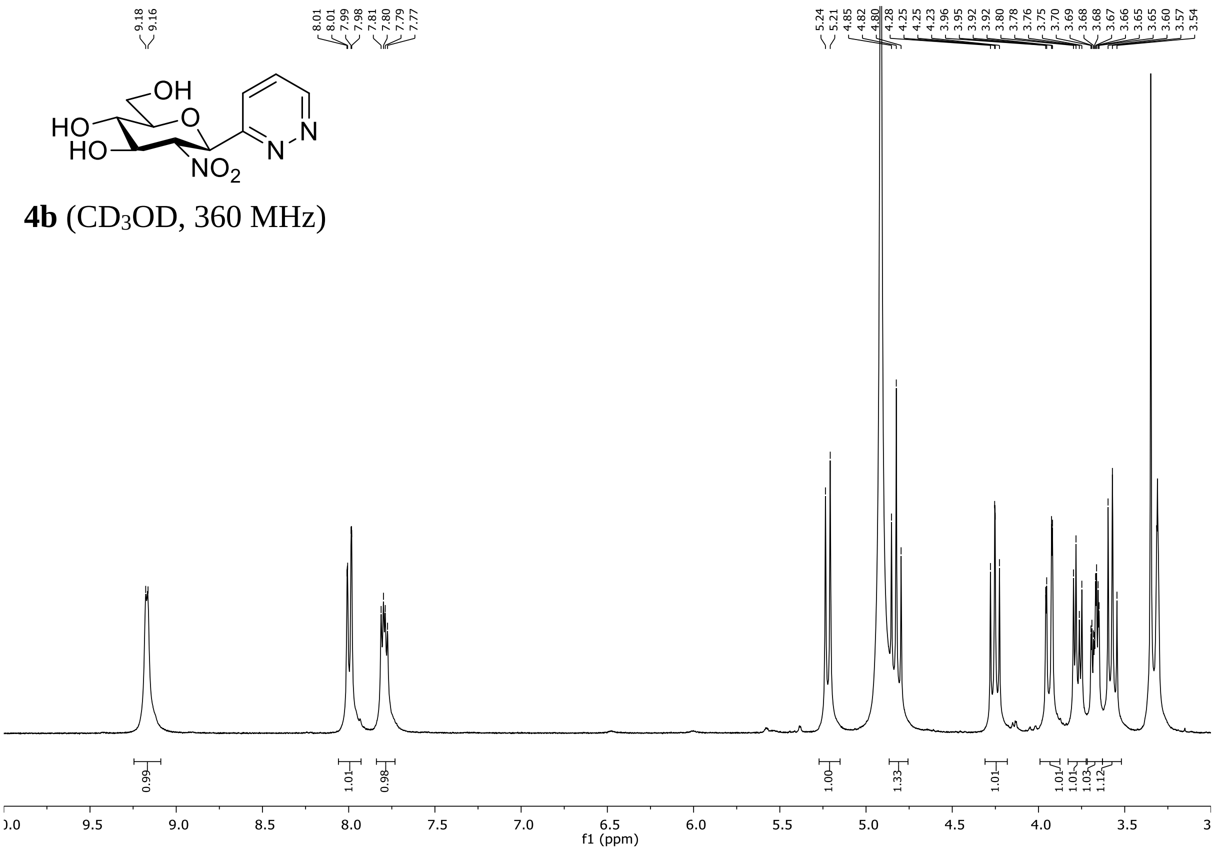

4b (CD_3OD, 360 MHz)

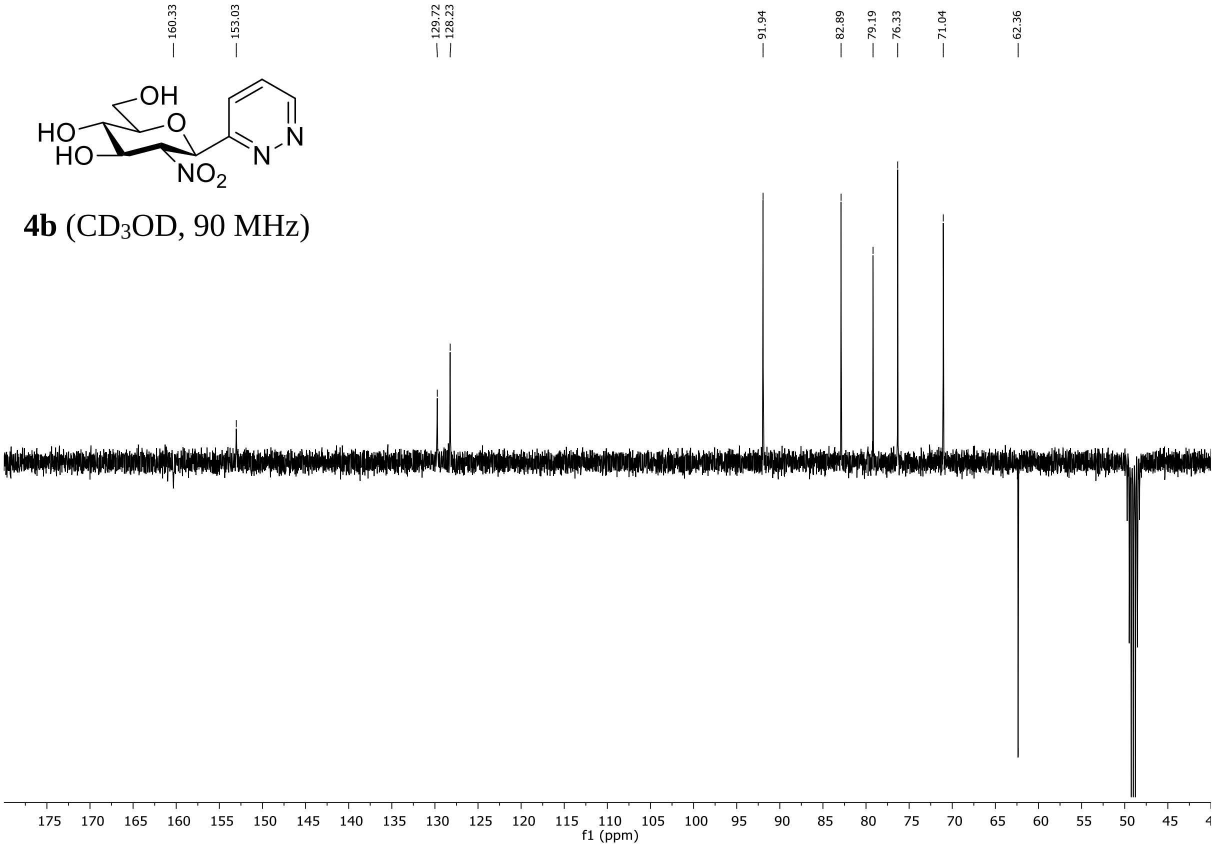

4b (CD_3OD, 90 MHz)

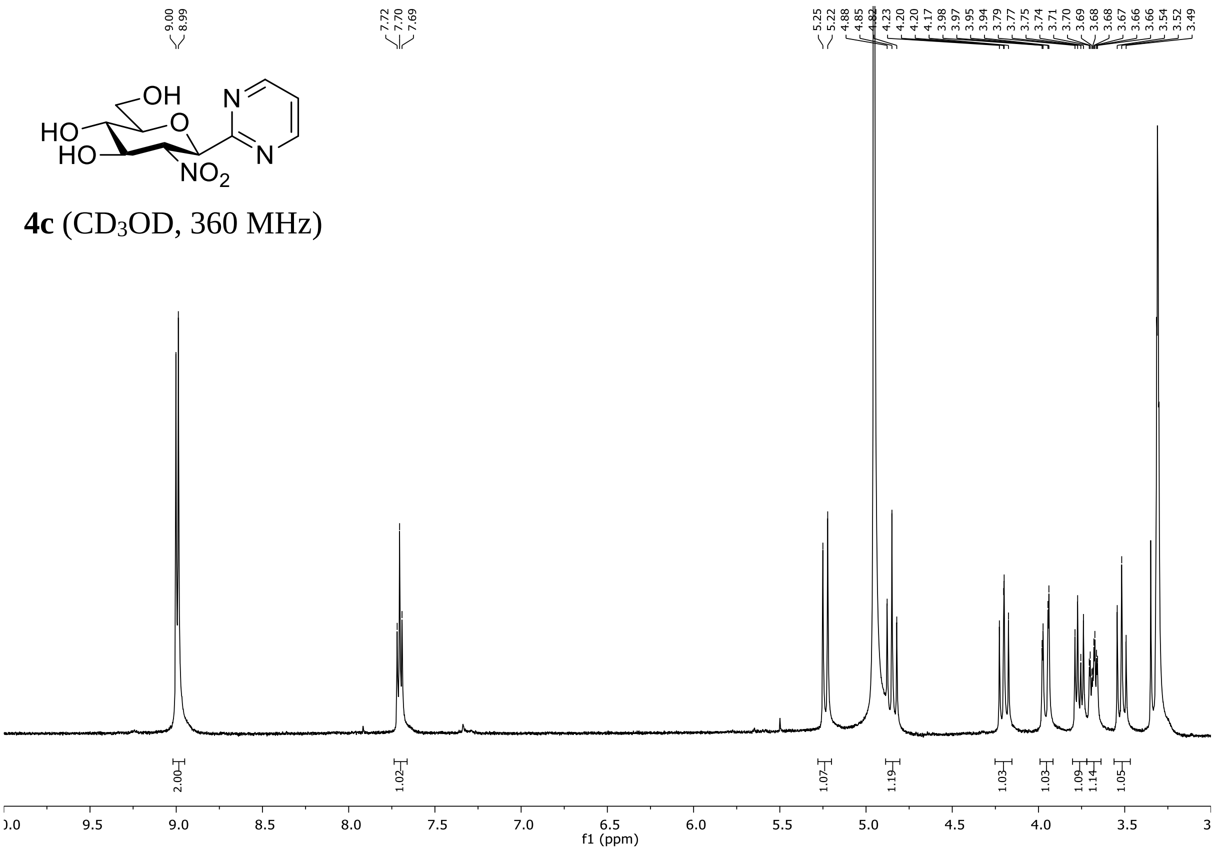

4c (CD_3OD, 360 MHz)

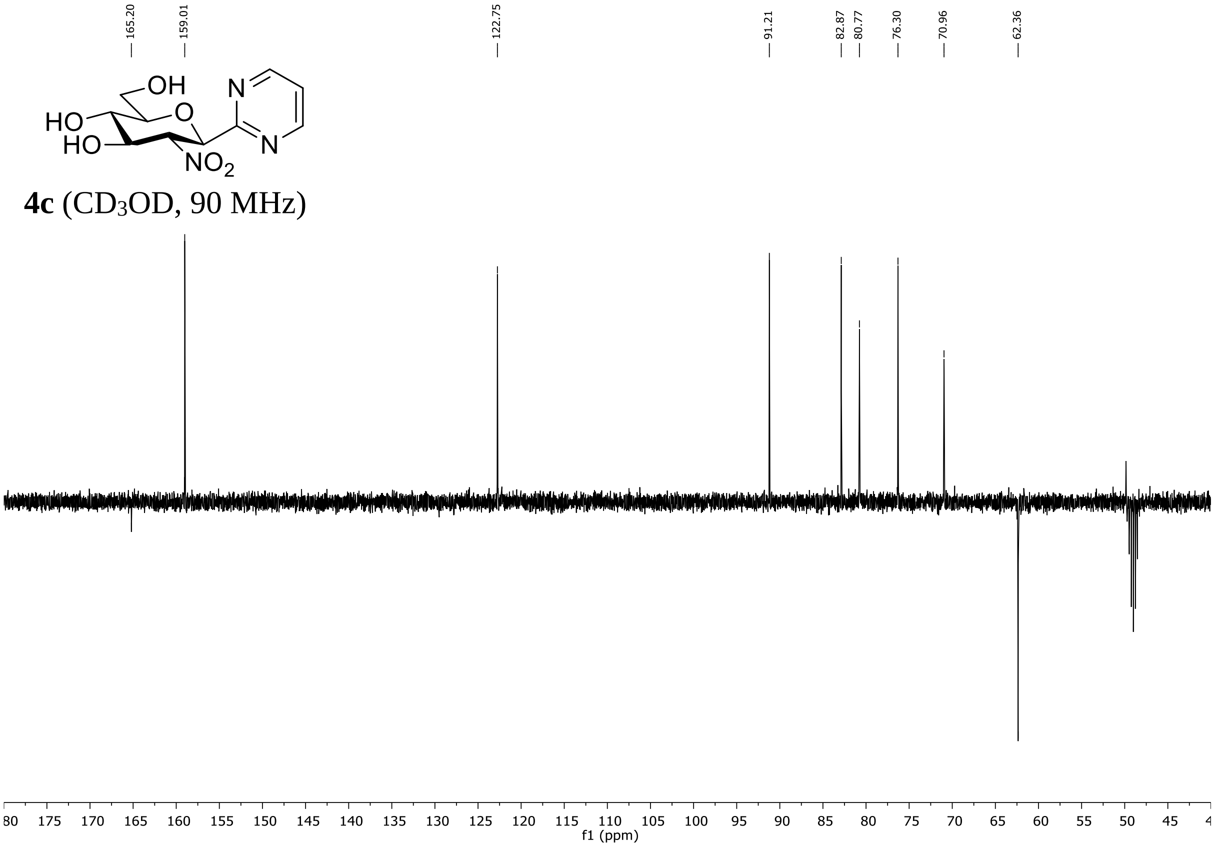

4c (CD_3OD, 90 MHz)

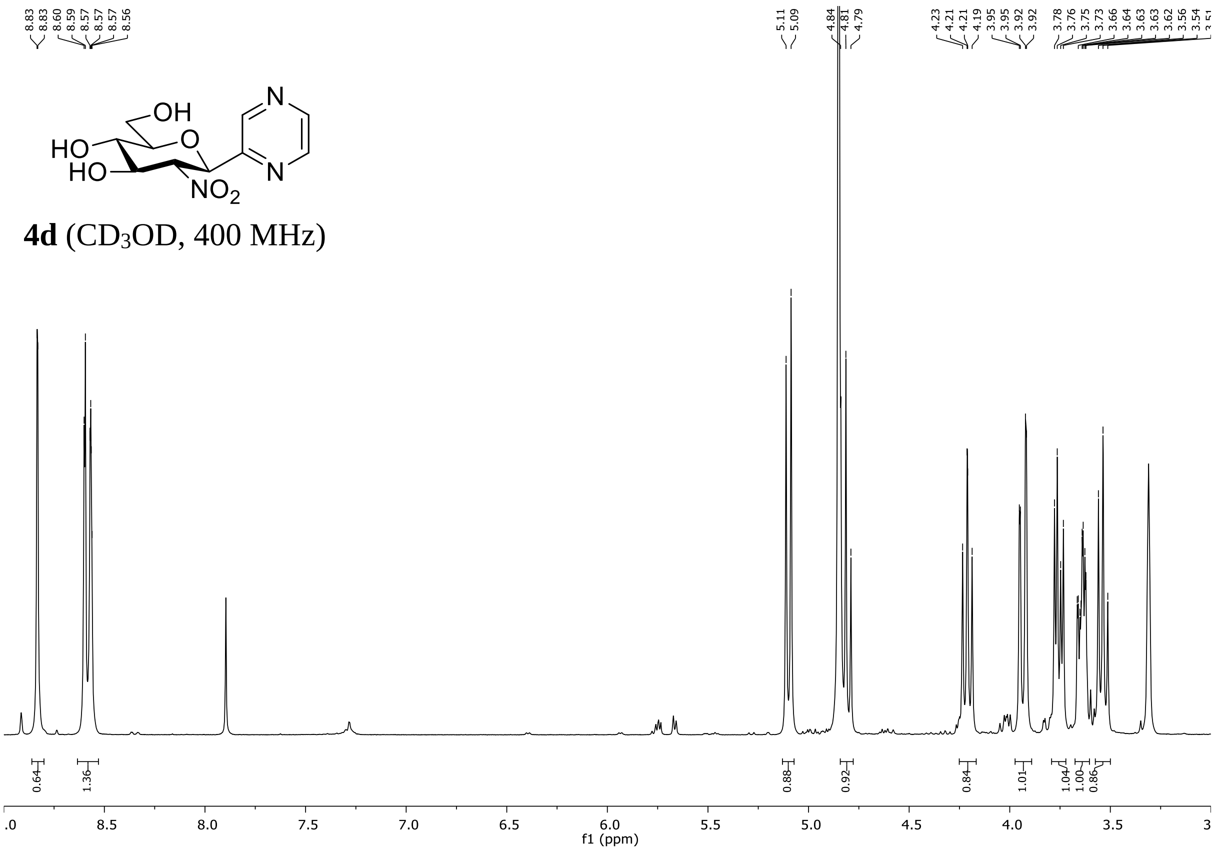
8.83 8.83 8.60 8.59 8.57 8.57 8.57 8.56
5.11 5.09
4.84 4.81 4.79
4.23 4.21 4.21 4.19 3.95 3.95 3.92 3.92
3.78 3.76 3.75 3.73 3.66 3.64 3.63 3.63 3.62 3.56 3.54
OH
HO
O
HO
NO2
N
N
4d (CD3OD, 400 MHz)
0.64 1.36 0.88 0.92 0.84 1.01 1.04 1.00 0.86
8.5 8.0 7.5 7.0 6.5 6.0 5.5 5.0 4.5 4.0 3.5
f1 (ppm)

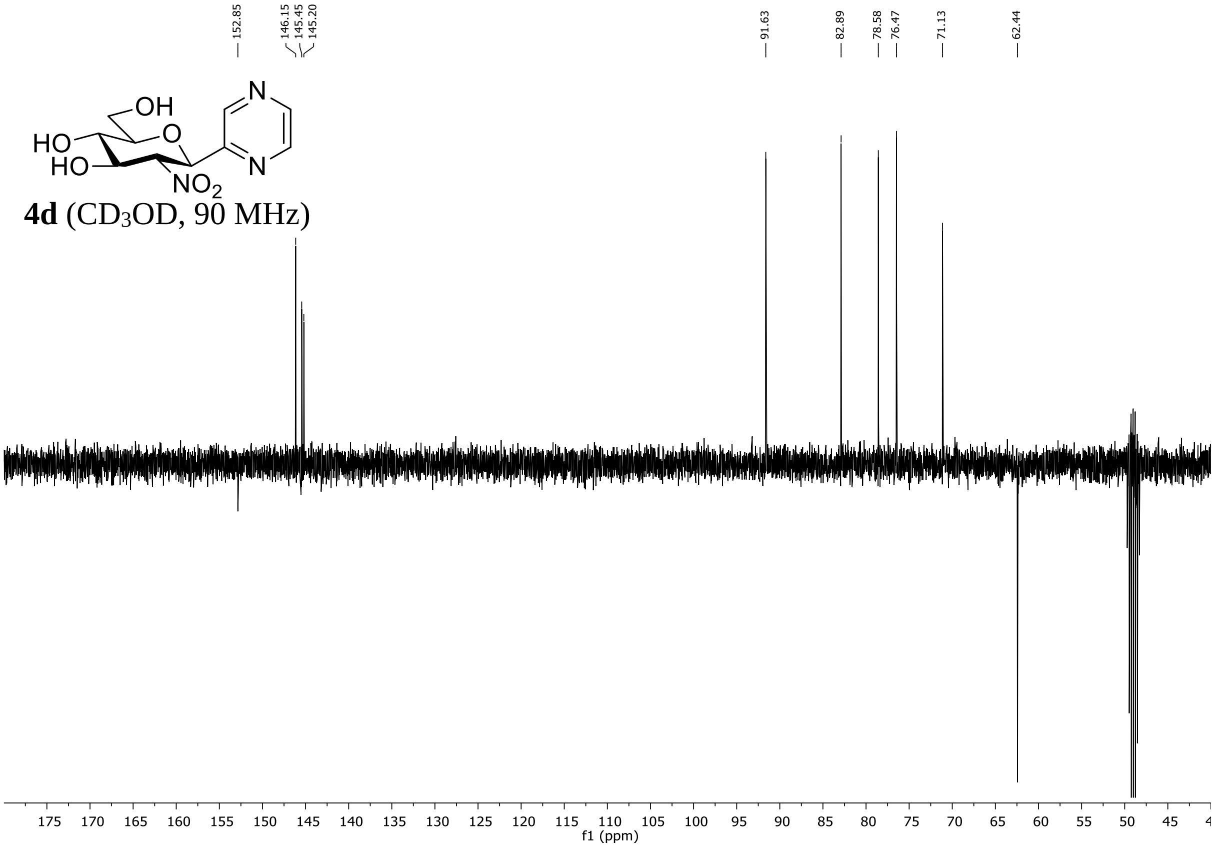
152.85
146.15 145.45 145.20
91.63
82.89
78.58
76.47
71.13
62.44
OH
HO
O
HO
NO2
N
N
4d (CD3OD, 90 MHz)
175 170 165 160 155 150 145 140 135 130 125 120 115 110 105 100 95 90 85 80 75 70 65 60 55 50 45
f1 (ppm)

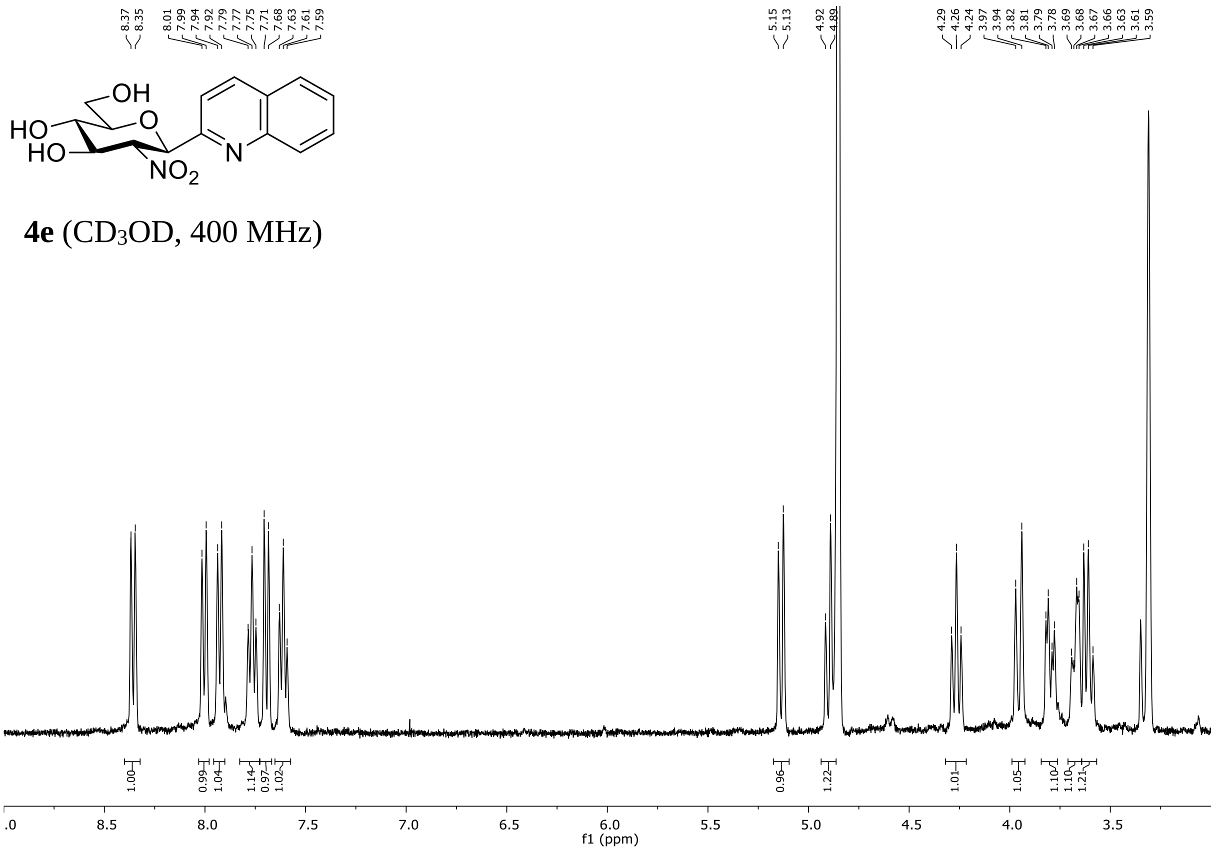

4e (CD_3OD, 400 MHz)

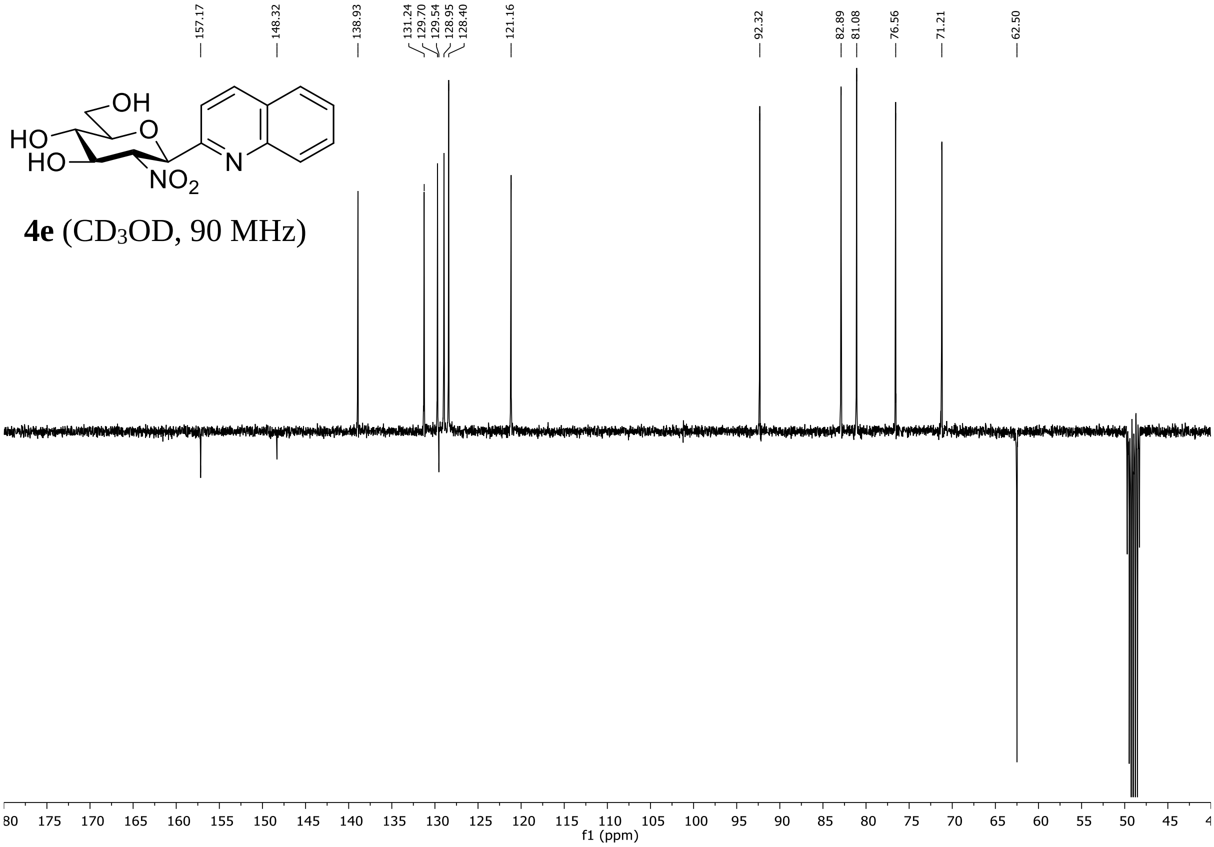

4e (CD_3OD, 90 MHz)

5a (CD_3OD, 400 MHz)

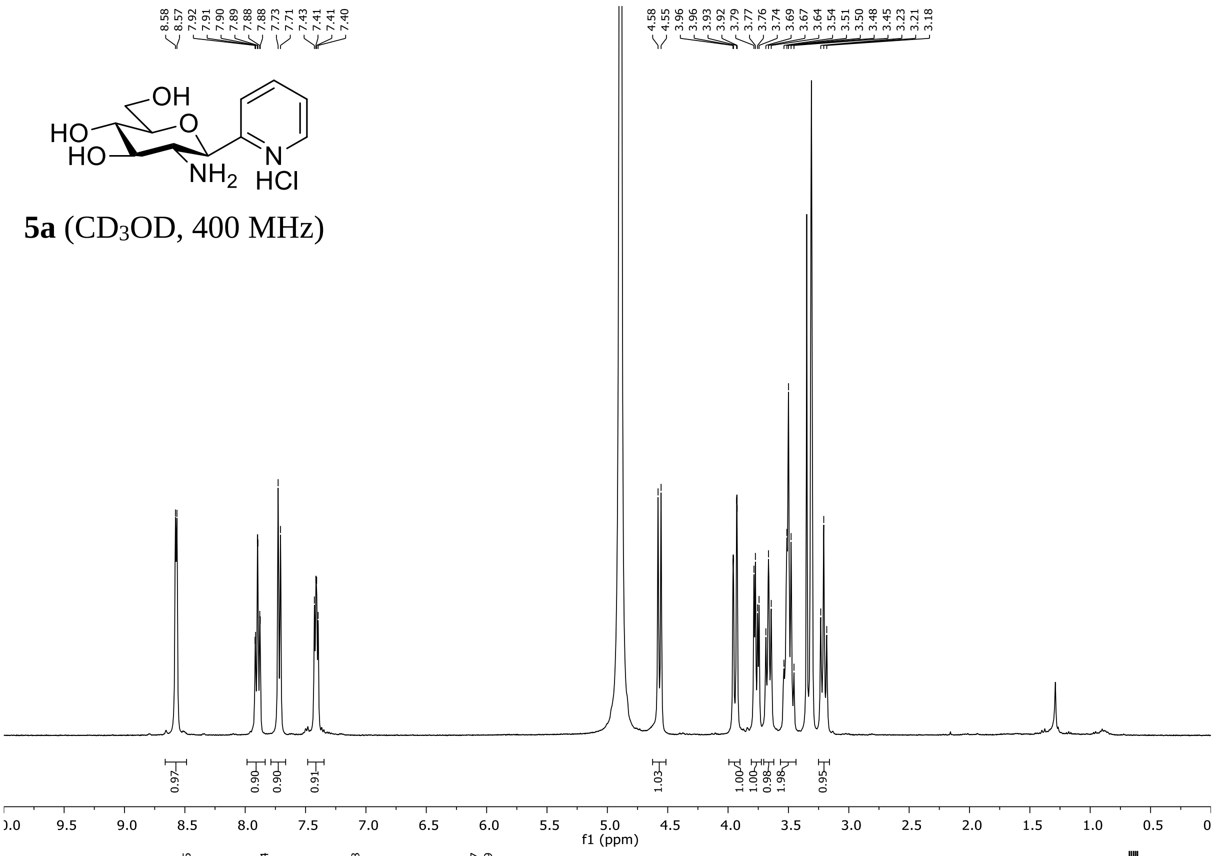

5a (CD_3OD, 90 MHz)

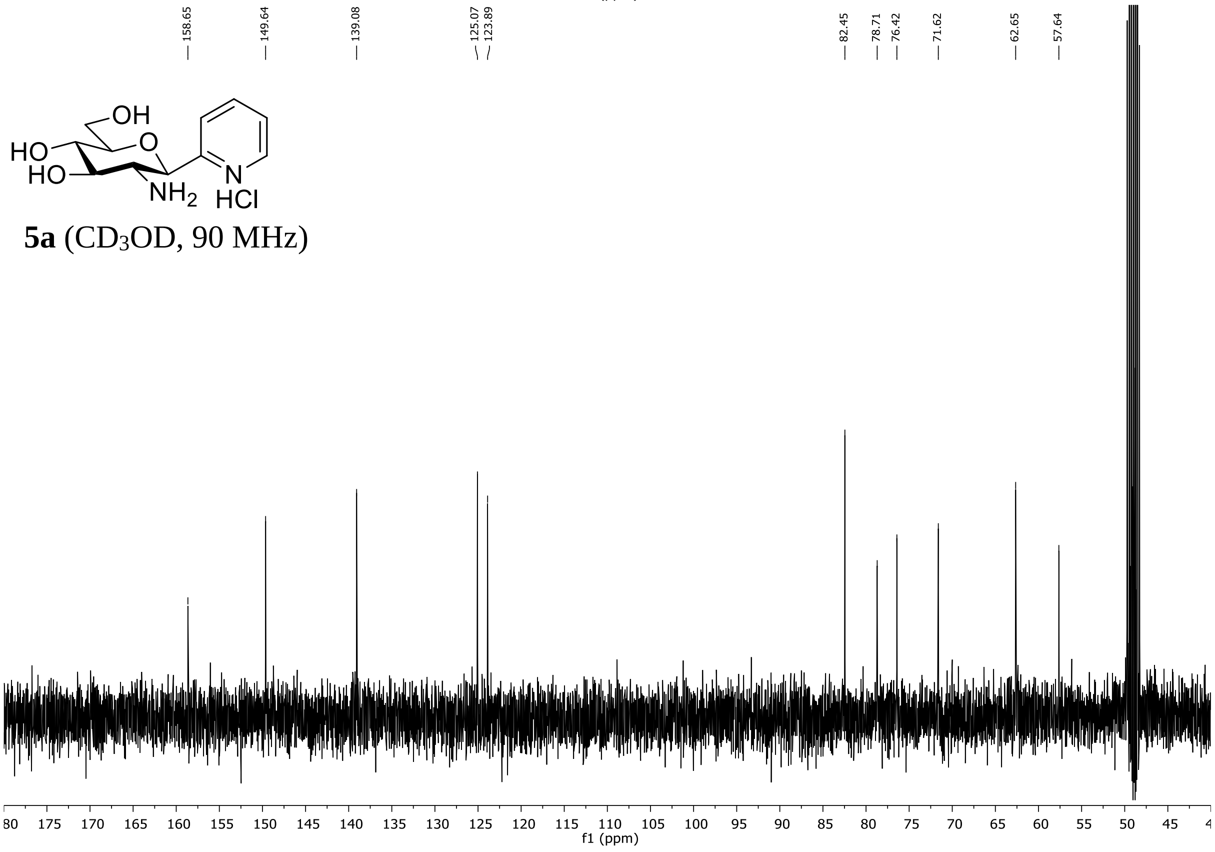

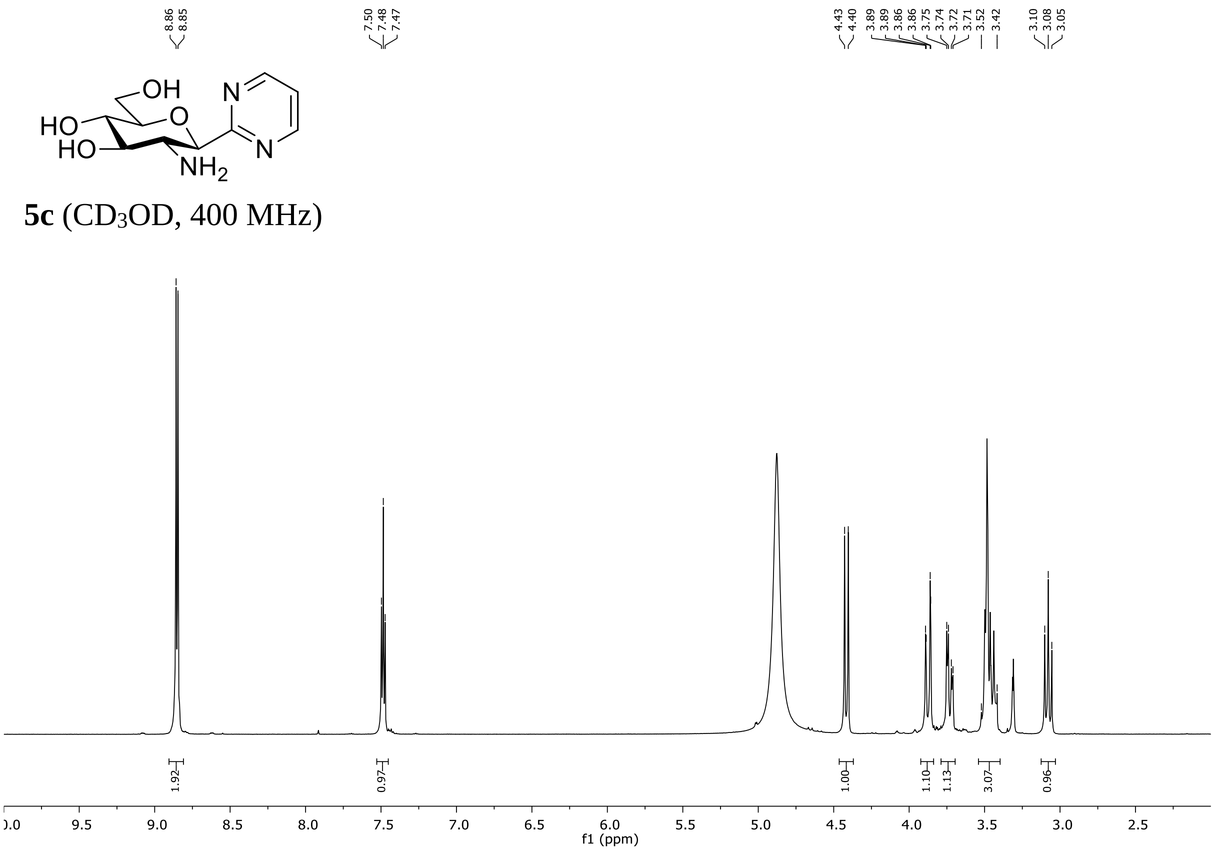

5c (CD_3OD, 400 MHz)

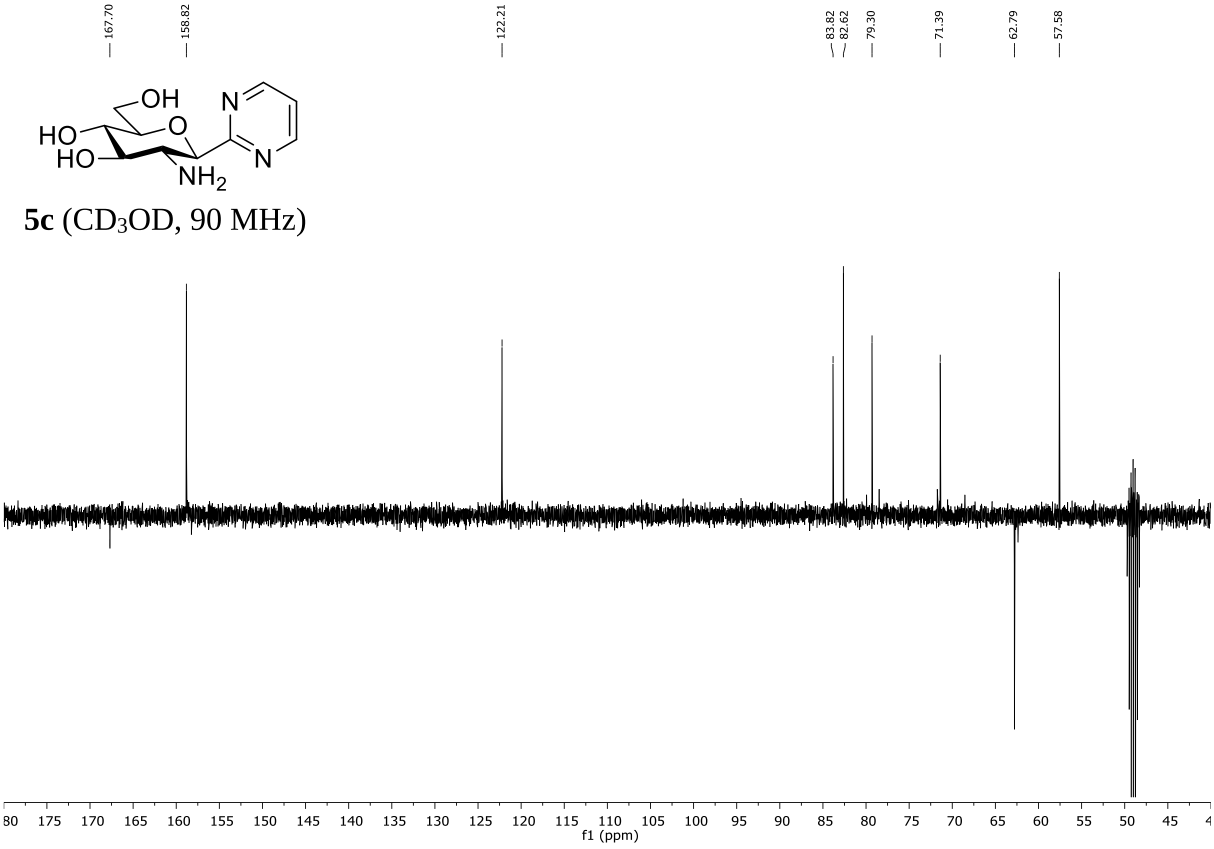

5c (CD_3OD, 90 MHz)

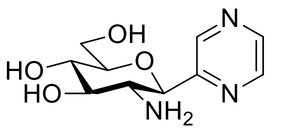

5d (CD3OD, 400 MHz)

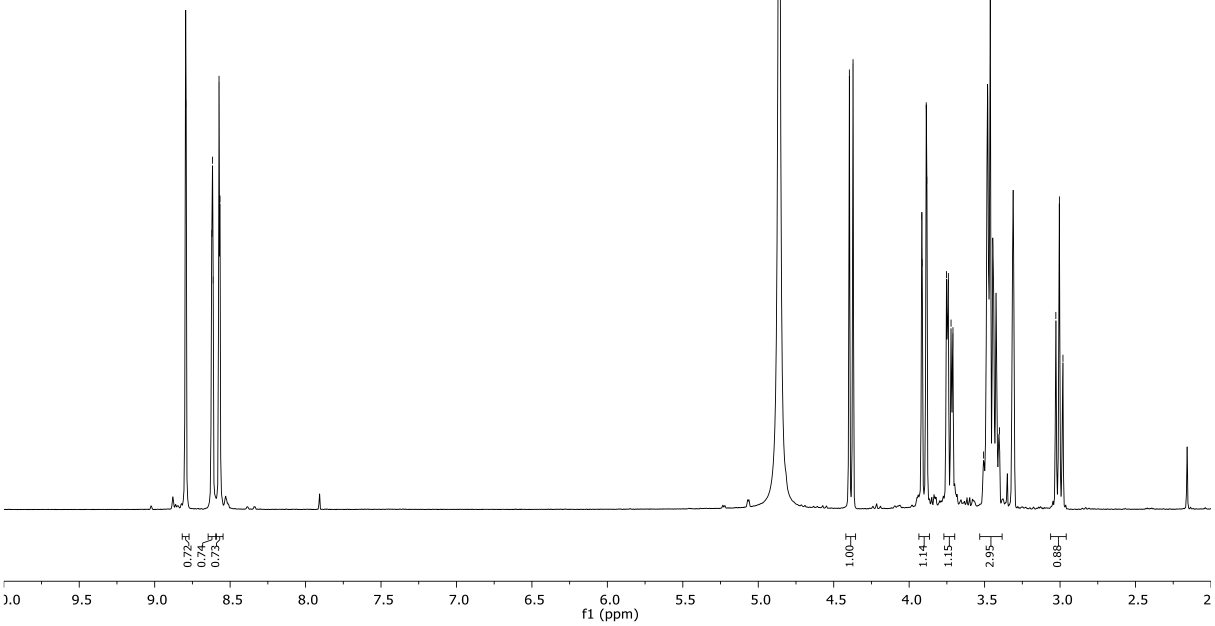

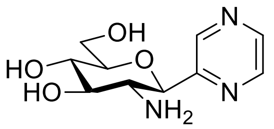

5d (CD3OD, 90 MHz)

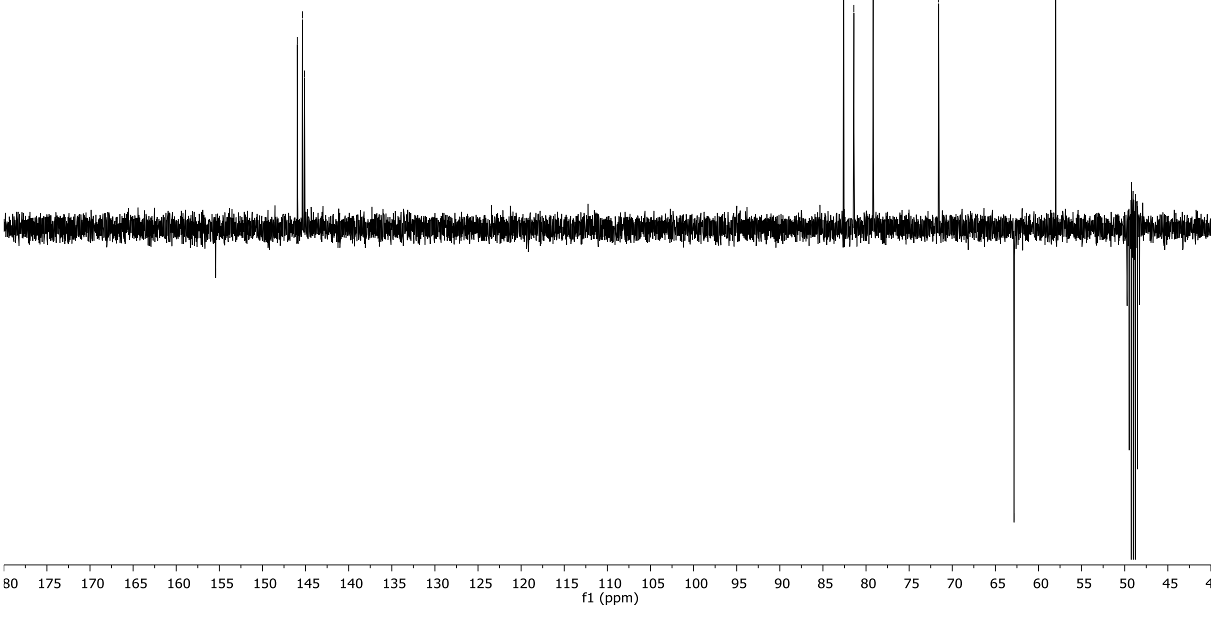

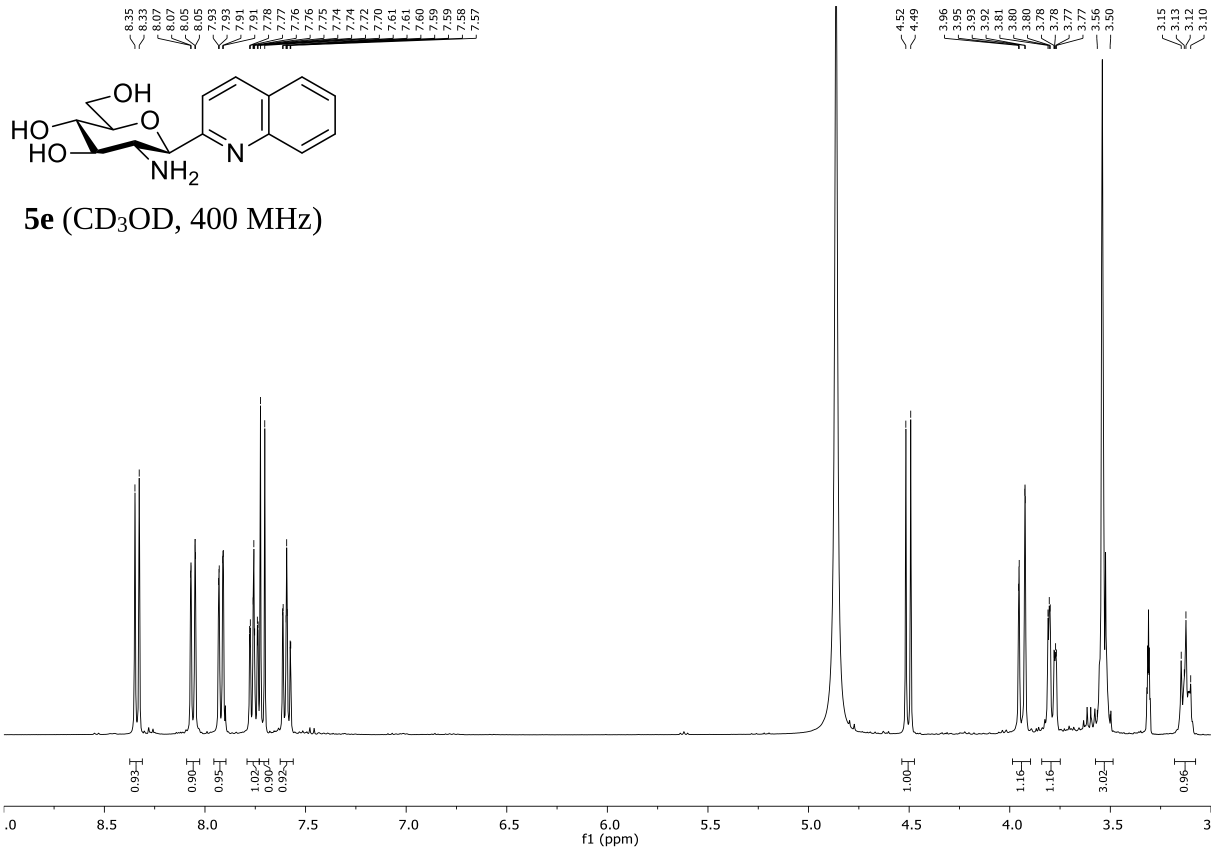

5e (CD_3OD, 400 MHz)

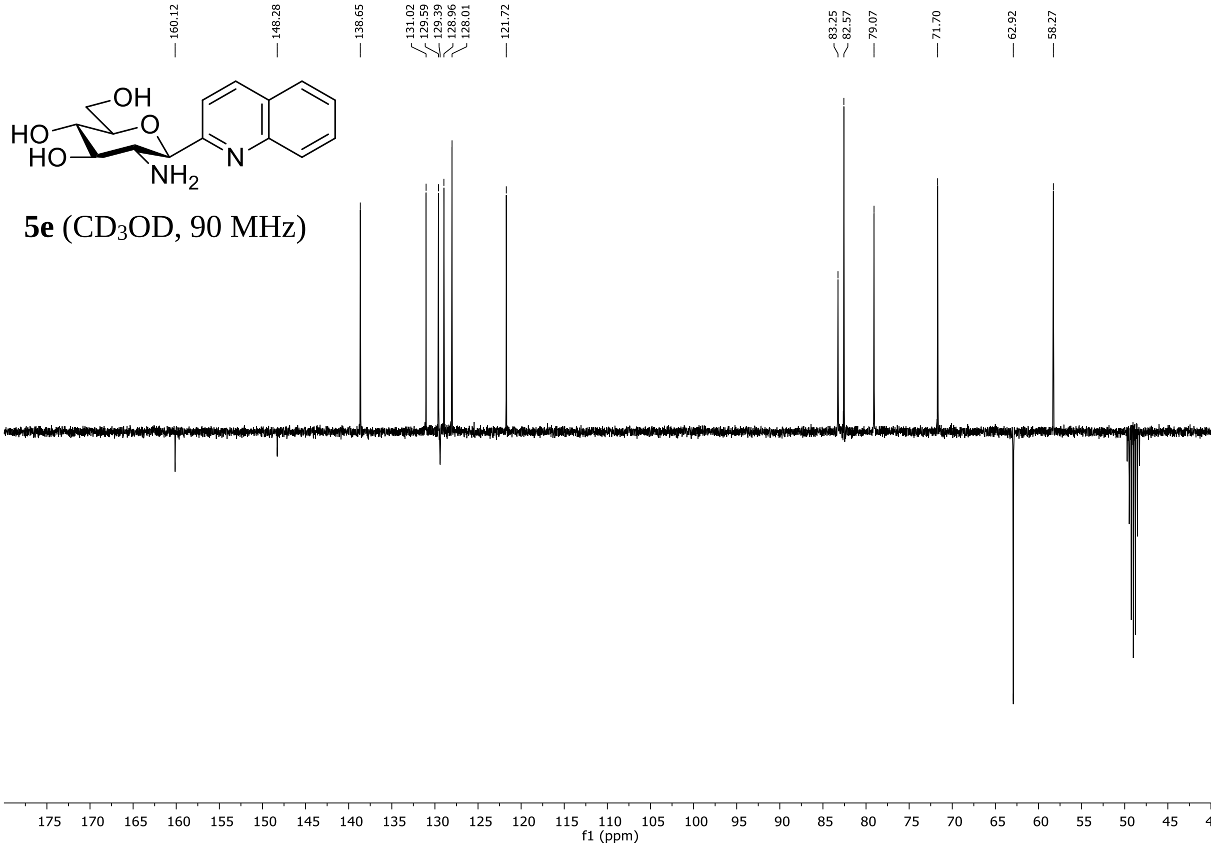

5e (CD_3OD, 90 MHz)

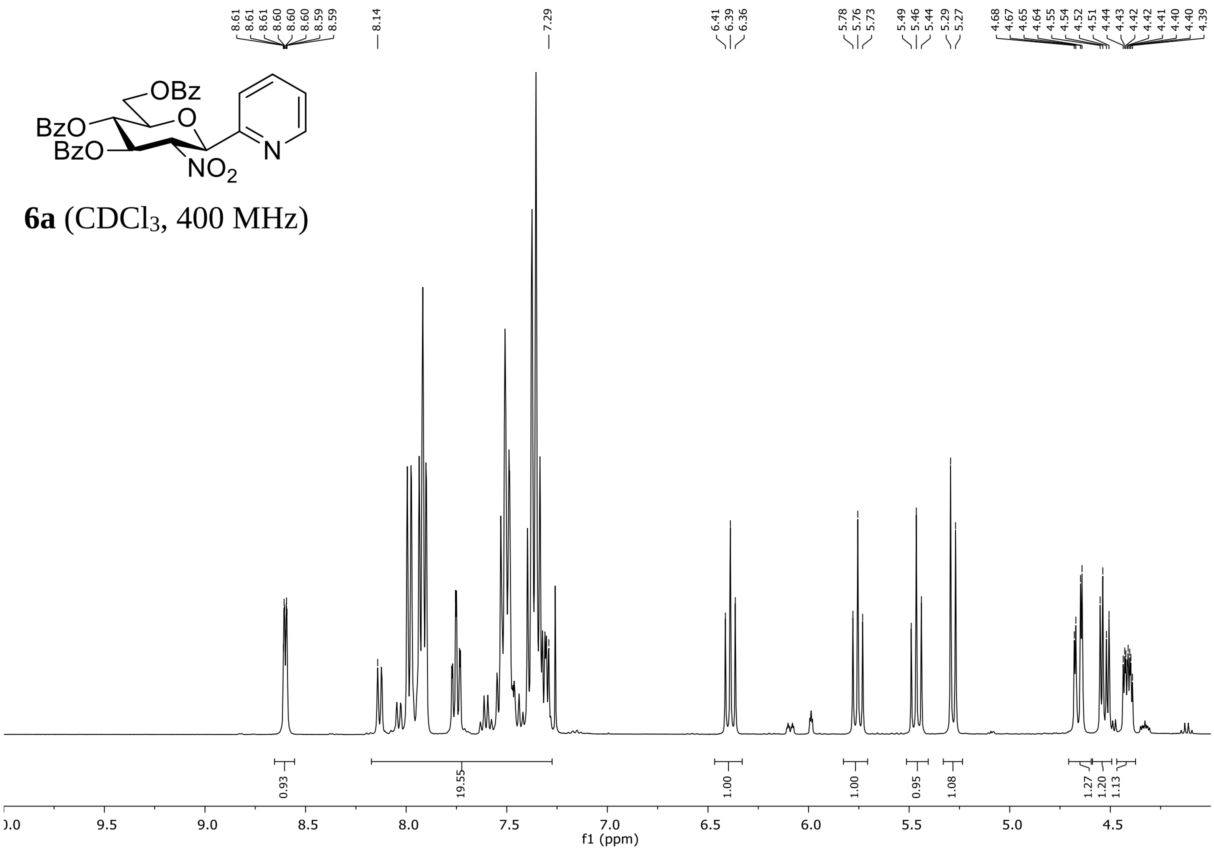

6a ($CDCl_3$, 400 MHz)

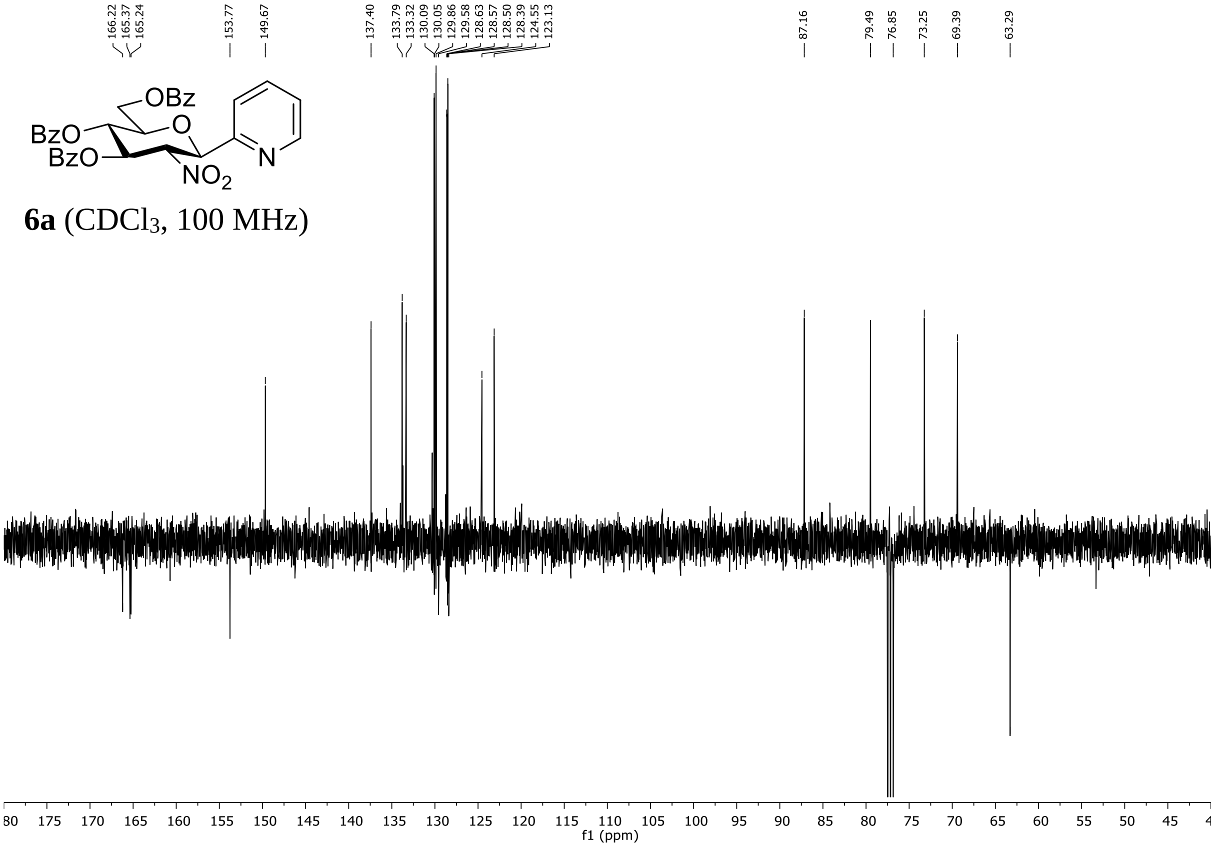

6a ($CDCl_3$, 100 MHz)

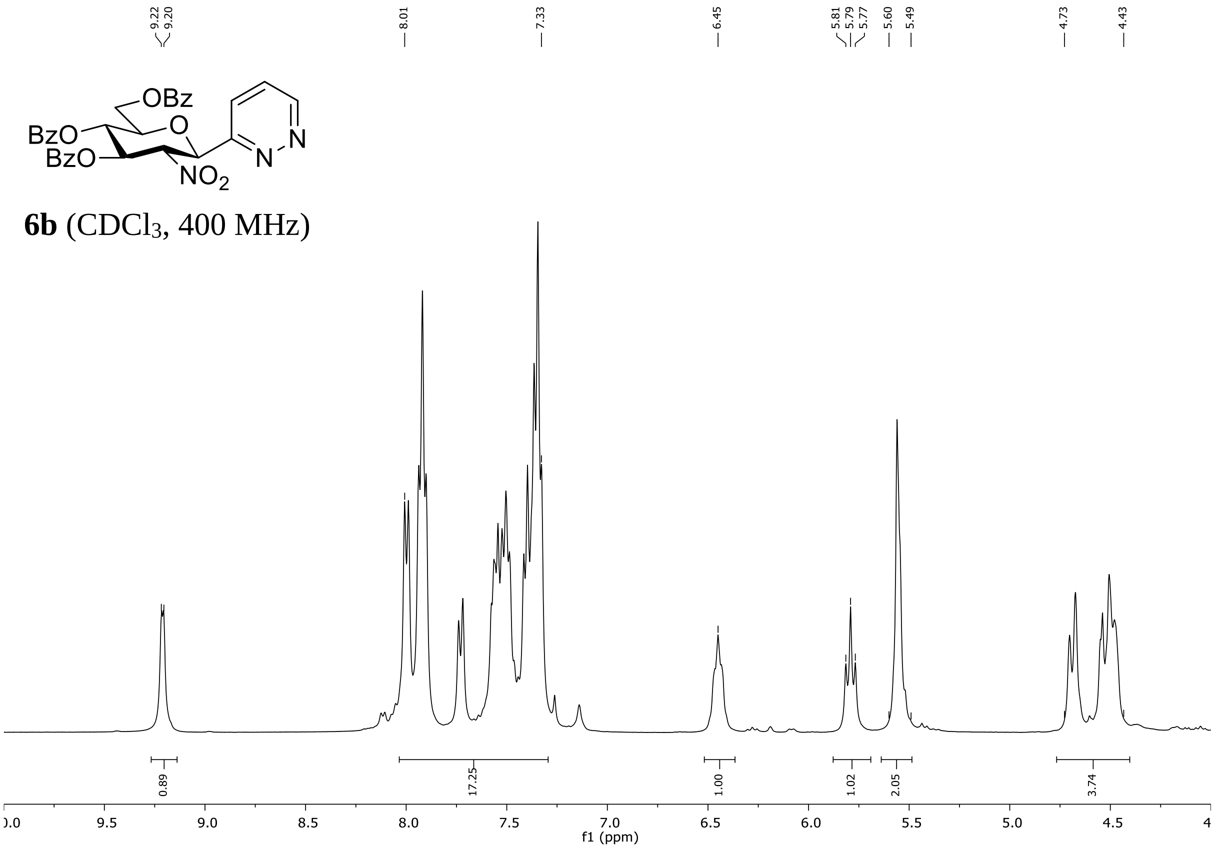

6b (CDCl$_3$, 400 MHz)

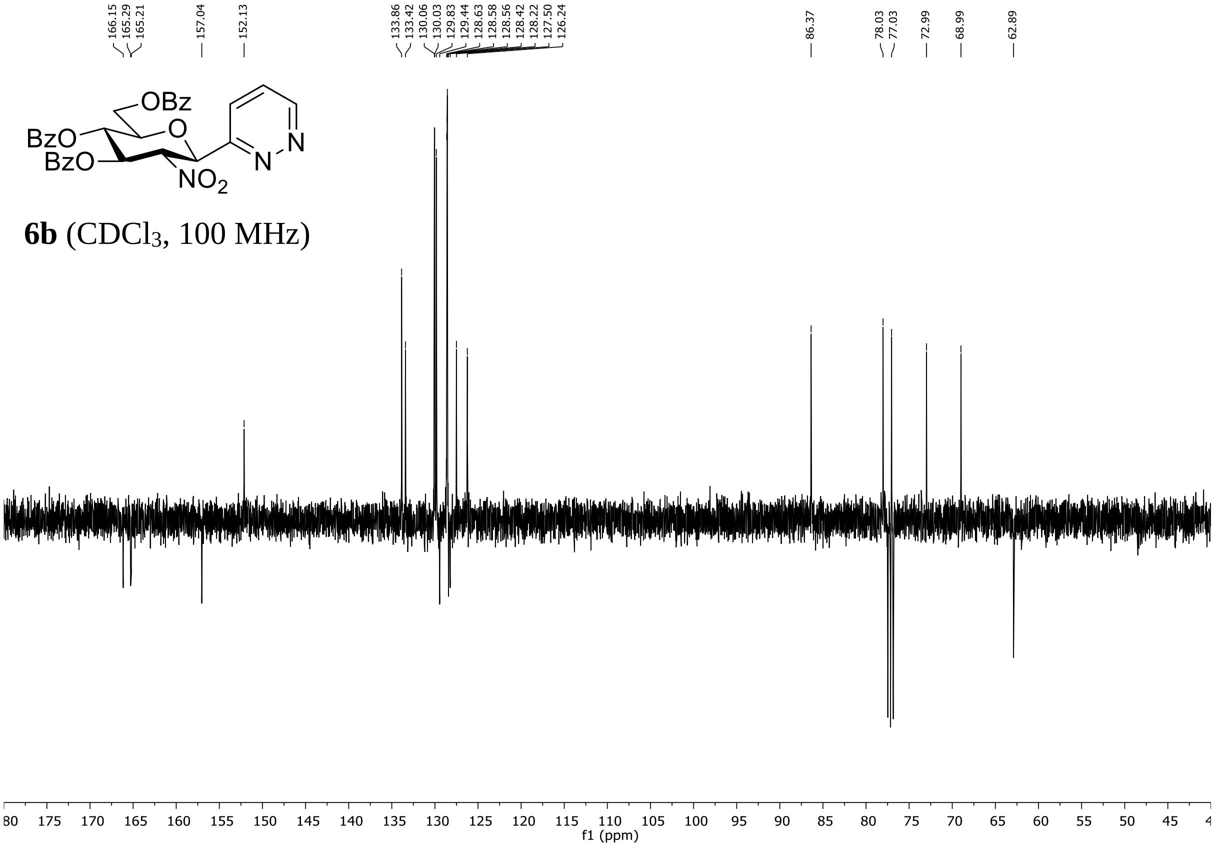

6b (CDCl$_3$, 100 MHz)

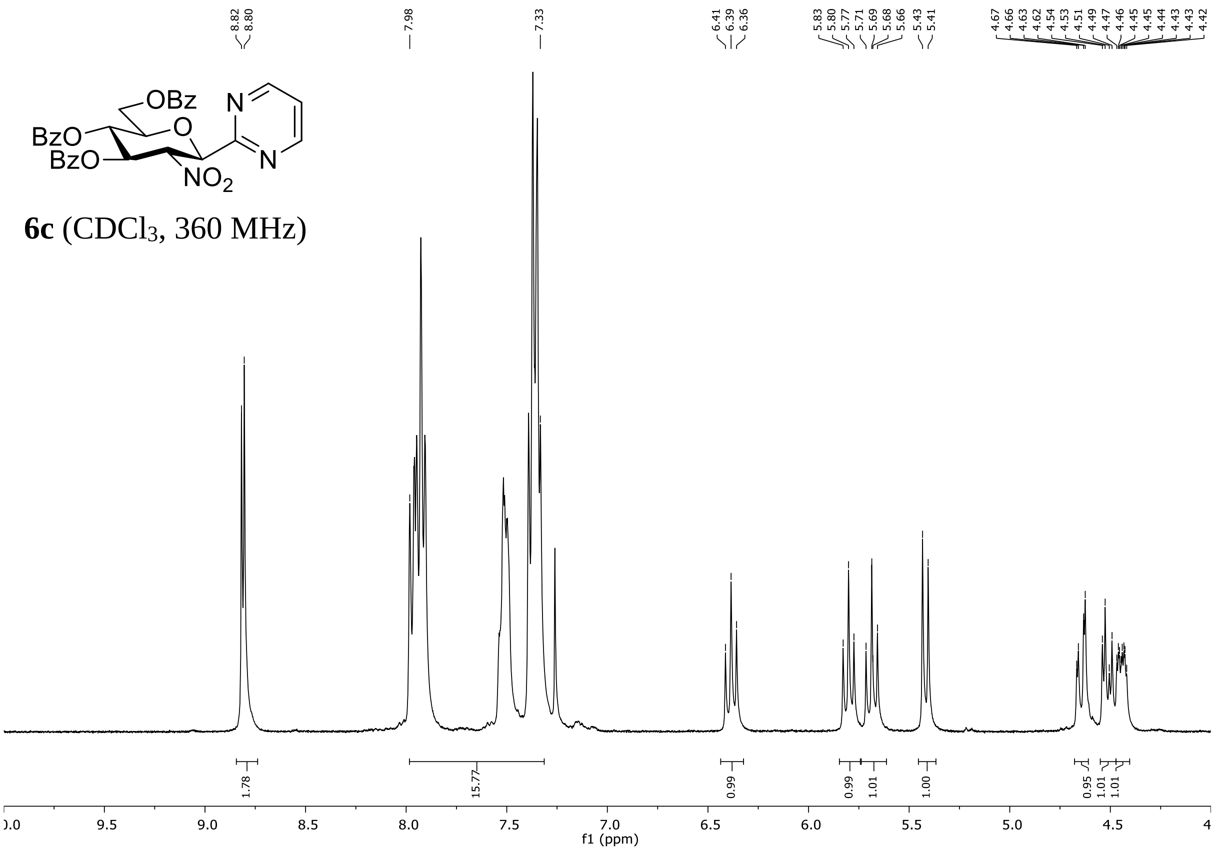

6c ($CDCl_3$, 360 MHz)

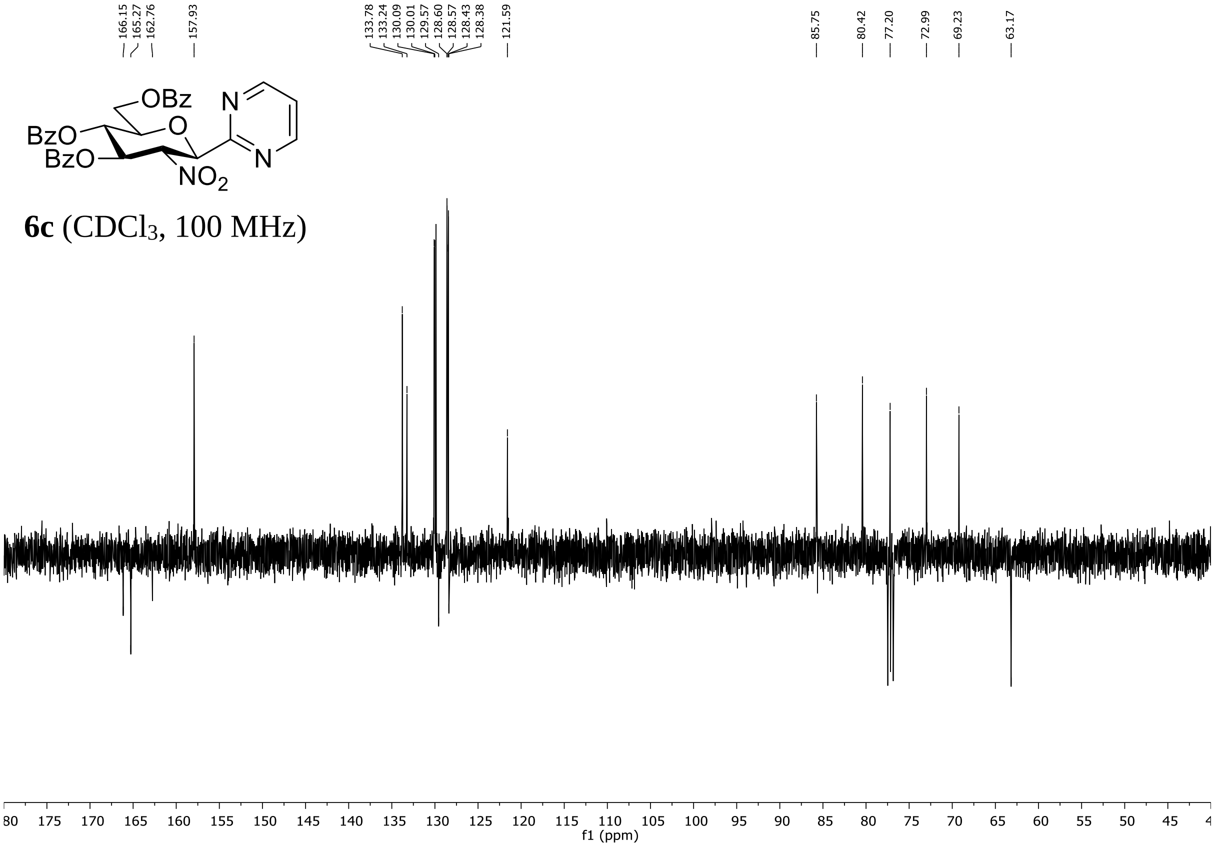

6c ($CDCl_3$, 100 MHz)

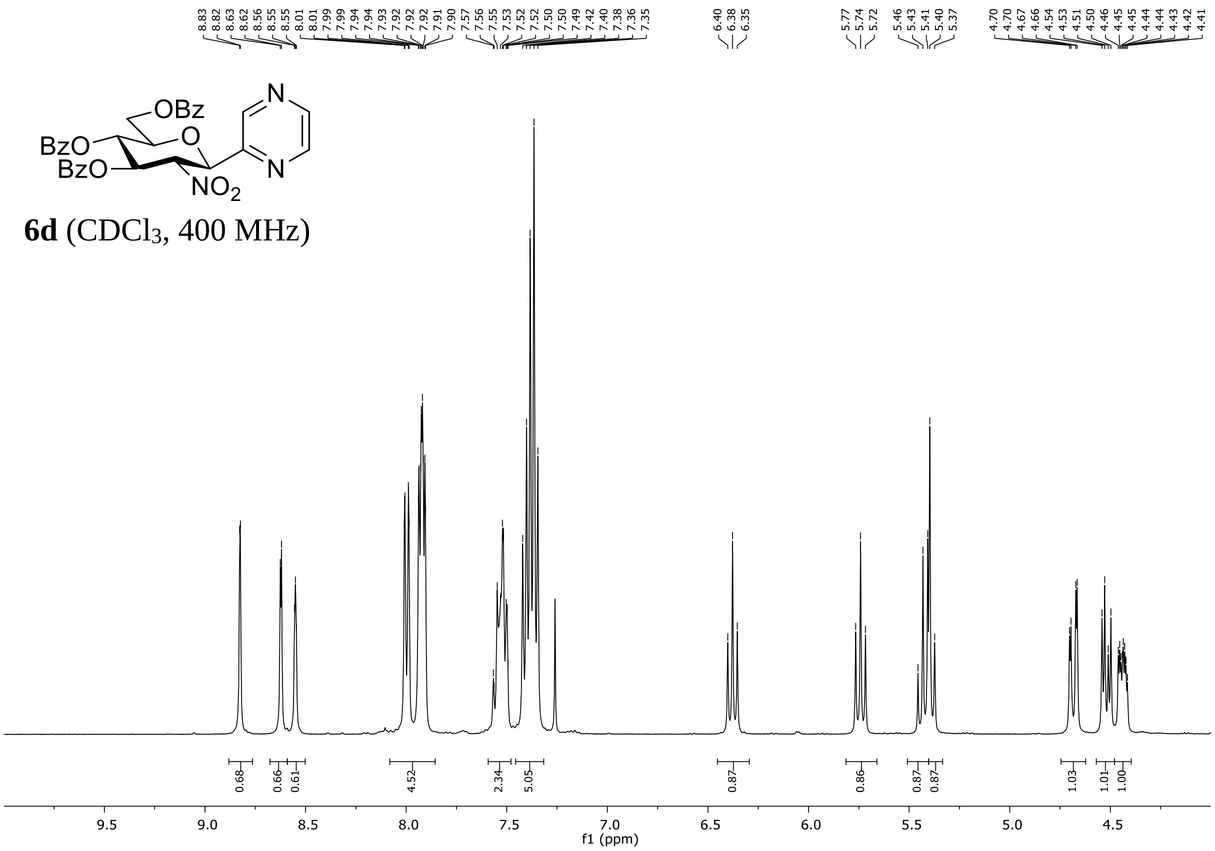

6d ($CDCl_3$, 400 MHz)

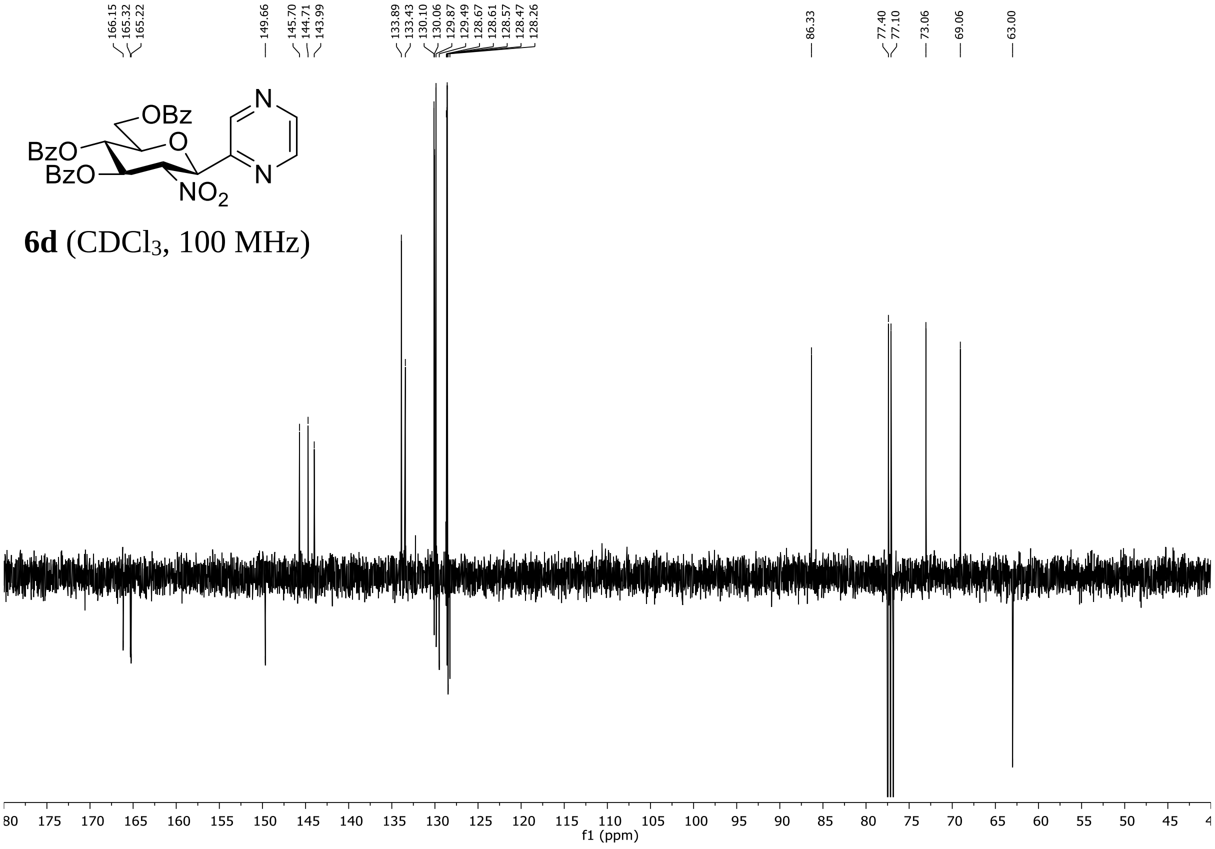

6d ($CDCl_3$, 100 MHz)

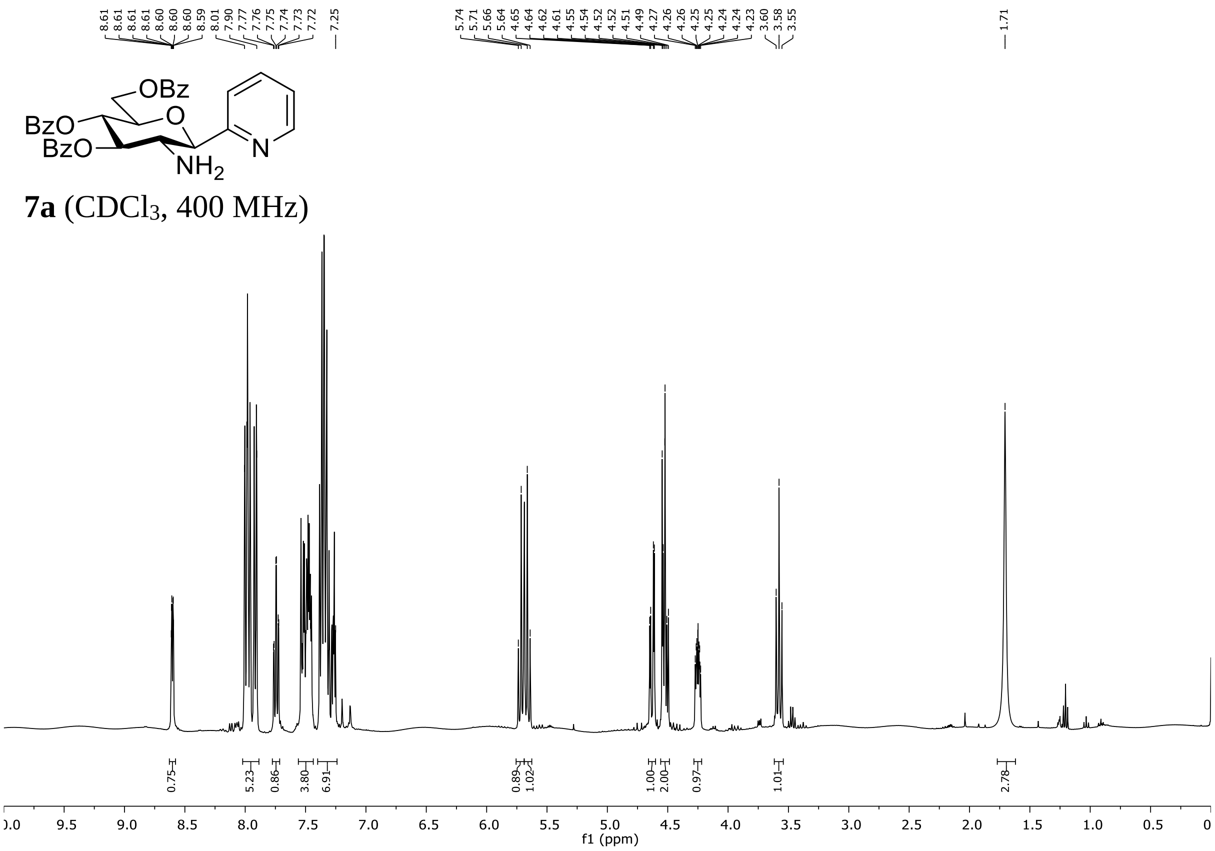

7a ($CDCl_3$, 400 MHz)

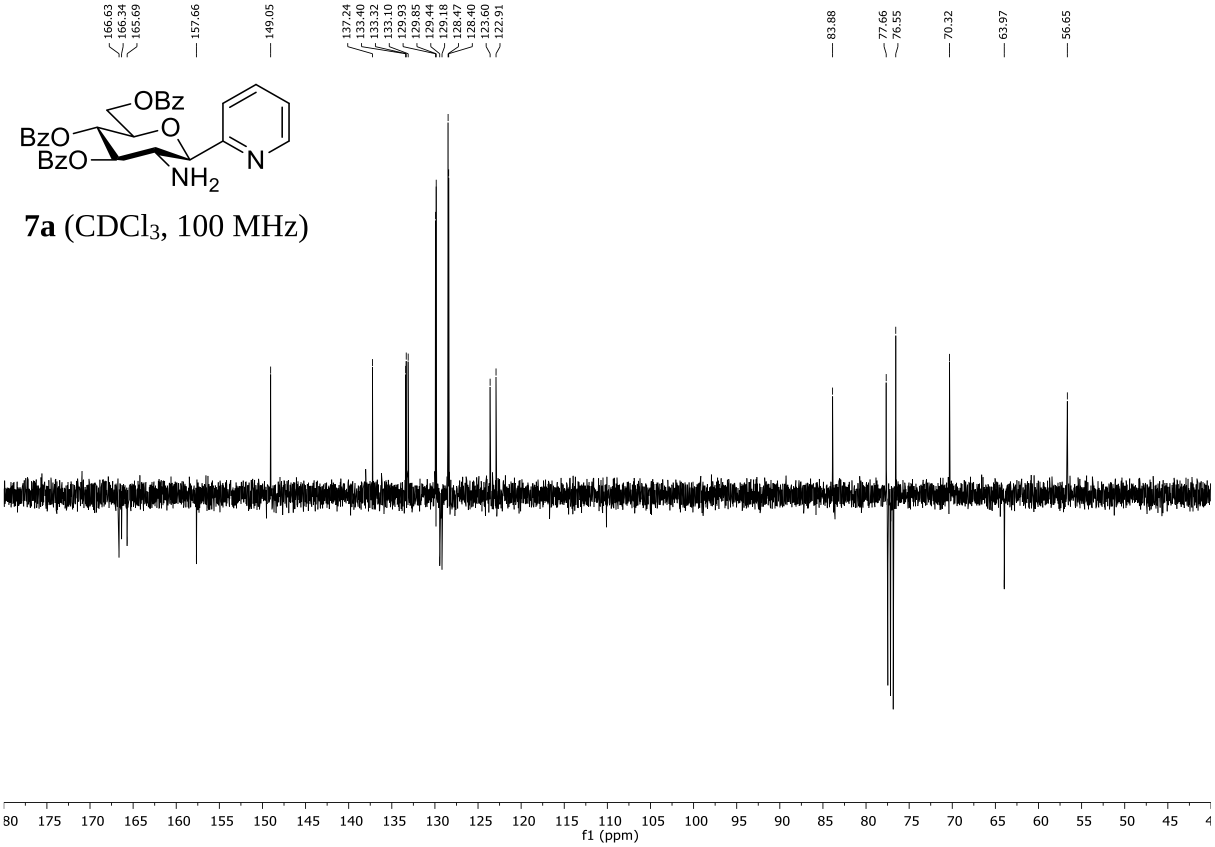

7a ($CDCl_3$, 100 MHz)

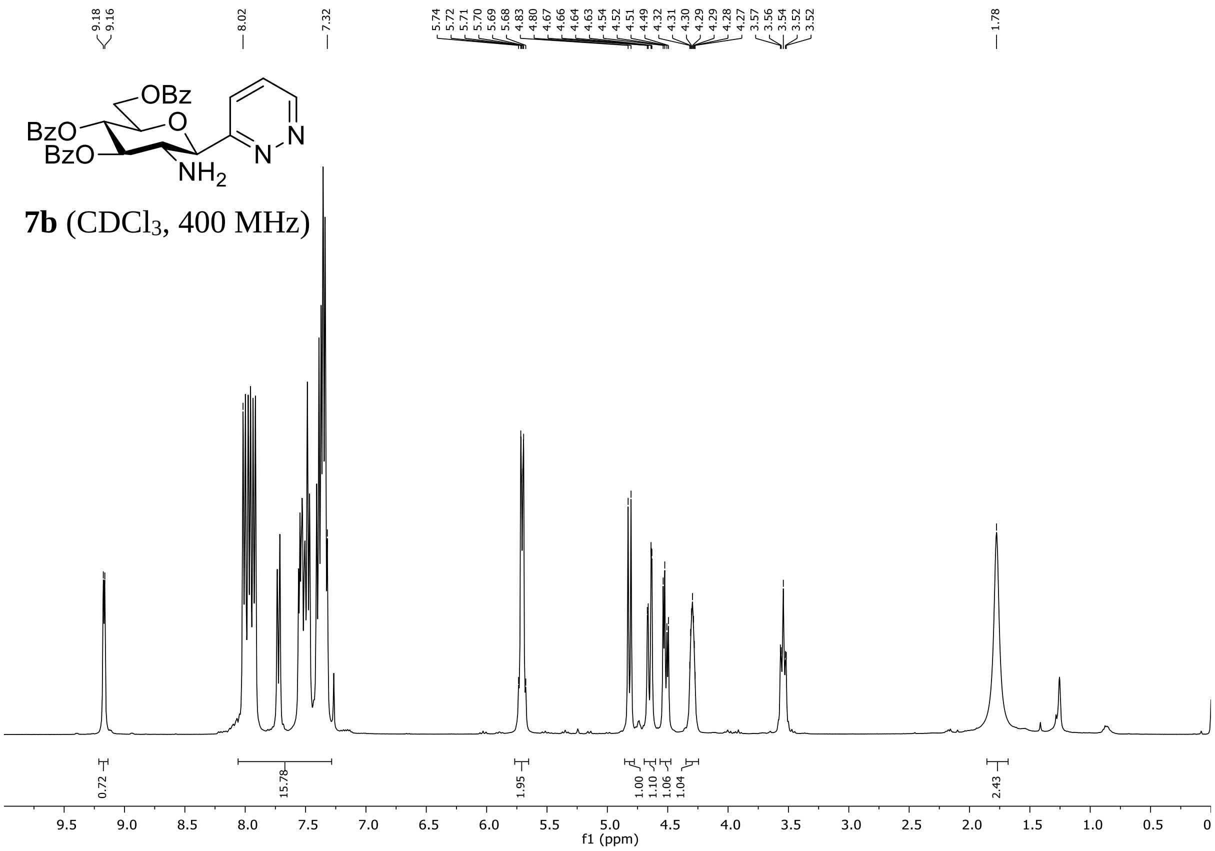

7b ($CDCl_3$, 400 MHz)

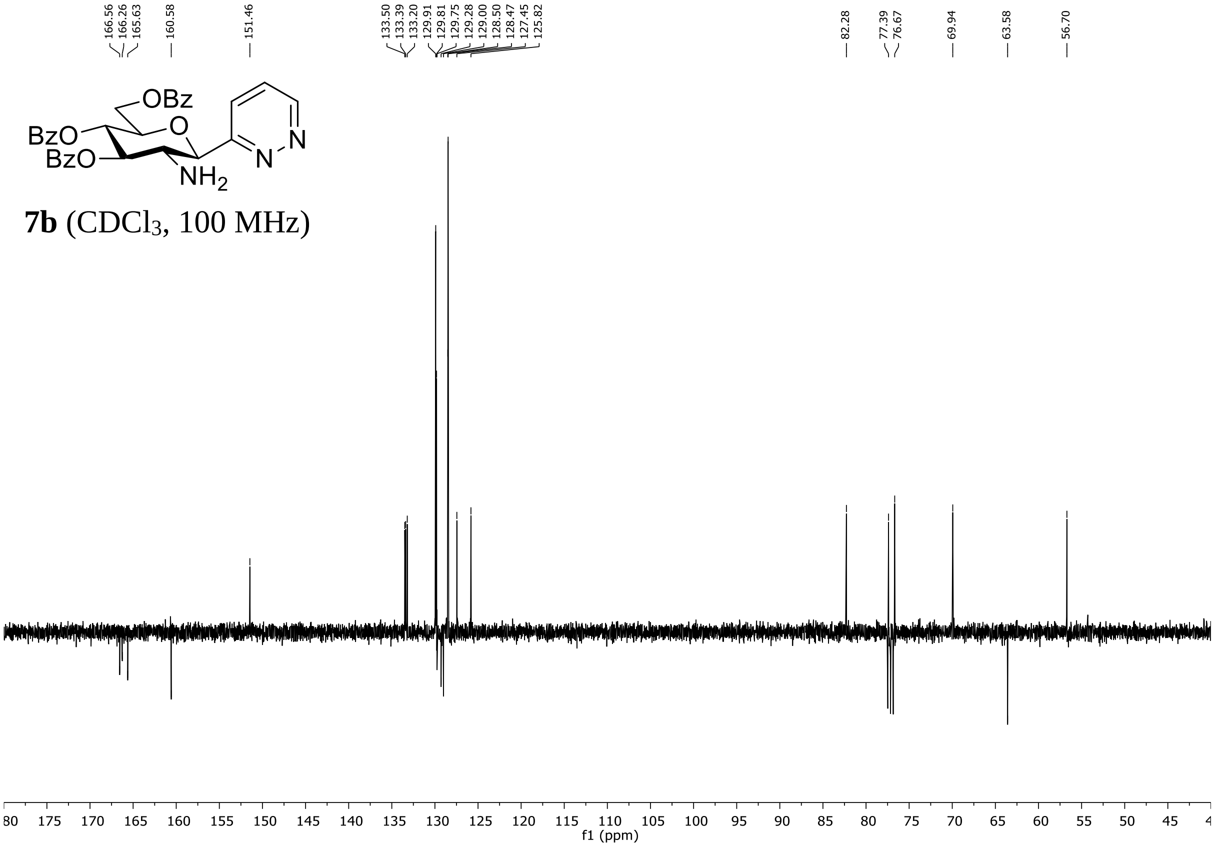

7b ($CDCl_3$, 100 MHz)

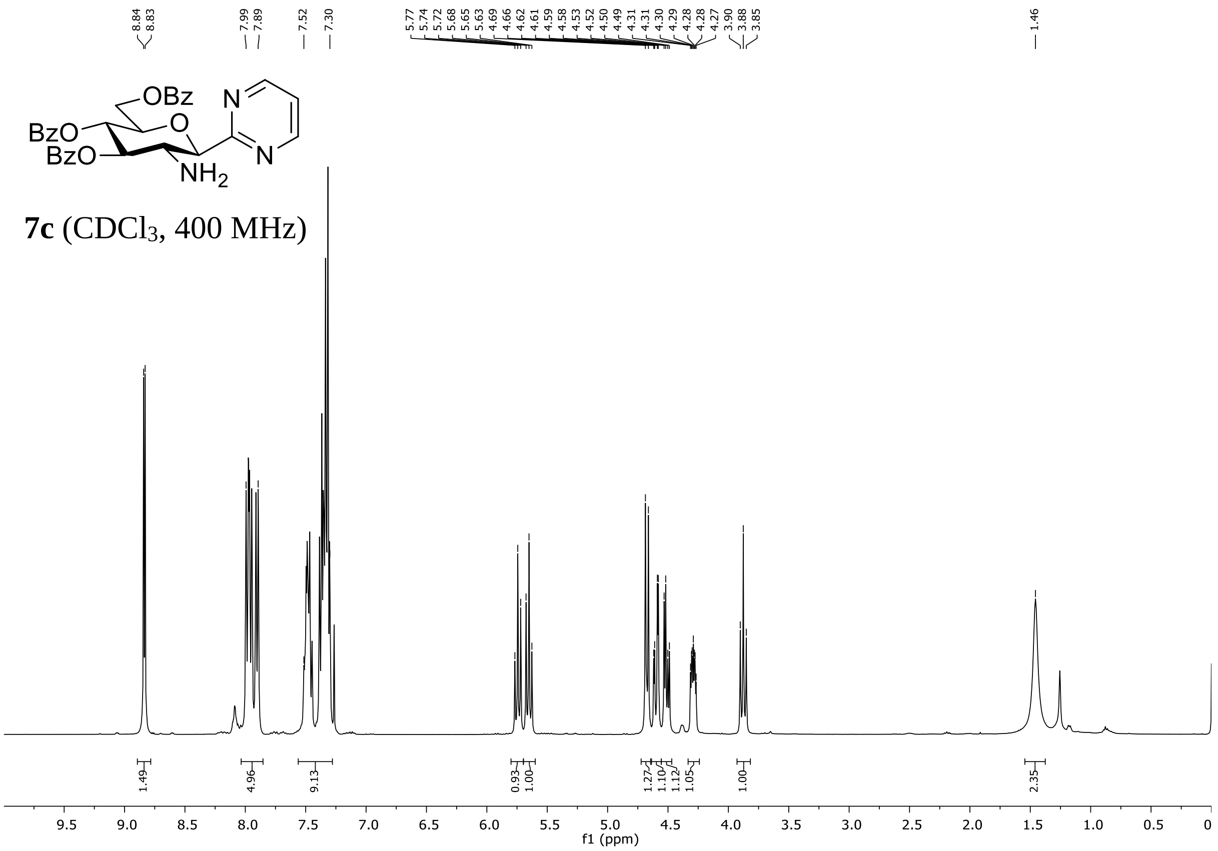
7c (CDCl3, 400 MHz)

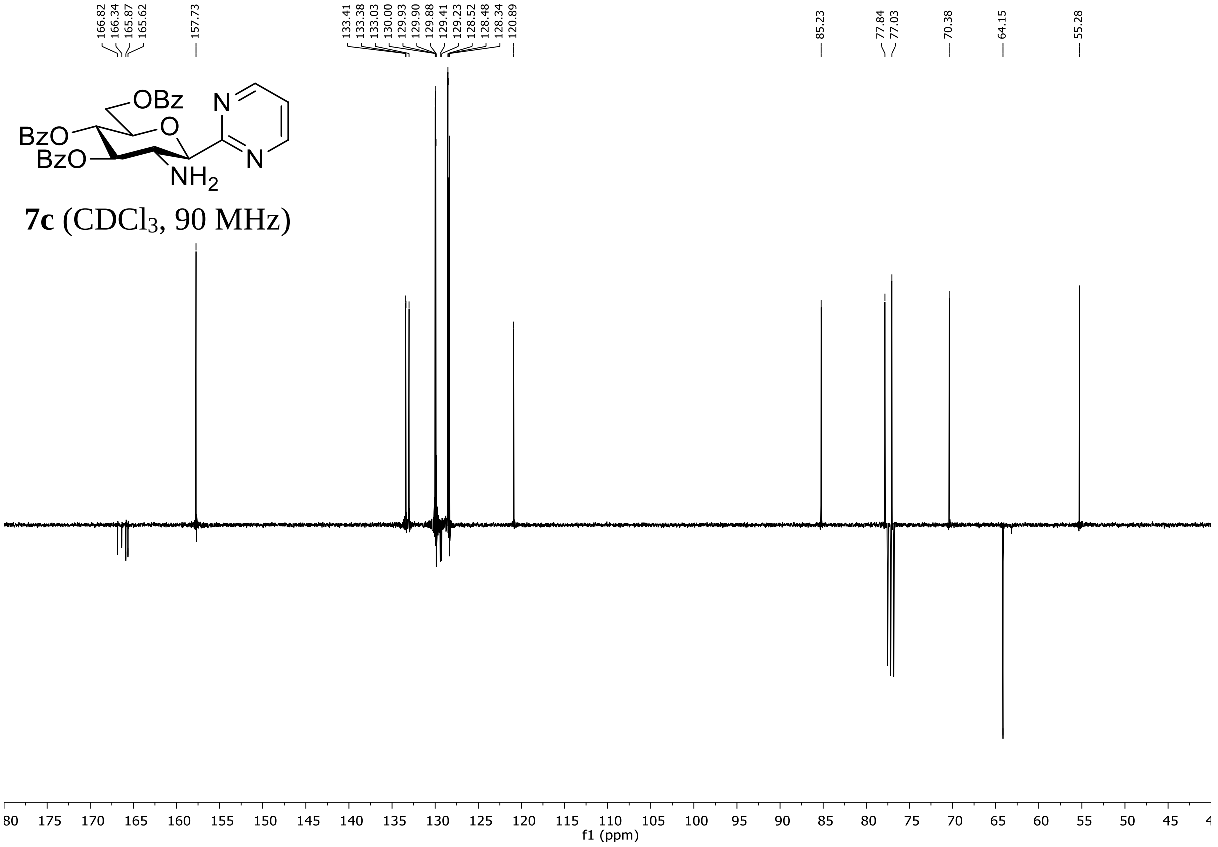
7c (CDCl3, 90 MHz)

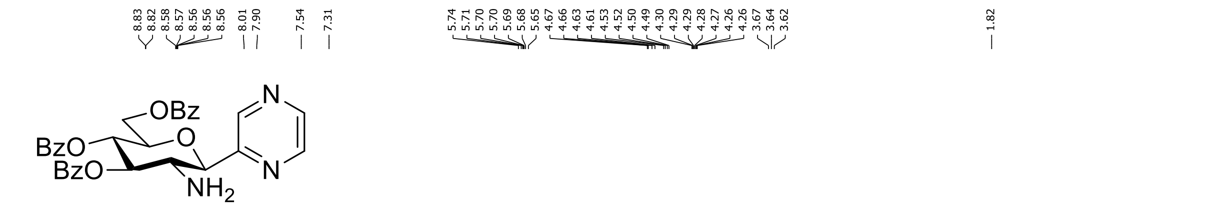

7d ($CDCl_3$, 400 MHz)

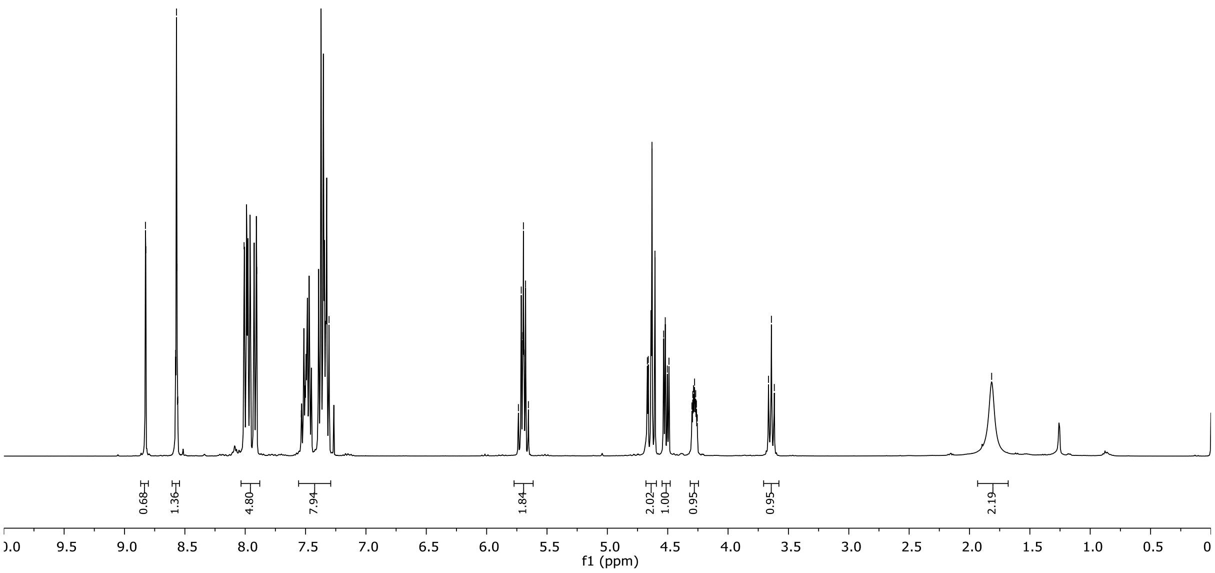

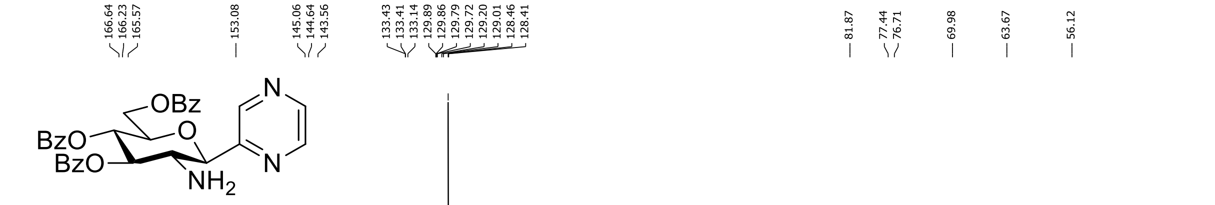

7d ($CDCl_3$, 100 MHz)

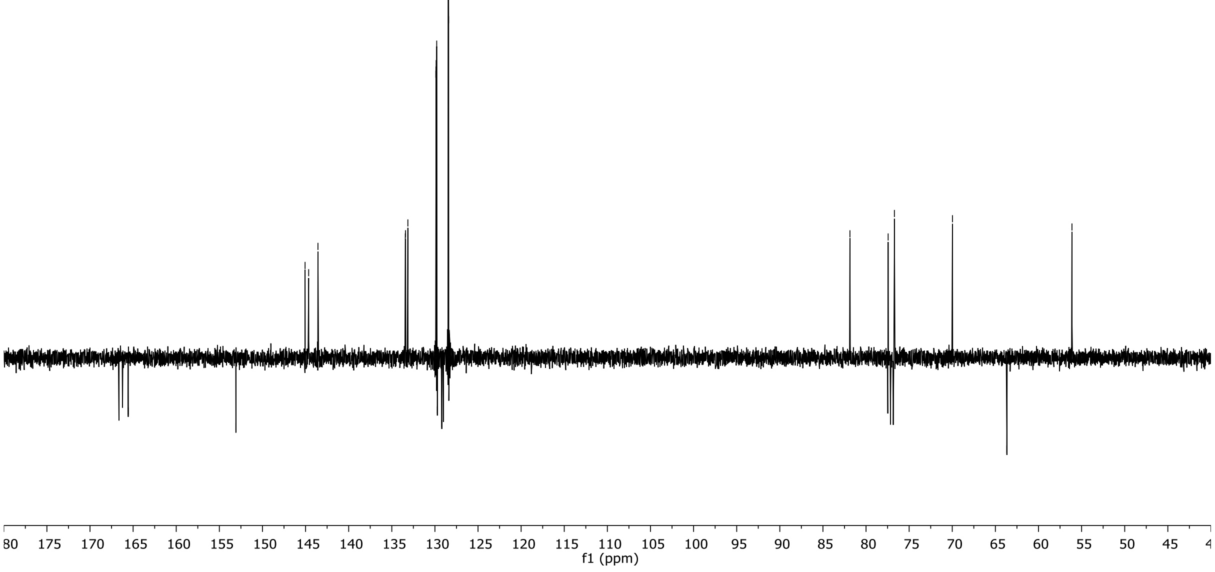

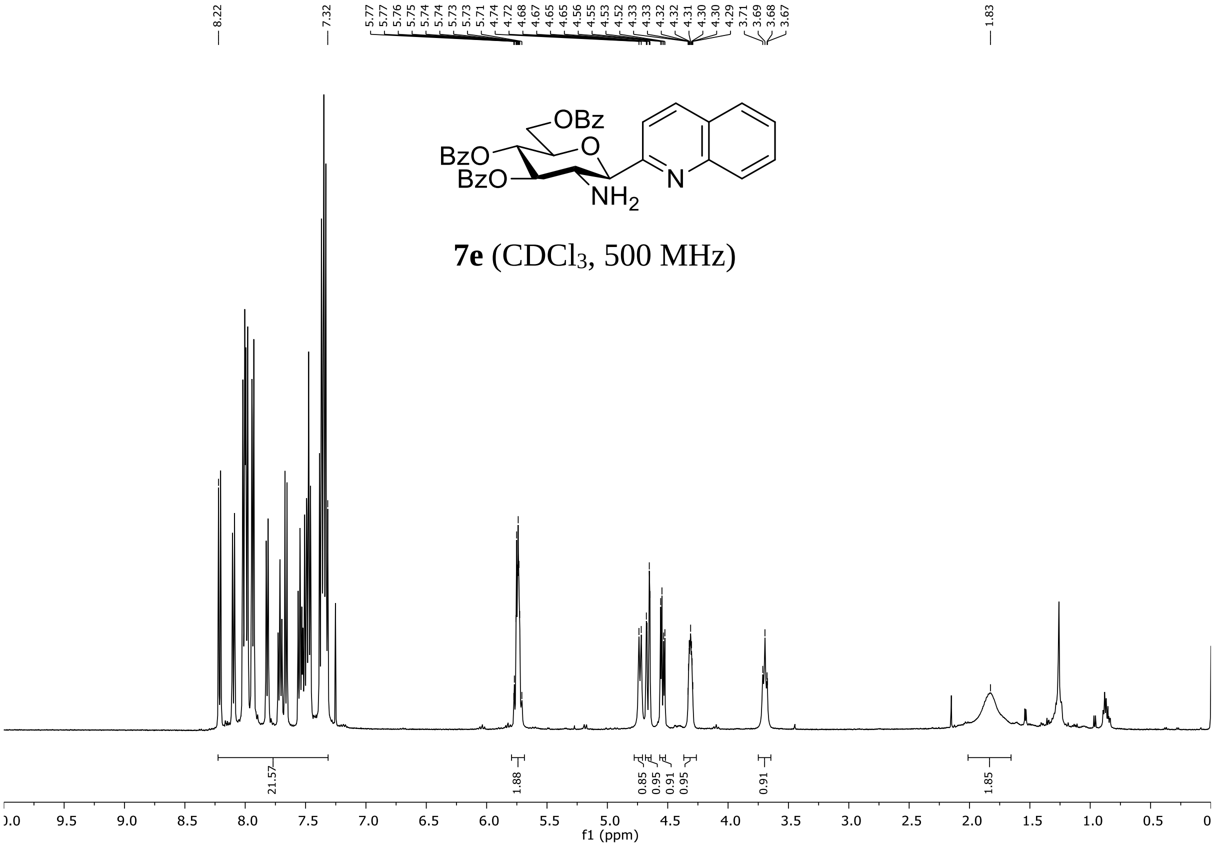

7e ($CDCl_3$, 500 MHz)

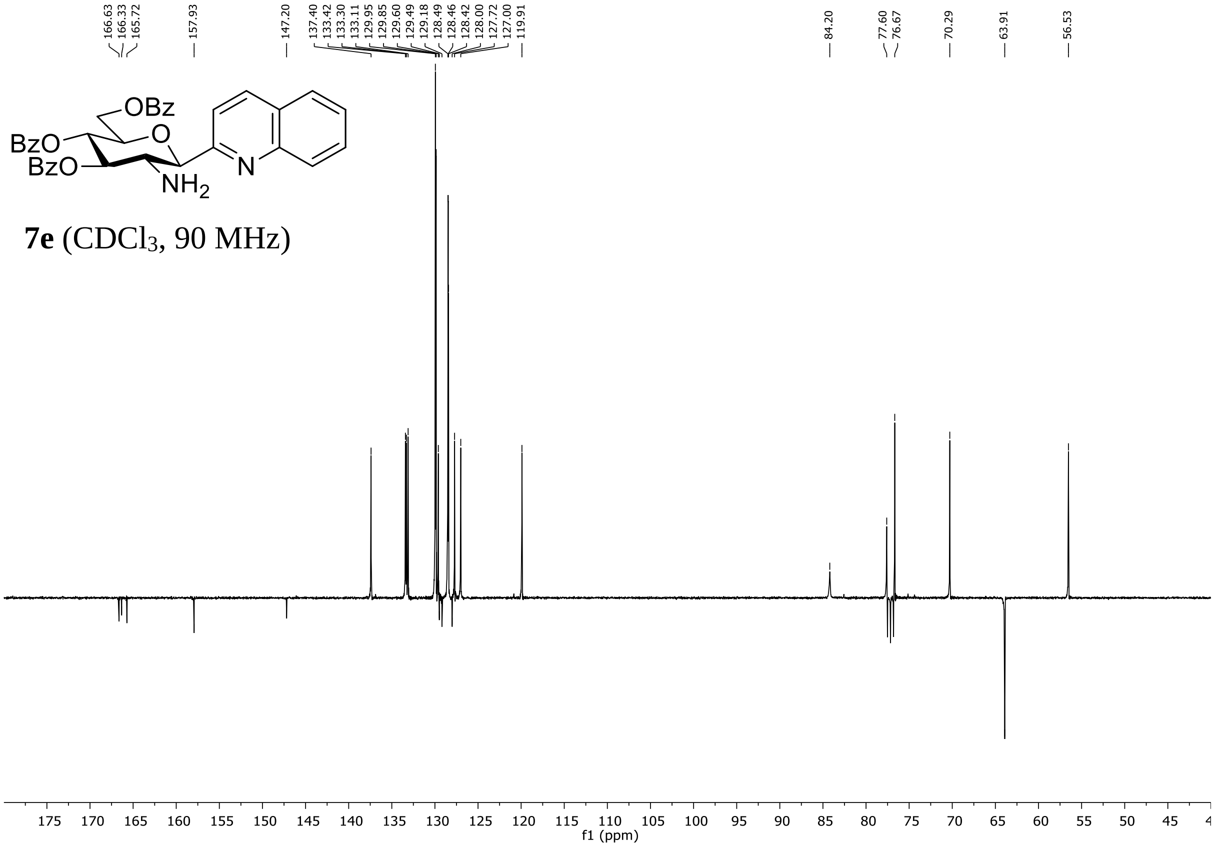

7e ($CDCl_3$, 90 MHz)

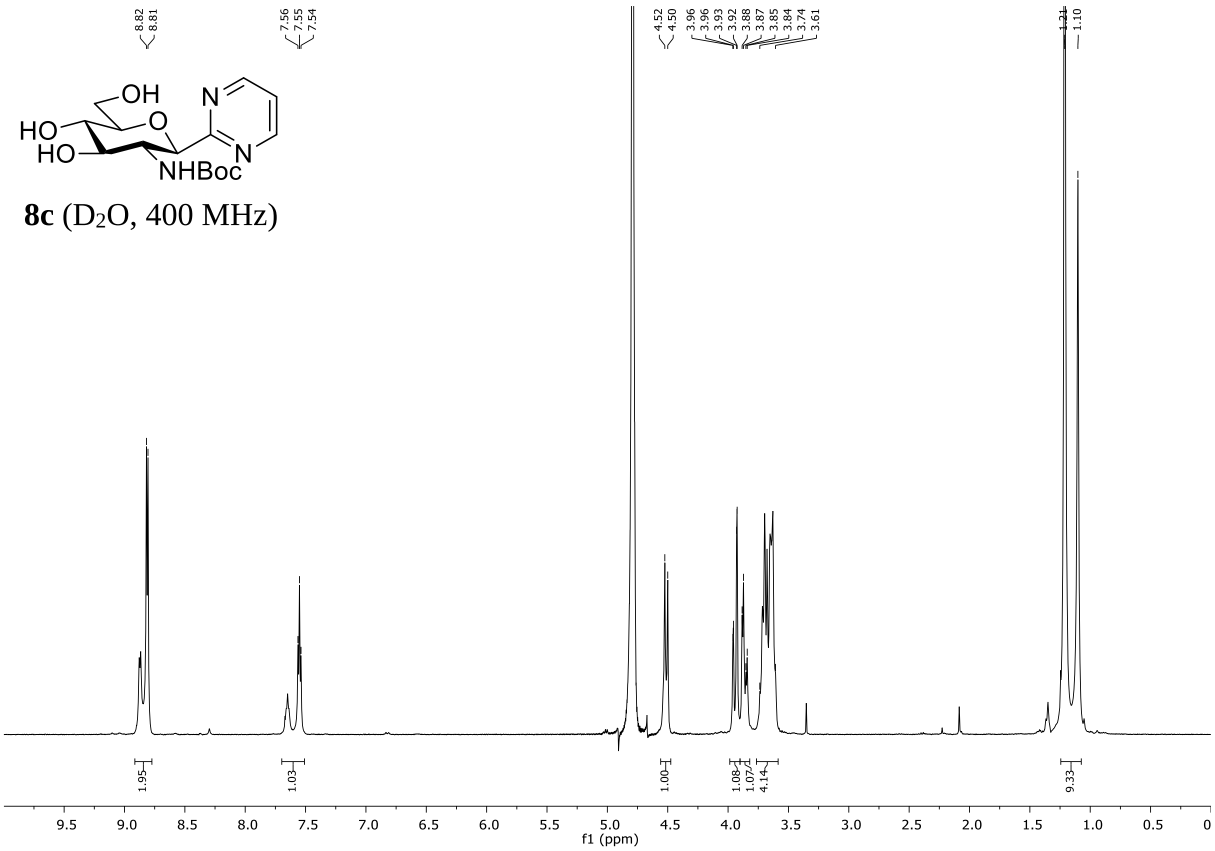

8c (D_2O, 400 MHz)

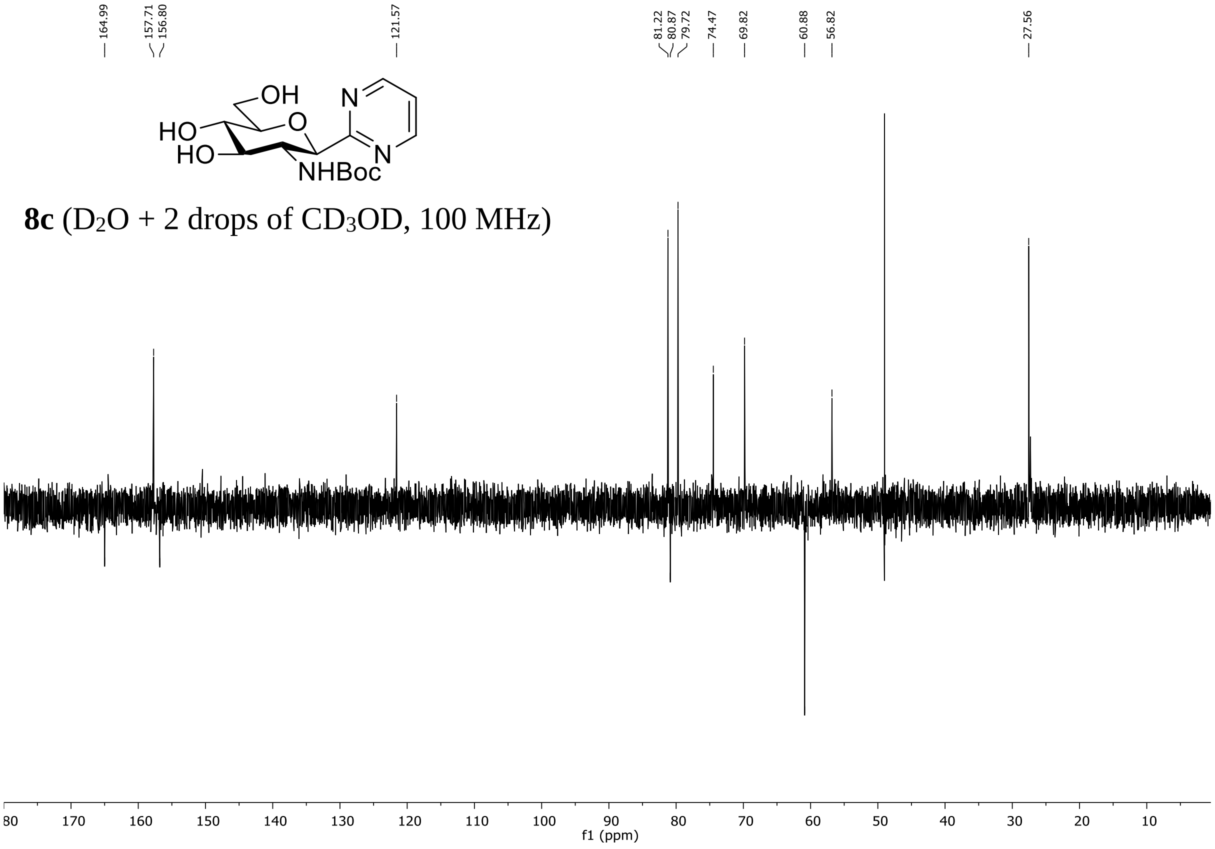

8c (D_2O + 2 drops of CD_3OD, 100 MHz)

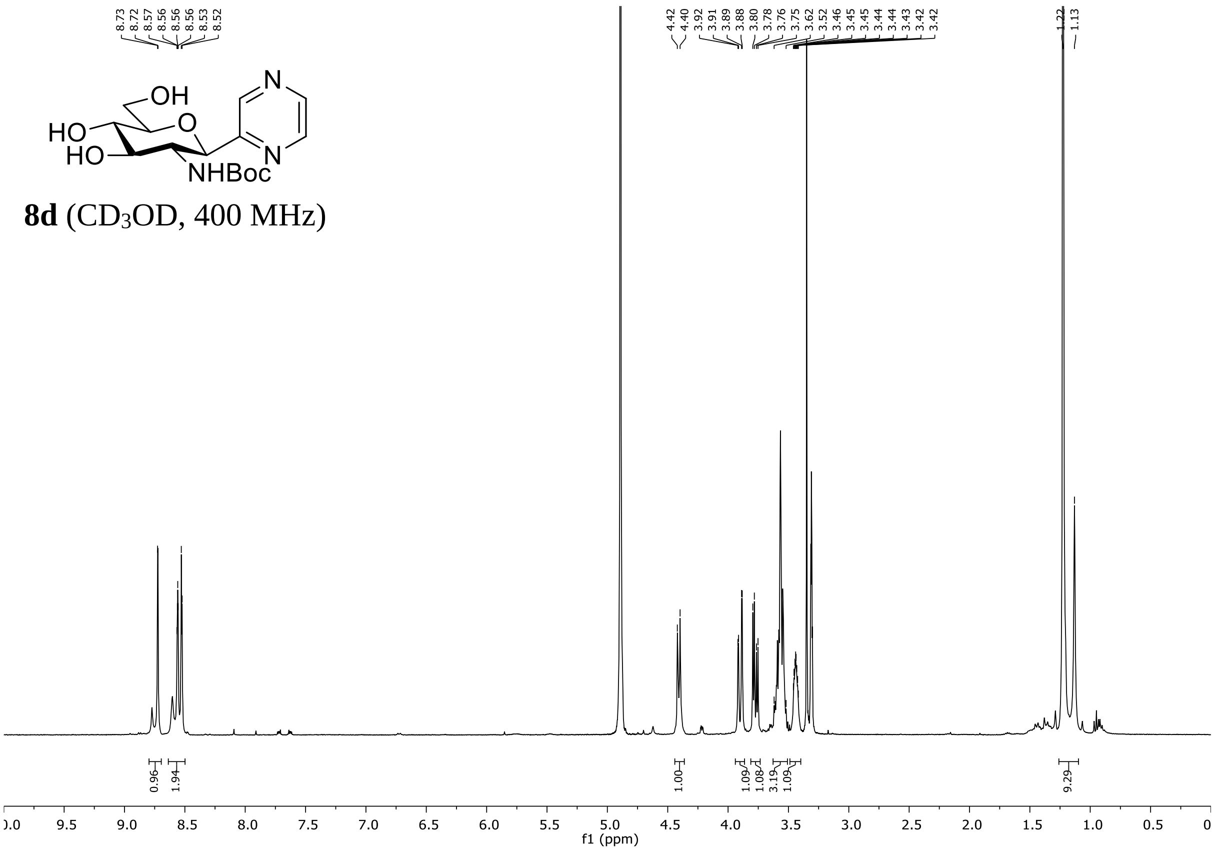

8d (CD_3OD, 400 MHz)

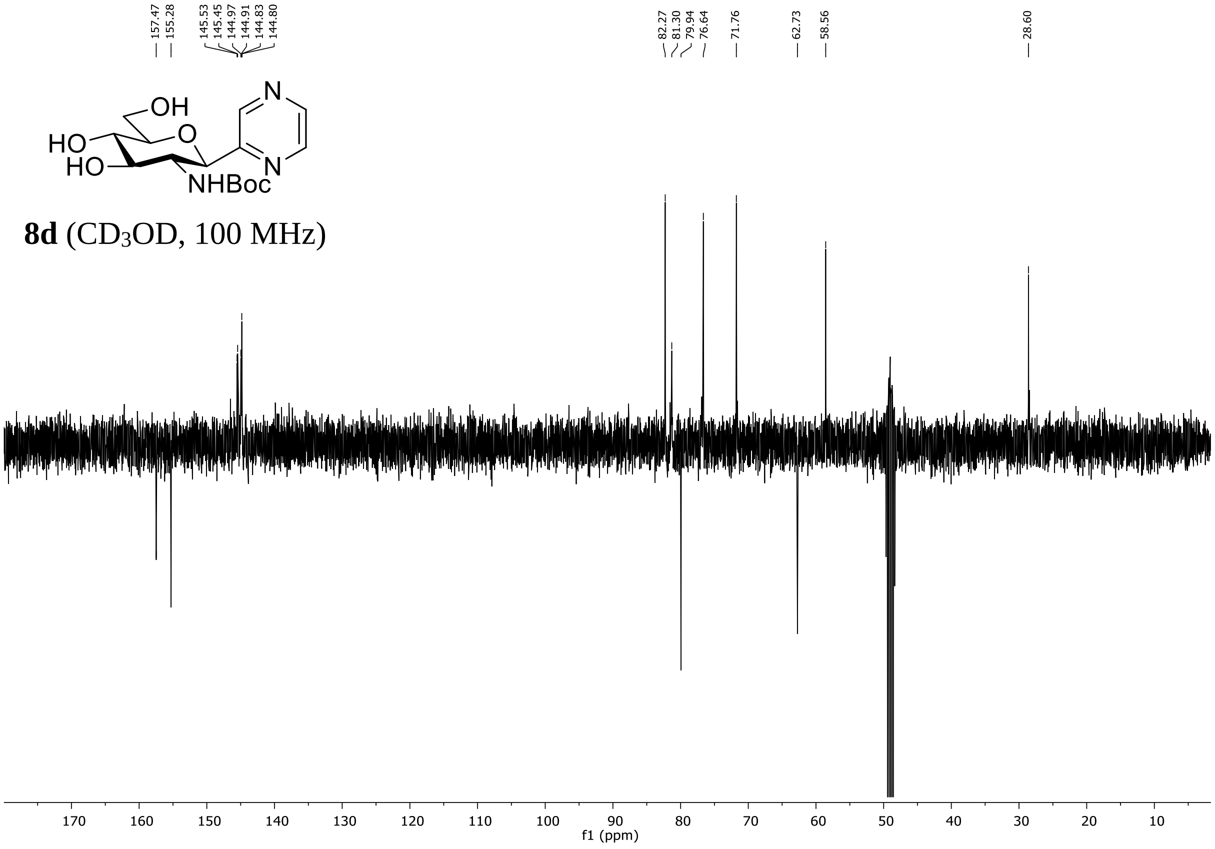

8d (CD_3OD, 100 MHz)

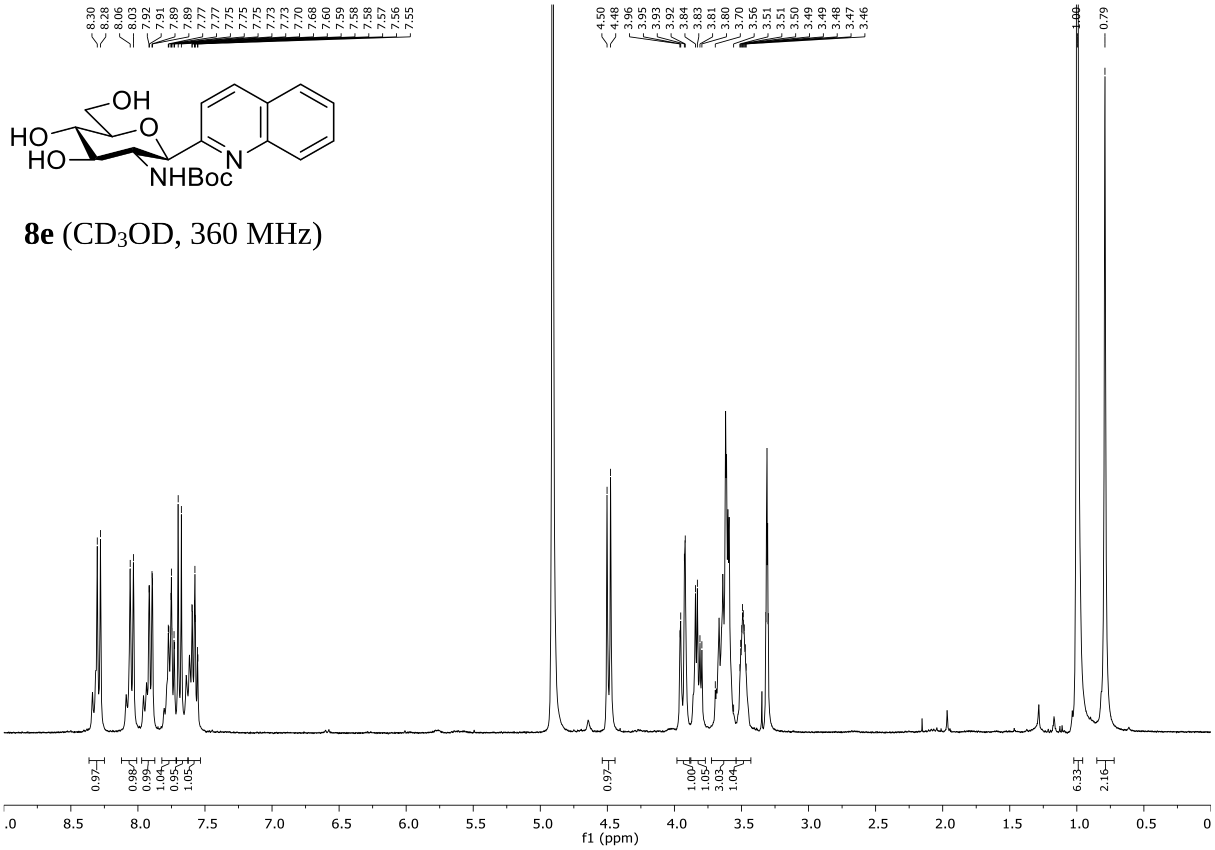

8e (CD_3OD, 360 MHz)

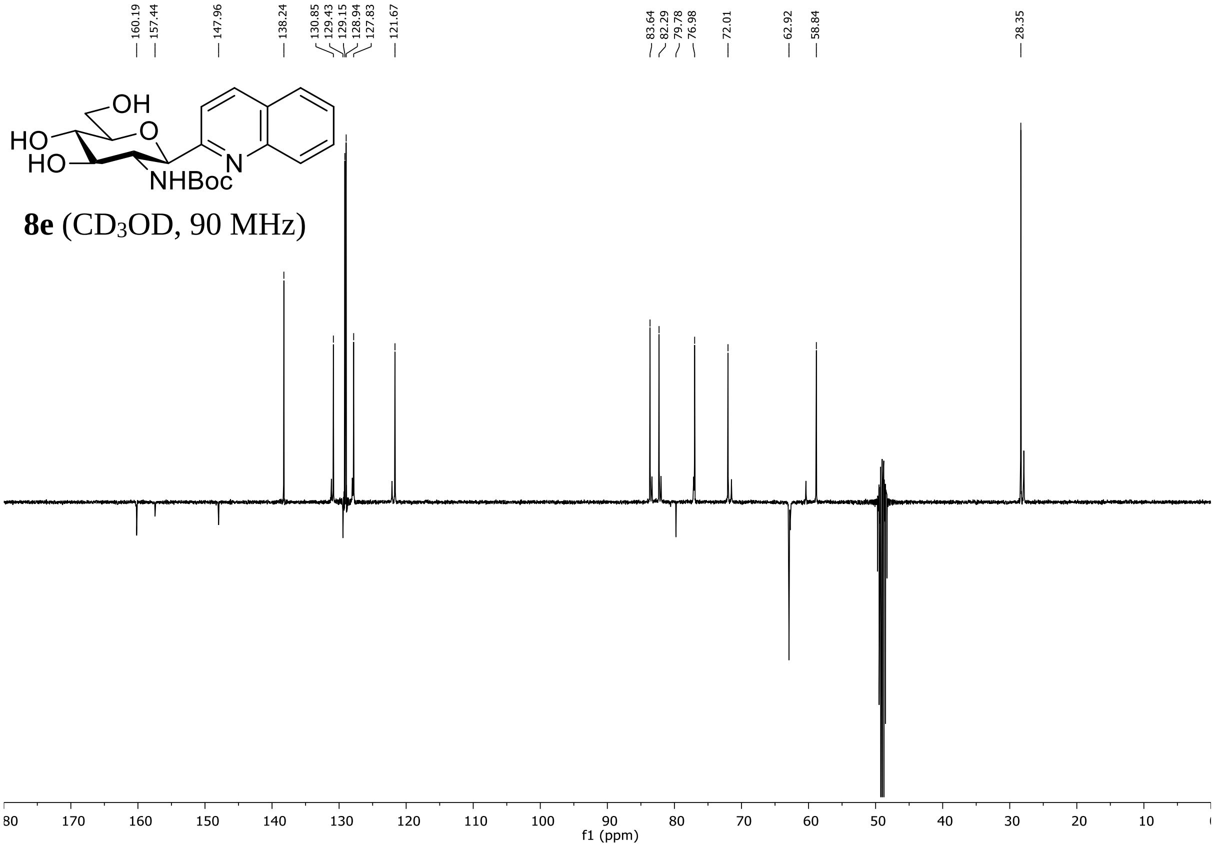

8e (CD_3OD, 90 MHz)

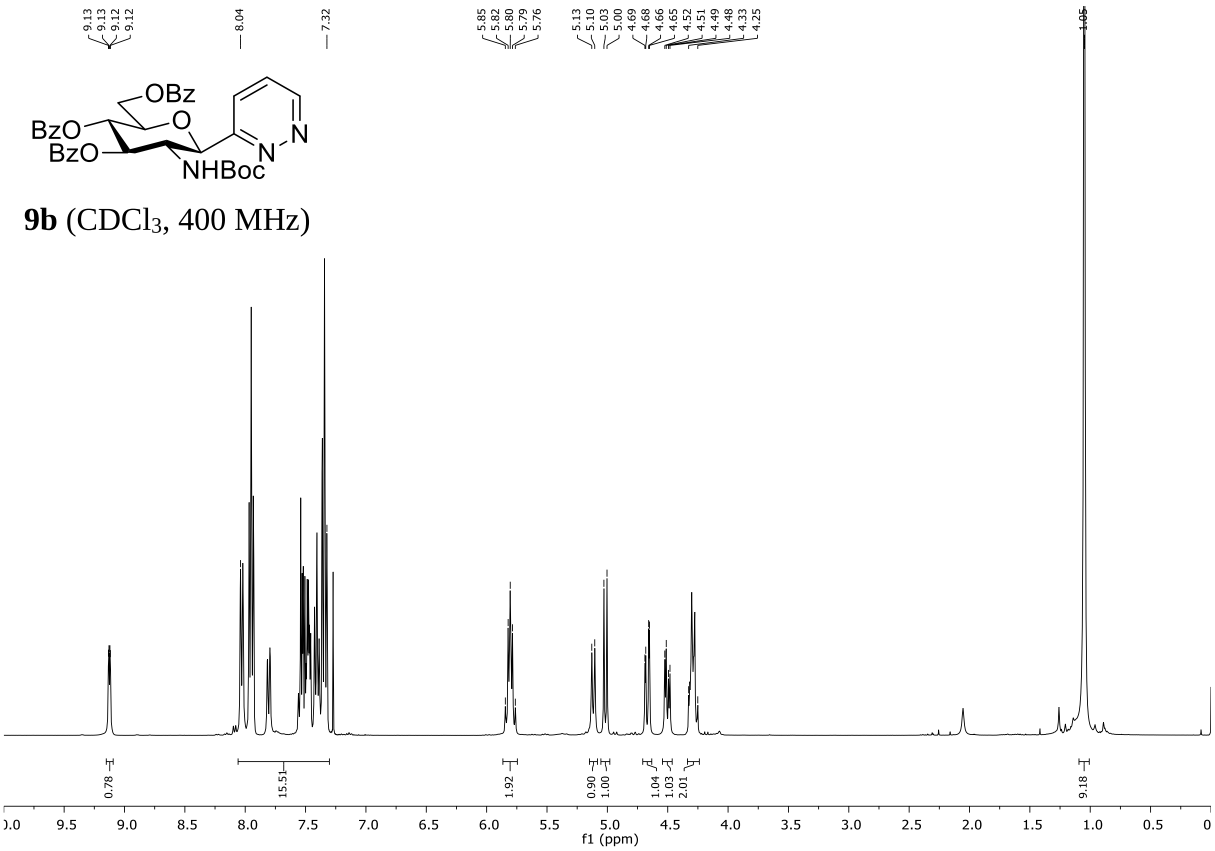

9b ($CDCl_3$, 400 MHz)

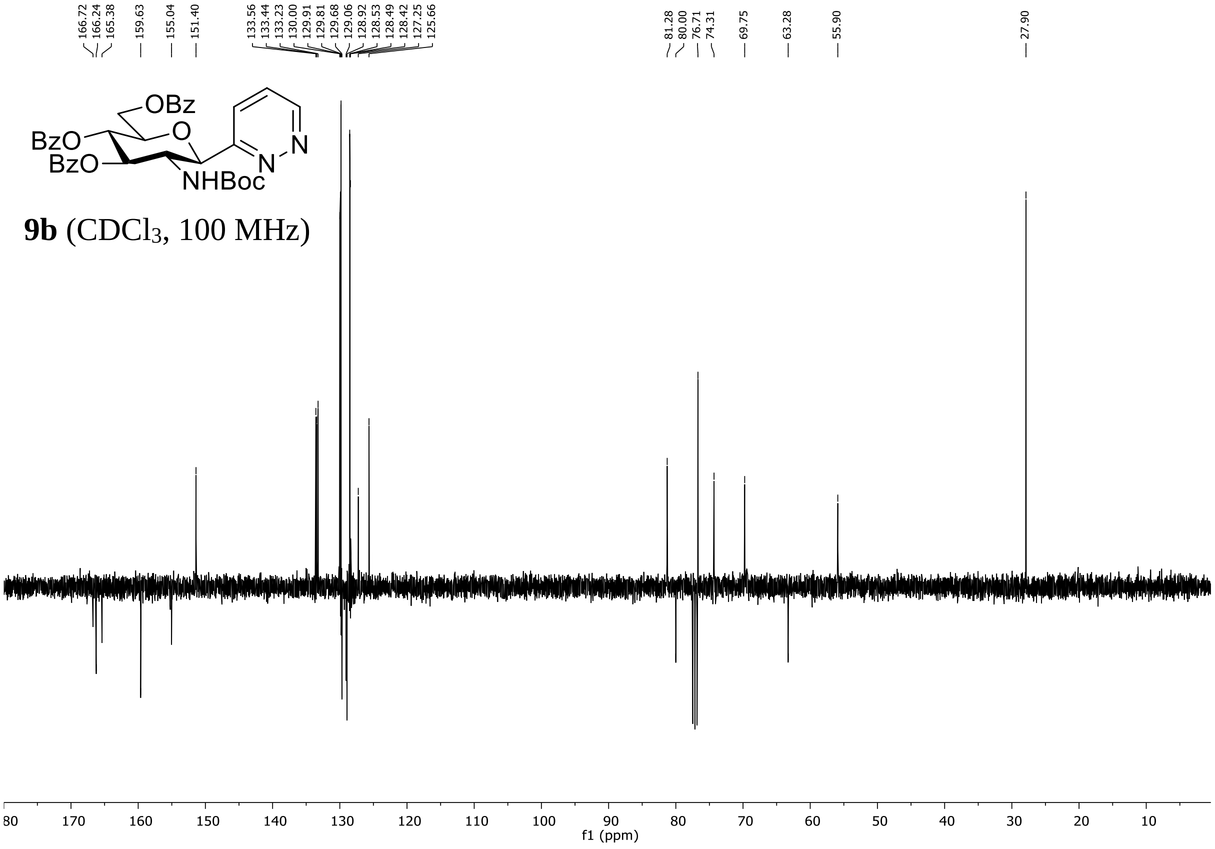

9b ($CDCl_3$, 100 MHz)

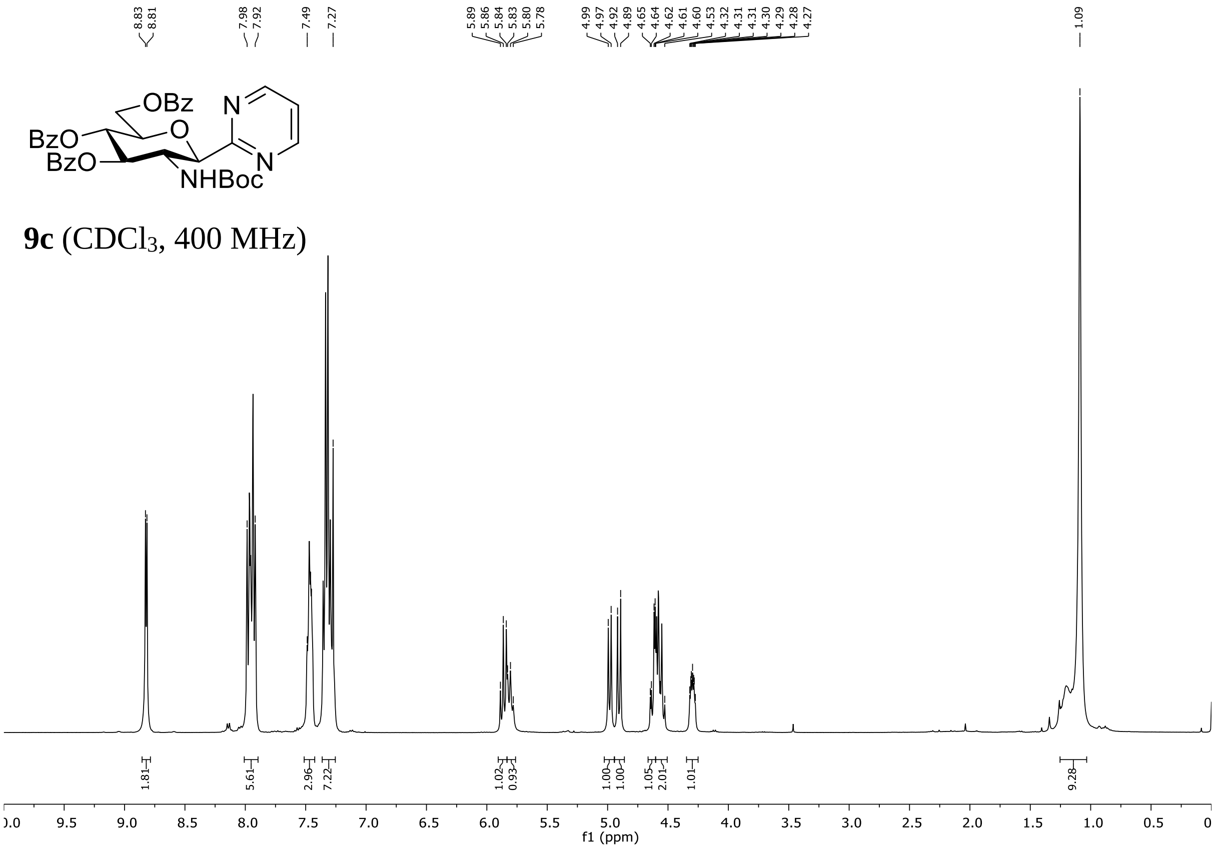

9c ($CDCl_3$, 400 MHz)

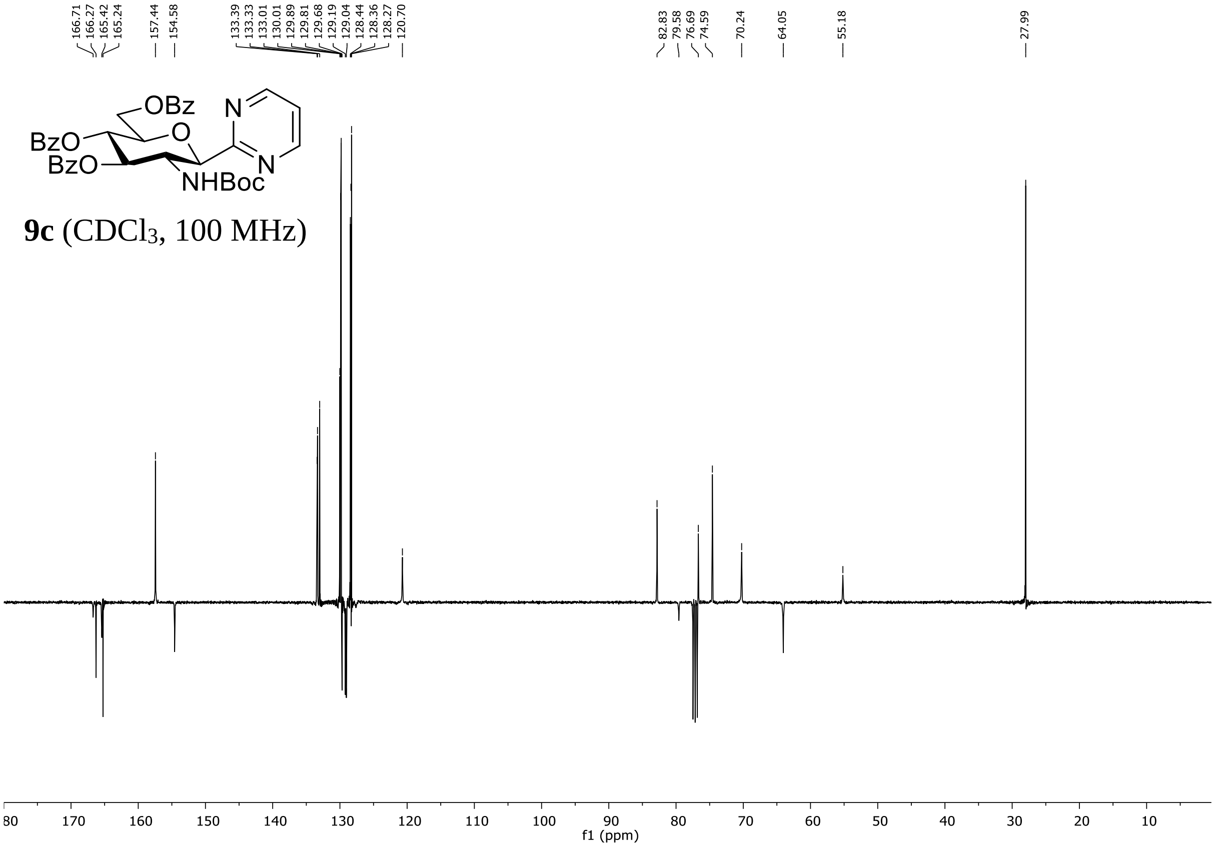

9c ($CDCl_3$, 100 MHz)

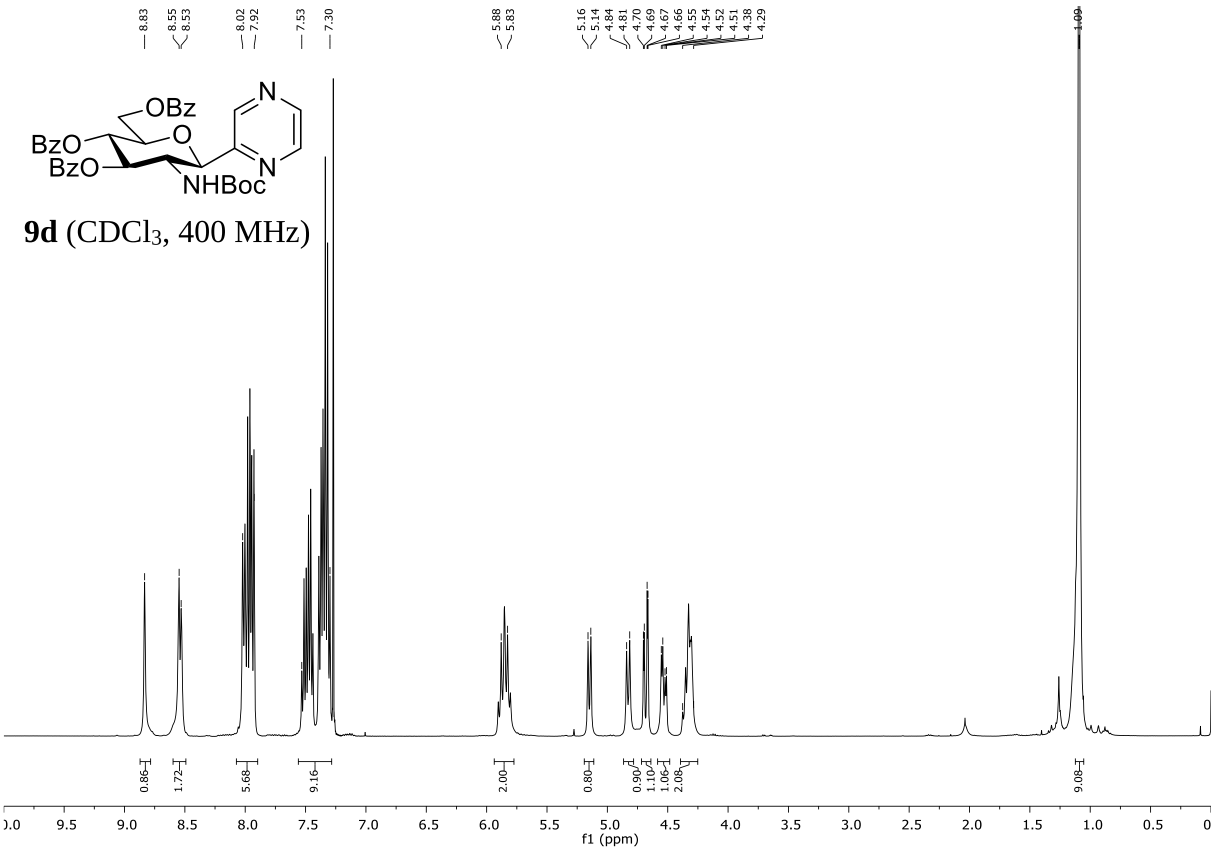
8.83
8.55
8.53
8.02
7.92
7.53
7.30
5.88
5.83
5.16
5.14
4.84
4.81
4.70
4.69
4.67
4.66
4.55
4.54
4.52
4.51
4.38
4.29
1.09
OBz
BzO
BzO
O
N
N
NHBoc
9d ($CDCl_3$, 400 MHz)
f1 (ppm)

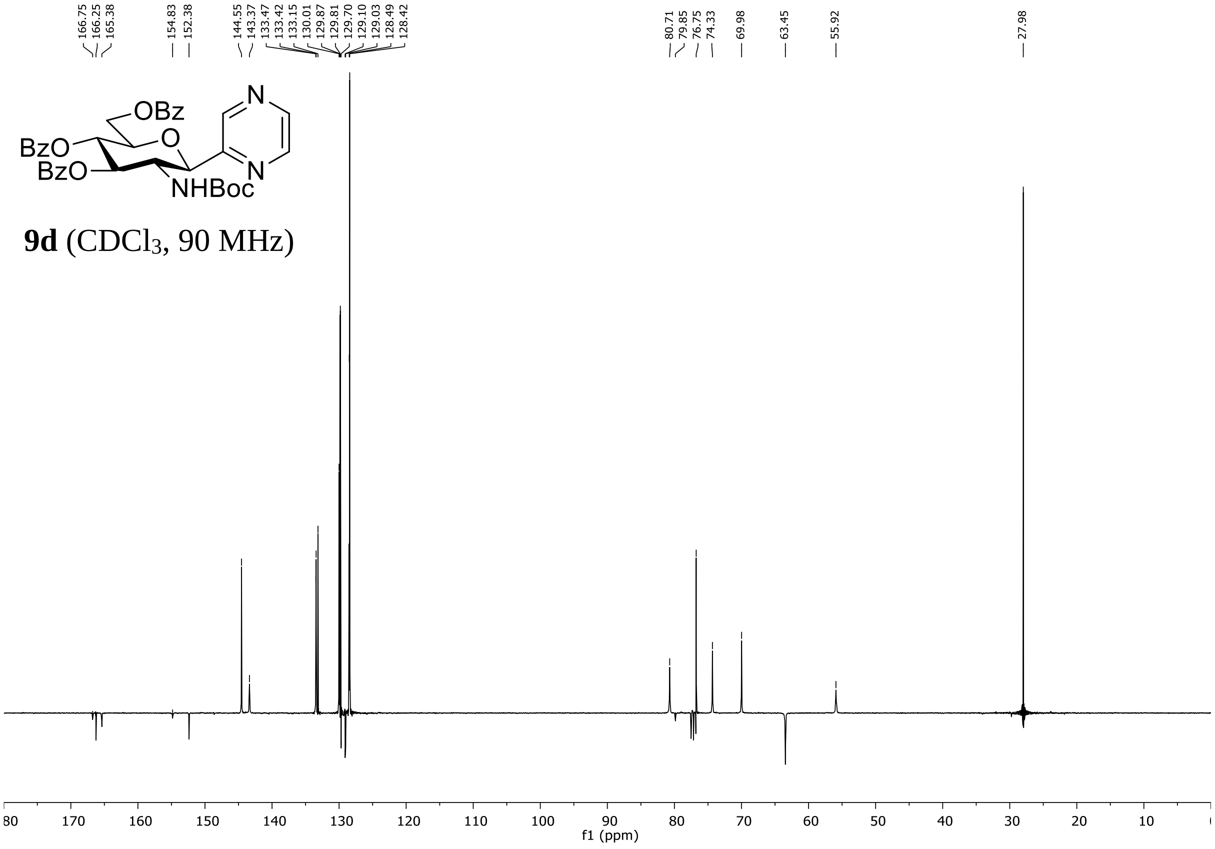
166.75
166.25
165.38
154.83
152.38
144.55
143.37
133.47
133.42
133.15
130.01
129.87
129.81
129.70
129.10
129.03
128.49
128.42
80.71
79.85
76.75
74.33
69.98
63.45
55.92
27.98
OBz
BzO
BzO
O
N
N
NHBoc
9d ($CDCl_3$, 90 MHz)
f1 (ppm)

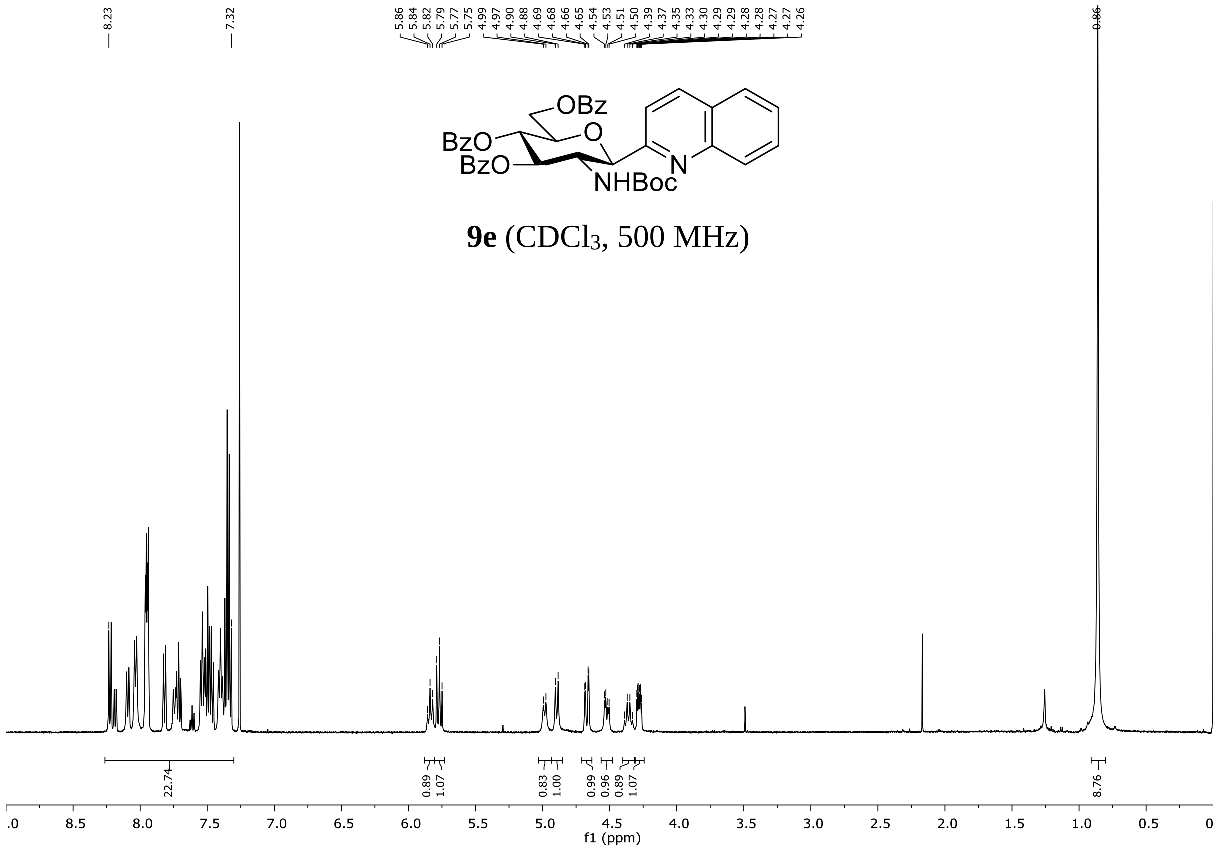

9e ($CDCl_3$, 500 MHz)

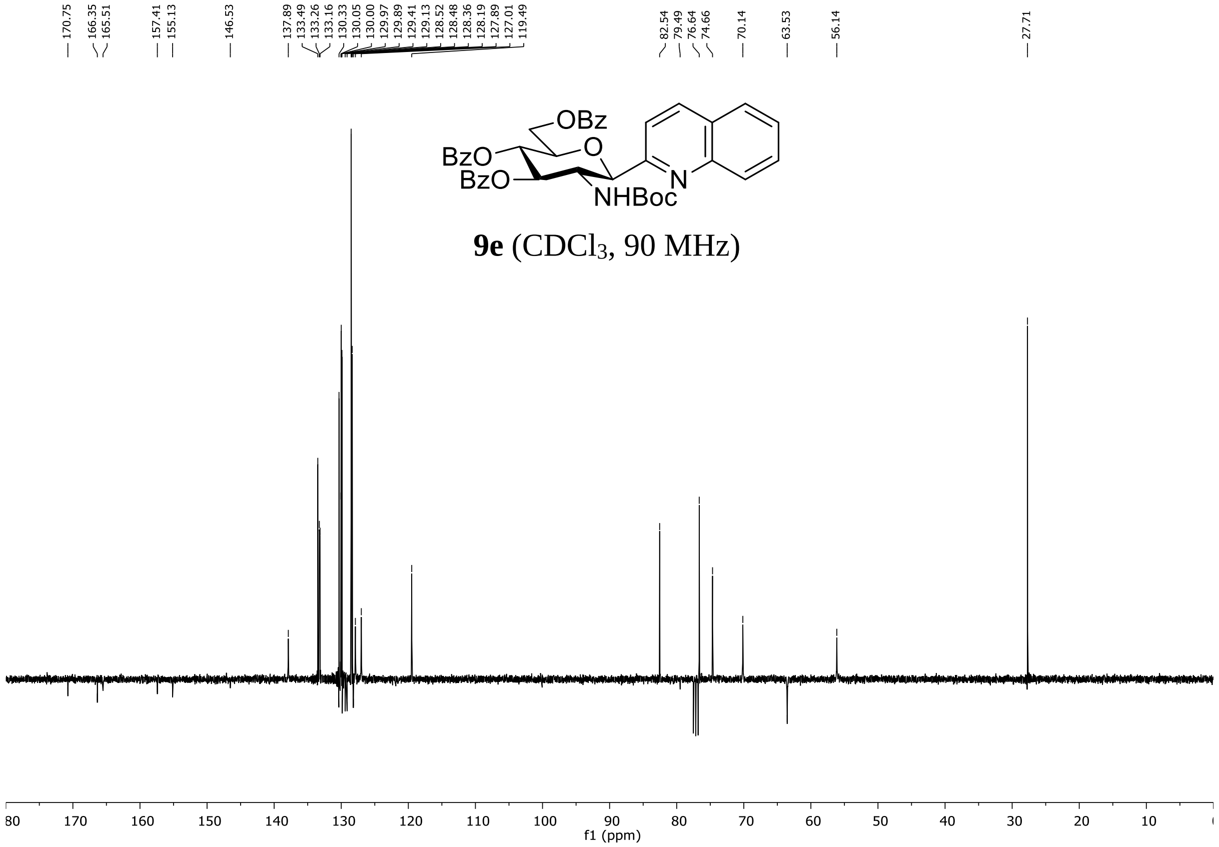

9e ($CDCl_3$, 90 MHz)

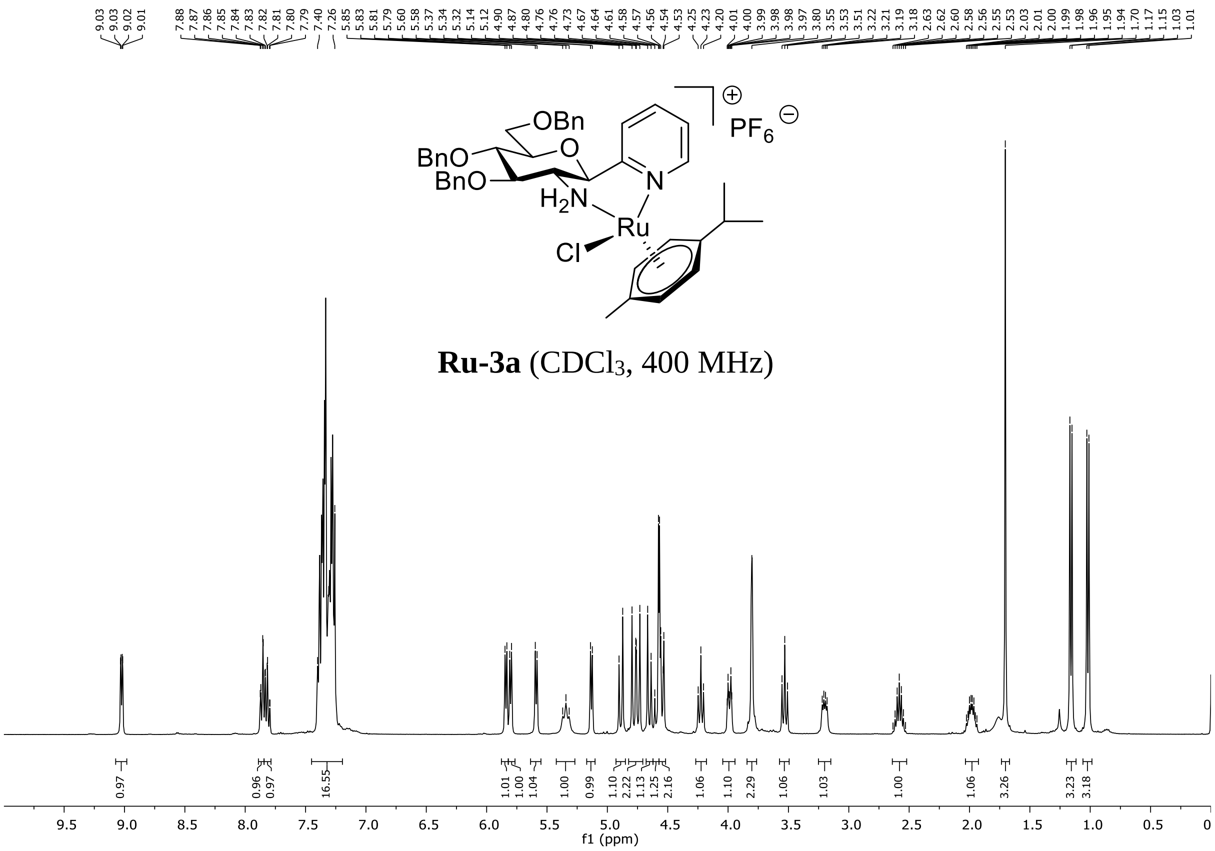
OBn
BnO
BnO
O
N
H2N
Ru
Cl
PF6
Ru-3a (CDCl3, 400 MHz)
f1 (ppm)

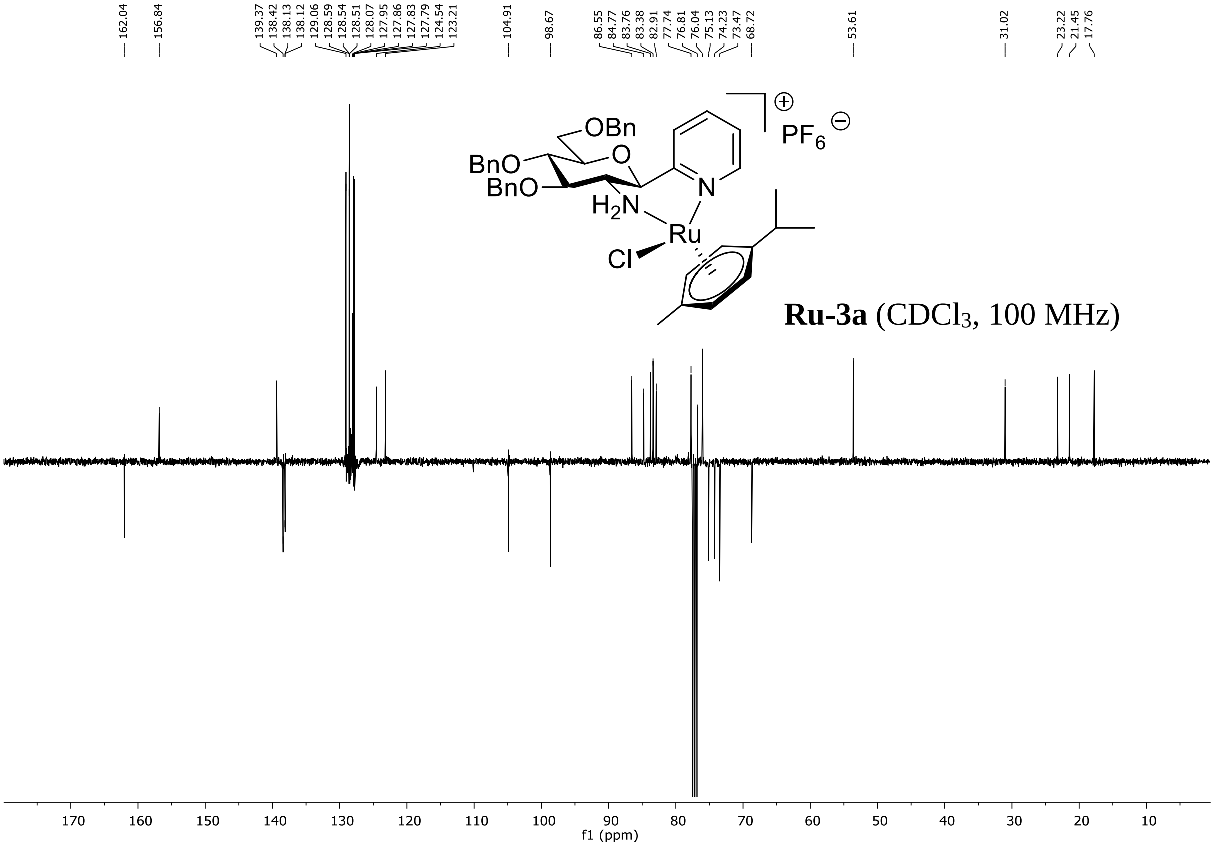
OBn
BnO
BnO
O
N
H2N
Ru
Cl
PF6
Ru-3a (CDCl3, 100 MHz)
f1 (ppm)

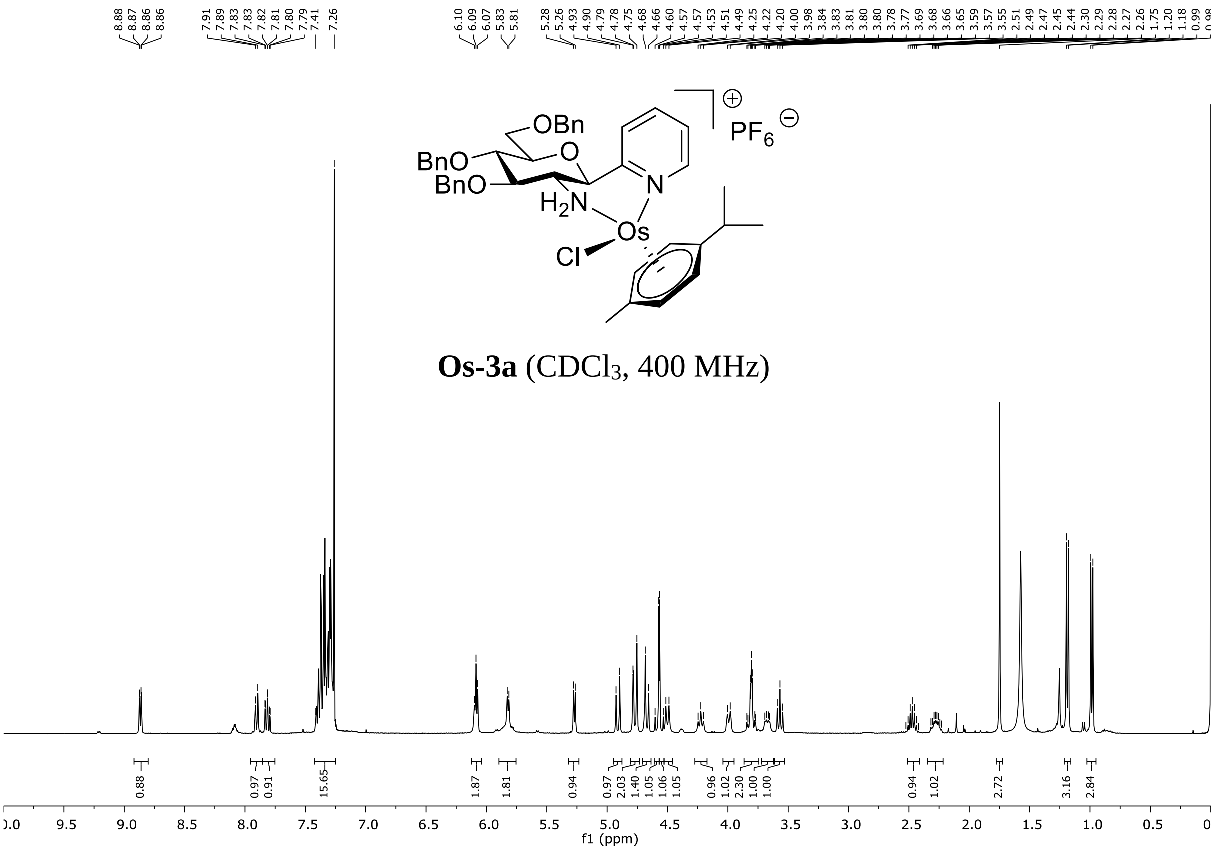

Os-3a ($CDCl_3$, 400 MHz)

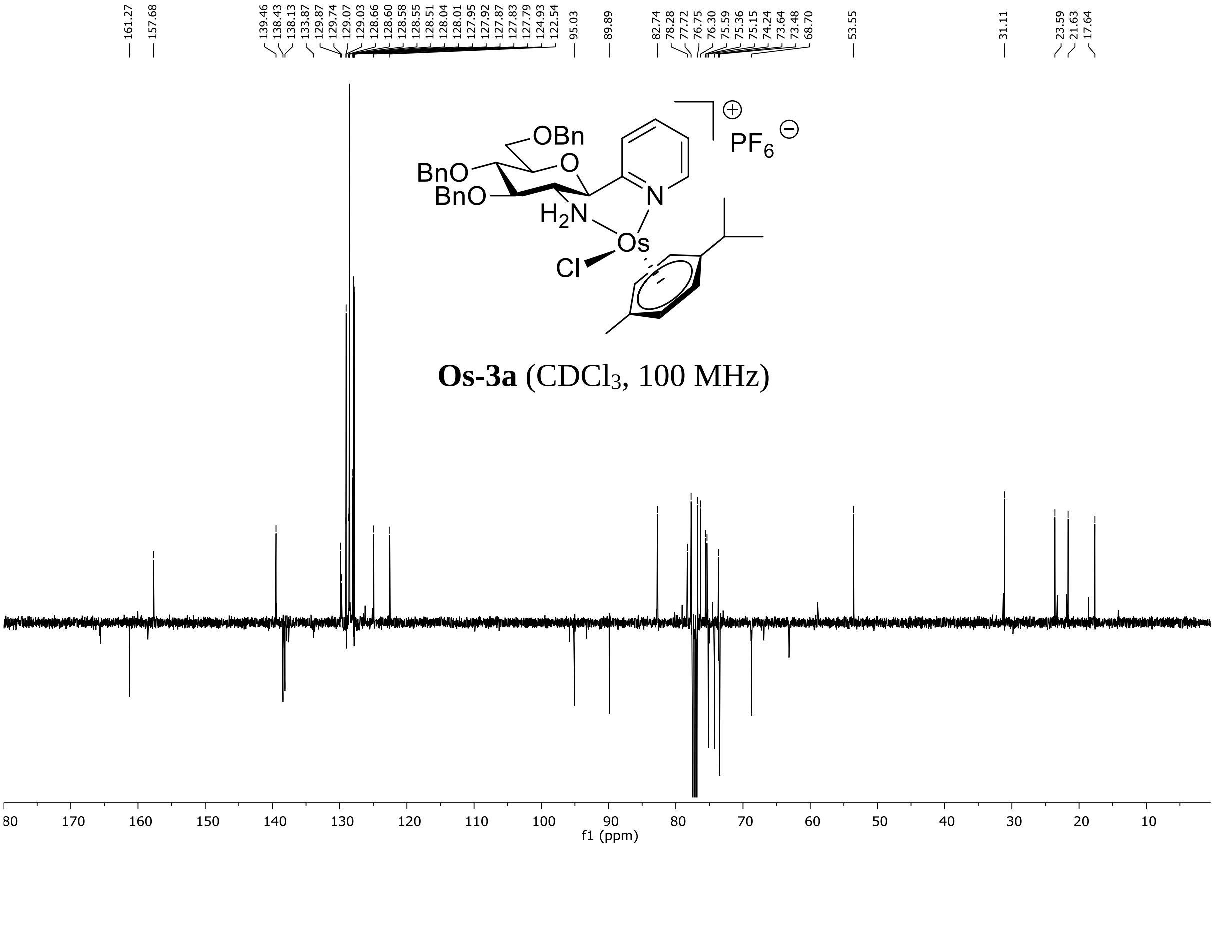

Os-3a ($CDCl_3$, 100 MHz)

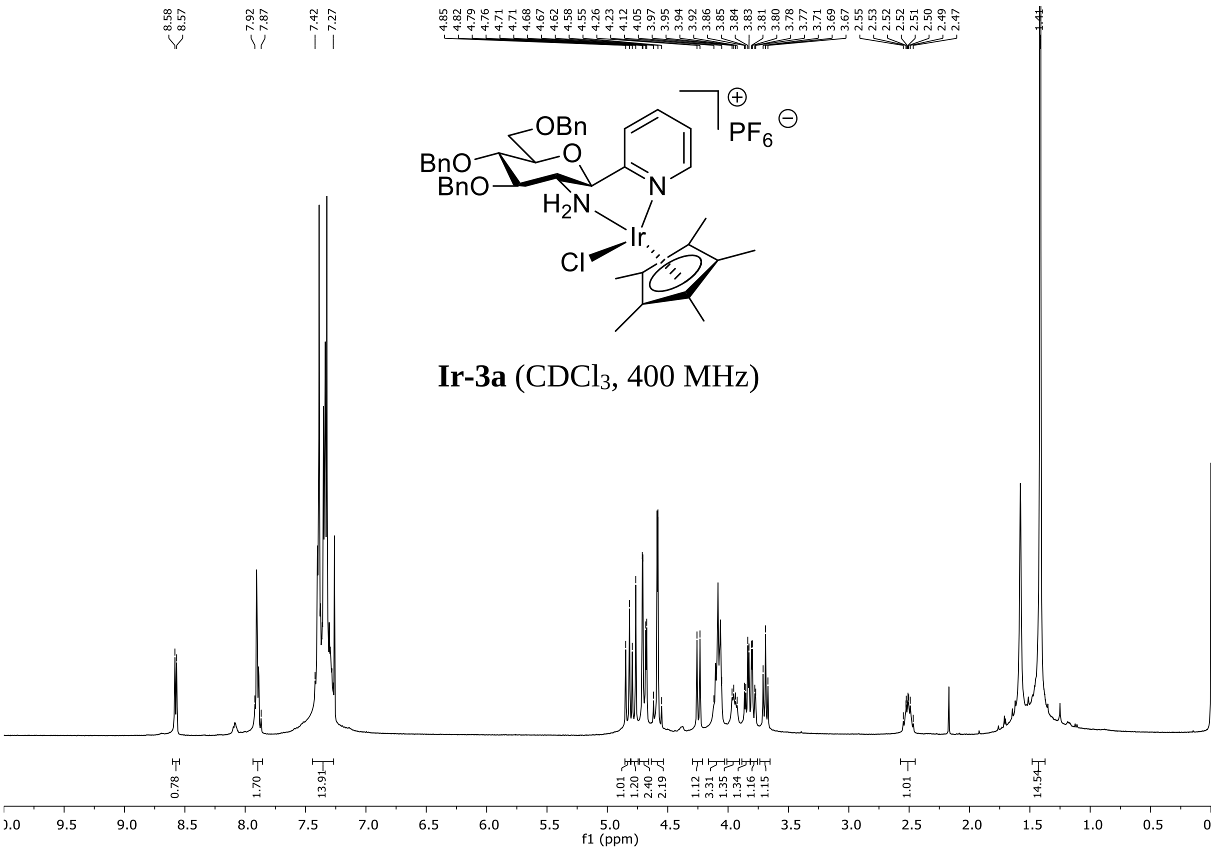

Ir-3a (CDCl3, 400 MHz)
f1 (ppm)

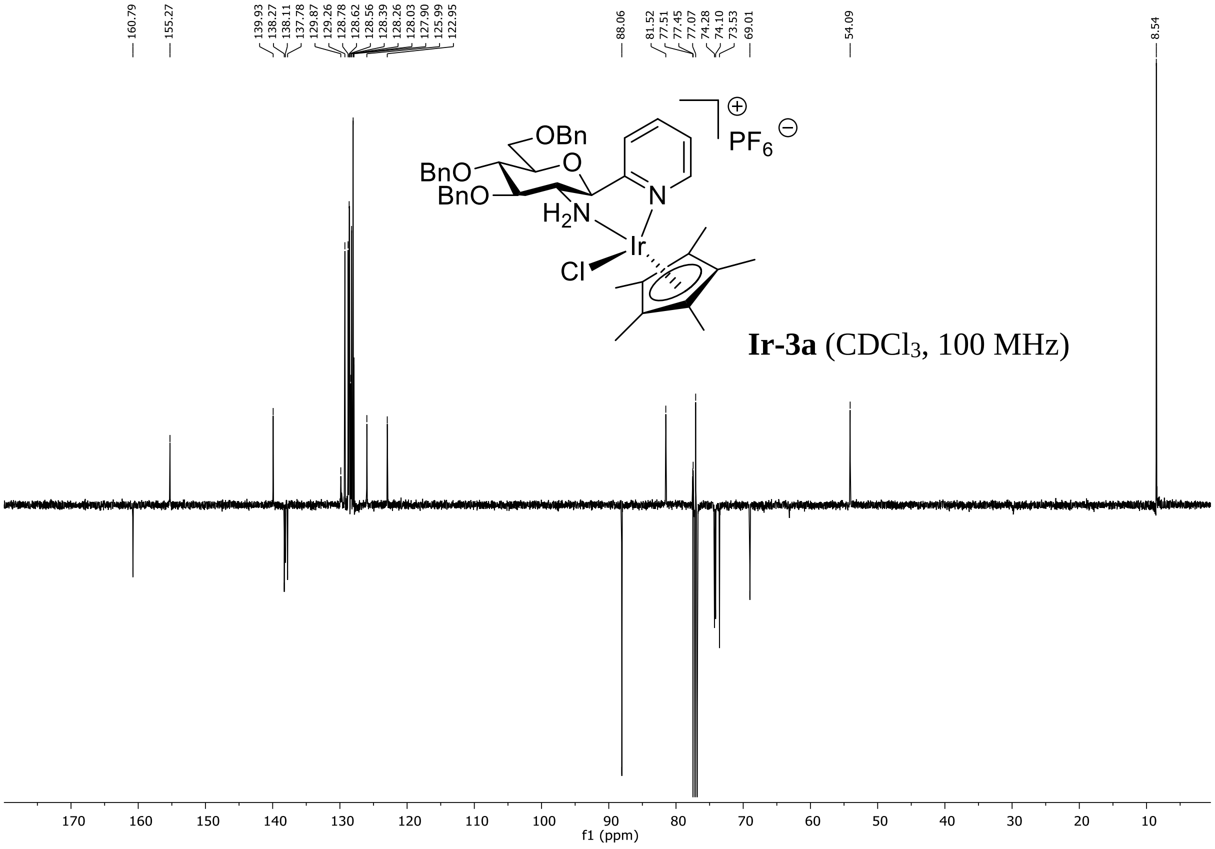

Ir-3a (CDCl3, 100 MHz)
f1 (ppm)

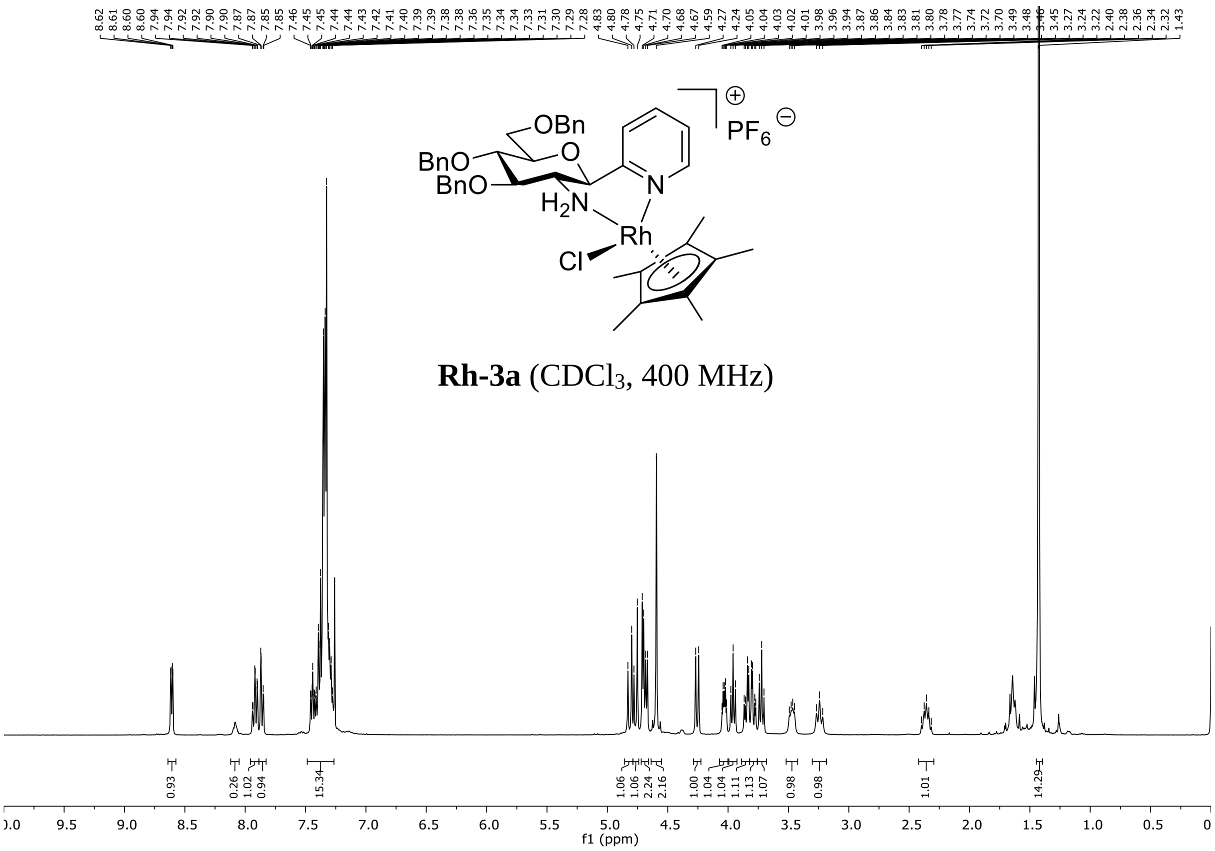

Rh-3a ($CDCl_3$, 400 MHz)

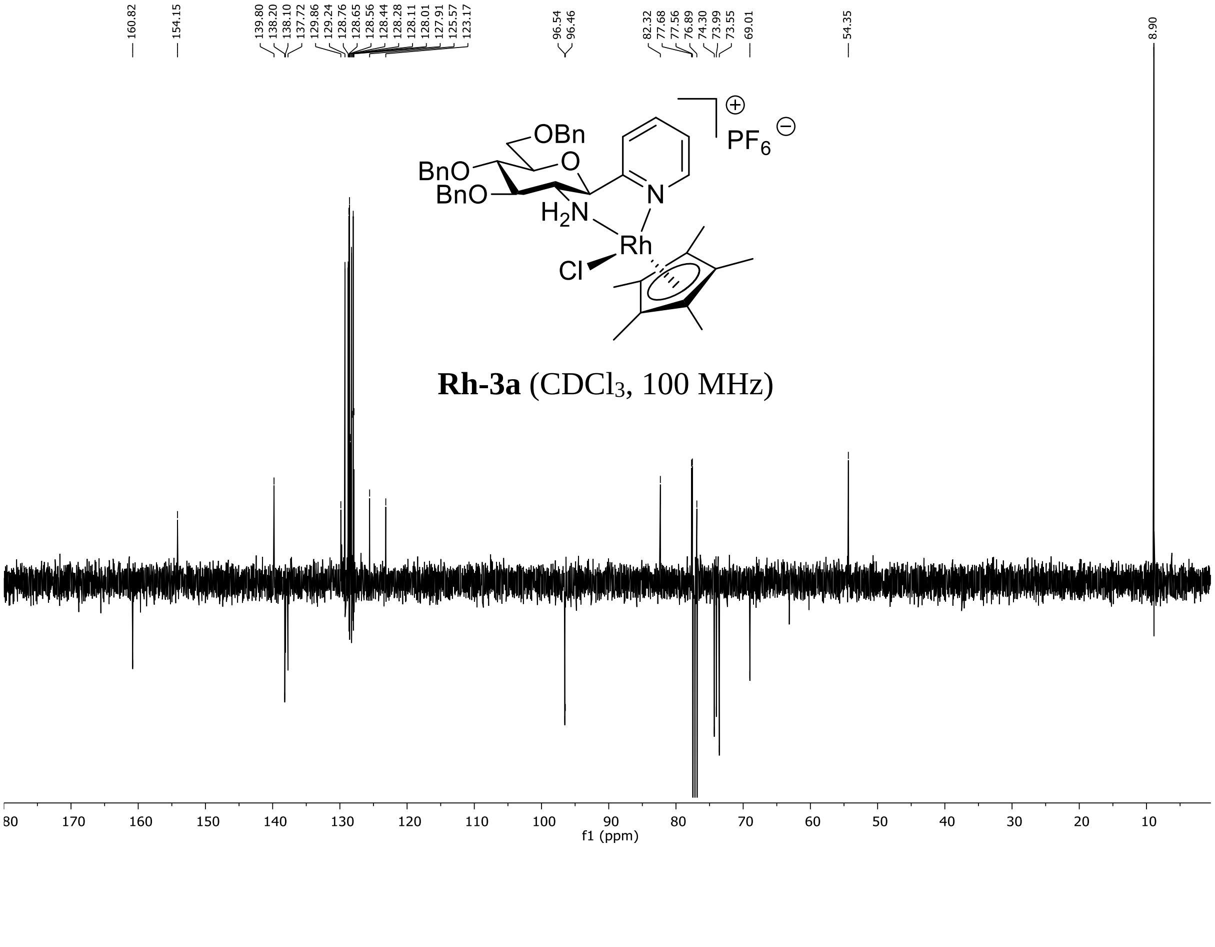

Rh-3a ($CDCl_3$, 100 MHz)

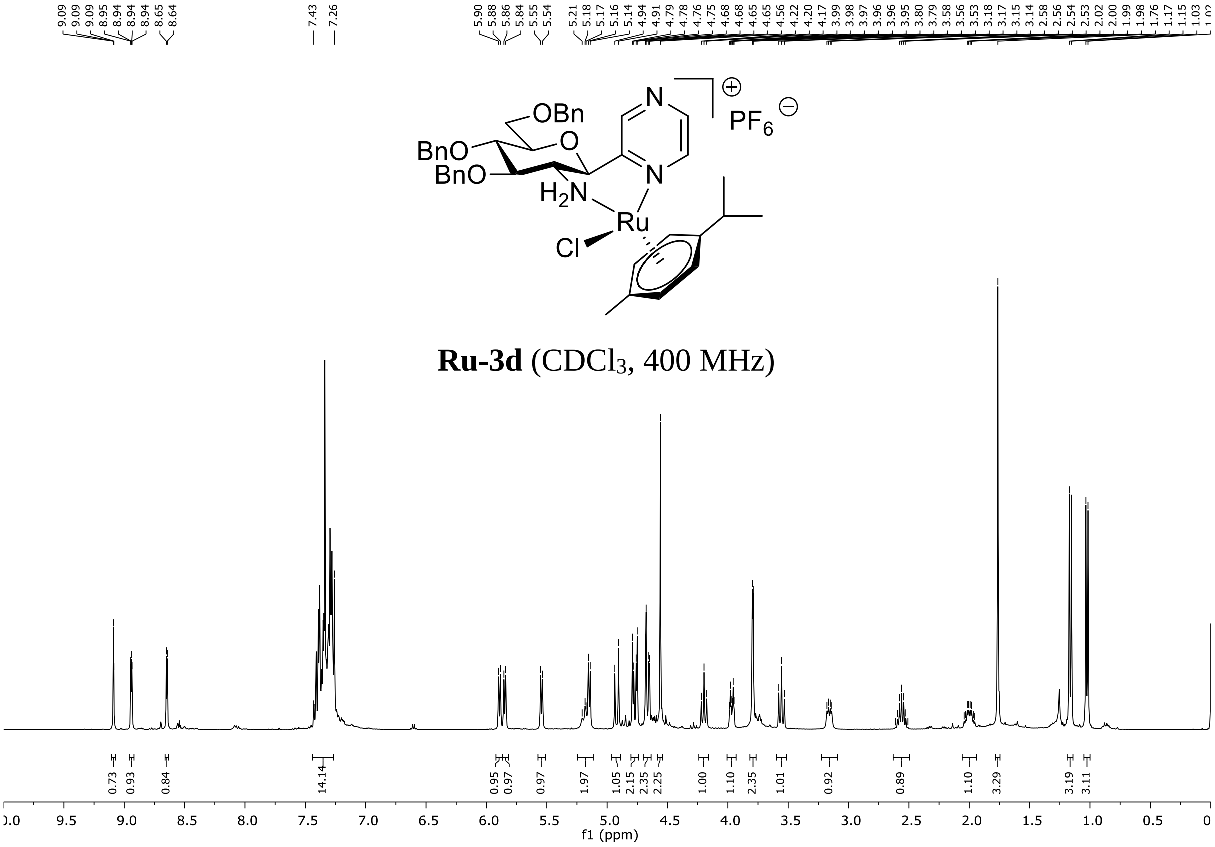

Ru-3d ($CDCl_3$, 400 MHz)

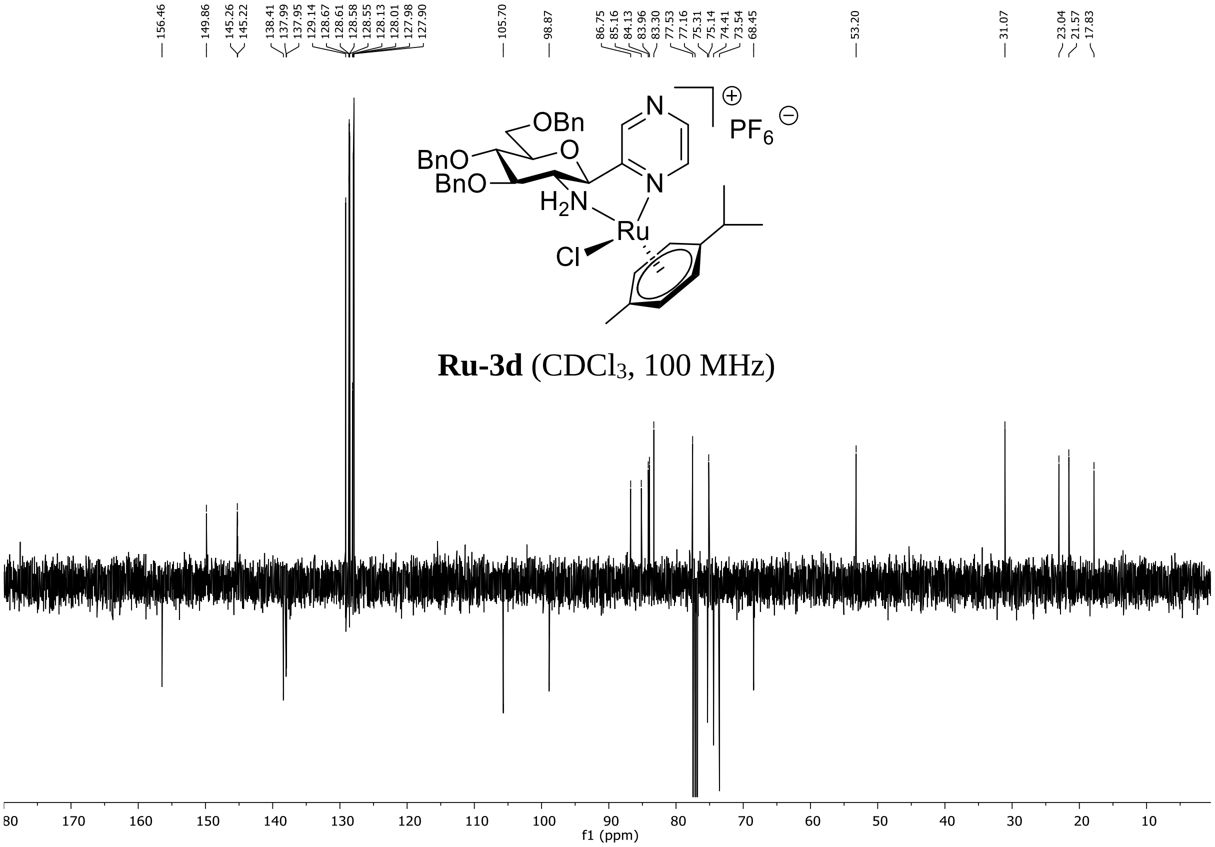

Ru-3d ($CDCl_3$, 100 MHz)

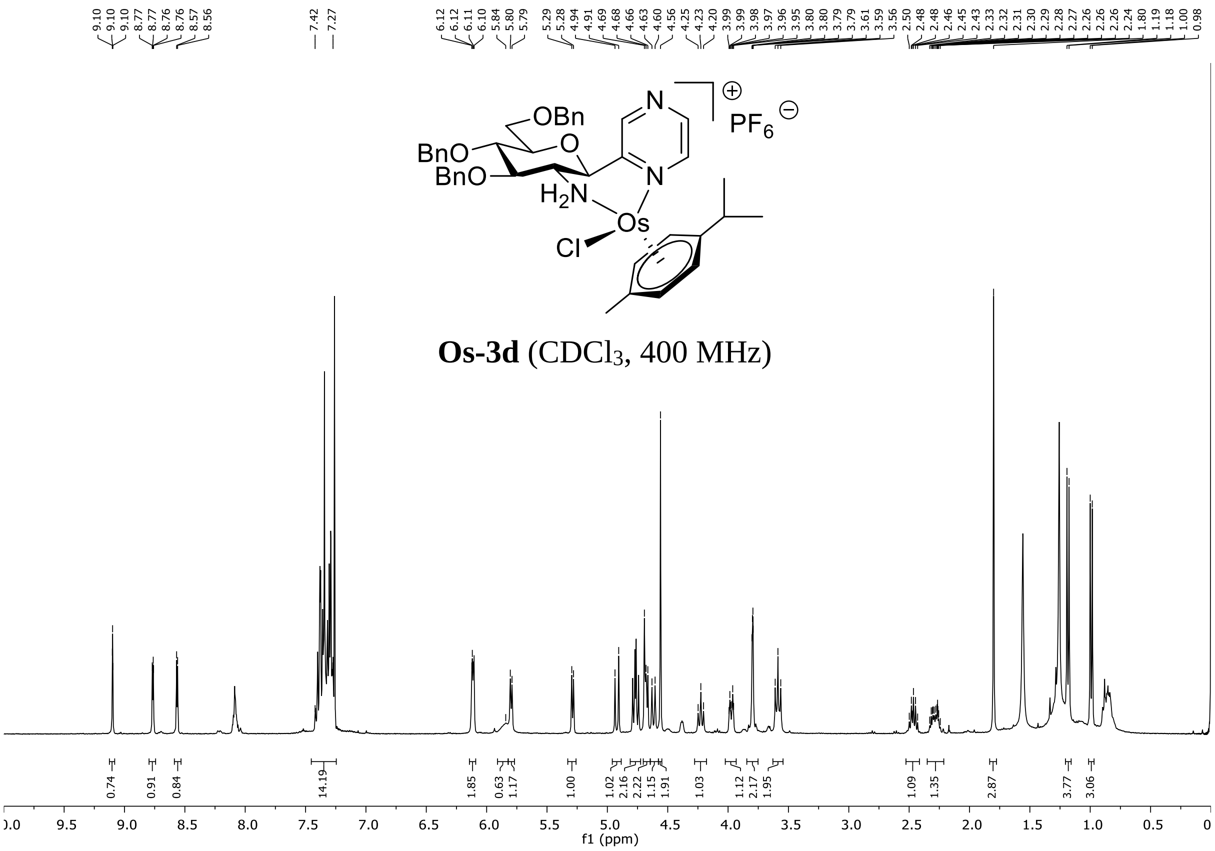

Os-3d ($CDCl_3$, 400 MHz)

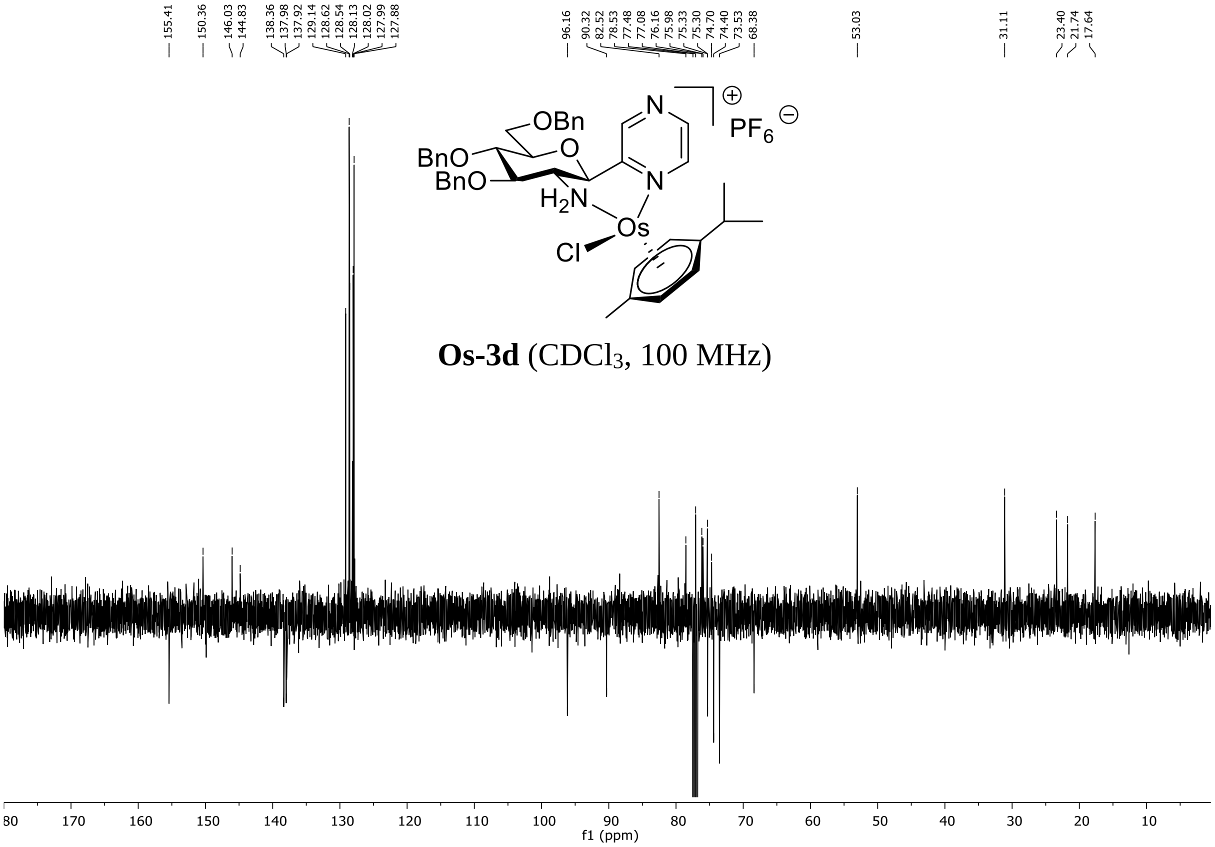

Os-3d ($CDCl_3$, 100 MHz)

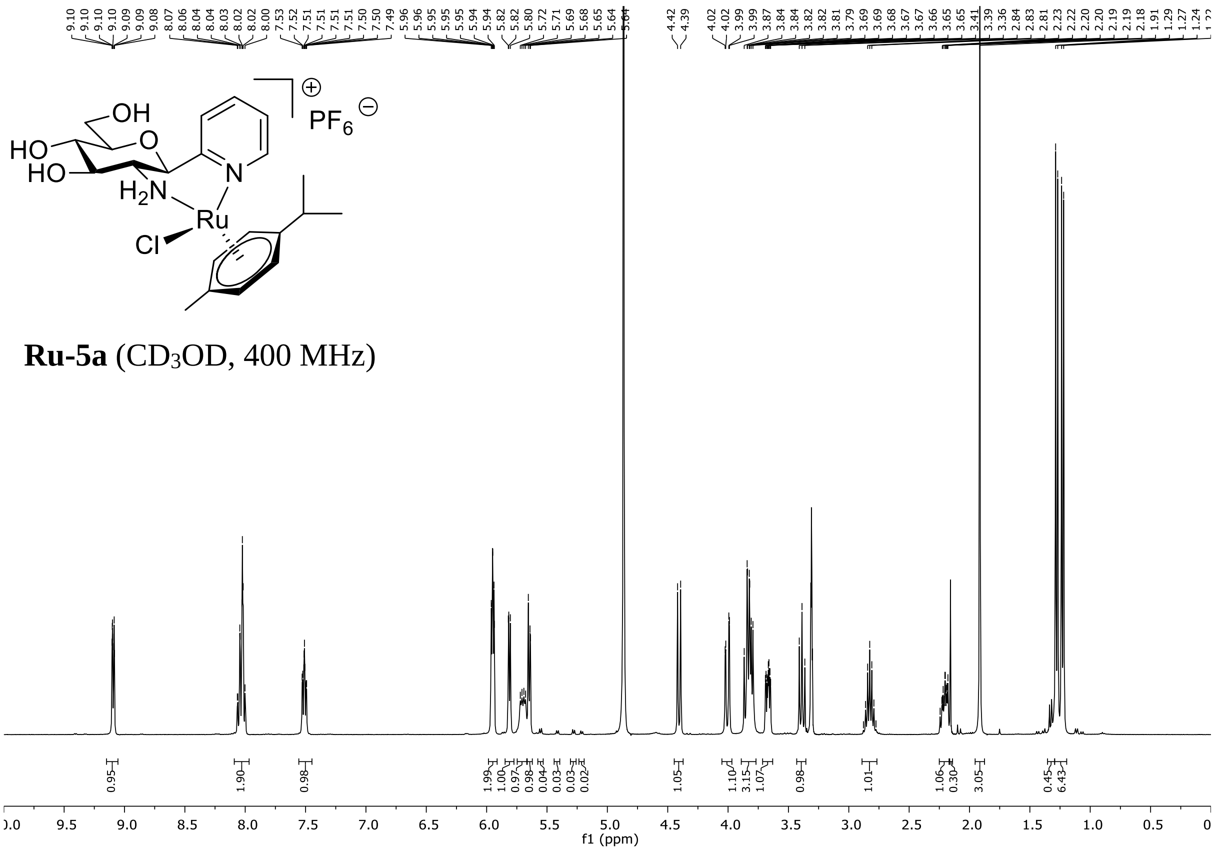
PF6
Ru-5a (CD3OD, 400 MHz)
f1 (ppm)

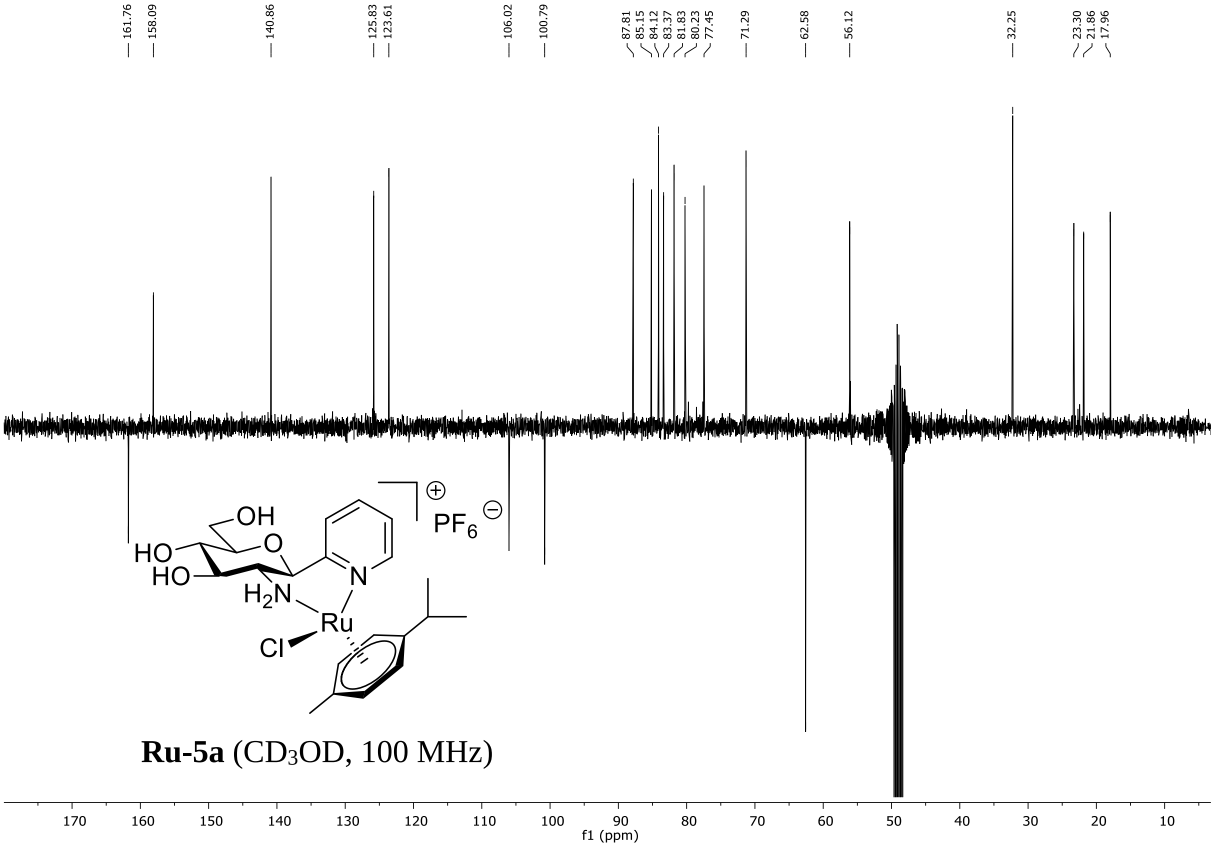
PF6
Ru-5a (CD3OD, 100 MHz)
f1 (ppm)

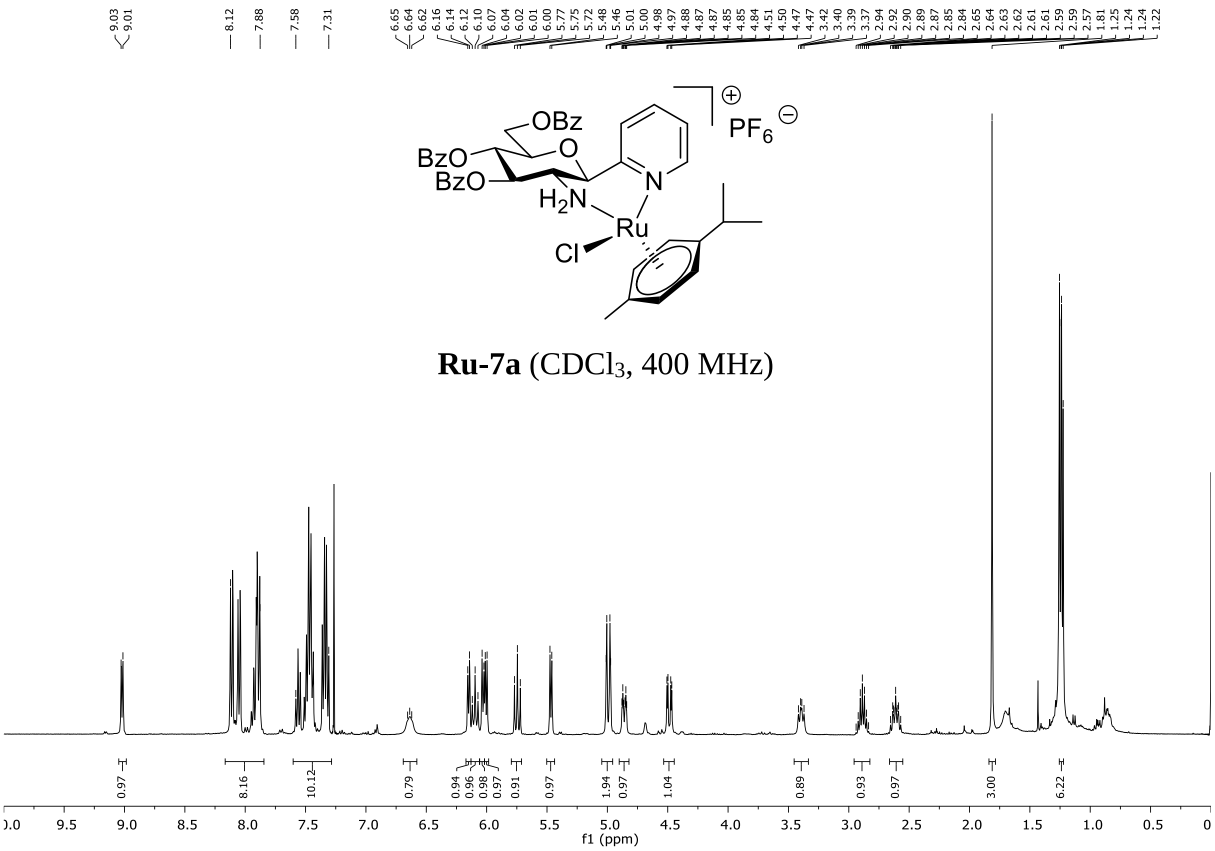

Ru-7a ($CDCl_3$, 400 MHz)

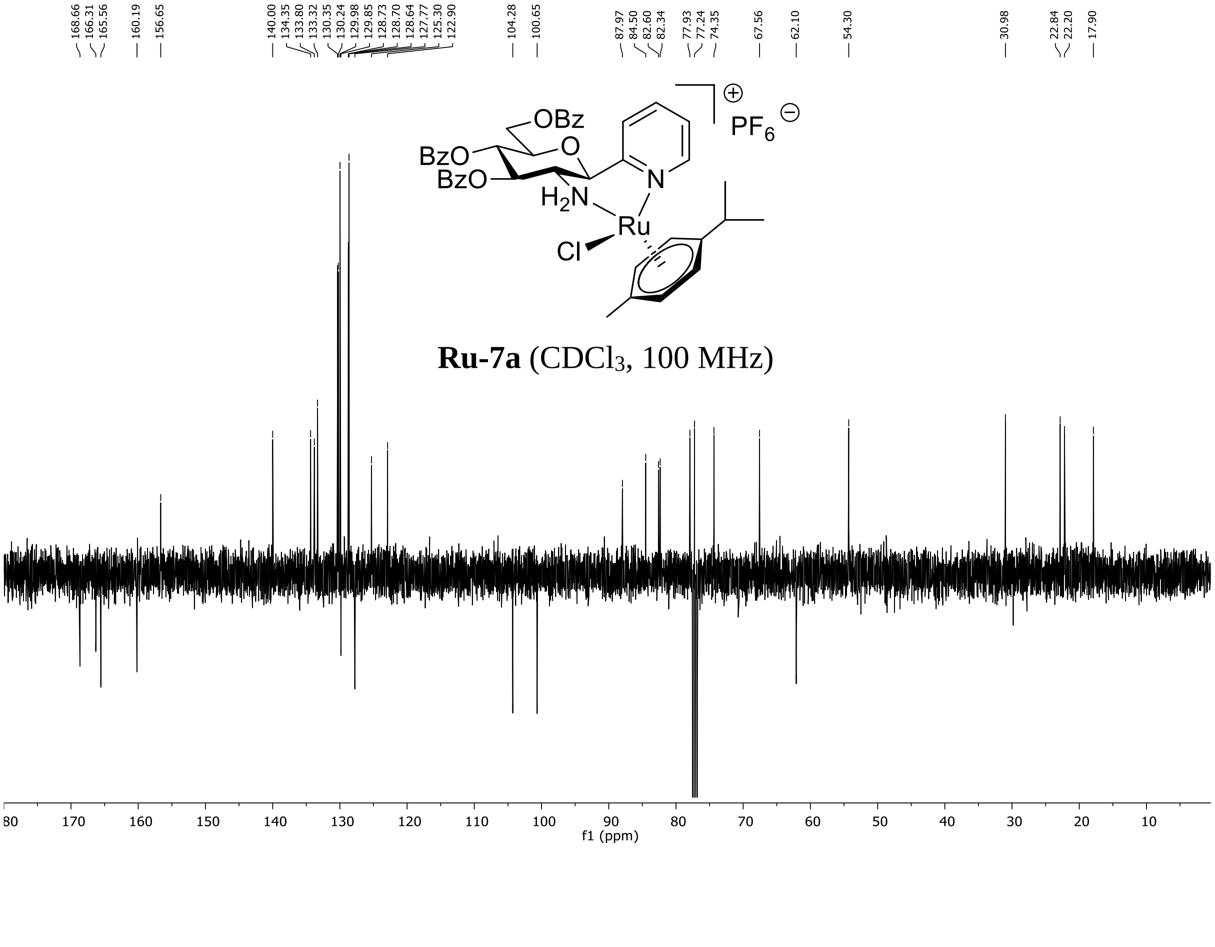

Ru-7a ($CDCl_3$, 100 MHz)

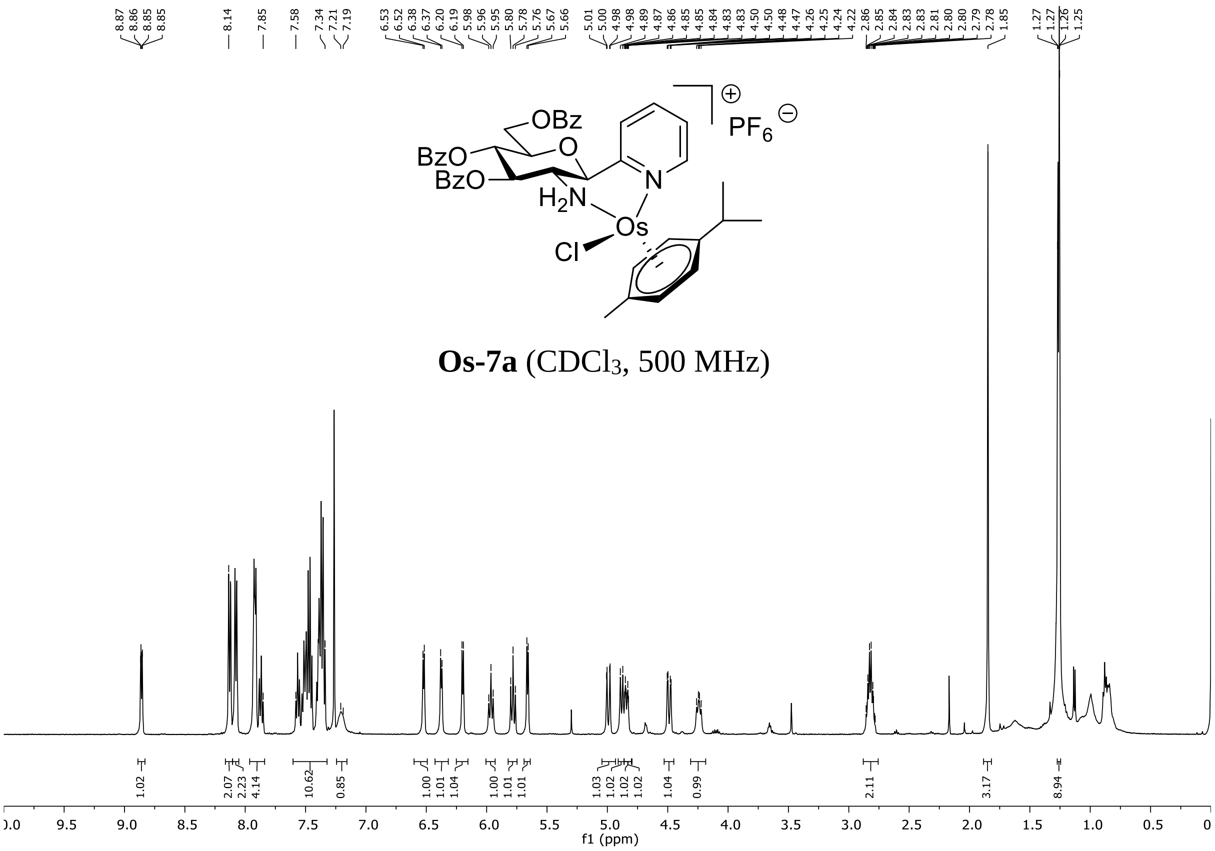

Os-7a (CDCl$_3$, 500 MHz)

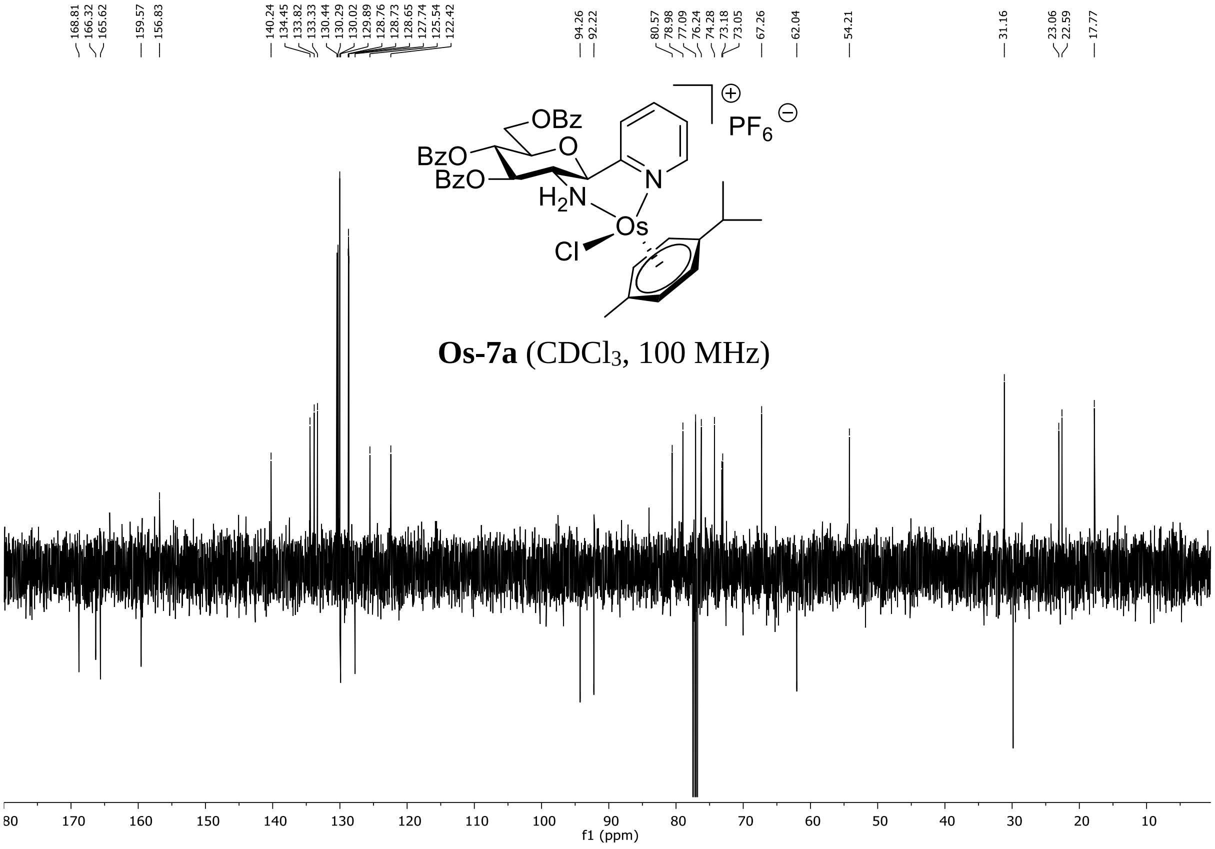

Os-7a (CDCl$_3$, 100 MHz)

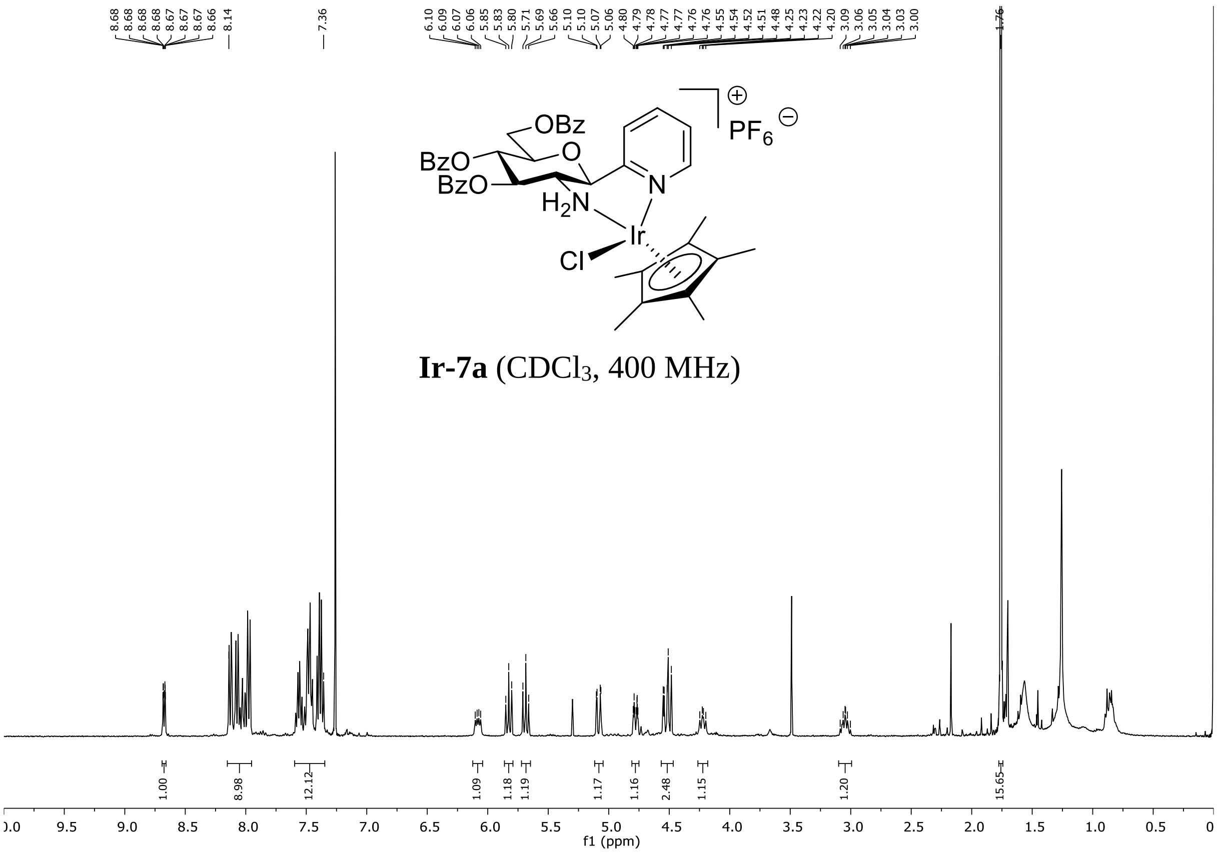
8.68 8.68 8.68 8.68 8.67 8.67 8.67 8.66 8.14
7.36
6.10 6.09 6.07 6.06 5.85 5.83 5.80 5.71 5.69 5.66 5.10 5.10 5.07 5.06 4.80 4.79 4.78 4.77 4.77 4.76 4.76 4.55 4.54 4.52 4.51 4.48 4.25 4.23 4.22 4.20 3.09 3.06 3.05 3.04 3.03 3.00
1.76
OBz
BzO
BzO
O
N
H_2N
Ir
Cl
PF_6
Ir-7a ($CDCl_3$, 400 MHz)
1.00
8.98
12.12
1.09
1.18
1.19
1.17
1.16
2.48
1.15
1.20
15.65
f1 (ppm)

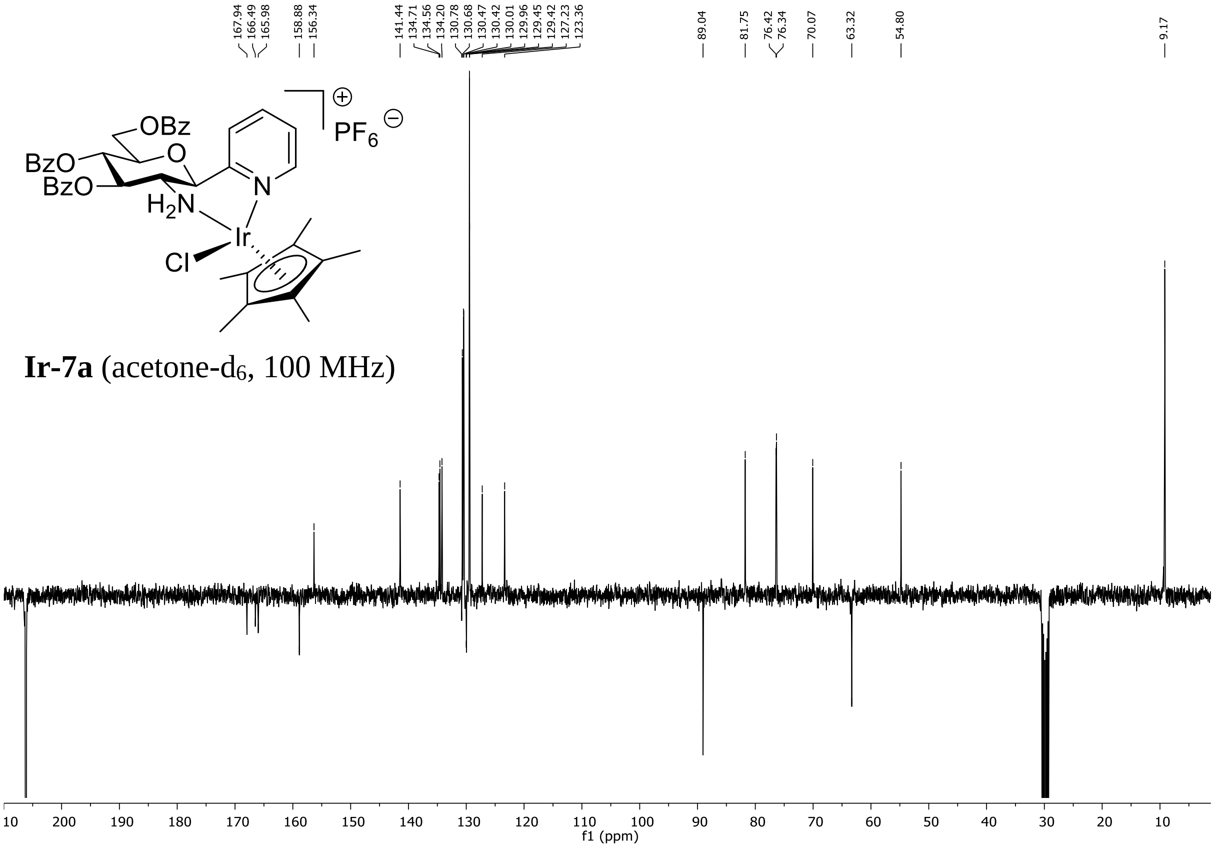
167.94 166.49 165.98
158.88 156.34
141.44 134.71 134.56 134.20 130.78 130.68 130.47 130.42 130.01 129.96 129.45 129.42 127.23 123.36
89.04
81.75
76.42 76.34
70.07
63.32
54.80
9.17
OBz
BzO
BzO
O
N
H_2N
Ir
Cl
PF_6
Ir-7a (acetone-d_6, 100 MHz)
f1 (ppm)

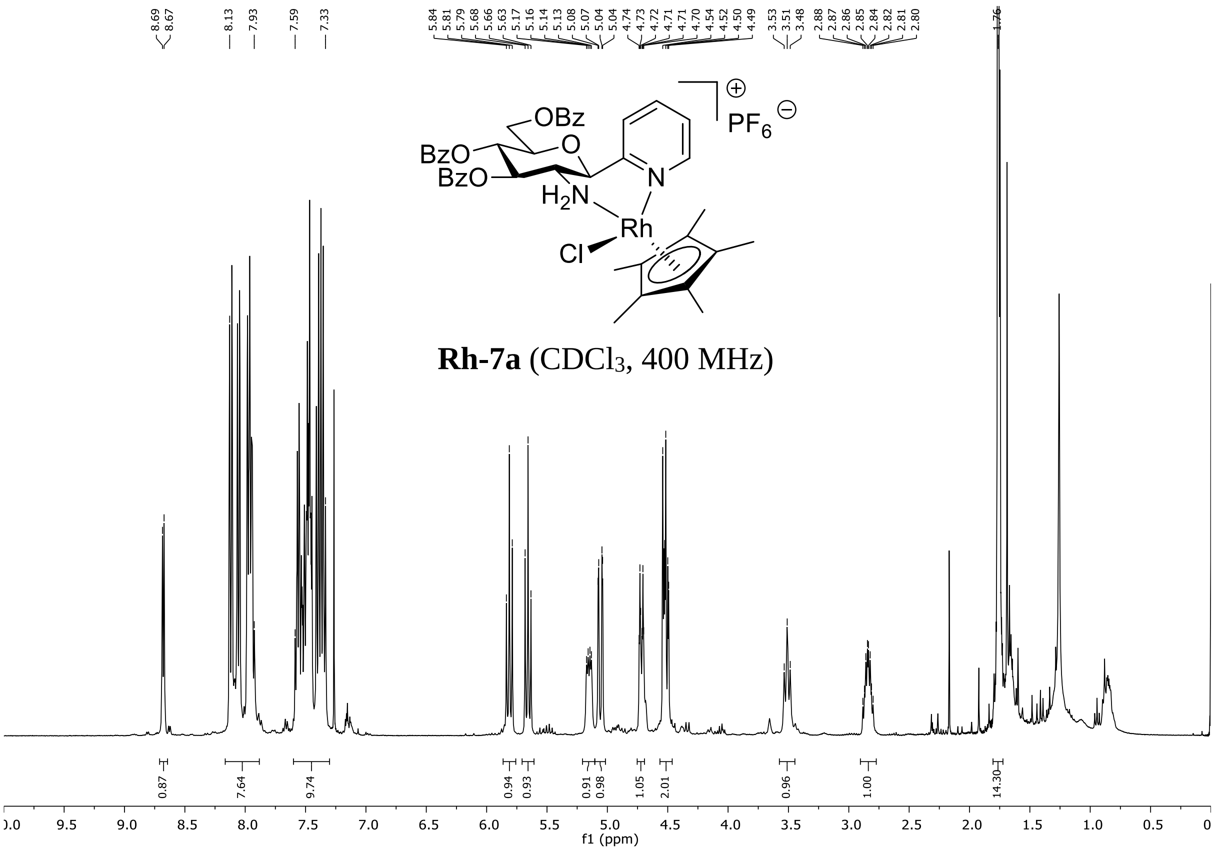

Rh-7a ($CDCl_3$, 400 MHz)

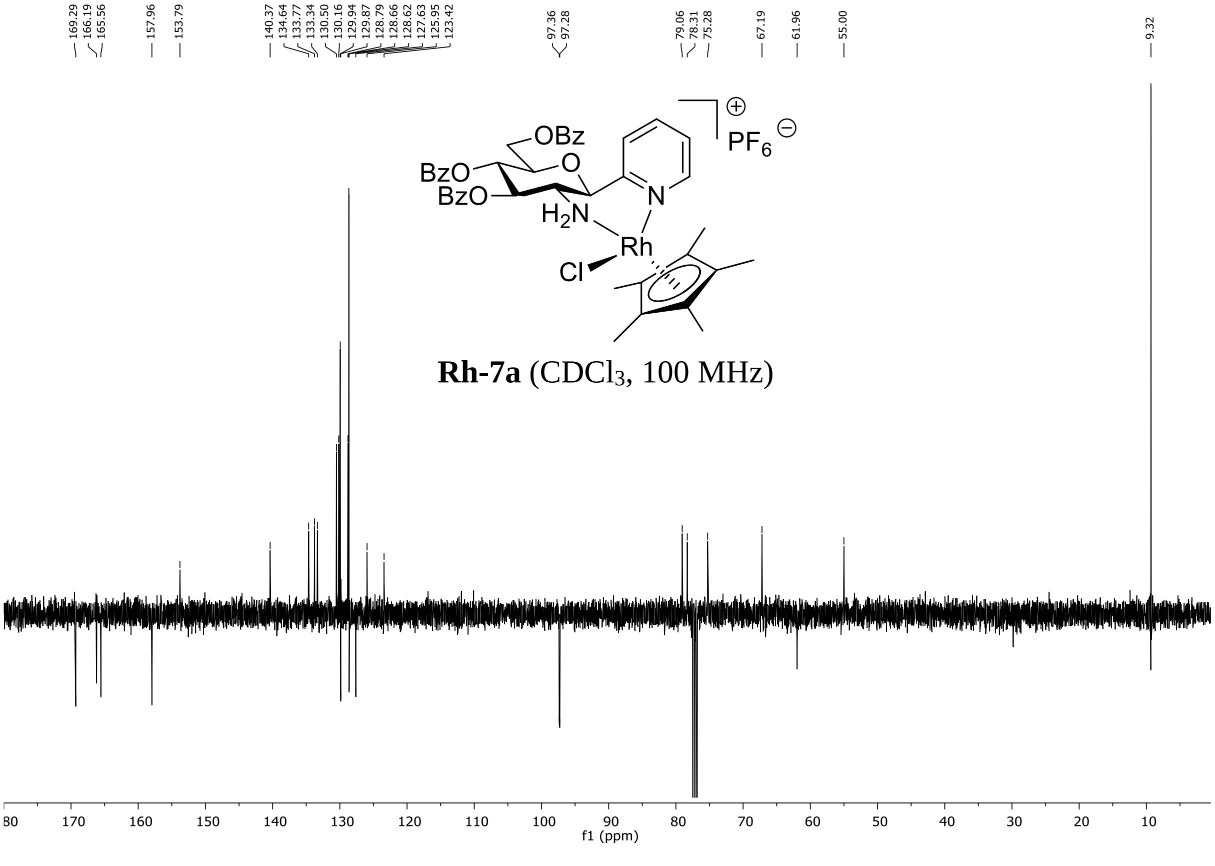

Rh-7a ($CDCl_3$, 100 MHz)

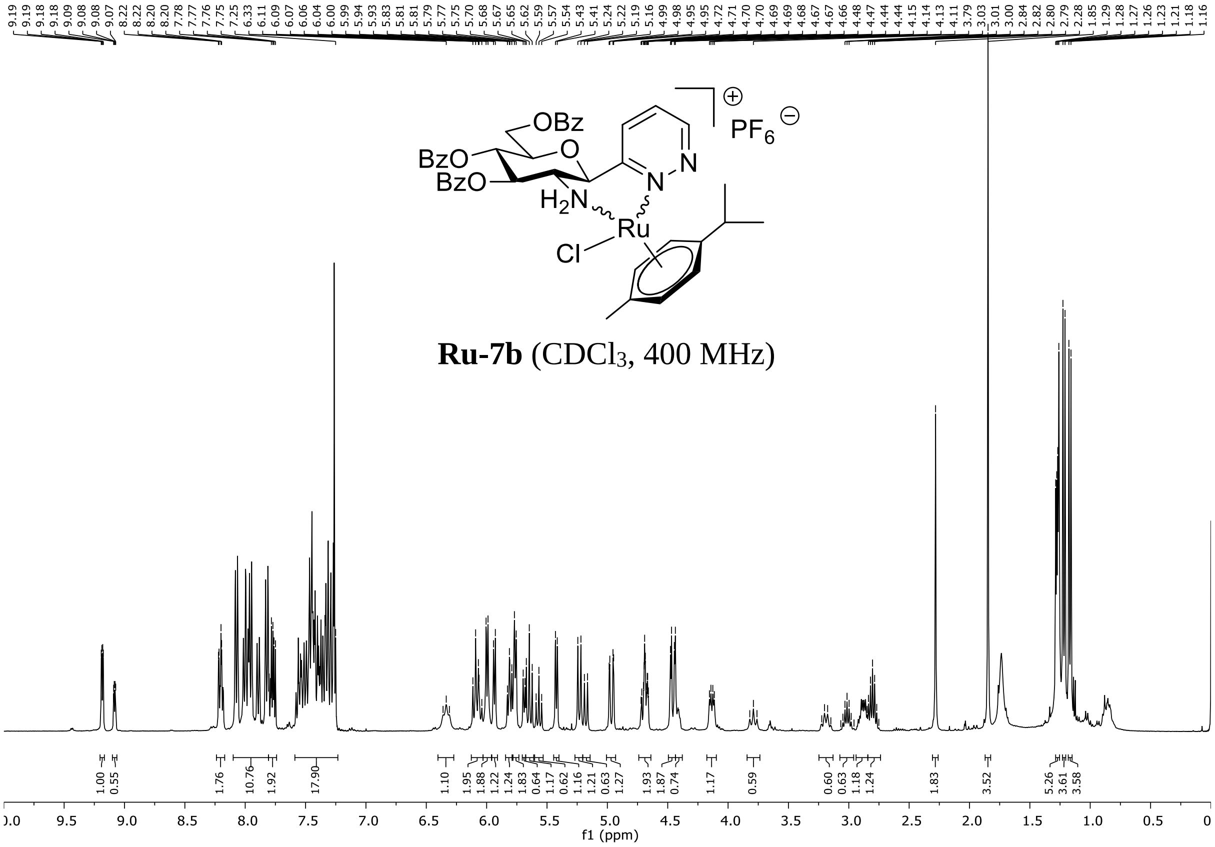

Ru-7b ($CDCl_3$, 400 MHz)

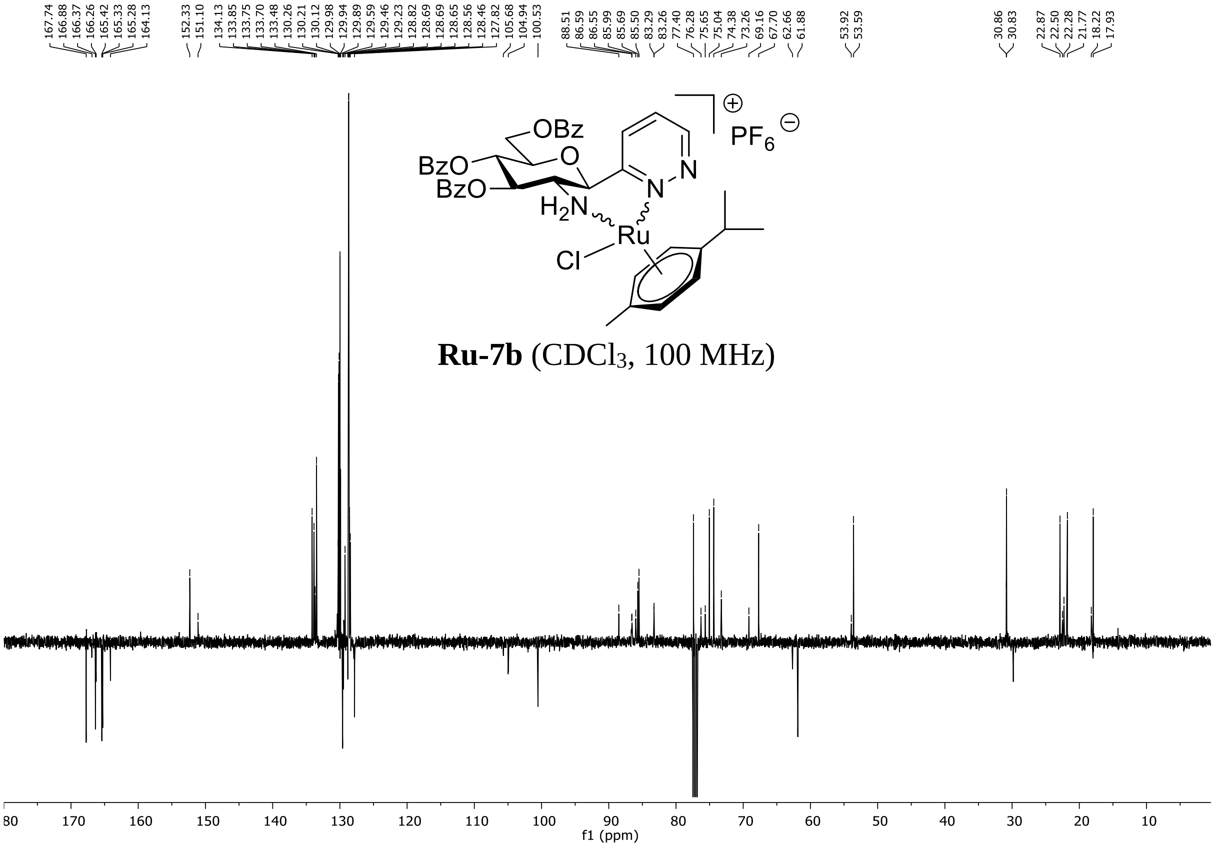

Ru-7b ($CDCl_3$, 100 MHz)

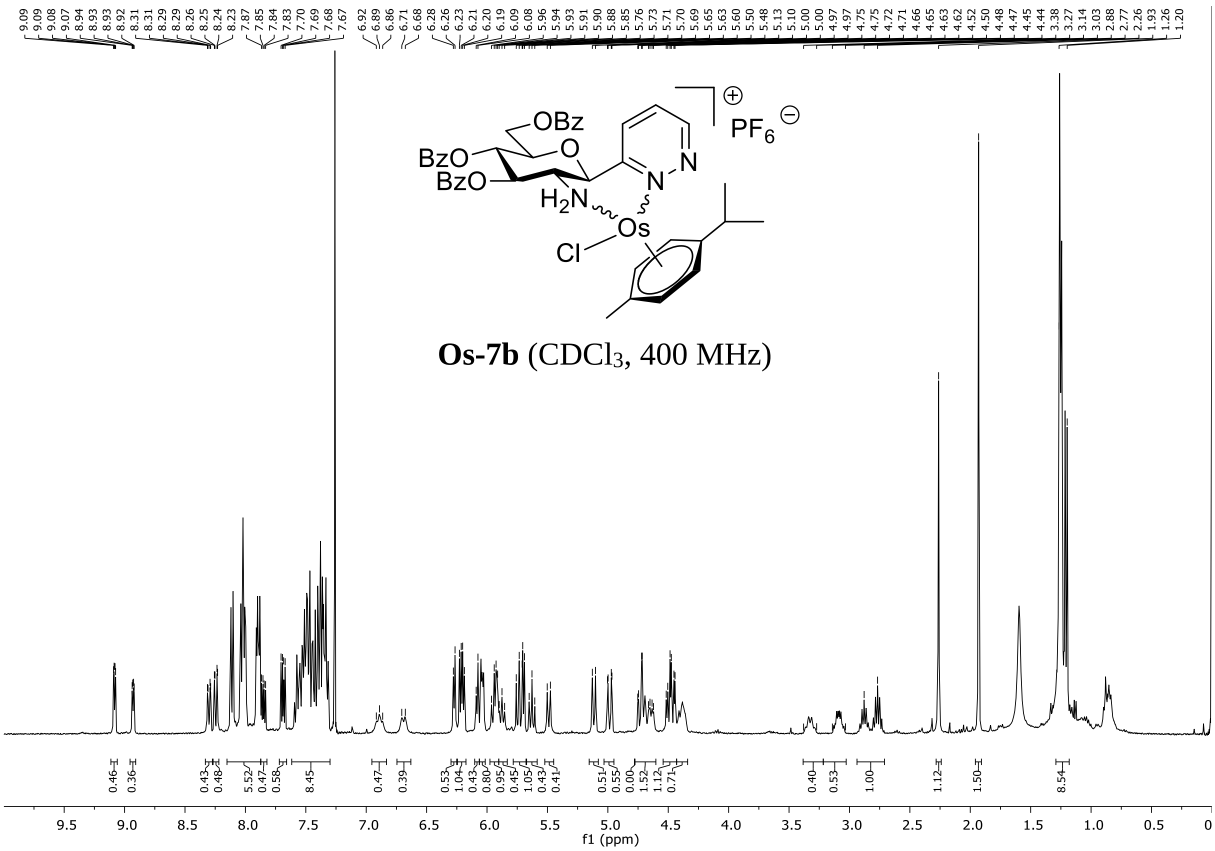

Os-7b ($CDCl_3$, 400 MHz)

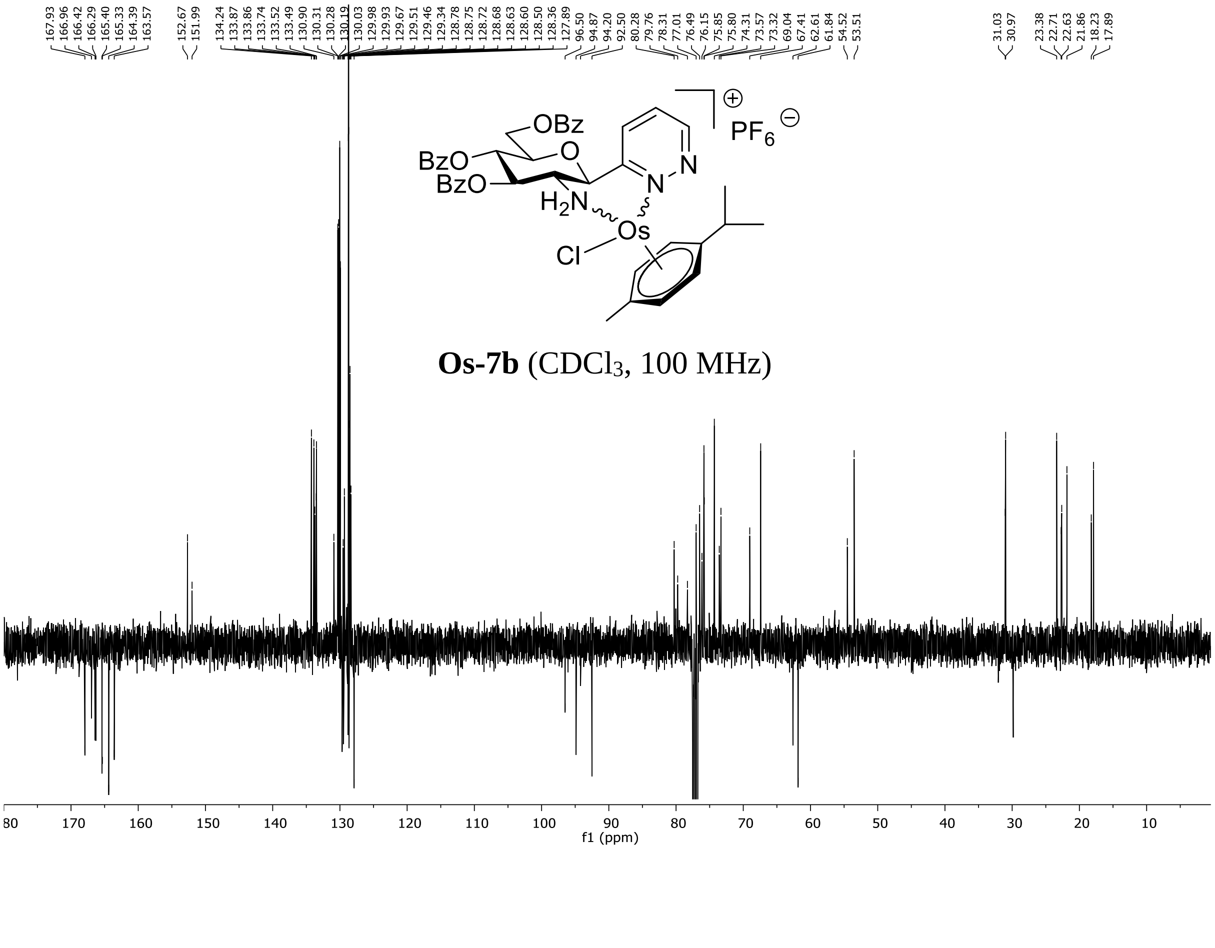

Os-7b ($CDCl_3$, 100 MHz)

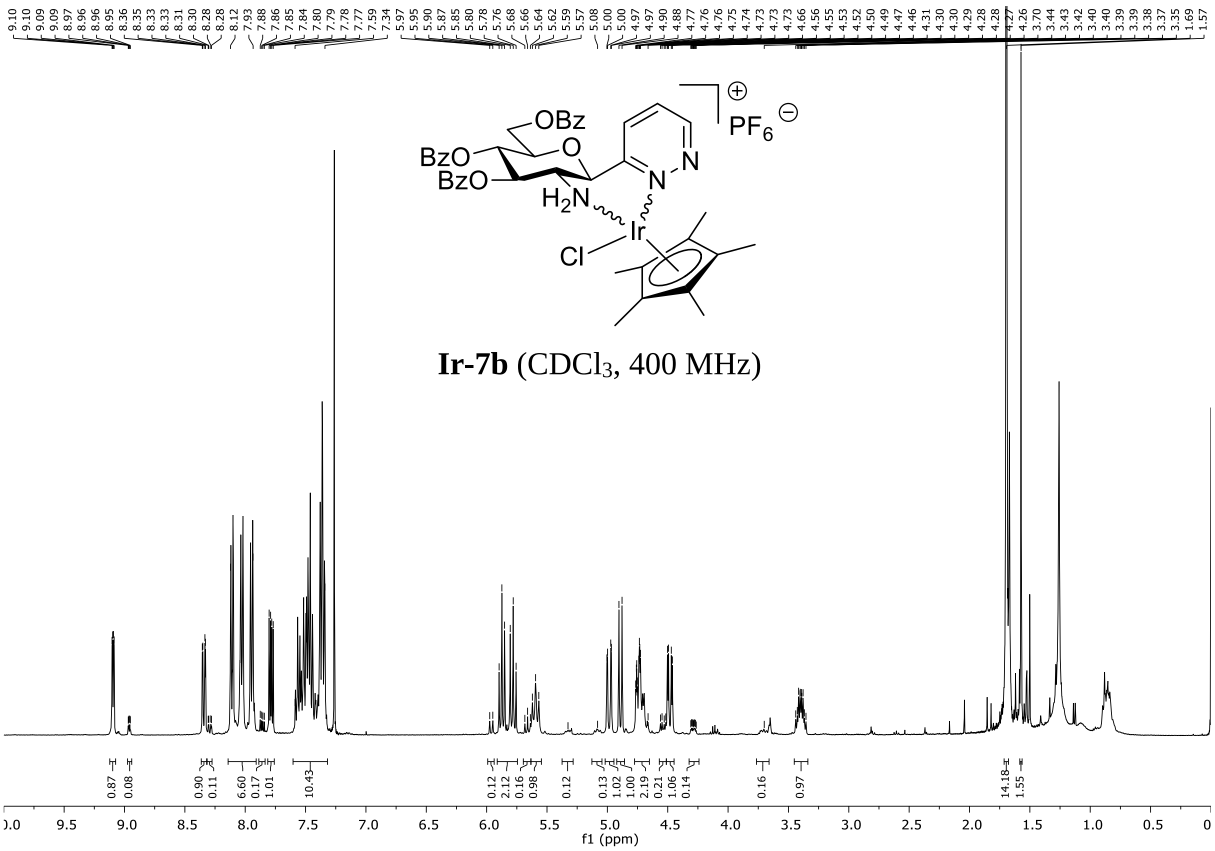
PF6
BzO
BzO
OBz
H2N
Ir
Cl
Ir-7b (CDCl3, 400 MHz)
f1 (ppm)

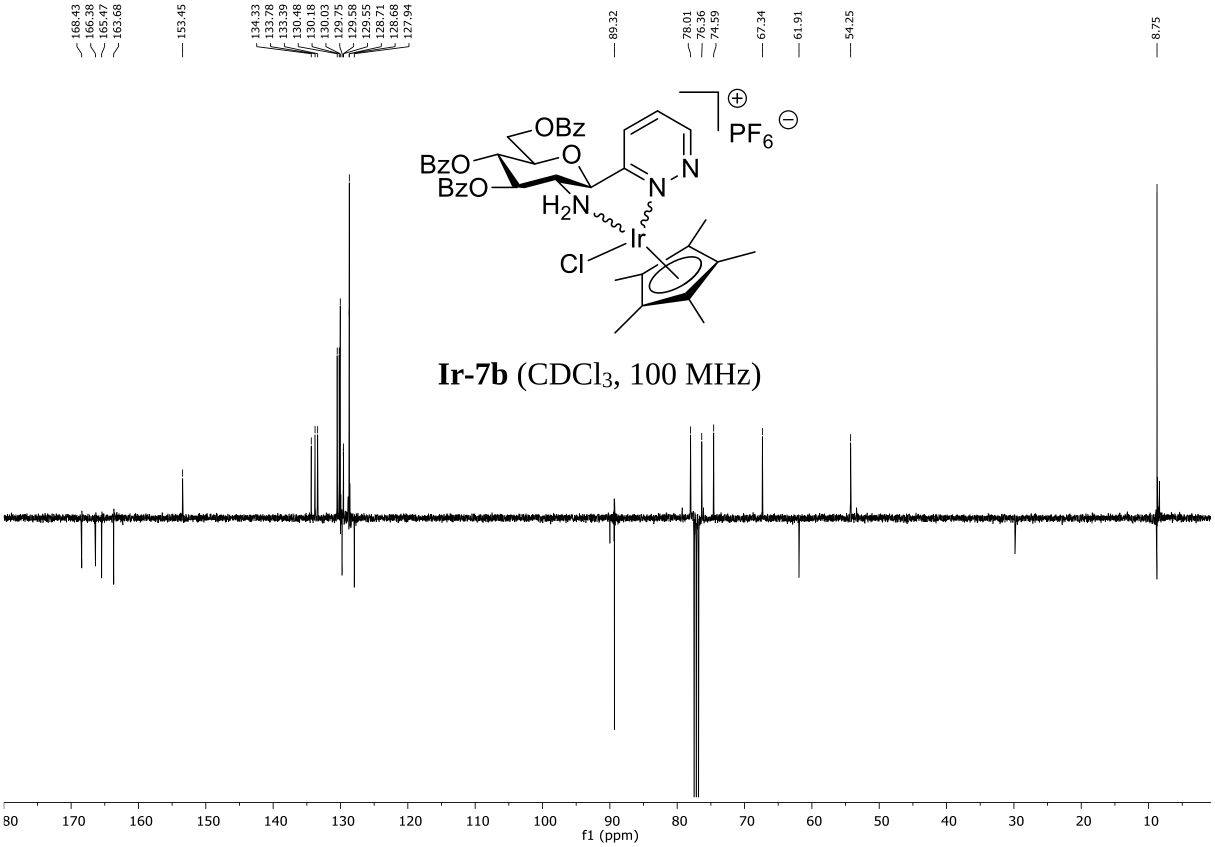
PF6
BzO
BzO
OBz
H2N
Ir
Cl
Ir-7b (CDCl3, 100 MHz)
f1 (ppm)

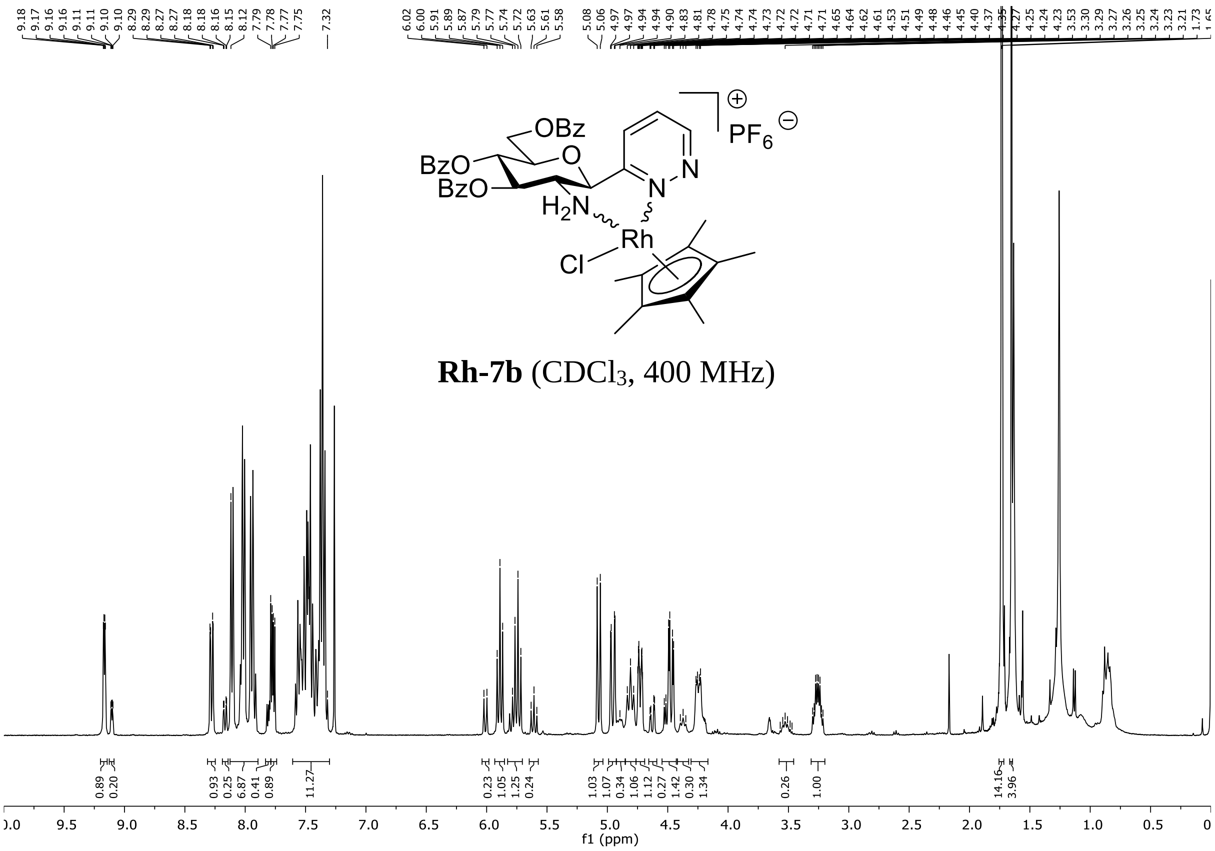

Rh-7b ($CDCl_3$, 400 MHz)

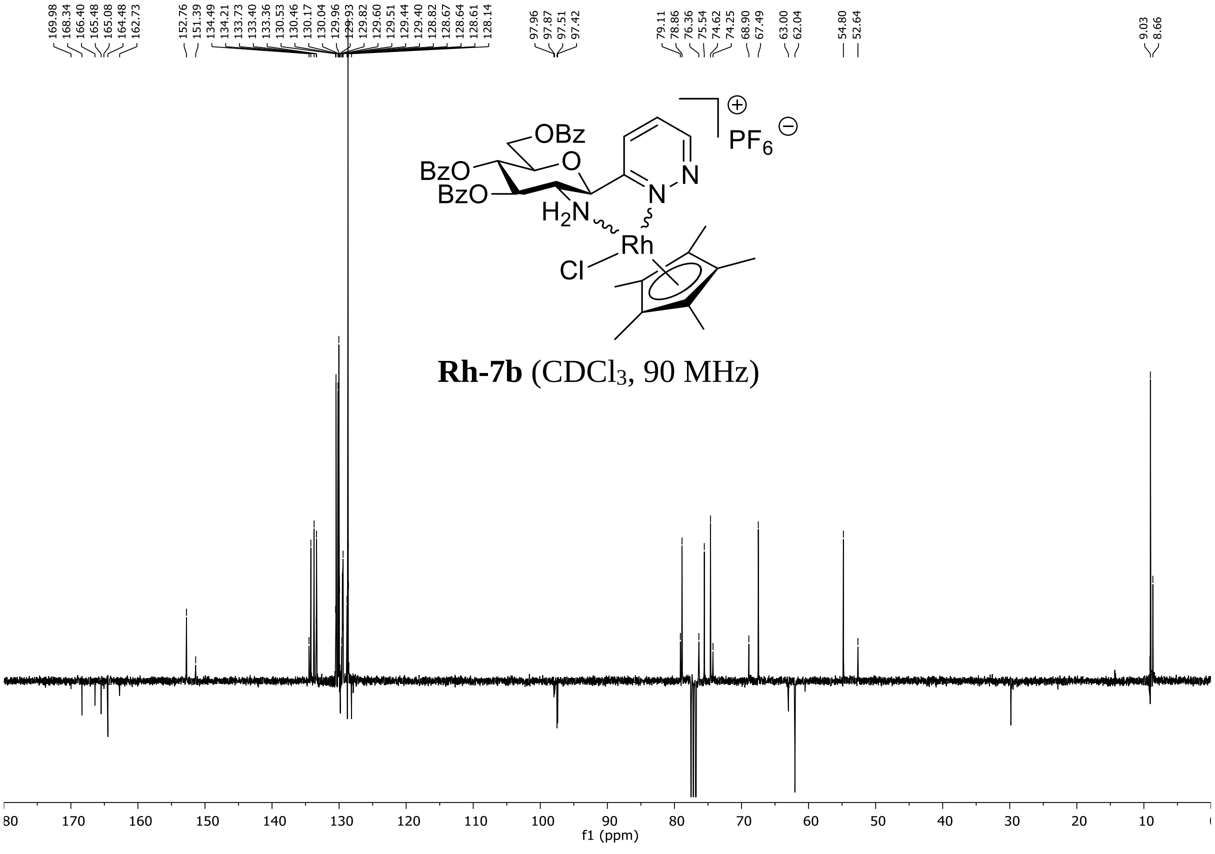

Rh-7b ($CDCl_3$, 90 MHz)

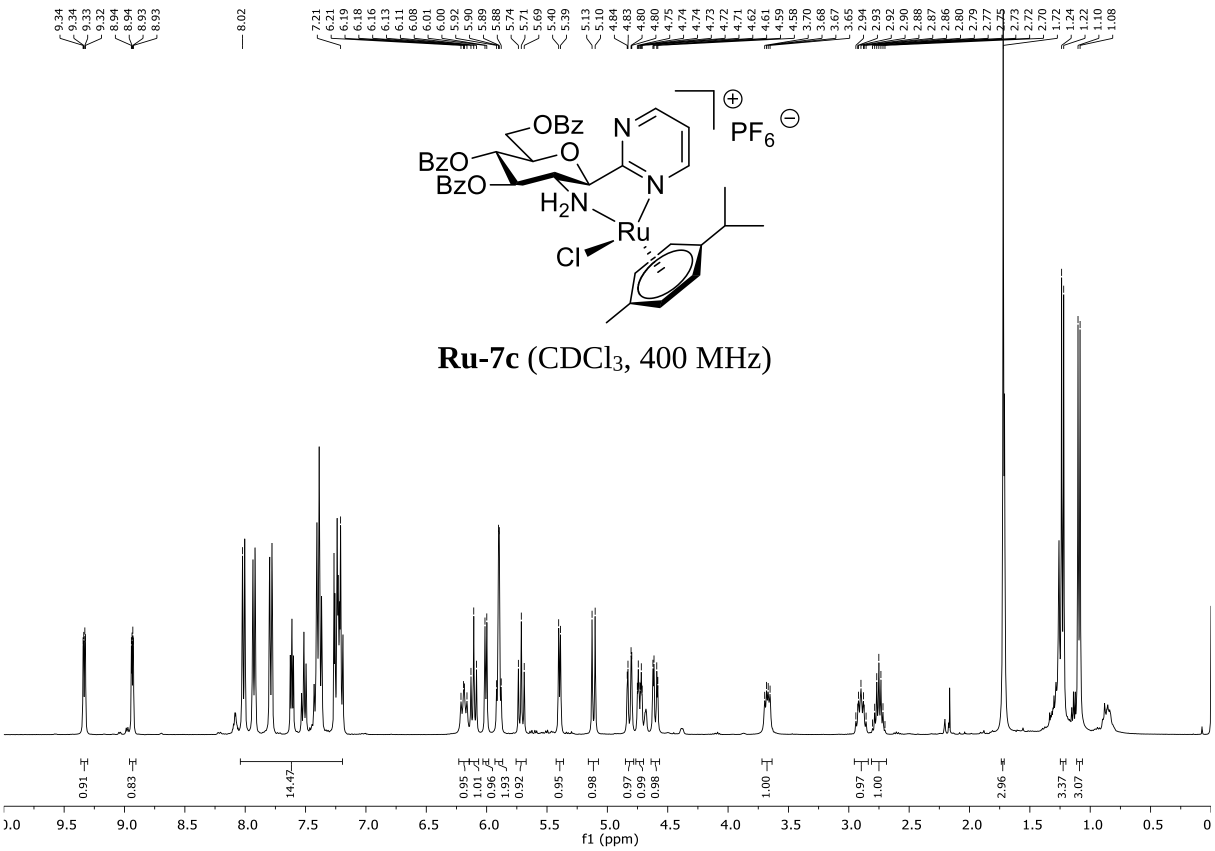

Ru-7c ($CDCl_3$, 400 MHz)

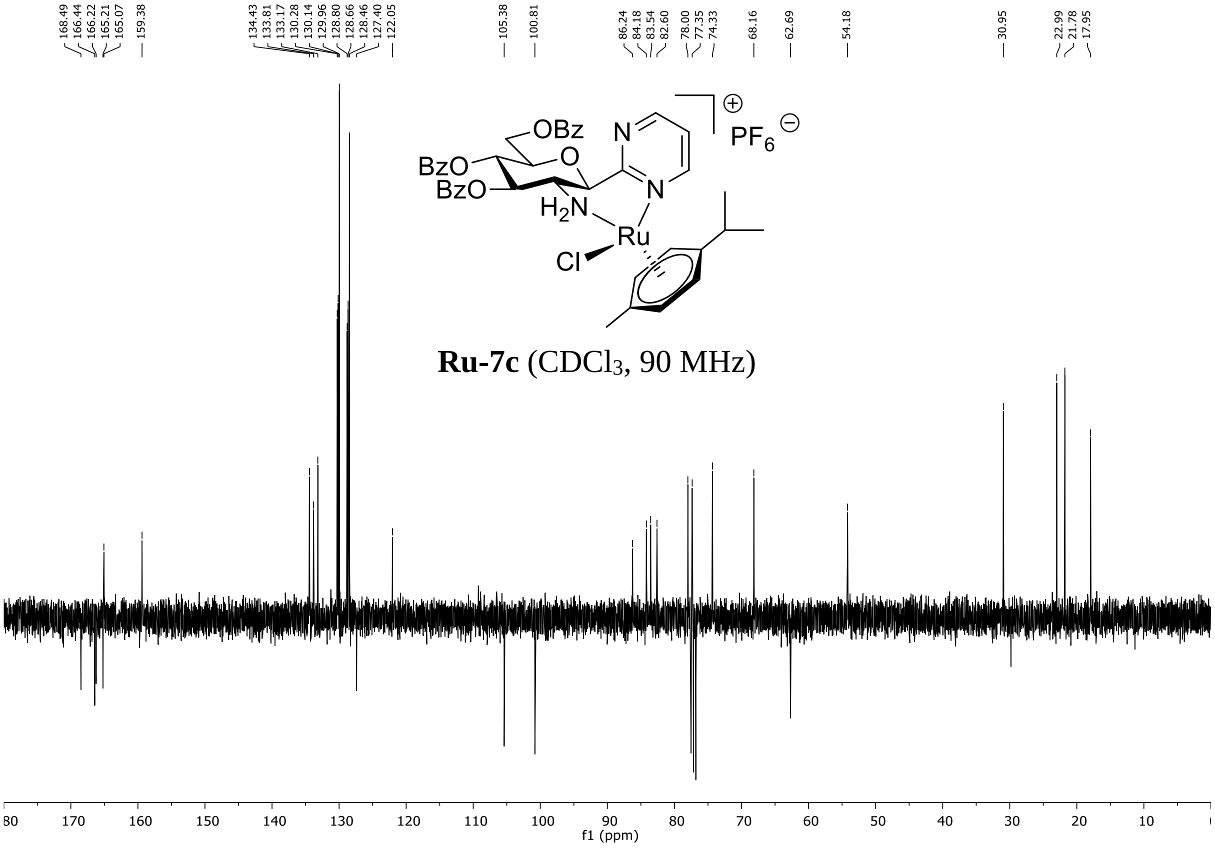

Ru-7c ($CDCl_3$, 90 MHz)

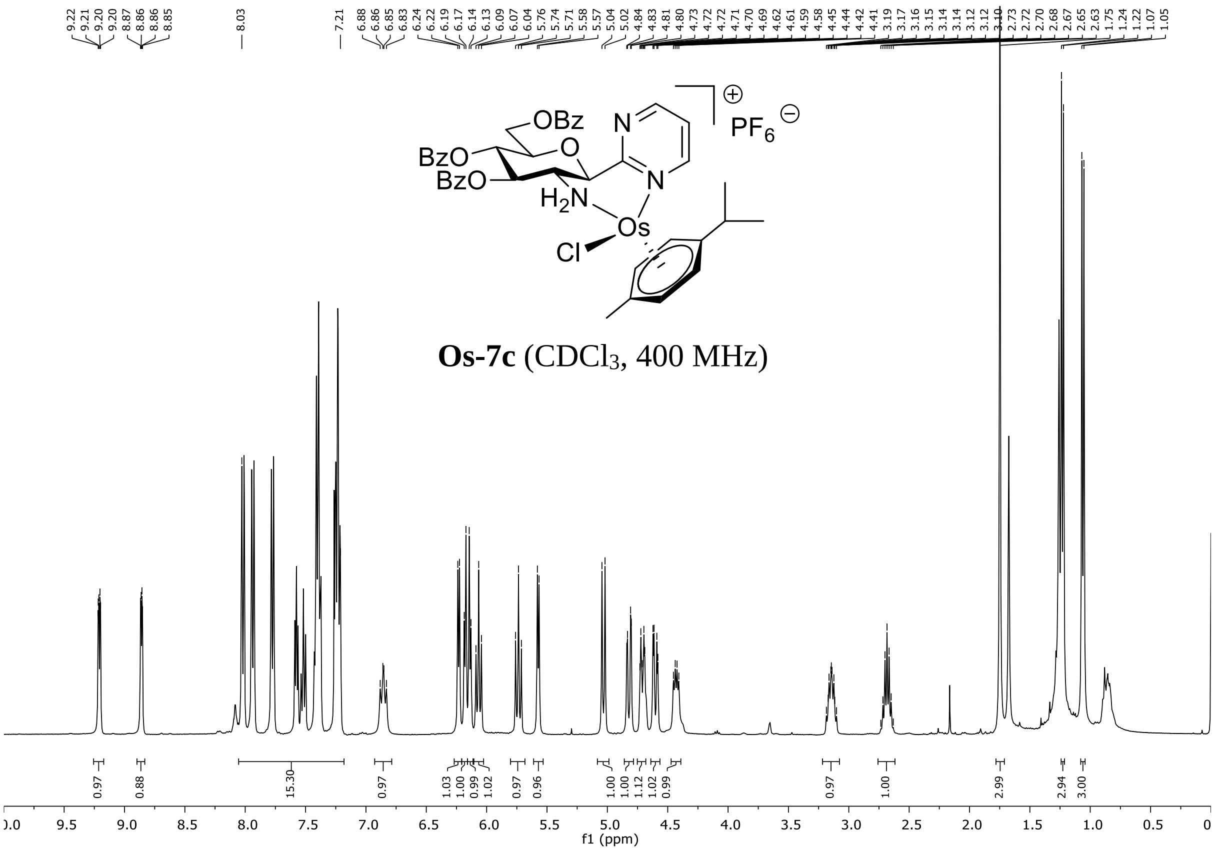

Os-7c (CDCl$_3$, 400 MHz)

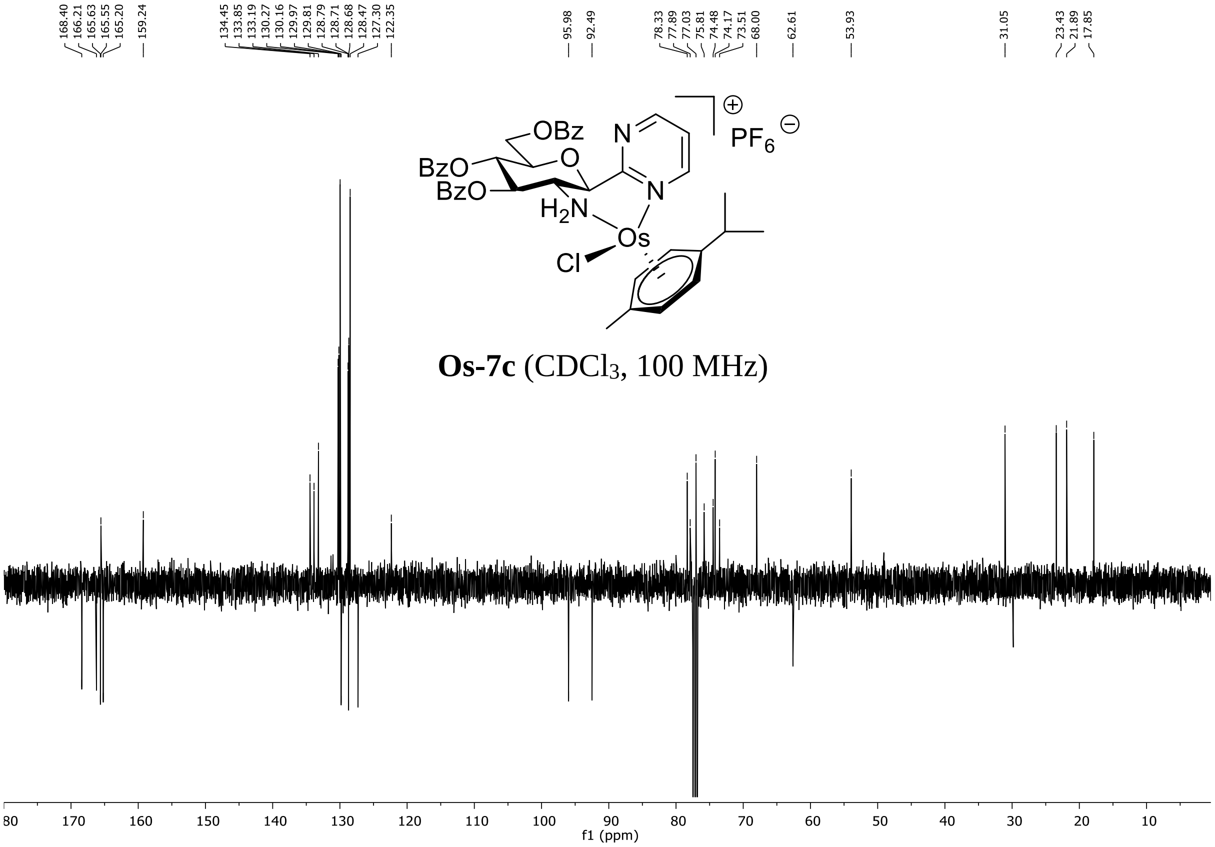

Os-7c (CDCl$_3$, 100 MHz)

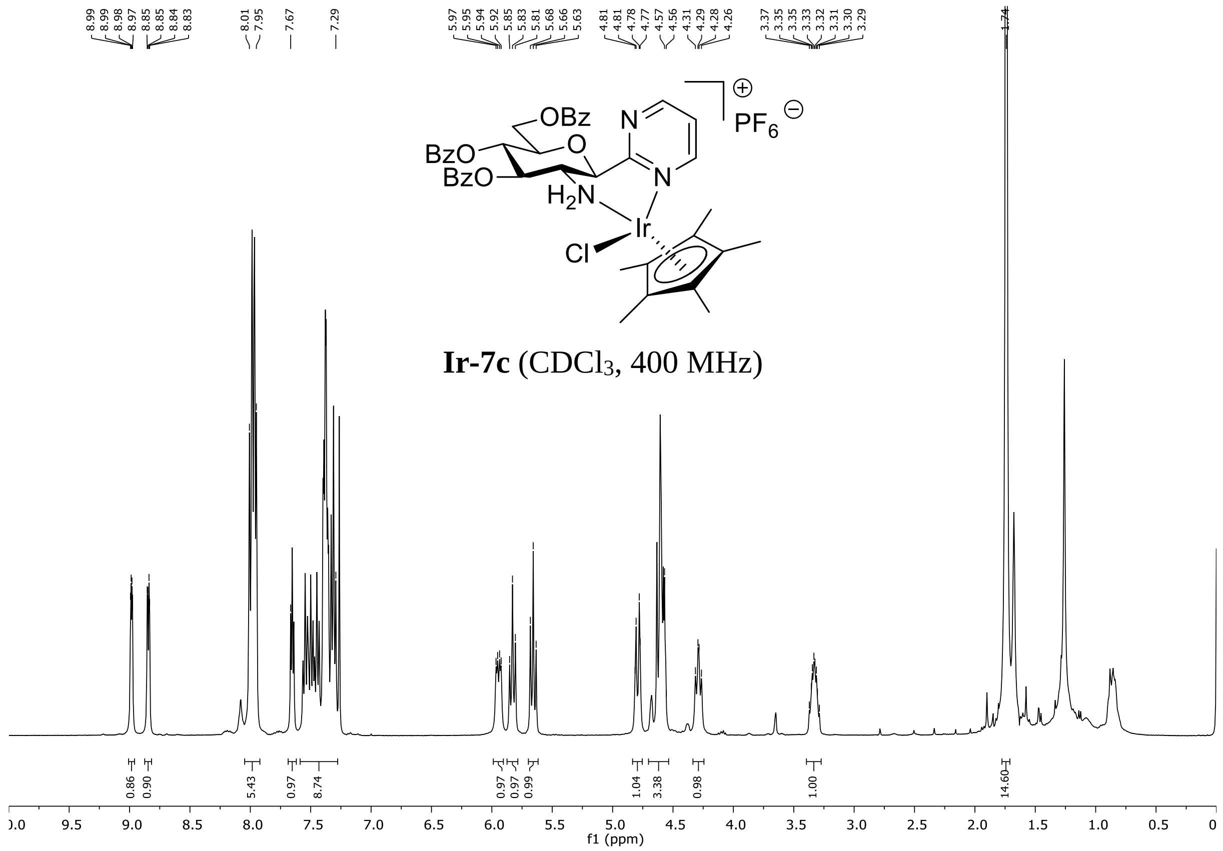

Ir-7c ($CDCl_3$, 400 MHz)
BzO
BzO
OBz
O
N
N
H2N
Ir
Cl
PF6
f1 (ppm)

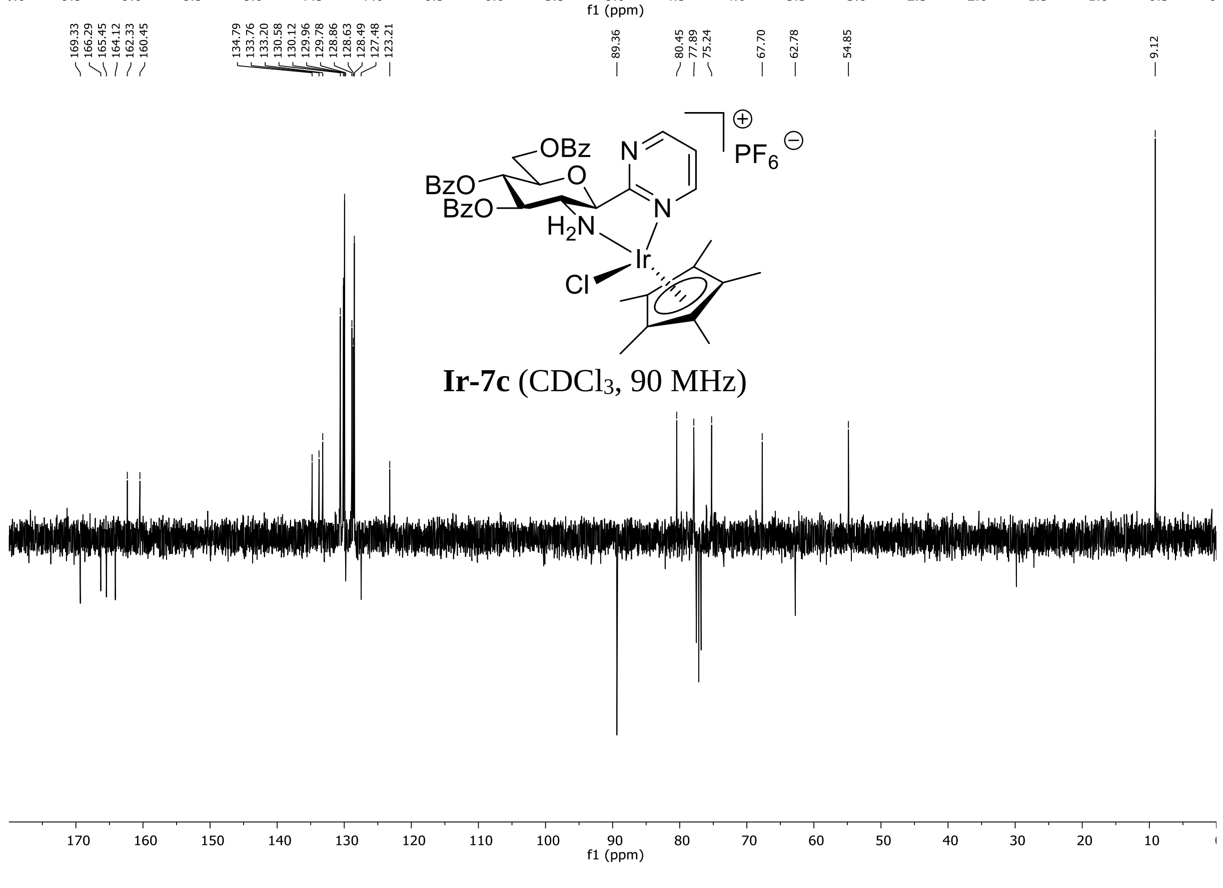

Ir-7c ($CDCl_3$, 90 MHz)
f1 (ppm)

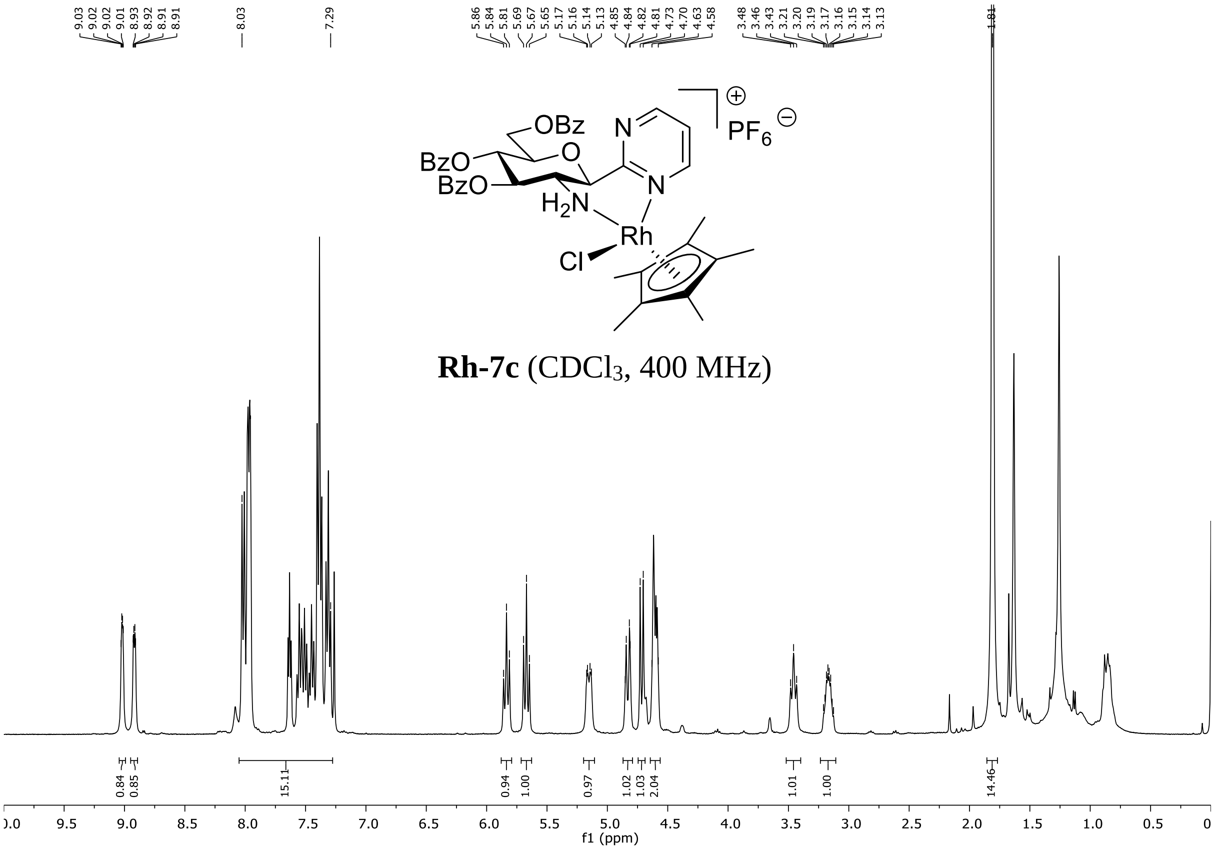
Rh-7c (CDCl3, 400 MHz)
f1 (ppm)

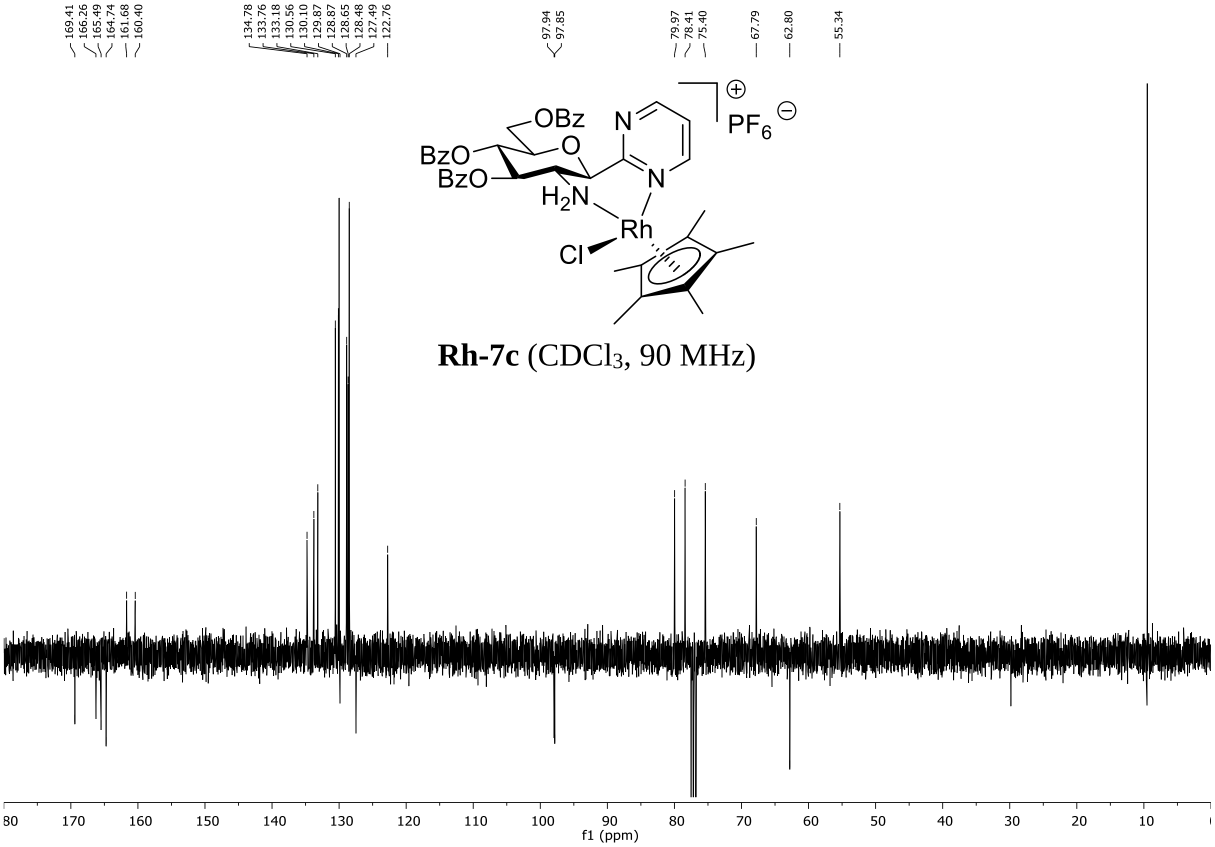
Rh-7c (CDCl3, 90 MHz)
f1 (ppm)

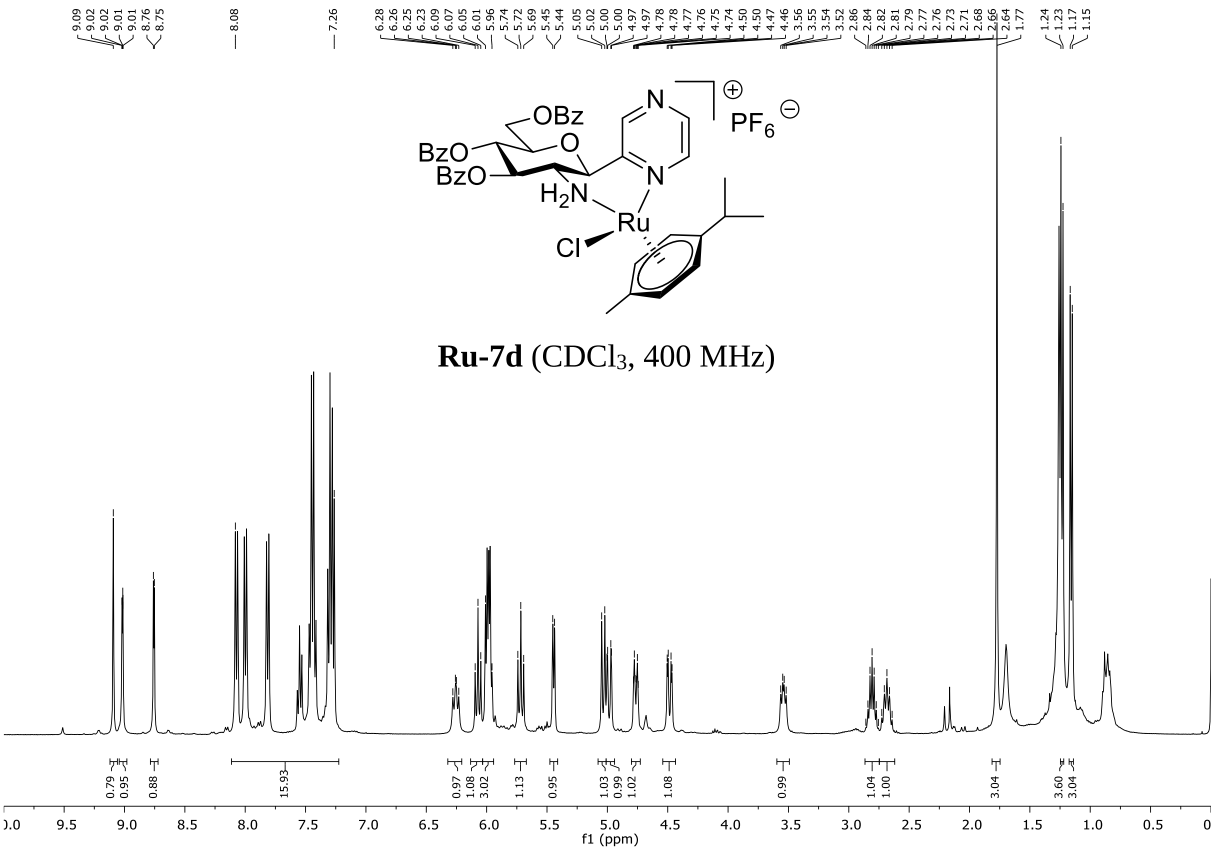

Ru-7d ($CDCl_3$, 400 MHz)

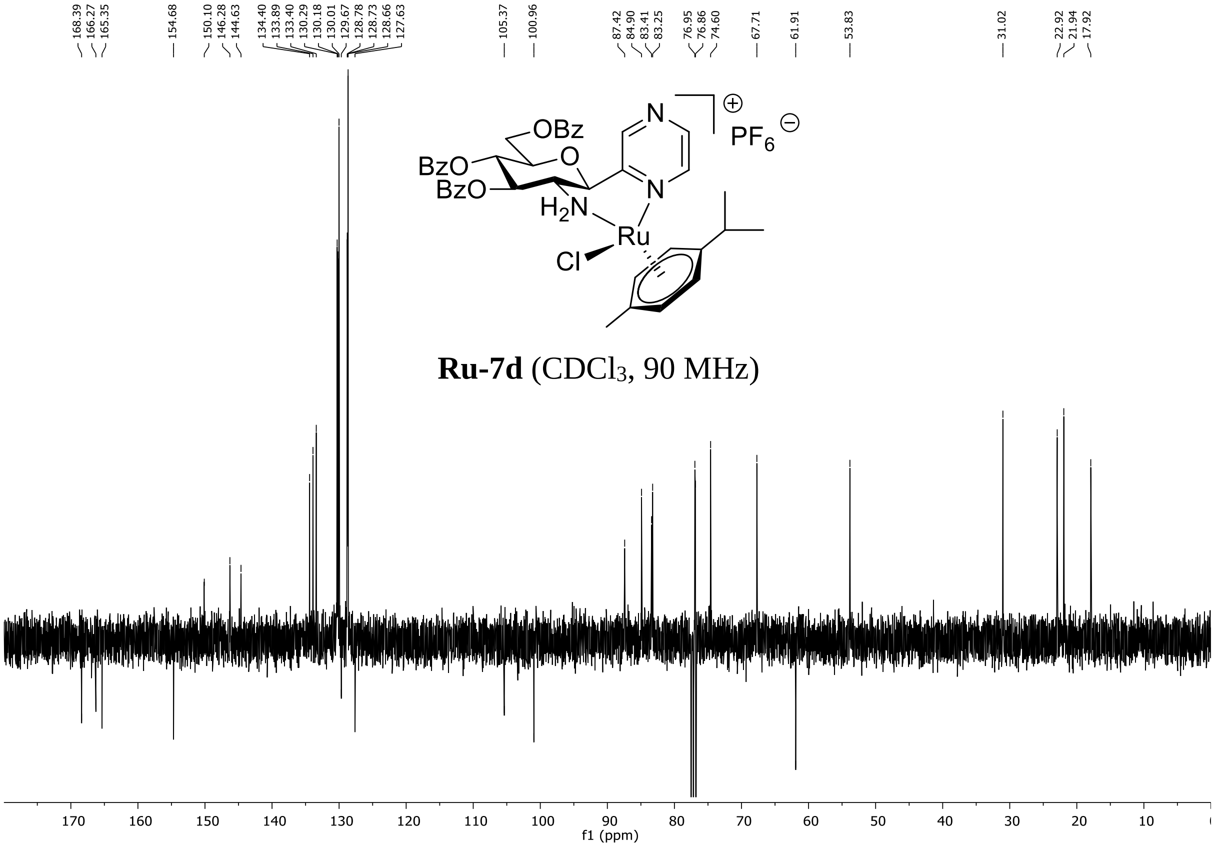

Ru-7d ($CDCl_3$, 90 MHz)

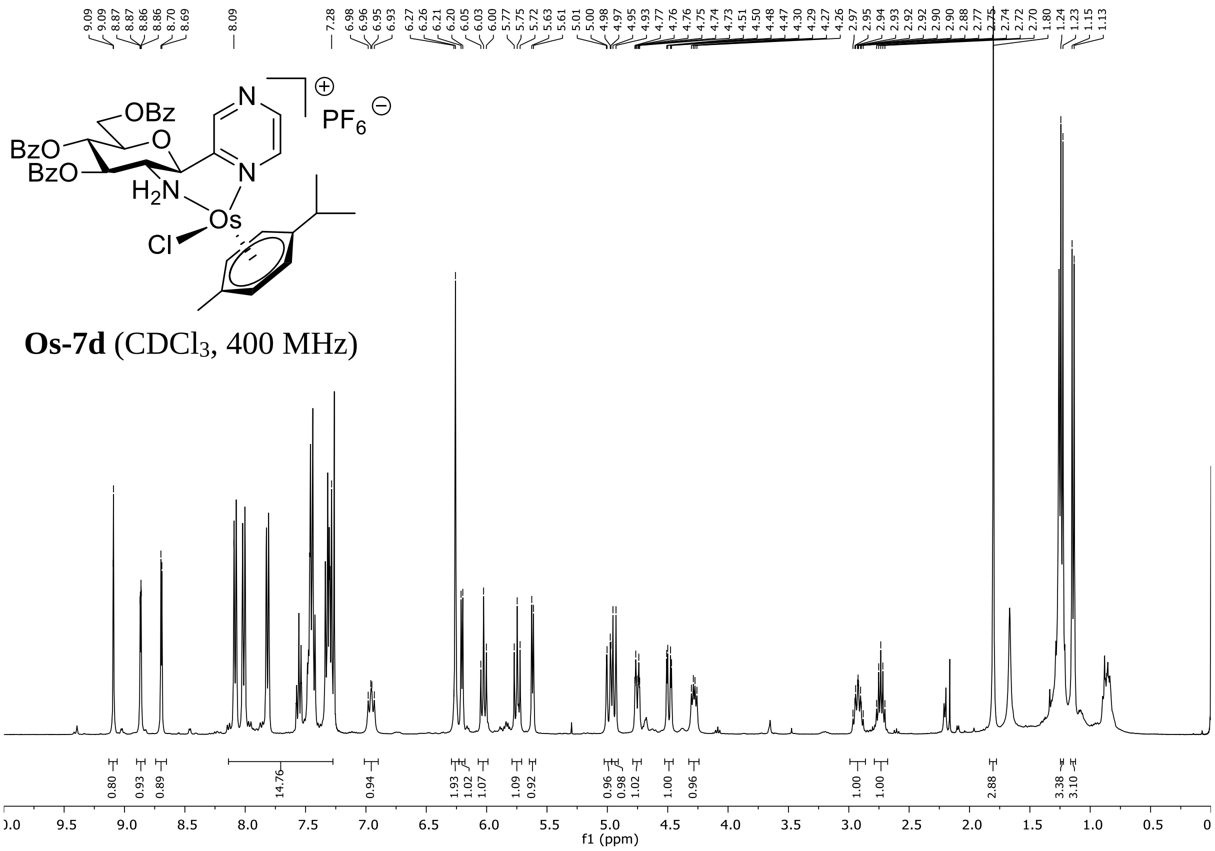
OBz
BzO
BzO
O
H2N
N
N
Os
Cl
PF6
Os-7d (CDCl3, 400 MHz)
f1 (ppm)

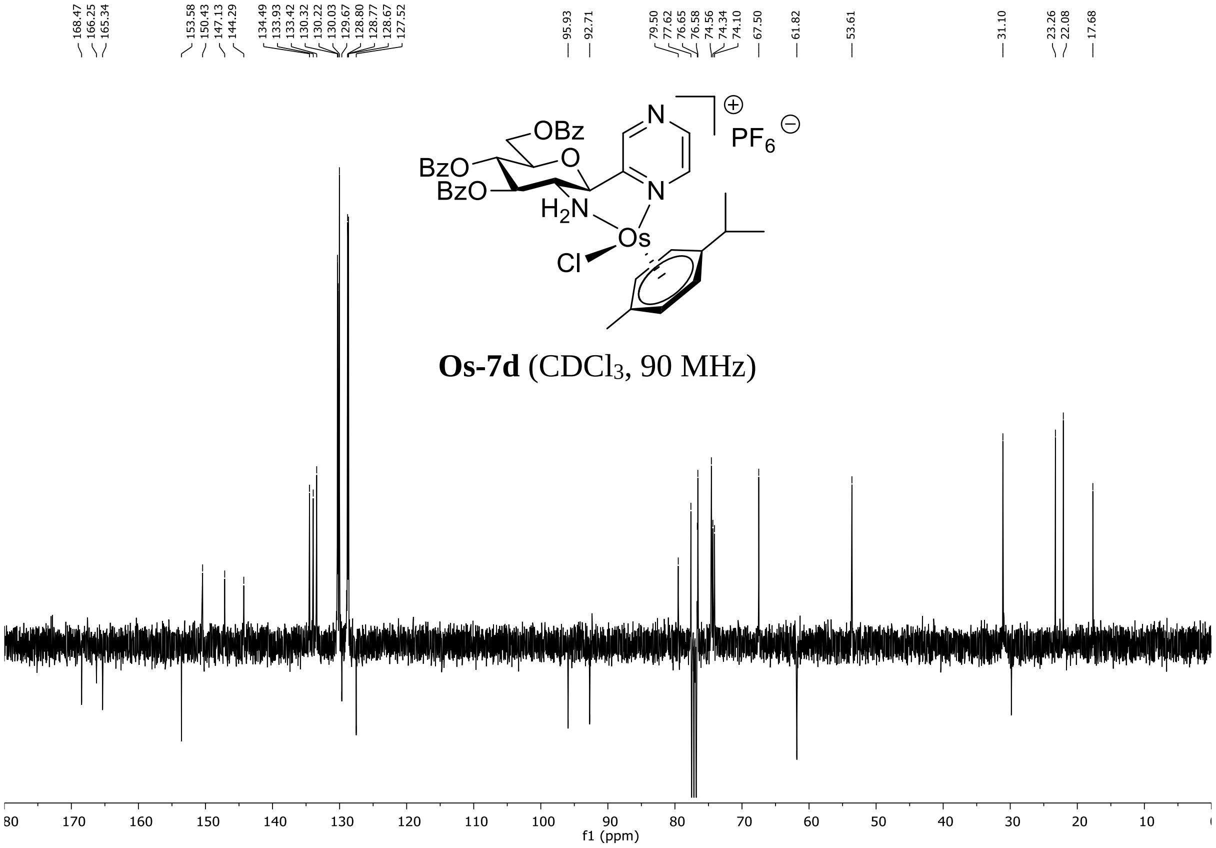
OBz
BzO
BzO
O
H2N
N
N
Os
Cl
PF6
Os-7d (CDCl3, 90 MHz)
f1 (ppm)

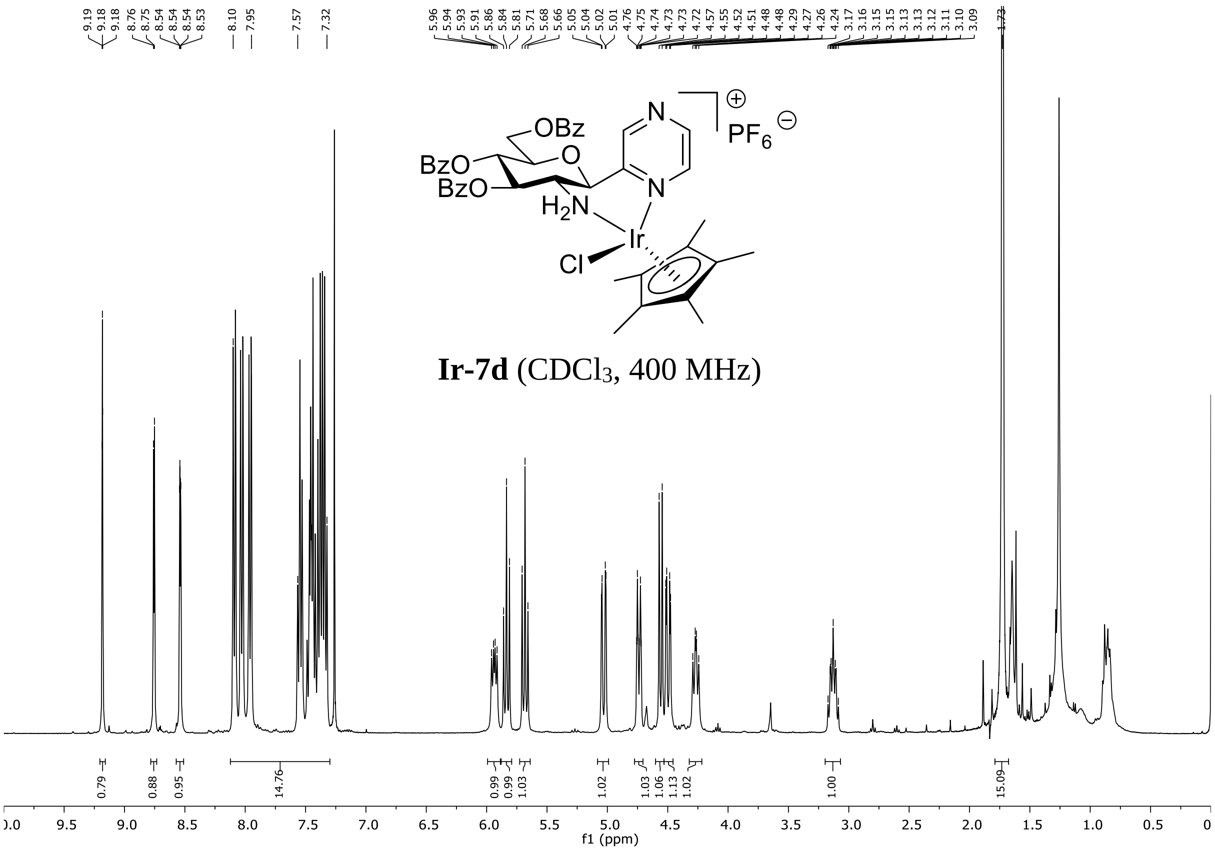
OBz
BzO
BzO
O
N
N
H2N
Ir
Cl
PF6
Ir-7d (CDCl3, 400 MHz)
f1 (ppm)

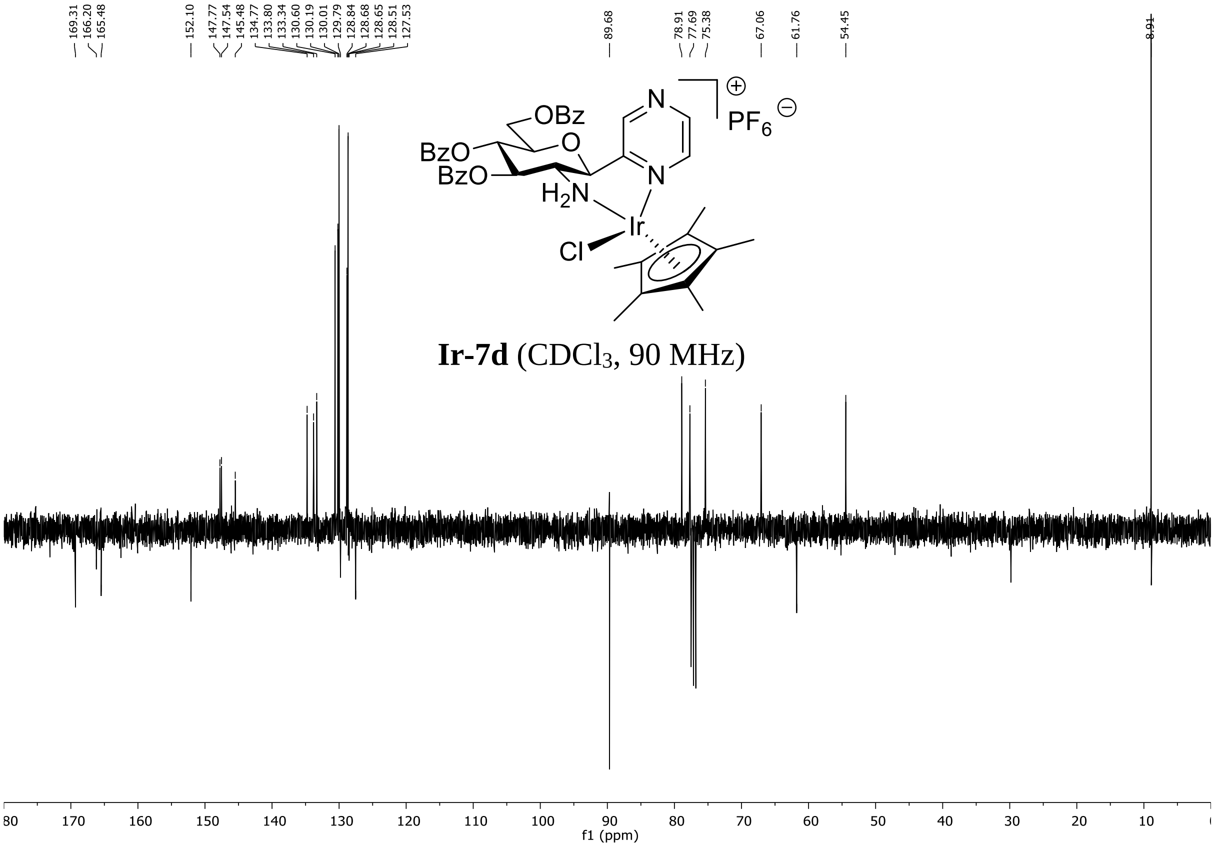
OBz
BzO
BzO
O
N
N
H2N
Ir
Cl
PF6
Ir-7d (CDCl3, 90 MHz)
f1 (ppm)

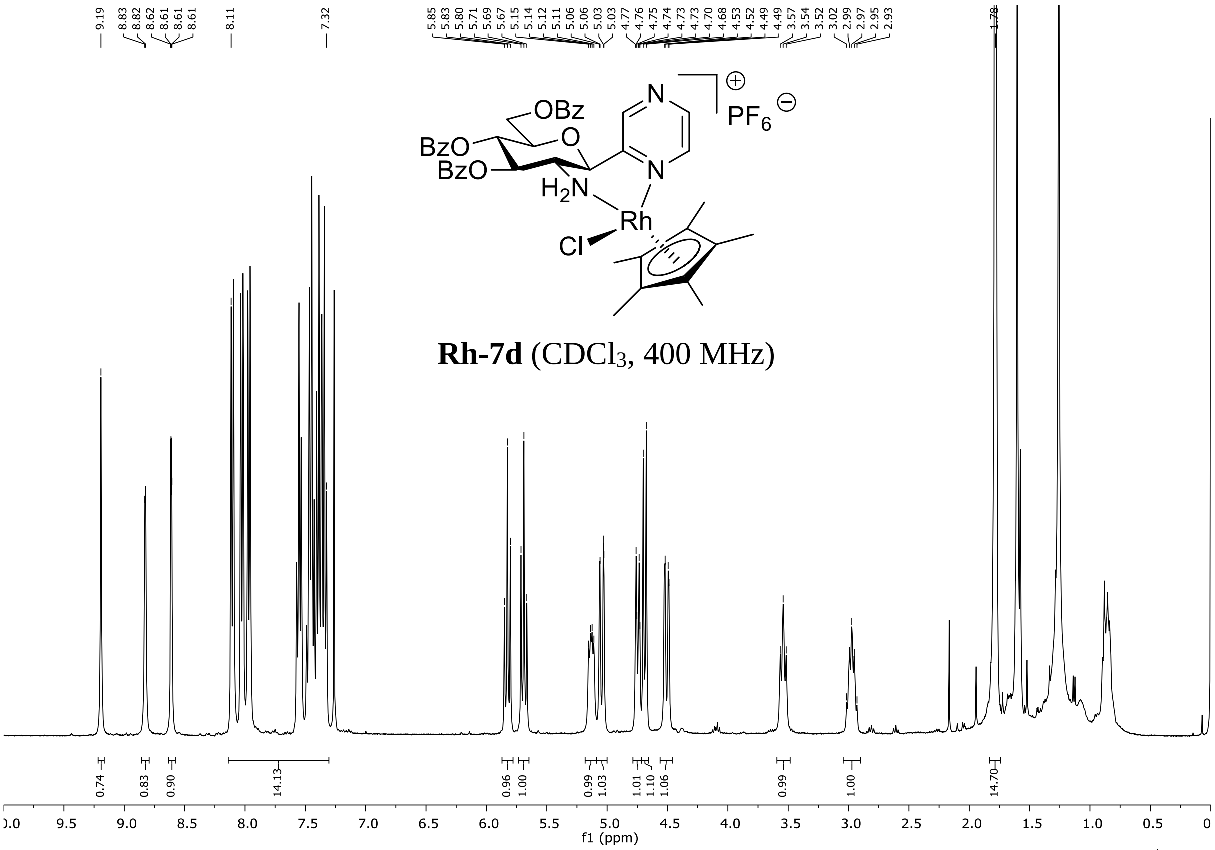

Rh-7d ($CDCl_3$, 400 MHz)

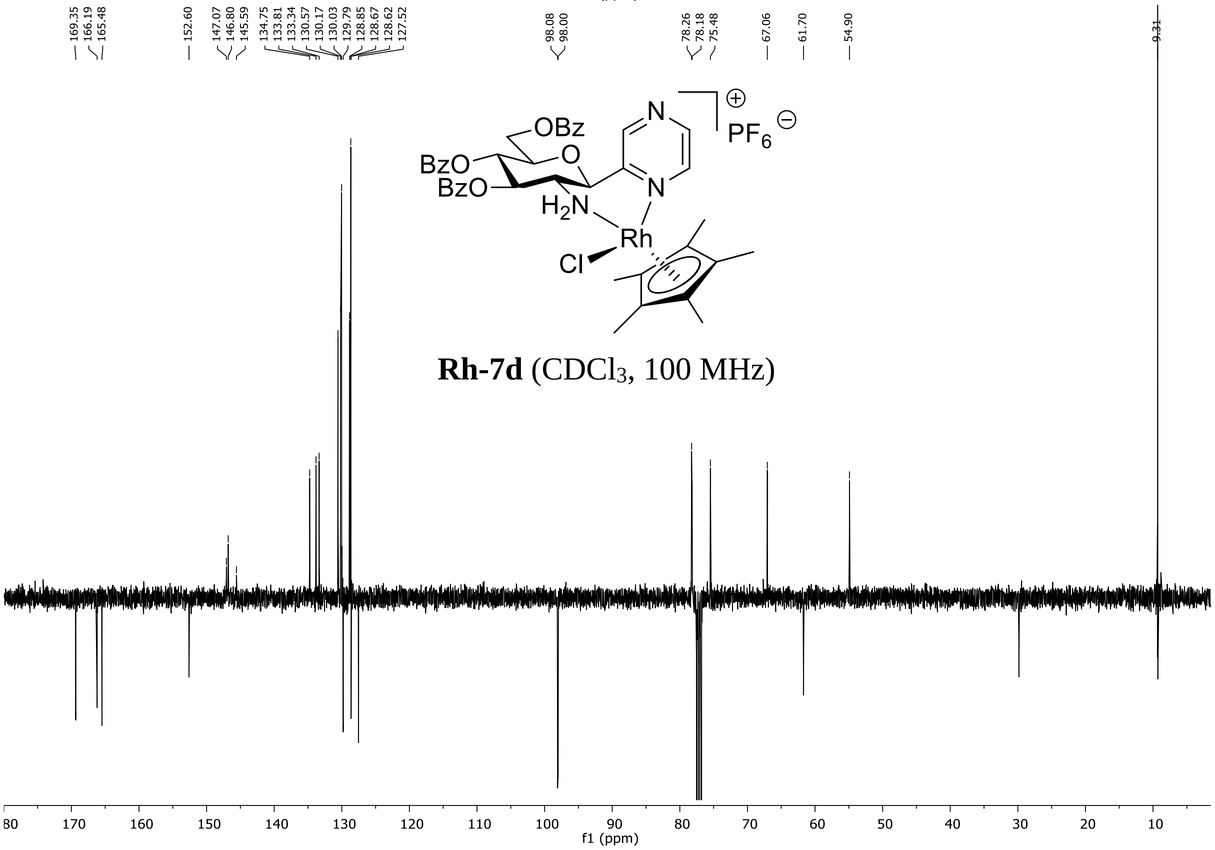

Rh-7d ($CDCl_3$, 100 MHz)

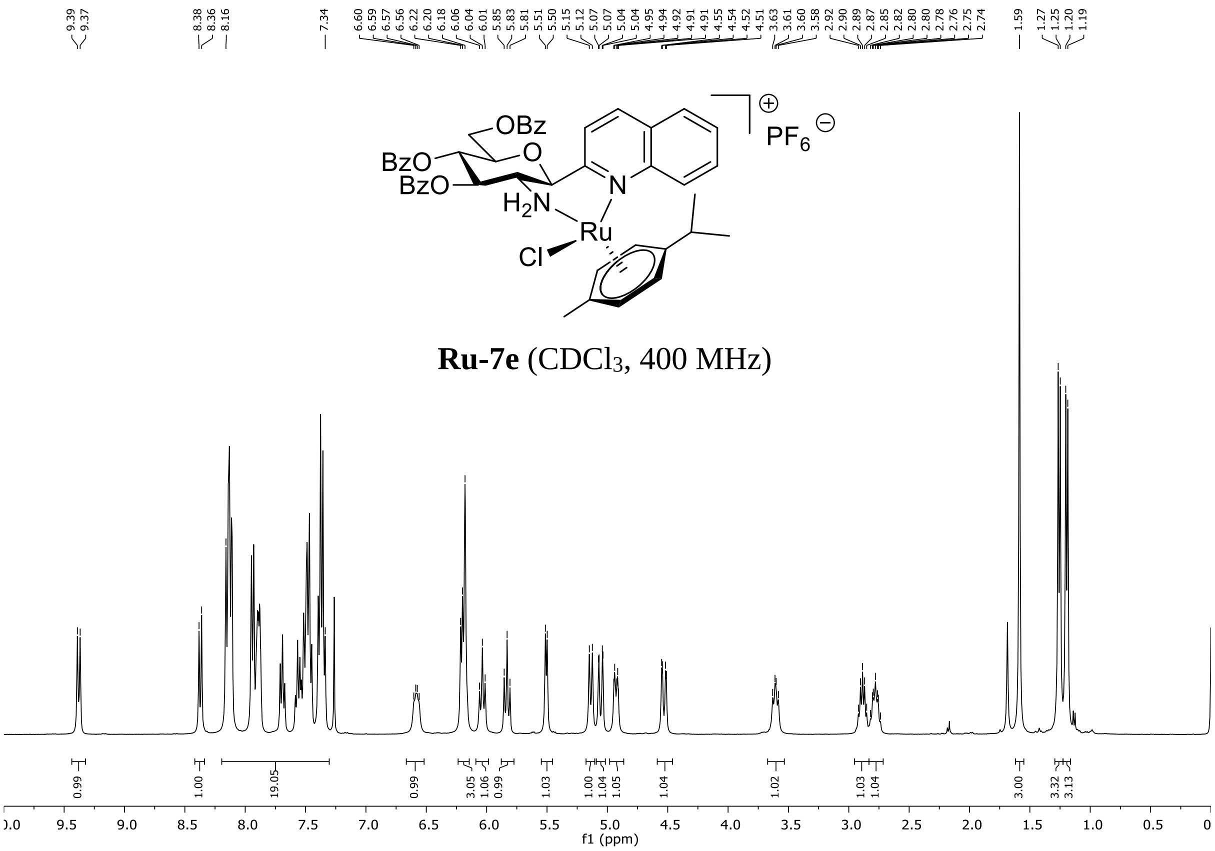

Ru-7e (CDCl3, 400 MHz)

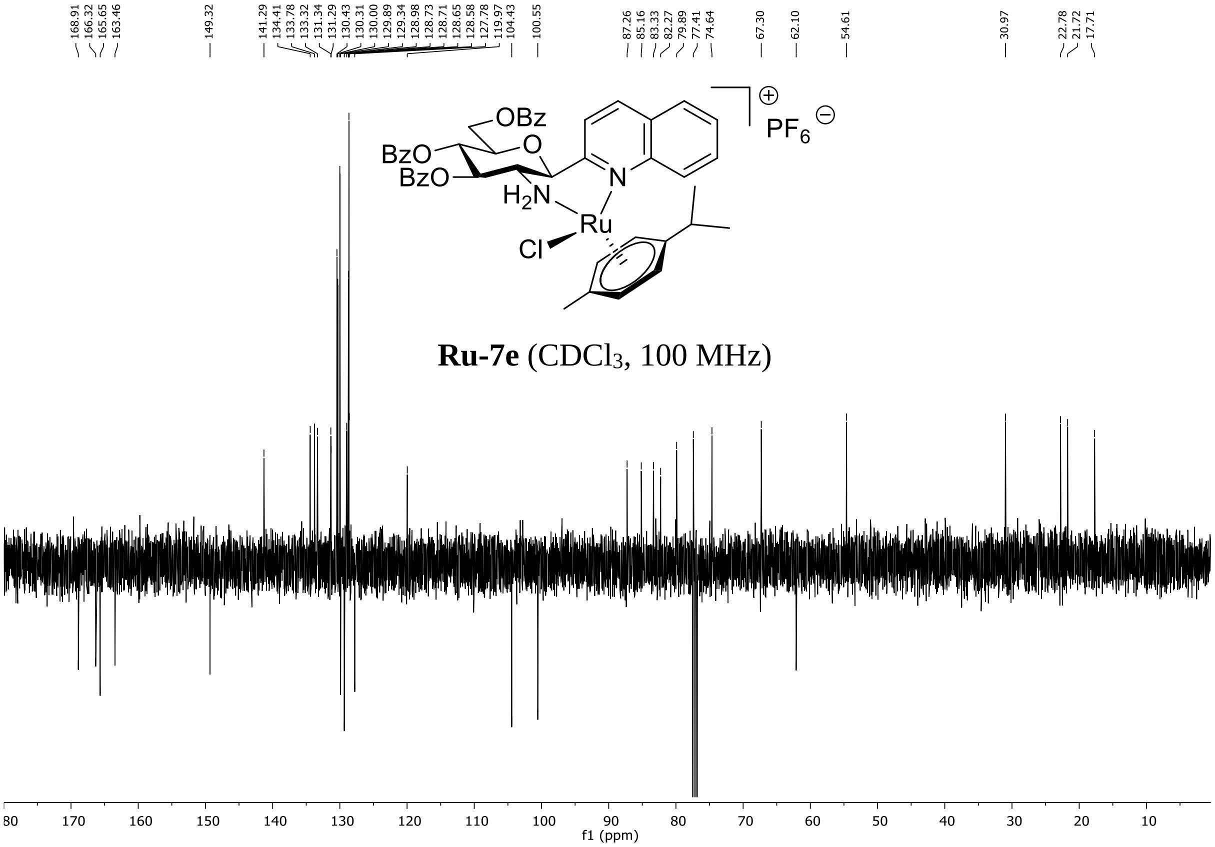

Ru-7e (CDCl3, 100 MHz)

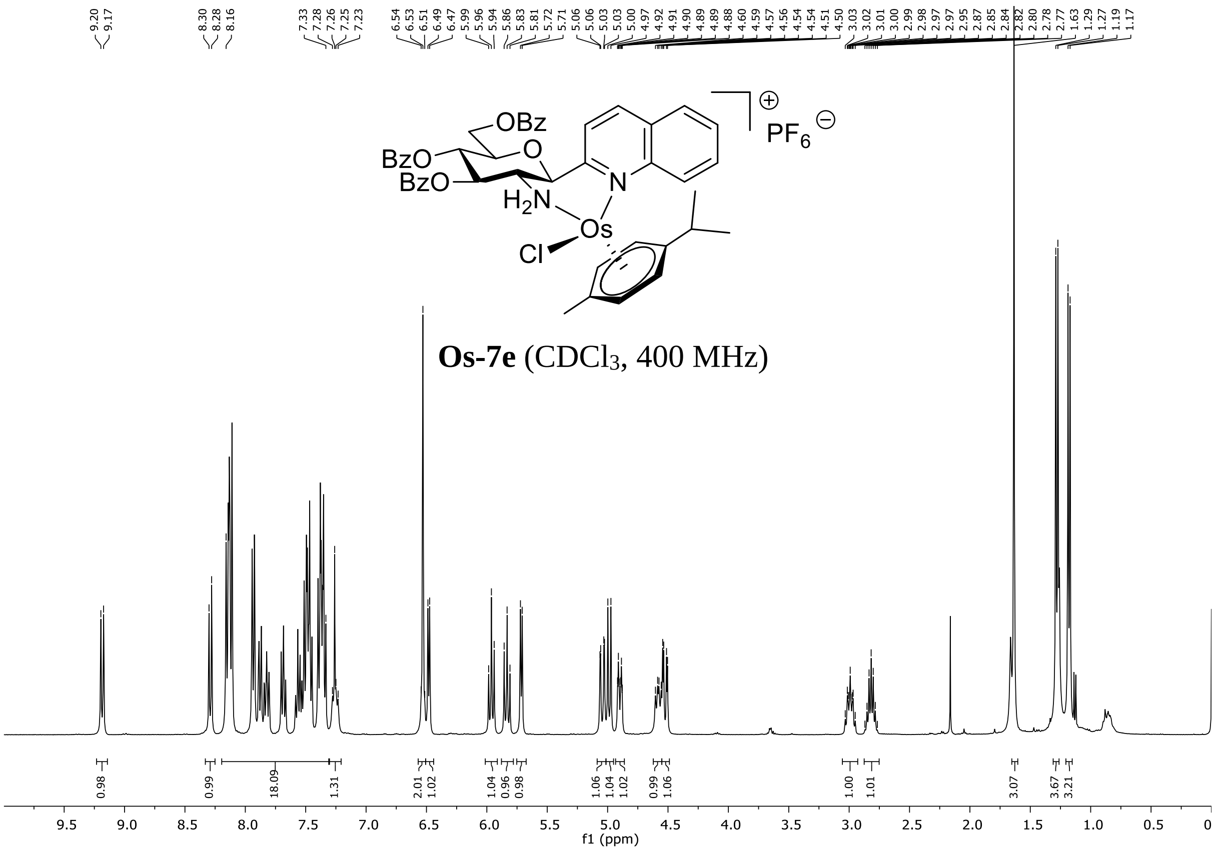
PF6
Os-7e (CDCl3, 400 MHz)
f1 (ppm)

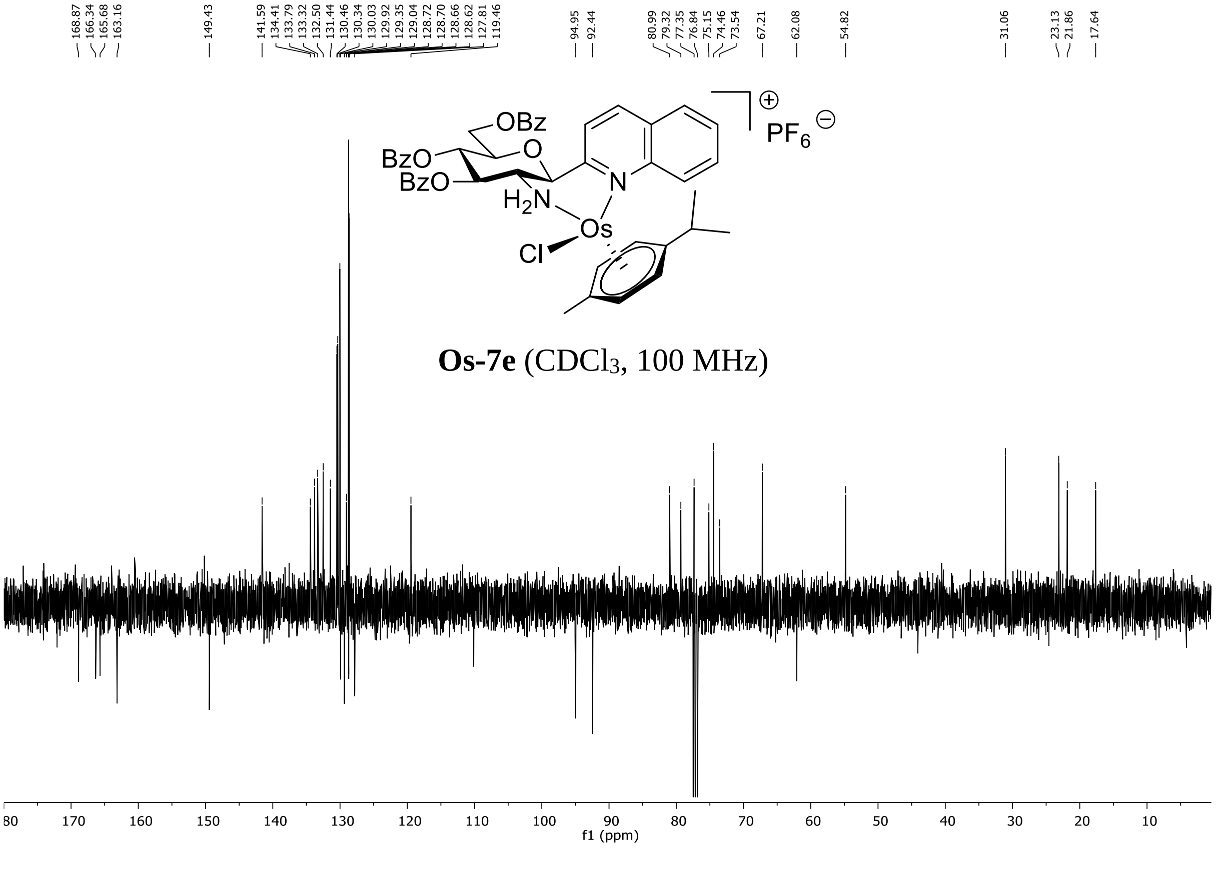
PF6
Os-7e (CDCl3, 100 MHz)
f1 (ppm)

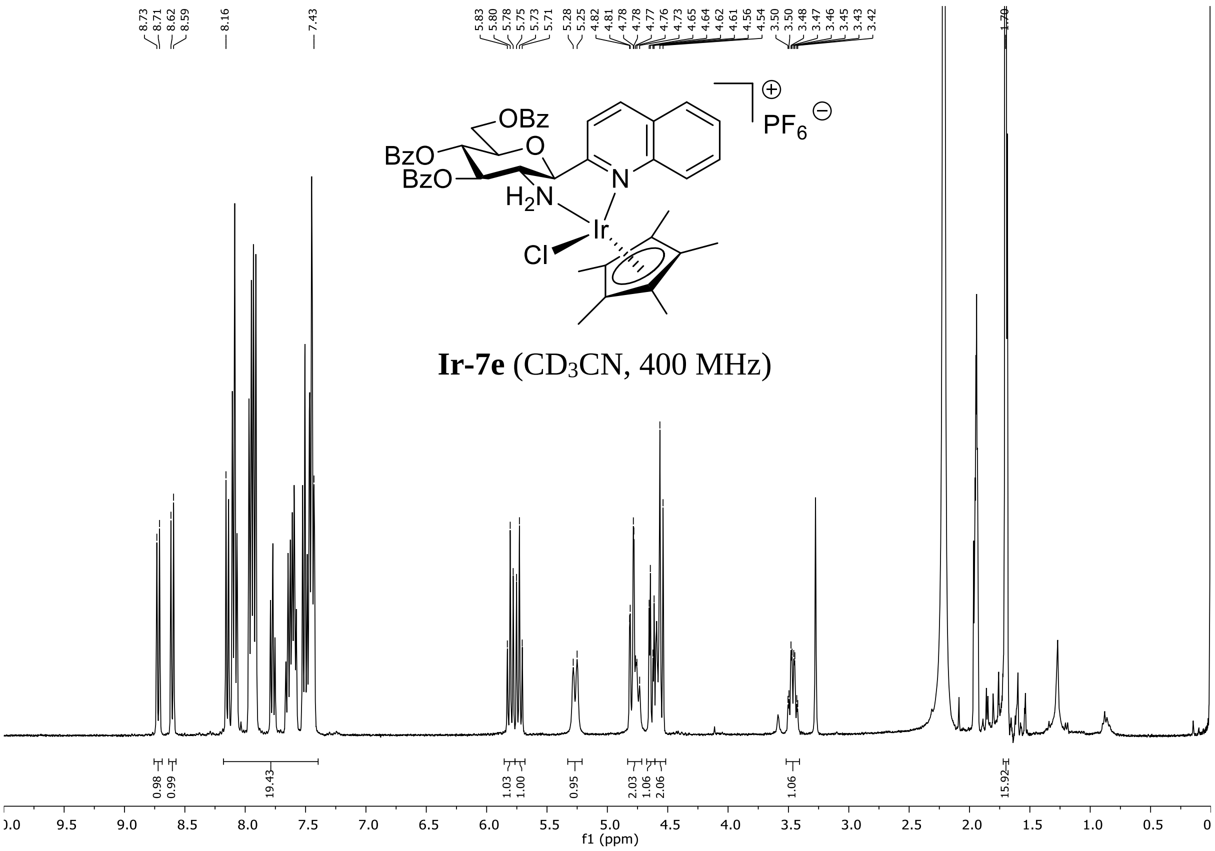
Ir-7e (CD3CN, 400 MHz)
OBz
BzO
BzO
O
N
H2N
Ir
Cl
PF6
f1 (ppm)

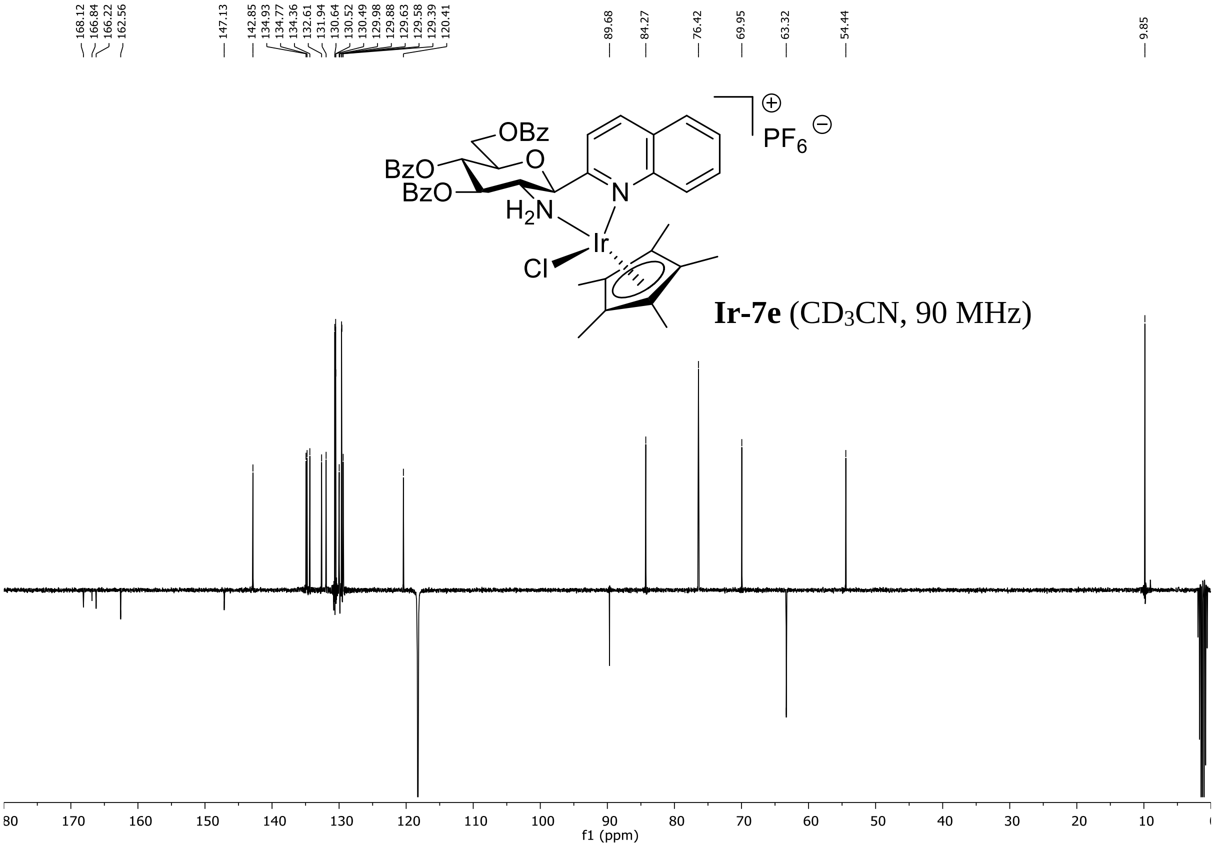
Ir-7e (CD3CN, 90 MHz)
OBz
BzO
BzO
O
N
H2N
Ir
Cl
PF6
f1 (ppm)

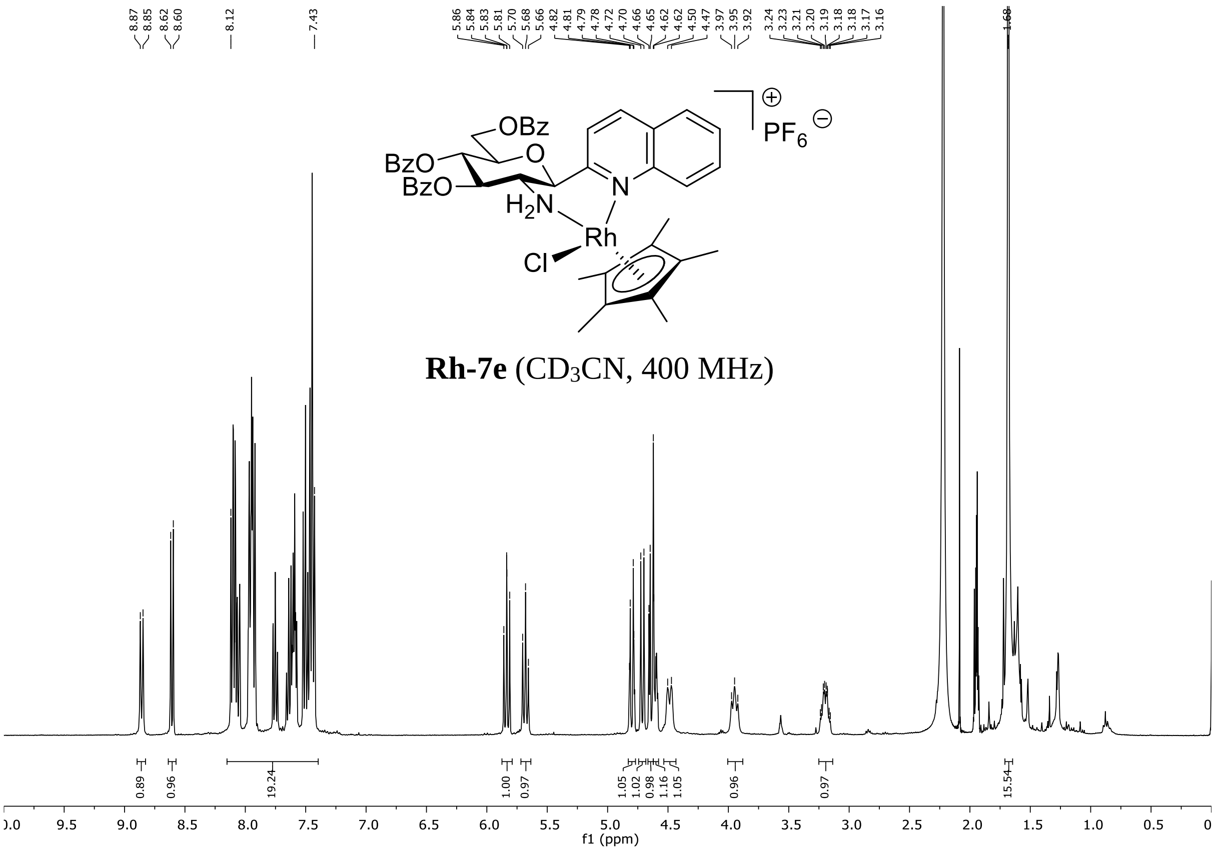

Rh-7e (CD3CN, 400 MHz)
BzO
OBz
PF6
H2N
Rh
Cl
f1 (ppm)

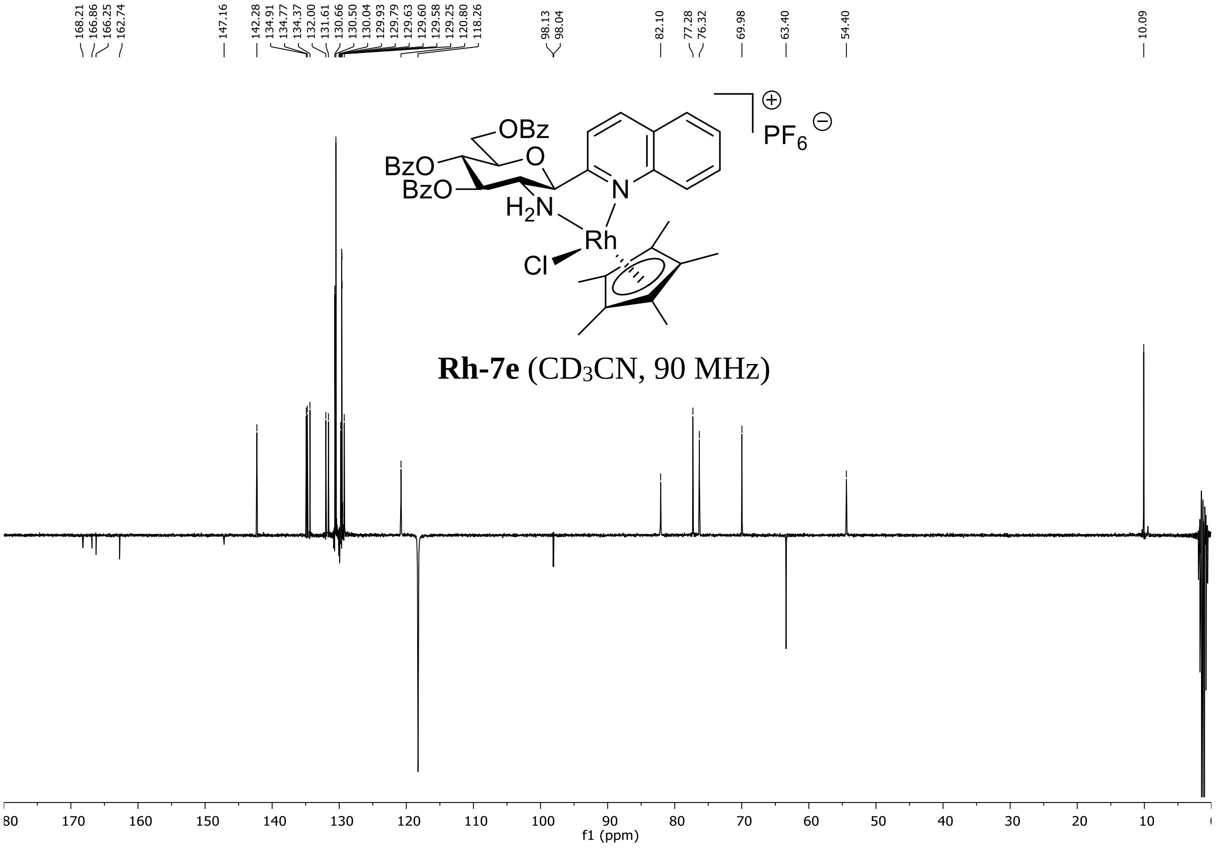

Rh-7e (CD3CN, 90 MHz)
BzO
OBz
PF6
H2N
Rh
Cl
f1 (ppm)

2. Table S1. Changes of the chemical shifts of selected ^{1}H NMR resonances as a result of the complex formation ($\Delta = \delta_{complex} – \delta_{ligand\ or\ dimer}$)

	H-6	H-5	H-4	H-3	H-6'a	H-6'b	H-5'	H-2'	H-1'	*i*-Pr-*CH*	Ar-*CH*$_3$
**Ru-3a (*R*)**	+0.46	+0.04	+0.12	+0.39	+0.03	+0.03	+0.29	-1.24	+0.27	-0.35	-0.46
**Os-3a (*R*)**	+0.31	+0.05	+0.11	+0.43	+0.03	+0.03	+0.29	-0.95	+0.23	-0.31	-0.46
**Ir-3a (*R*)**	+0.02	+0.06	+0.20	+0.43	+0.07	+0.01	+0.39	-0.71	-0.02	-	-0.18
**Rh-3a (*R*)**	+0.05	+0.08	+0.16	+0.45	+0.07	+0.01	+0.33	-0.86	-0.01	-	-0.19
**Ru-3d (*R*)**	+0.13	+0.41	-	+0.32	+0.02	+0.02	+0.26	-1.25	+0.36	-0.37	-0.40
**Os-3d (*R*)**	+0.04	+0.23	-	+0.33	+0.03	+0.03	+0.26	-0.96	+0.31	-0.32	-0.41
**Ru-7a (*R*)**	+0.42	-	-	-	+0.29	-0.03	+0.61	-0.97	+0.39	-0.04	-0.35
**Os-7a (*R*)**	+0.26	-	-	-	+0.36	-0.03	+0.59	-0.76	+0.35	+0.04	-0.36
**Ir-7a (*R*)**	+0.07	-	-	-	+0.45	+0.01	+0.59	-0.54	-0.04	-	+0.17
**Rh-7a (*R*)**	+0.08	-	-	-	+0.43	-0.01	+0.47	-0.74	0.00	-	+0.14
**Ru-7b (*R*)**	+0.01	-	+0.49	-	+0.32	-0.06	+0.41	-0.67	+0.41	-0.13	-0.31
**Ru-7b (*S*)**	-0.09	-	+0.47	-	+0.05	-0.05	+0.15	-0.36	+0.36	+0.08	+0.12
**Os-7b (*R*)**	-0.09	-	+0.52	-	+0.33	-0.04	+0.43	-0.46	+0.30	-0.01	-0.28
**Os-7b (*S*)**	-0.24	-	+0.58	-	+0.08	-0.04	+0.13	-0.23	+0.67	+0.10	+0.05
**Ir-7b (R)**	-0.08	-	+0.62	-	+0.34	-0.04	+0.48	-0.15	+0.07	-	+0.10
**Ir-7b (*S*)**	-0.21	-	+0.57	-	+0.07	+0.02	+0.02	+0.16	+1.14	-	-0.02
**Rh-7b (*R*)**	0.00	-	+0.56	-	+0.30	-0.04	+0.46	-0.29	+0.25	-	+0.11
**Rh-7b (*S*)**	-0.07	-	+0.45	-	-0.02	-0.02	-0.06	-0.03	+1.19	-	+0.03
**Ru-7c (*R*)**	+0.49	-	+0.09	-	+0.22	+0.09	+0.44	-0.89	+0.44	-0.18	-0.44
**Os-7c (*R*)**	+0.37	-	+0.02	-	+0.22	+0.09	+0.42	-0.74	+0.36	-0.10	-0.46
**Ir-7c (*R*)**	0.00	-	+0.14	-	+0.09	+0.18	+0.40	-0.55	+0.02	-	+0.15
**Rh-7c (*R*)**	+0.08	-	+0.18	-	+0.06	+0.15	+0.37	-0.71	+0.05	-	+0.19
**Ru-7d (*R*)**	+0.19	+0.45	-	+0.27	+0.33	-0.03	+0.48	-0.96	+0.41	-0.12	-0.39
**Os-7d (*R*)**	+0.10	+0.30	-	+0.27	+0.34	-0.02	+0.47	-0.72	+0.32	-0.04	-0.41
**Ir-7d (*R*)**	+0.19	-0.03	-	+0.36	+0.38	-0.01	+0.46	-0.51	-0.06	-	+0.14
**Rh-7d (*R*)**	+0.26	+0.04	-	+0.37	+0.40	0.00	+0.47	-0.66	+0.07	-	+0.16
**Ru-7e (*R*)**	-	-	-	-	+0.39	-0.01	+0.62	-0.92	+0.41	-0.04	-0.57
**Os-7e (*R*)**	-	-	-	-	+0.38	-0.02	+0.59	-0.70	+0.26	+0.04	-0.58
**Ru-5a (*R*)**	+0.52	+0.11	+0.14	+0.32	+0.07	+0.06	+0.17	-1.01	-0.16	+0.05	-0.29

3. **Table S2.** Distribution coefficient of the synthesized complexes (logD)*

Complex		logD*	Complex		logD*
**Ru-3a**	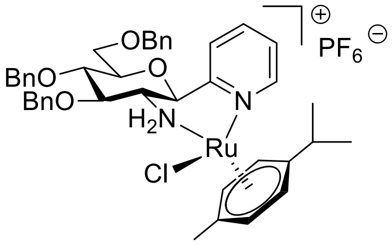	+2.72	**Os-3a**	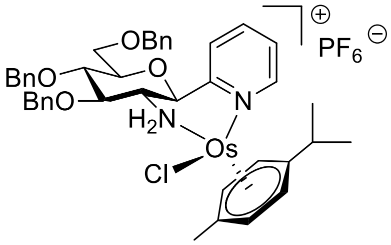	+1.78
**Ir-3a**	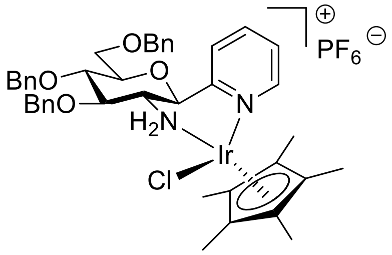	+3.18	**Rh-3a**	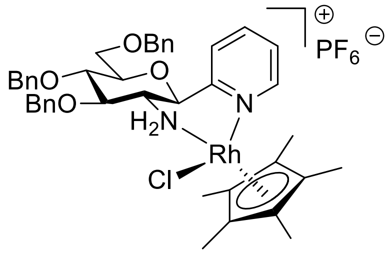	+1.64
**Ru-3d**	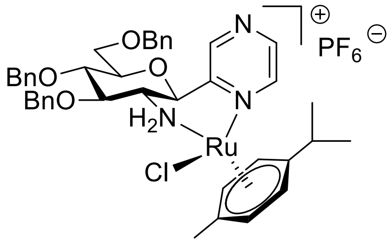	+2.06	**Os-3d**	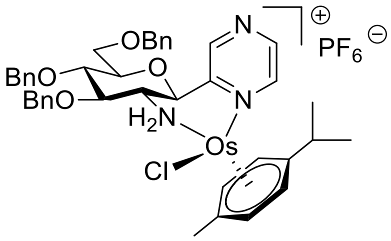	+2.64
**Ru-5a**	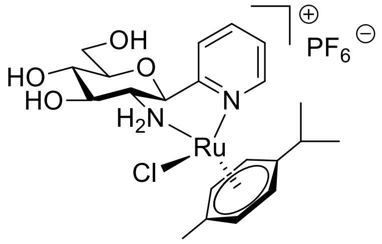	-1.91	-	-	-
**Ru-7a**	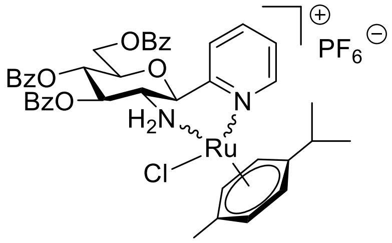	+1.15	**Os-7a**	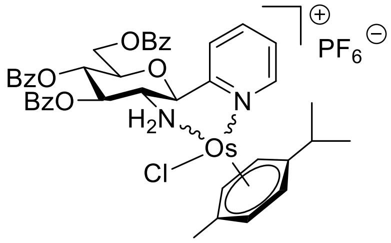	+2.15
**Ir-7a**	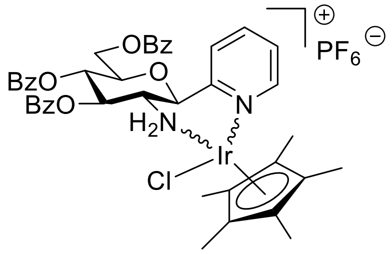	+1.08	**Rh-7a**	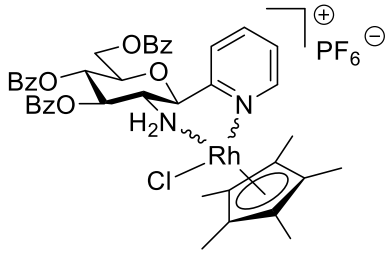	+1.20
**Ru-7b**	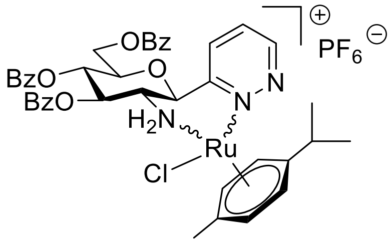	+1.39	**Os-7b**	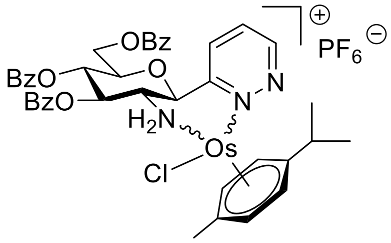	+1.32

**Ir-7b**	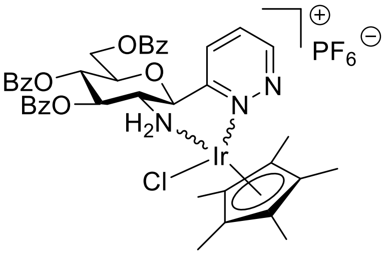	+1.17	**Rh-7b**	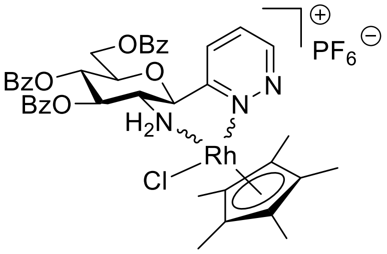	+1.41
**Ru-7c**	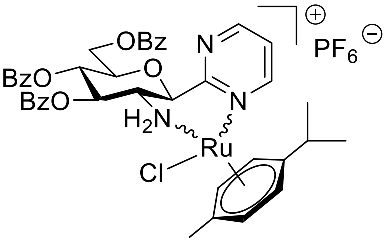	+1.31	**Os-7c**	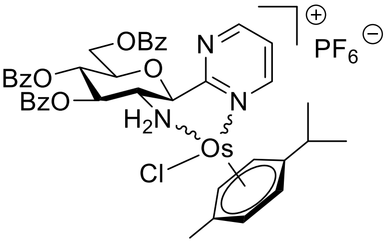	+1.59
**Ir-7c**	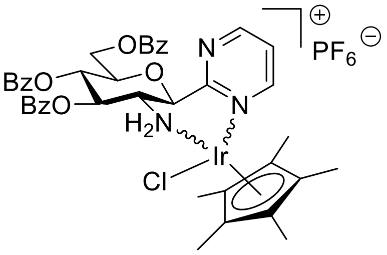	+1.40	**Rh-7c**	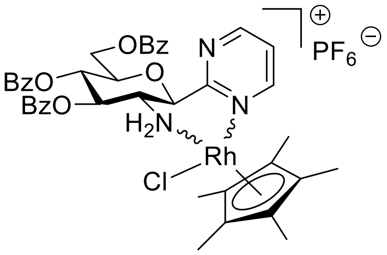	+1.13
**Ru-7d**	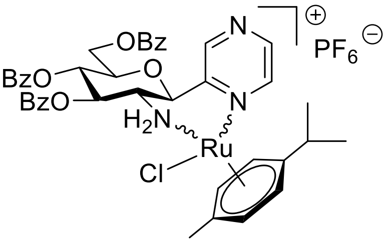	+1.26	**Os-7d**	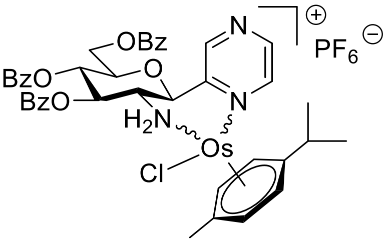	+1.42
**Ir-7d**	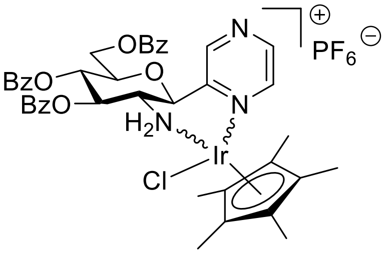	+1.30	**Rh-7d**	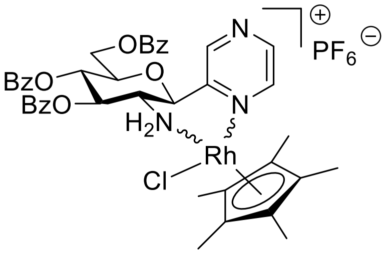	+1.04
**Ru-7e**	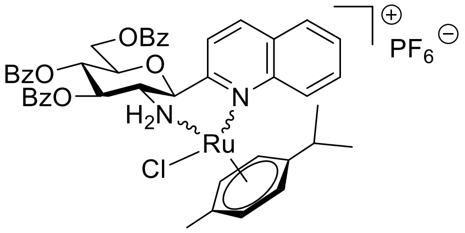	+2.22	**Os-7e**	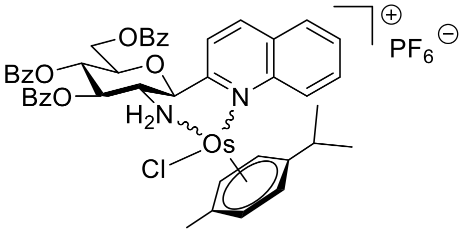	+2.21
**Ir-7e**	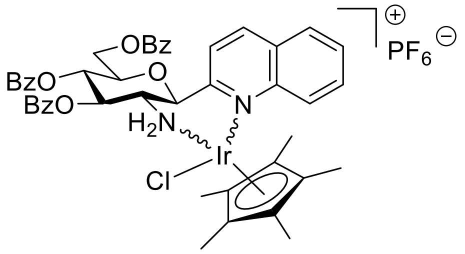	+1.39	**Rh-7e**	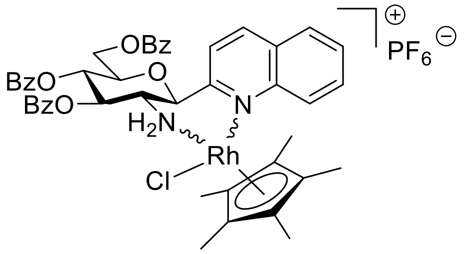	+1.66

*logD was determined in an *n*-octanol-PBS (pH = 7.4) system

4. X-Ray diffraction study of Ru-3a

General information

X-Ray-quality crystals of **Ru-3a** were grown by slow evaporation of a $CHCl_3$-MeOH solvent mixture. A crystal well-looking in polarized light microscope was fixed under a microscope onto a Mitegen loop using high-density oil. Diffraction intensity data were collected at room temperature using a Bruker-D8 Venture diffractometer (Bruker AXS GmbH, Karlsruhe, Germany) equipped with INCOATEC IµS 3.0 (Incoatec GmbH, Geesthacht, Germany) dual (Cu and Mo) sealed tube micro sources and a Photon II Charge-Integrating Pixel Array detector (Bruker AXS GmbH, Karlsruhe, Germany) using Mo Kα (λ = 0.71073 Å) radiation. High multiplicity data collection and integration were performed using APEX4 (version 2021-4.0, Bruker AXS Inc., 2021, Madison, USA) software. Data reduction and multi-scan absorption correction were performed using SAINT (version 8.40B, Bruker AXS Inc., 2019, Madison, USA). The structure was solved using direct methods and refined on F^2 using the SHELXL program [1] incorporated into the APEX4 suite. Refinement was performed anisotropically for all non-hydrogen atoms. Hydrogen atoms were placed into geometric positions except the amino protons which could be found on the difference electron density map and the respective N-H distances should be restrained. Multi-scan absorption correction had to be applied because of the presence of heavy atoms and shape of the crystal. Further experimental details are shown in Table S3. The CIF file was manually edited using Publcif software [2], while graphics were prepared using the Mercury program [3]. The results for the X-ray diffraction structure determinations were good enough and acceptable according to the Checkcif functionality of PLATON software (Utrecht University, Utrecht, The Netherlands) [4], and structural parameters such as bond length and angle data were in the expected range (Table S5) and except the short Ru-Cl distance. The solid state structure is stabilized by strong N-H..Cl and N-H..F as well as weak C-H..Cl hydrogen bonds (Figure S2 and Table S4).

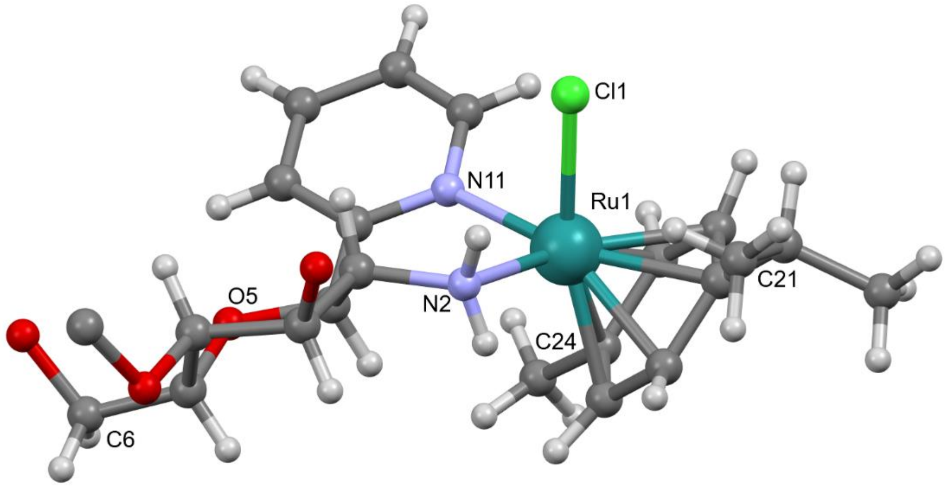

Figure S1. Ball and stick model of **Ru-3a** with partial numbering scheme. The structure is truncated for the better visibility of the coordination geometry.

Table S3. Experimental details

Crystal data	
Chemical formula	$C_{42}H_{48}ClF_6N_2O_4PRu$
M_r	926.31
Crystal system, space group	Monoclinic, $P2_1$
Temperature (K)	294
a, *b*, *c* (Å)	11.6071 (16), 16.655 (2), 11.7541 (19)
β (°)	113.396 (3)
V (Å^3)	2085.4 (5)
Z	2
Radiation type	Mo $K\alpha$
μ (mm^{-1})	0.55
Crystal size (mm)	0.26 × 0.24 × 0.05
Data collection	
Diffractometer	Bruker D8 VENTURE
Absorption correction	Multi-scan<br>*SADABS2016*/2 - Bruker AXS area detector scaling and absorption correction
T_{min}, T_{max}	0.87, 0.97
No. of measured, independent and observed [$I > 2\sigma(I)$] reflections	36696, 7605, 4883
R_{int}	0.171
$(\sin \theta/\lambda)_{max}$ (Å^{-1})	0.603
Refinement	
$R[F^2 > 2\sigma(F^2)]$, $wR(F^2)$, S	0.083, 0.185, 1.07
No. of reflections	7605
No. of parameters	524
No. of restraints	3
H-atom treatment	H atoms treated by a mixture of independent and constrained refinement
$\Delta\rangle_{max}$, $\Delta\rangle_{min}$ (e Å^{-3})	0.59, -0.78
Absolute structure	Flack x determined using 1527 quotients [(I+)-(I-)]/[(I+)+(I-)] [5]
Absolute structure parameter	0.05 (5)

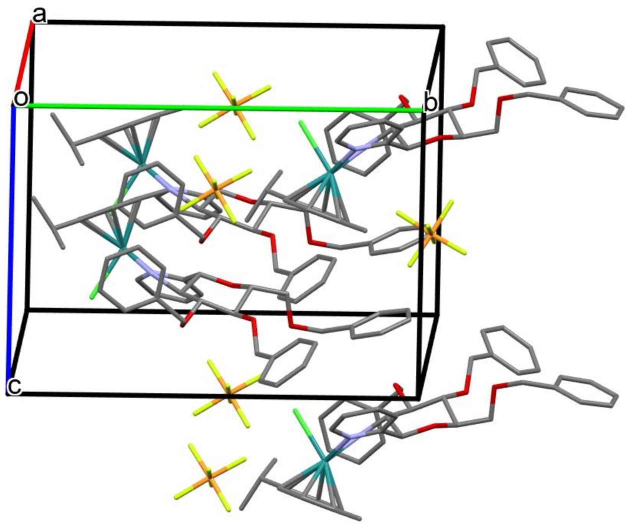

Figure S2. Packing diagram of **Ru-3a**. Hydrogen atoms are omitted for clarity.

Table S4. Hydrogen-bond geometry (Å, º) for **Ru-3a**

D—H···*A*	*D*—H	H···*A*	*D*···*A*	*D*—H···*A*
C37—H37*A*···O4	0.97	2.56	3.15 (2)	119
C47—H47*A*···Cl1^{i}	0.97	2.93	3.868 (19)	162
C67—H67*B*···Cl1^{i}	0.97	2.92	3.56 (2)	124
N2—H2*A*···Cl1	0.87 (3)	2.53 (17)	2.987 (15)	114 (14)
N2—H2*B*···F5	0.87 (3)	2.56 (9)	3.37 (2)	155 (17)
Symmetry code: (i) -*x*+1, *y*+1/2, -*z*+2.				

Table S5. Geometric parameters (Å, º) for **Ru-3a**

Distances, Å			
C1—O5	1.428 (16)	C30—H30C	0.9600
C1—C12	1.516 (19)	C31—C32	1.33 (3)
C1—C2	1.536 (19)	C31—C36	1.38 (3)
C1—H1A	0.9800	C31—C37	1.50 (3)
C3—O3	1.431 (18)	C32—C33	1.43 (3)
C3—C4	1.52 (2)	C32—H32	0.9300
C3—C2	1.527 (19)	C33—C34	1.34 (4)
C3—H3	0.9800	C33—H33	0.9300
C4—O4	1.438 (19)	C34—C35	1.38 (4)
C4—C5	1.53 (2)	C34—H34	0.9300
C4—H4	0.9800	C35—C36	1.41 (3)
C5—O5	1.455 (18)	C35—H35	0.9300
C5—C6	1.50 (2)	C36—H36	0.9300
C5—H5	0.9800	C37—O3	1.44 (2)
C6—O6	1.38 (2)	C37—H37A	0.9700
C6—H6A	0.9700	C37—H37B	0.9700
C6—H6B	0.9700	C41—C42	1.34 (2)
C2—N2	1.48 (2)	C41—C46	1.40 (2)
C2—H2	0.9800	C41—C47	1.51 (2)
C12—N11	1.353 (19)	C42—C43	1.36 (3)
C12—C13	1.36 (2)	C42—H42	0.9300
C13—C14	1.38 (2)	C43—C44	1.37 (3)
C13—H13A	0.9300	C43—H43	0.9300
C14—C15	1.38 (2)	C44—C45	1.40 (3)
C14—H14A	0.9300	C44—H44	0.9300
C15—C16	1.35 (2)	C45—C46	1.41 (3)
C15—H15A	0.9300	C45—H45	0.9300
C16—N11	1.359 (18)	C46—H46	0.9300
C16—H16A	0.9300	C47—O4	1.41 (2)
C21—C22	1.38 (2)	C47—H47A	0.9700
C21—C26	1.40 (2)	C47—H47B	0.9700
C21—C27	1.53 (2)	C61—C62	1.38 (3)
C21—Ru1	2.184 (15)	C61—C66	1.39 (3)
C22—C23	1.40 (2)	C61—C67	1.49 (3)
C22—Ru1	2.184 (15)	C62—C63	1.38 (3)
C22—H22	0.9800	C62—H62	0.9300
C23—C24	1.43 (2)	C63—C64	1.40 (4)
C23—Ru1	2.177 (17)	C63—H63	0.9300

C23—H23	0.9800	C64—C65	1.35 (4)
C24—C25	1.38 (2)	C64—H64	0.9300
C24—C30	1.49 (2)	C65—C66	1.36 (3)
C24—Ru1	2.207 (18)	C65—H65	0.9300
C25—C26	1.43 (2)	C66—H66	0.9300
C25—Ru1	2.164 (18)	N2—Ru1	2.153 (13)
C25—H25	0.9800	N2—H2A	0.87 (3)
C26—Ru1	2.166 (17)	N2—H2B	0.87 (3)
C26—H26	0.9800	N11—Ru1	2.091 (14)
C27—C28	1.49 (3)	O6—C67	1.43 (2)
C27—C29	1.51 (2)	C67—H67A	0.9700
C27—H27	0.9800	C67—H67B	0.9700
C28—H28A	0.9600	F1—P1	1.505 (16)
C28—H28B	0.9600	F2—P1	1.569 (14)
C28—H28C	0.9600	F3—P1	1.551 (15)
C29—H29A	0.9600	F4—P1	1.573 (16)
C29—H29B	0.9600	F5—P1	1.599 (13)
C29—H29C	0.9600	F6—P1	1.533 (16)
C30—H30A	0.9600	Cl1—Ru1	2.374 (5)
C30—H30B	0.9600		
O5—C1—C12	104.9 (12)	C34—C35—H35	120.2000
O5—C1—C2	112.1 (11)	C36—C35—H35	120.2000
C12—C1—C2	110.3 (12)	C31—C36—C35	118 (2)
O5—C1—H1A	109.8000	C31—C36—H36	121.2000
C12—C1—H1A	109.8000	C35—C36—H36	121.2000
C2—C1—H1A	109.8000	O3—C37—C31	113.8 (15)
O3—C3—C4	111.7 (14)	O3—C37—H37A	108.8000
O3—C3—C2	105.7 (12)	C31—C37—H37A	108.8000
C4—C3—C2	109.8 (13)	O3—C37—H37B	108.8000
O3—C3—H3	109.9000	C31—C37—H37B	108.8000
C4—C3—H3	109.9000	H37A—C37—H37B	107.7000
C2—C3—H3	109.9000	C42—C41—C46	119.1 (17)
O4—C4—C3	108.7 (14)	C42—C41—C47	120.5 (17)
O4—C4—C5	109.1 (14)	C46—C41—C47	120.4 (17)
C3—C4—C5	107.2 (14)	C41—C42—C43	123 (2)
O4—C4—H4	110.6000	C41—C42—H42	118.4000
C3—C4—H4	110.6000	C43—C42—H42	118.4000
C5—C4—H4	110.6000	C42—C43—C44	119 (3)
O5—C5—C6	107.2 (14)	C42—C43—H43	120.4000
O5—C5—C4	105.1 (13)	C44—C43—H43	120.4000

C6—C5—C4	115.1 (14)	C43—C44—C45	121 (2)
O5—C5—H5	109.8000	C43—C44—H44	119.6000
C6—C5—H5	109.8000	C45—C44—H44	119.6000
C4—C5—H5	109.8000	C44—C45—C46	118 (2)
O6—C6—C5	111.6 (14)	C44—C45—H45	120.9000
O6—C6—H6A	109.3000	C46—C45—H45	120.9000
C5—C6—H6A	109.3000	C41—C46—C45	119 (2)
O6—C6—H6B	109.3000	C41—C46—H46	120.3000
C5—C6—H6B	109.3000	C45—C46—H46	120.3000
H6A—C6—H6B	108.0000	O4—C47—C41	108.8 (14)
N2—C2—C3	108.6 (13)	O4—C47—H47A	109.9000
N2—C2—C1	108.4 (12)	C41—C47—H47A	109.9000
C3—C2—C1	114.4 (12)	O4—C47—H47B	109.9000
N2—C2—H2	108.4000	C41—C47—H47B	109.9000
C3—C2—H2	108.4000	H47A—C47—H47B	108.3000
C1—C2—H2	108.4000	C62—C61—C66	118 (2)
N11—C12—C13	122.5 (15)	C62—C61—C67	122.3 (18)
N11—C12—C1	115.8 (14)	C66—C61—C67	119.2 (19)
C13—C12—C1	121.5 (14)	C61—C62—C63	121 (2)
C12—C13—C14	121.3 (18)	C61—C62—H62	119.4000
C12—C13—H13A	119.3000	C63—C62—H62	119.4000
C14—C13—H13A	119.3000	C62—C63—C64	118 (2)
C13—C14—C15	116.9 (18)	C62—C63—H63	120.8000
C13—C14—H14A	121.6000	C64—C63—H63	120.8000
C15—C14—H14A	121.6000	C65—C64—C63	120 (3)
C16—C15—C14	119.1 (17)	C65—C64—H64	120.0000
C16—C15—H15A	120.5000	C63—C64—H64	120.0000
C14—C15—H15A	120.5000	C64—C65—C66	121 (3)
C15—C16—N11	125.1 (16)	C64—C65—H65	119.3000
C15—C16—H16A	117.5000	C66—C65—H65	119.3000
N11—C16—H16A	117.5000	C65—C66—C61	120 (2)
C22—C21—C26	118.1 (15)	C65—C66—H66	119.8000
C22—C21—C27	120.1 (14)	C61—C66—H66	119.8000
C26—C21—C27	121.8 (15)	C2—N2—Ru1	120.2 (10)
C22—C21—Ru1	71.6 (9)	C2—N2—H2A	102 (10)
C26—C21—Ru1	70.5 (9)	Ru1—N2—H2A	100 (10)
C27—C21—Ru1	131.4 (12)	C2—N2—H2B	114 (10)
C21—C22—C23	121.9 (15)	Ru1—N2—H2B	82 (10)
C21—C22—Ru1	71.6 (9)	H2A—N2—H2B	137 (10)
C23—C22—Ru1	71.0 (9)	C12—N11—C16	115.1 (14)

C21—C22—H22	118.2000	C12—N11—Ru1	126.6 (11)
C23—C22—H22	118.2000	C16—N11—Ru1	118.2 (10)
Ru1—C22—H22	118.2000	C3—O3—C37	113.5 (15)
C22—C23—C24	120.3 (15)	C47—O4—C4	111.6 (14)
C22—C23—Ru1	71.5 (9)	C1—O5—C5	114.5 (11)
C24—C23—Ru1	72.0 (10)	C6—O6—C67	113.9 (14)
C22—C23—H23	119.4000	O6—C67—C61	115.2 (15)
C24—C23—H23	119.4000	O6—C67—H67A	108.5000
Ru1—C23—H23	119.4000	C61—C67—H67A	108.5000
C25—C24—C23	118.0 (16)	O6—C67—H67B	108.5000
C25—C24—C30	121.3 (16)	C61—C67—H67B	108.5000
C23—C24—C30	120.6 (17)	H67A—C67—H67B	107.5000
C25—C24—Ru1	69.9 (10)	N11—Ru1—N2	87.3 (5)
C23—C24—Ru1	69.8 (11)	N11—Ru1—C25	115.6 (6)
C30—C24—Ru1	128.1 (14)	N2—Ru1—C25	96.4 (6)
C24—C25—C26	120.7 (15)	N11—Ru1—C26	153.8 (5)
C24—C25—Ru1	73.3 (11)	N2—Ru1—C26	91.8 (6)
C26—C25—Ru1	70.8 (10)	C25—Ru1—C26	38.5 (6)
C24—C25—H25	119.2000	N11—Ru1—C23	93.7 (6)
C26—C25—H25	119.2000	N2—Ru1—C23	162.6 (6)
Ru1—C25—H25	119.2000	C25—Ru1—C23	67.5 (6)
C21—C26—C25	120.9 (15)	C26—Ru1—C23	79.7 (6)
C21—C26—Ru1	71.9 (9)	N11—Ru1—C22	121.4 (6)
C25—C26—Ru1	70.6 (10)	N2—Ru1—C22	149.9 (6)
C21—C26—H26	118.9000	C25—Ru1—C22	79.8 (6)
C25—C26—H26	118.9000	C26—Ru1—C22	66.6 (6)
Ru1—C26—H26	118.9000	C23—Ru1—C22	37.5 (6)
C28—C27—C29	113.1 (19)	N11—Ru1—C21	158.2 (5)
C28—C27—C21	108.2 (16)	N2—Ru1—C21	113.8 (5)
C29—C27—C21	113.7 (14)	C25—Ru1—C21	69.0 (6)
C28—C27—H27	107.2000	C26—Ru1—C21	37.6 (5)
C29—C27—H27	107.2000	C23—Ru1—C21	67.8 (6)
C21—C27—H27	107.2000	C22—Ru1—C21	36.9 (6)
C27—C28—H28A	109.5000	N11—Ru1—C24	91.2 (6)
C27—C28—H28B	109.5000	N2—Ru1—C24	124.5 (6)
H28A—C28—H28B	109.5000	C25—Ru1—C24	36.8 (7)
C27—C28—H28C	109.5000	C26—Ru1—C24	67.9 (7)
H28A—C28—H28C	109.5000	C23—Ru1—C24	38.2 (5)
H28B—C28—H28C	109.5000	C22—Ru1—C24	68.1 (6)
C27—C29—H29A	109.5000	C21—Ru1—C24	81.1 (6)

C27—C29—H29B	109.5000	N11—Ru1—Cl1	85.0 (4)
H29A—C29—H29B	109.5000	N2—Ru1—Cl1	82.4 (4)
C27—C29—H29C	109.5000	C25—Ru1—Cl1	159.3 (5)
H29A—C29—H29C	109.5000	C26—Ru1—Cl1	120.8 (5)
H29B—C29—H29C	109.5000	C23—Ru1—Cl1	115.0 (5)
C24—C30—H30A	109.5000	C22—Ru1—Cl1	90.9 (5)
C24—C30—H30B	109.5000	C21—Ru1—Cl1	92.4 (4)
H30A—C30—H30B	109.5000	C24—Ru1—Cl1	152.7 (5)
C24—C30—H30C	109.5000	F1—P1—F6	91.4 (13)
H30A—C30—H30C	109.5000	F1—P1—F3	91.8 (12)
H30B—C30—H30C	109.5000	F6—P1—F3	176.3 (12)
C32—C31—C36	122 (2)	F1—P1—F2	90.4 (10)
C32—C31—C37	121 (2)	F6—P1—F2	88.9 (9)
C36—C31—C37	117 (2)	F3—P1—F2	93.0 (10)
C31—C32—C33	121 (2)	F1—P1—F4	179.1 (13)
C31—C32—H32	119.6000	F6—P1—F4	89.5 (12)
C33—C32—H32	119.6000	F3—P1—F4	87.3 (12)
C34—C33—C32	118 (3)	F2—P1—F4	89.9 (9)
C34—C33—H33	121.2000	F1—P1—F5	92.7 (10)
C32—C33—H33	121.2000	F6—P1—F5	89.0 (9)
C33—C34—C35	122 (3)	F3—P1—F5	88.9 (9)
C33—C34—H34	118.9000	F2—P1—F5	176.3 (10)
C35—C34—H34	118.9000	F4—P1—F5	87.0 (8)
C34—C35—C36	120 (2)		
O3—C3—C4—O4	-65.5 (19)	Ru1—C21—C27—C28	178.6 (14)
C2—C3—C4—O4	177.6 (14)	C22—C21—C27—C29	143.4 (19)
O3—C3—C4—C5	176.6 (13)	C26—C21—C27—C29	-39 (3)
C2—C3—C4—C5	59.7 (19)	Ru1—C21—C27—C29	52 (2)
O4—C4—C5—O5	173.5 (13)	C36—C31—C32—C33	3 (3)
C3—C4—C5—O5	-68.9 (17)	C37—C31—C32—C33	-179.7 (18)
O4—C4—C5—C6	56 (2)	C31—C32—C33—C34	-3 (3)
C3—C4—C5—C6	173.5 (15)	C32—C33—C34—C35	3 (4)
O5—C5—C6—O6	-71.3 (17)	C33—C34—C35—C36	-3 (4)
C4—C5—C6—O6	45 (2)	C32—C31—C36—C35	-3 (3)
O3—C3—C2—N2	72.6 (16)	C37—C31—C36—C35	179.6 (18)
C4—C3—C2—N2	-166.8 (14)	C34—C35—C36—C31	3 (3)
O3—C3—C2—C1	-166.1 (13)	C32—C31—C37—O3	-106 (2)
C4—C3—C2—C1	-46 (2)	C36—C31—C37—O3	71 (2)
O5—C1—C2—N2	161.6 (13)	C46—C41—C42—C43	1 (3)

C12—C1—C2—N2	-81.9 (16)	C47—C41—C42—C43	-178.1 (18)
O5—C1—C2—C3	40.2 (18)	C41—C42—C43—C44	-1 (3)
C12—C1—C2—C3	156.7 (14)	C42—C43—C44—C45	2 (3)
O5—C1—C12—N11	176.6 (13)	C43—C44—C45—C46	-3 (3)
C2—C1—C12—N11	55.8 (17)	C42—C41—C46—C45	-1 (3)
O5—C1—C12—C13	2.9 (19)	C47—C41—C46—C45	177.5 (18)
C2—C1—C12—C13	-118.0 (16)	C44—C45—C46—C41	2 (3)
N11—C12—C13—C14	3 (3)	C42—C41—C47—O4	-46 (2)
C1—C12—C13—C14	175.9 (15)	C46—C41—C47—O4	134.7 (17)
C12—C13—C14—C15	-2 (3)	C66—C61—C62—C63	3 (3)
C13—C14—C15—C16	0 (3)	C67—C61—C62—C63	180 (2)
C14—C15—C16—N11	1 (3)	C61—C62—C63—C64	-3 (4)
C26—C21—C22—C23	2 (2)	C62—C63—C64—C65	2 (4)
C27—C21—C22—C23	179.9 (16)	C63—C64—C65—C66	0 (5)
Ru1—C21—C22—C23	-52.3 (15)	C64—C65—C66—C61	-1 (4)
C26—C21—C22—Ru1	54.8 (14)	C62—C61—C66—C65	-1 (3)
C27—C21—C22—Ru1	-127.8 (15)	C67—C61—C66—C65	-177 (2)
C21—C22—C23—C24	-3 (3)	C3—C2—N2—Ru1	165.5 (10)
Ru1—C22—C23—C24	-55.1 (15)	C1—C2—N2—Ru1	40.7 (16)
C21—C22—C23—Ru1	52.5 (15)	C13—C12—N11—C16	-1 (2)
C22—C23—C24—C25	2 (3)	C1—C12—N11—C16	-174.7 (12)
Ru1—C23—C24—C25	-52.4 (15)	C13—C12—N11—Ru1	-177.2 (12)
C22—C23—C24—C30	178.0 (17)	C1—C12—N11—Ru1	9.1 (19)
Ru1—C23—C24—C30	123.2 (18)	C15—C16—N11—C12	-1 (2)
C22—C23—C24—Ru1	54.9 (15)	C15—C16—N11—Ru1	175.7 (14)
C23—C24—C25—C26	-2 (3)	C4—C3—O3—C37	98.5 (17)
C30—C24—C25—C26	-177.9 (17)	C2—C3—O3—C37	-142.2 (14)
Ru1—C24—C25—C26	-54.7 (15)	C31—C37—O3—C3	89 (2)
C23—C24—C25—Ru1	52.4 (15)	C41—C47—O4—C4	-172.2 (14)
C30—C24—C25—Ru1	-123.2 (18)	C3—C4—O4—C47	107.2 (17)
C22—C21—C26—C25	-2 (2)	C5—C4—O4—C47	-136.2 (16)
C27—C21—C26—C25	-179.8 (15)	C12—C1—O5—C5	-171.9 (12)
Ru1—C21—C26—C25	52.9 (15)	C2—C1—O5—C5	-52.2 (17)
C22—C21—C26—Ru1	-55.3 (14)	C6—C5—O5—C1	-170.4 (12)
C27—C21—C26—Ru1	127.3 (16)	C4—C5—O5—C1	66.7 (17)
C24—C25—C26—C21	2 (3)	C5—C6—O6—C67	165.4 (14)
Ru1—C25—C26—C21	-53.5 (14)	C6—O6—C67—C61	63 (2)
C24—C25—C26—Ru1	55.9 (16)	C62—C61—C67—O6	40 (3)
C22—C21—C27—C28	-90 (2)	C66—C61—C67—O6	-143.6 (19)
C26—C21—C27—C28	87 (2)		

5. References (the number of the reference list of the main text is given in parenthesis)

1.(80.) Sheldrick, G., A short history of SHELX. *Acta Crystallogr. A* **2008,** *64*, 112-122, Doi: 10.1107/S0108767307043930.

2.(81.) Westrip, S., publCIF: software for editing, validating and formatting crystallographic information files. *J. Appl. Cryst.* **2010,** *43*, 920-925, Doi: 10.1107/S0021889810022120.

3.(82.) Macrae, C. F.; Edgington, P. R.; McCabe, P.; Pidcock, E.; Shields, G. P.; Taylor, R.; Towler, M.; van de Streek, J., Mercury: visualization and analysis of crystal structures. *J. Appl. Cryst.* **2006,** *39*, 453-457, Doi: 10.1107/S002188980600731X.

4.(83.) Spek, A., Single-crystal structure validation with the program PLATON. *J. Appl. Cryst.* **2003,** *36*, 7-13, Doi: 10.1107/S0021889802022112.

5.(59.) Parsons, S.; Flack, H. D.; Wagner, T., Use of intensity quotients and differences in absolute structure refinement. *Acta Crystallogr. B* **2013,** *69*, 249-259, Doi: 10.1107/s2052519213010014.
