# Supplementary material for: Half-Sandwich Type Platinum-Group Metal Complexes of C-Glucosaminyl Azines: Synthesis and Antineoplastic and Antimicrobial Activities
Source: Molecules. 2023 Mar 29;28(7):3058. doi: 10.3390/molecules28073058 (PMC10096180; doi:10.3390/molecules28073058)

# checkCIF/PLATON report

Structure factors have been supplied for datablock(s) Ru_3a

THIS REPORT IS FOR GUIDANCE ONLY. IF USED AS PART OF A REVIEW PROCEDURE FOR PUBLICATION, IT SHOULD NOT REPLACE THE EXPERTISE OF AN EXPERIENCED CRYSTALLOGRAPHIC REFEREE.

No syntax errors found. CIF dictionary Interpreting this report

# Datablock: Ru_3a

---

```
Bond precision:   C-C = 0.0310 A          Wavelength=0.71073

Cell:            a=11.6071(16)    b=16.655(2)        c=11.7541(19)
                 alpha=90         beta=113.396(3)    gamma=90
Temperature:     294 K

                 Calculated                       Reported
Volume           2085.4(5)                        2085.4(5)
Space group      P 21                             P 1 21 1
Hall group       P 2yb                            P 2yb
Moiety formula   C42 H48 Cl N2 O4 Ru, F6 P   C42 H48 Cl F6 N2 O4 P Ru
Sum formula      C42 H48 Cl F6 N2 O4 P Ru    C42 H48 Cl F6 N2 O4 P Ru
Mr               926.31                           926.31
Dx,g cm-3        1.475                            1.475
Z                2                                2
Mu (mm-1)        0.548                            0.548
F000             952.0                            952.0
F000'            950.46
h,k,lmax         13,20,14                         13,20,14
Nref             7650[ 3968]                      7605
Tmin,Tmax        0.868,0.972                      0.870,0.970
Tmin'            0.868

Correction method= # Reported T Limits: Tmin=0.870 Tmax=0.970
AbsCorr = MULTI-SCAN

Data completeness= 1.92/0.99       Theta(max)= 25.370

R(reflections)= 0.0826( 4883)                     wR2(reflections)=
                                                  0.1847( 7605)
S = 1.065                   Npar= 524
```

The following ALERTS were generated. Each ALERT has the format
**test-name_ALERT_alert-type_alert-level.**
Click on the hyperlinks for more details of the test.

### Alert level B

```
PLAT342_ALERT_3_B Low Bond Precision on  C-C Bonds ..............    0.03098 Ang.
```

**Author Response: This error is probably related to the combined effect of the highly irregular plate morphology of the crystal (absorption) as well asthe presence of heavy element Ru. The structure is considered to be correct and no attempt was made to smash the error.**

### Alert level C

```
RINTA01_ALERT_3_C  The value of Rint is greater than 0.12
            Rint given   0.171
PLAT020_ALERT_3_C The Value of Rint is Greater Than 0.12 .........      0.171 Report
PLAT031_ALERT_4_C Refined Extinction Parameter Within Range of ...      3.143 Sigma
PLAT042_ALERT_1_C Calc. and Reported MoietyFormula Strings  Differ     Please Check
PLAT090_ALERT_3_C Poor Data / Parameter Ratio (Zmax > 18) ........       7.54 Note
PLAT241_ALERT_2_C High   'MainMol' Ueq as Compared to Neighbors of        C34 Check
PLAT241_ALERT_2_C High   'MainMol' Ueq as Compared to Neighbors of        C37 Check
PLAT241_ALERT_2_C High   'MainMol' Ueq as Compared to Neighbors of        C65 Check
PLAT242_ALERT_2_C Low    'MainMol' Ueq as Compared to Neighbors of        C31 Check
PLAT242_ALERT_2_C Low    'MainMol' Ueq as Compared to Neighbors of        C41 Check
PLAT260_ALERT_2_C Large Average Ueq of Residue Including        P1      0.141 Check
PLAT911_ALERT_3_C Missing FCF Refl Between Thmin & STh/L=    0.600          5 Report
PLAT973_ALERT_2_C Check Calcd Positive Resid. Density on       Ru1       1.13 eA-3
```

### Alert level G

```
PLAT002_ALERT_2_G Number of Distance or Angle Restraints on AtSite          3 Note
PLAT066_ALERT_1_G Predicted and Reported Tmin&Tmax Range Identical          ? Check
PLAT083_ALERT_2_G SHELXL Second Parameter in WGHT  Unusually Large       9.49 Why ?
PLAT172_ALERT_4_G The CIF-Embedded .res File Contains DFIX Records          1 Report
PLAT244_ALERT_4_G Low    'Solvent' Ueq as Compared to Neighbors of         P1 Check
PLAT303_ALERT_2_G Full Occupancy Atom H2B       with # Connections       2.00 Check
PLAT480_ALERT_4_G Long H...A H-Bond Reported H47A      ..CL1      .      2.93 Ang.
PLAT480_ALERT_4_G Long H...A H-Bond Reported H67B      ..CL1      .      2.92 Ang.
PLAT480_ALERT_4_G Long H...A H-Bond Reported H2B       ..F5       .      2.56 Ang.
PLAT721_ALERT_1_G Bond    Calc     0.97000, Rep     0.96000 Dev...       0.01 Ang.
              C28      -H28B           1_555   1_555 ........     #   52 Check
PLAT791_ALERT_4_G Model has Chirality at C1          (Sohnke SpGr)          R Verify
PLAT791_ALERT_4_G Model has Chirality at C2          (Sohnke SpGr)          S Verify
PLAT791_ALERT_4_G Model has Chirality at C3          (Sohnke SpGr)          R Verify
PLAT791_ALERT_4_G Model has Chirality at C4          (Sohnke SpGr)          S Verify
PLAT791_ALERT_4_G Model has Chirality at C5          (Sohnke SpGr)          R Verify
PLAT860_ALERT_3_G Number of Least-Squares Restraints .............          3 Note
PLAT912_ALERT_4_G Missing # of FCF Reflections Above STh/L=  0.600         15 Note
PLAT978_ALERT_2_G Number C-C Bonds with Positive Residual Density.          0 Info
```

```
 0 ALERT level A = Most likely a serious problem - resolve or explain
 1 ALERT level B = A potentially serious problem, consider carefully
13 ALERT level C = Check. Ensure it is not caused by an omission or oversight
18 ALERT level G = General information/check it is not something unexpected

 3 ALERT type 1 CIF construction/syntax error, inconsistent or missing data
11 ALERT type 2 Indicator that the structure model may be wrong or deficient
 6 ALERT type 3 Indicator that the structure quality may be low
12 ALERT type 4 Improvement, methodology, query or suggestion
 0 ALERT type 5 Informative message, check
```

It is advisable to attempt to resolve as many as possible of the alerts in all categories. Often the minor alerts point to easily fixed oversights, errors and omissions in your CIF or refinement strategy, so attention to these fine details can be worthwhile. In order to resolve some of the more serious problems it may be necessary to carry out additional measurements or structure refinements. However, the purpose of your study may justify the reported deviations and the more serious of these should normally be commented upon in the discussion or experimental section of a paper or in the "special_details" fields of the CIF. checkCIF was carefully designed to identify outliers and unusual parameters, but every test has its limitations and alerts that are not important in a particular case may appear. Conversely, the absence of alerts does not guarantee there are no aspects of the results needing attention. It is up to the individual to critically assess their own results and, if necessary, seek expert advice.

**Publication of your CIF in IUCr journals**

A basic structural check has been run on your CIF. These basic checks will be run on all CIFs submitted for publication in IUCr journals (*Acta Crystallographica*, *Journal of Applied Crystallography*, *Journal of Synchrotron Radiation*); however, if you intend to submit to *Acta Crystallographica Section C* or *E* or *IUCrData*, you should make sure that full publication checks are run on the final version of your CIF prior to submission.

**Publication of your CIF in other journals**

Please refer to the *Notes for Authors* of the relevant journal for any special instructions relating to CIF submission.

**PLATON version of 28/11/2022; check.def file version of 28/11/2022**

**Datablock Ru_3a** - ellipsoid plot

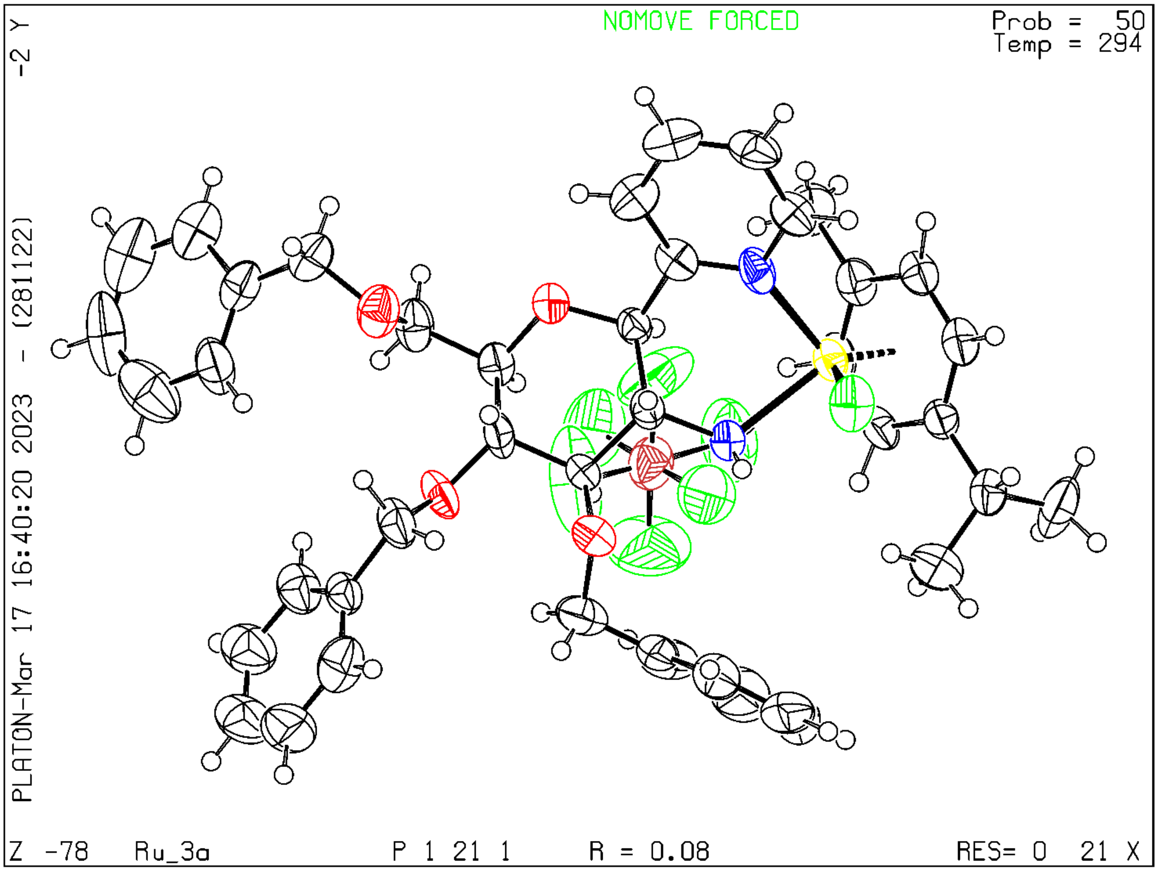

Supplement: Supplementary file 1 [file molecules-28-03058-s001.zip › Kaciretal_checkcif2.pdf]
